# The influence of obesity-related factors in the etiology of renal cell carcinoma—A mendelian randomization study

**DOI:** 10.1371/journal.pmed.1002724

**Published:** 2019-01-03

**Authors:** Mattias Johansson, Robert Carreras-Torres, Ghislaine Scelo, Mark P. Purdue, Daniela Mariosa, David C. Muller, Nicolas J. Timpson, Philip C. Haycock, Kevin M. Brown, Zhaoming Wang, Yuanqing Ye, Jonathan N. Hofmann, Matthieu Foll, Valerie Gaborieau, Mitchell J. Machiela, Leandro M. Colli, Peng Li, Jean-Guillaume Garnier, Helene Blanche, Anne Boland, Laurie Burdette, Egor Prokhortchouk, Konstantin G. Skryabin, Meredith Yeager, Sanja Radojevic-Skodric, Simona Ognjanovic, Lenka Foretova, Ivana Holcatova, Vladimir Janout, Dana Mates, Anush Mukeriya, Stefan Rascu, David Zaridze, Vladimir Bencko, Cezary Cybulski, Eleonora Fabianova, Viorel Jinga, Jolanta Lissowska, Jan Lubinski, Marie Navratilova, Peter Rudnai, Simone Benhamou, Geraldine Cancel-Tassin, Olivier Cussenot, Elisabete Weiderpass, Börje Ljungberg, Raviprakash Tumkur Sitaram, Christel Häggström, Fiona Bruinsma, Susan J. Jordan, Gianluca Severi, Ingrid Winship, Kristian Hveem, Lars J. Vatten, Tony Fletcher, Susanna C. Larsson, Alicja Wolk, Rosamonde E. Banks, Peter J. Selby, Douglas F. Easton, Gabriella Andreotti, Laura E. Beane Freeman, Stella Koutros, Satu Männistö, Stephanie Weinstein, Peter E. Clark, Todd L. Edwards, Loren Lipworth, Susan M. Gapstur, Victoria L. Stevens, Hallie Carol, Matthew L. Freedman, Mark M. Pomerantz, Eunyoung Cho, Kathryn M. Wilson, J. Michael Gaziano, Howard D. Sesso, Neal D. Freedman, Alexander S. Parker, Jeanette E. Eckel-Passow, Wen-Yi Huang, Richard J. Kahnoski, Brian R. Lane, Sabrina L. Noyes, David Petillo, Bin Tean Teh, Ulrike Peters, Emily White, Garnet L. Anderson, Lisa Johnson, Juhua Luo, Julie Buring, I-Min Lee, Wong-Ho Chow, Lee E. Moore, Timothy Eisen, Marc Henrion, James Larkin, Poulami Barman, Bradley C. Leibovich, Toni K. Choueiri, G. Mark Lathrop, Jean-Francois Deleuze, Marc Gunter, James D. McKay, Xifeng Wu, Richard S. Houlston, Stephen J. Chanock, Caroline Relton, J. Brent Richards, Richard M. Martin, George Davey Smith, Paul Brennan

**Affiliations:** 1 International Agency for Research on Cancer (IARC), Lyon, France; 2 Division of Cancer Epidemiology and Genetics, National Cancer Institute, National Institutes of Health, Department Health and Human Services, Bethesda, Maryland, United States of America; 3 Imperial College, London, United Kingdom; 4 MRC Integrative Epidemiology Unit, University of Bristol, Bristol, United Kingdom; 5 St. Jude Children's Research Hospital, Memphis, Tennessee, United States of America; 6 Department of Epidemiology, Division of Cancer Prevention and Population Sciences, The University of Texas MD Anderson Cancer Center, Houston, Texas, United States of America; 7 Max Planck Institute for Demographic Research, Rostock, Germany; 8 Centre National de Genotypage, Institut de Genomique, Centre de l'Energie Atomique et aux Energies Alternatives, Evry, France; 9 Fondation Jean Dausset - Centre d'Etude du Polymorphisme Humain, Paris, France; 10 Federal Research Centre “Fundamentals of Biotechnology” of the Russian Academy of Sciences, Moscow, Russian Federation; 11 Kurchatov Scientific Center, Moscow, Russian Federation; 12 Institute of Pathology, Medical School of Belgrade, Belgrade, Serbia; 13 Clinic of Urology, Clinical Center of Serbia, Belgrade, Serbia; 14 Mayo Clinic Graduate School of Biomedical Sciences, Rochester, Minnesota, United States of America; 15 International Organization for Cancer Prevention and Research (IOCPR), Belgrade, Serbia; 16 Department of Cancer Epidemiology and Genetics, Masaryk Memorial Cancer Institute, Brno, Czech Republic; 17 Institute of Public Health and Preventive Medicine, 2nd Faculty of Medicine, Charles University, Prague, Czech Republic; 18 Department of Preventive Medicine, Faculty of Medicine, Palacky University, Olomouc, Czech Republic; 19 National Institute of Public Health, Bucharest, Romania; 20 Russian N.N. Blokhin Cancer Research Centre, Moscow, Russian Federation; 21 Carol Davila University of Medicine and Pharmacy, Th. Burghele Hospital, Bucharest, Romania; 22 Institute of Hygiene and Epidemiology, 1st Faculty of Medicine, Charles University, Prague, Czech Republic; 23 International Hereditary Cancer Center, Department of Genetics and Pathology, Pomeranian Medical University, Szczecin, Poland; 24 Regional Authority of Public Health in Banska Bystrica, Banska Bystrica, Slovakia; 25 The M Sklodowska-Curie Cancer Center and Institute of Oncology, Warsaw, Poland; 26 National Public Health Center, National Directorate of Environmental Health, Budapest, Hungary; 27 INSERM U946, Paris, France; 28 CNRS UMR8200, Institute Gustave Roussy, Villejuif, France; 29 CeRePP, Paris, France; 30 UPMC Univ Paris 06, GRC n°5, Institut Universitaire de Cancérologie, Paris, France; 31 AP-HP, Department of Urology, Hopitaux Universitaires Est Parisien Tenon, Paris, France; 32 Department of Research, Cancer Registry of Norway, Institute of Population-Based Cancer Research, Oslo, Norway; 33 Department of Medical Epidemiology and Biostatistics, Karolinska Institutet, Stockholm, Sweden; 34 Genetic Epidemiology Group, Folkhälsan Research Center, Helsinki, Finland; 35 Department of Community Medicine, University of Tromsø, The Arctic University of Norway, Tromsø, Norway; 36 Department of Surgical and Perioperative Sciences, Urology and Andrology, Umeå University, Umeå, Sweden; 37 Department of Biobank Research, Umeå University, Umeå, Sweden; 38 Department of Surgical Sciences, Uppsala University, Uppsala, Sweden; 39 Cancer Epidemiology Centre, Cancer Council Victoria, Melbourne, Australia; 40 QIMR Berghofer Medical Research Institute, Herston, Queensland, Australia; 41 School of Public Health, The University of Queensland, Brisbane, Australia; 42 “Health across generations” team, CESP Inserm, Facultés de Médicine Université Paris-Sud, UVSQ, Université Paris-Saclay, Gustave Roussy, Villejuif, France; 43 Human Genetics Foundation (HuGeF), Torino, Italy; 44 Department of Medicine, Royal Melbourne Hospital, University of Melbourne, Melbourne, Australia; 45 K. G. Jebsen Center for Genetic Epidemiology, Department of Public Health, Norwegian University of Science and Technology, Trondheim, Norway; 46 Department of Public Health and General Practice, Faculty of Medicine, Norwegian University of Science and Technology, Trondheim, Norway; 47 London School of Hygiene and Tropical Medicine, University of London, London, United Kingdom; 48 Institute of Environmental Medicine, Karolinska Institutet, Stockholm, Sweden; 49 Leeds Institute of Cancer and Pathology, University of Leeds, St James's University Hospital, Leeds, United Kingdom; 50 National Institute for Health Research Diagnostic Evidence Cooperative, Division of Surgery, Imperial College London, St Mary’s Hospital, London, United Kingdom; 51 Department of Oncology, University of Cambridge, Cambridge, United Kingdom; 52 Department of Public Health and Primary Care, University of Cambridge, Cambridge, United Kingdom; 53 National Institute for Health and Welfare, Helsinki, Finland; 54 Vanderbilt-Ingram Cancer Center, Nashville, Tennessee, United States of America; 55 Department of Medicine, Division of Epidemiology, Vanderbilt-Ingram Cancer Center, Vanderbilt Genetics Institute, Nashville, Tennessee, United States of America; 56 American Cancer Society, Atlanta, Georgia, United States of America; 57 Dana-Farber Cancer Institute, Boston, Massachusetts, United States of America; 58 Brown University, Providence, Rhode Island, United States of America; 59 Harvard T.H. Chan School of Public Health, Boston, Massachusetts, United States of America; 60 Brigham and Women's Hospital, Harvard Medical School, Boston, Massachusetts, United States of America; 61 Department of Medicine, Brigham and Women’s Hospital, Boston, Massachusetts, United States of America; 62 Department of Health Sciences Research, Mayo Clinic, Jacksonville, Florida, United States of America; 63 Department of Health Sciences Research, Division of Biomedical Statistics and Informatics, Mayo Clinic, Rochester, Minnesota, United States of America; 64 Division of Urology, Spectrum Health, Grand Rapids, Michigan, United States of America; 65 College of Human Medicine, Michigan State University, Grand Rapids, Michigan, United States of America; 66 Van Andel Research Institute, Center for Cancer Genomics and Quantitative Biology, Grand Rapids, Michigan, United States of America; 67 Spectrum Health, Grand Rapids, Michigan, United States of America; 68 Diagnostics Program at Ferris State University, Grand Rapids, Michigan, United States of America; 69 Program in Cancer and Stem Cell Biology, Duke-National, University of Singapore Medical School, Singapore, Singapore; 70 Institute of Molecular and Cell Biology, A*STAR, Singapore, Singapore; 71 Laboratory of Cancer Epigenome, Division of Medical Sciences, National Cancer Centre Singapore, Singapore, Singapore; 72 Cancer Science Institute of Singapore, National University of Singapore, Singapore, Singapore; 73 Cancer Prevention Program, Fred Hutchinson Cancer Research Center, Seattle, Washington, United States of America; 74 WHI Clinical Coordinating Center, Fred Hutchinson Cancer Research Center, Seattle, Washington, United States of America; 75 Fred Hutchinson Cancer Research Center, Seattle, Washington, United States of America; 76 Department of Epidemiology and Biostatistics, School of Public Health Indiana University Bloomington, Bloomington, Indiana, United States of America; 77 University of Cambridge, Cambridge, United Kingdom; 78 The Institute of Cancer Research, London, United Kingdom; 79 Dept. of Genetics and Genomic Sciences, Icahn School of Medicine at Mount Sinai, New York, New York, United States of America; 80 Royal Marsden NHS Foundation Trust, London, United Kingdom; 81 Department of Urology, Mayo Clinic, Rochester, Minnesota, United States of America; 82 McGill University and Genome Quebec Innovation Centre, Montreal, Quebec, Canada; 83 School of Social and Community Medicine, University of Bristol, Bristol, United Kingdom; 84 Departments of Medicine, Human Genetics, Epidemiology and Biostatistics, Jewish General Hospital, McGill University, Montreal, Quebec, Canada; 85 University Hospitals Bristol NHS Foundation Trust National Institute for Health Research Bristol Nutrition Biomedical Research Unit, University of Bristol, Bristol, United Kingdom; Imperial College London, UNITED KINGDOM

## Abstract

**Background:**

Several obesity-related factors have been associated with renal cell carcinoma (RCC), but it is unclear which individual factors directly influence risk. We addressed this question using genetic markers as proxies for putative risk factors and evaluated their relation to RCC risk in a mendelian randomization (MR) framework. This methodology limits bias due to confounding and is not affected by reverse causation.

**Methods and findings:**

Genetic markers associated with obesity measures, blood pressure, lipids, type 2 diabetes, insulin, and glucose were initially identified as instrumental variables, and their association with RCC risk was subsequently evaluated in a genome-wide association study (GWAS) of 10,784 RCC patients and 20,406 control participants in a 2-sample MR framework. The effect on RCC risk was estimated by calculating odds ratios (OR_SD_) for a standard deviation (SD) increment in each risk factor. The MR analysis indicated that higher body mass index increases the risk of RCC (OR_SD_: 1.56, 95% confidence interval [CI] 1.44–1.70), with comparable results for waist-to-hip ratio (OR_SD_: 1.63, 95% CI 1.40–1.90) and body fat percentage (OR_SD_: 1.66, 95% CI 1.44–1.90). This analysis further indicated that higher fasting insulin (OR_SD_: 1.82, 95% CI 1.30–2.55) and diastolic blood pressure (DBP; OR_SD_: 1.28, 95% CI 1.11–1.47), but not systolic blood pressure (OR_SD_: 0.98, 95% CI 0.84–1.14), increase the risk for RCC. No association with RCC risk was seen for lipids, overall type 2 diabetes, or fasting glucose.

**Conclusions:**

This study provides novel evidence for an etiological role of insulin in RCC, as well as confirmatory evidence that obesity and DBP influence RCC risk.

## Abstract 2

Using mendelian randomization approaches, Paul Brennan and colleagues reveal an association between 12 obesity-related factors, including insulin and the development of renal cell carcinoma.

## Author summary

**Why was this study done?:**

- Traditional observational studies wherein putative risk factors are measured directly have found several obesity-related factors associated with increased risk of renal cell carcinoma (RCC).
- Traditional observational studies are subject to confounding and reverse causation and have not been able to disentangle which obesity-related risk factors directly influence RCC risk.
- This study used an alternative methodology commonly referred to as mendelian randomization (MR). MR circumvents many of the inherent limitations of traditional observational study by use of genetic proxies of putative risk factors when evaluating their associations with disease risk, as they are not subject to reverse causation and are less likely to be confounded by other risk factors.

**What did the researchers do and find?:**

- First, we used large-scale genome-wide association studies (GWAS) to identify genetic variants associated with obesity measures, blood pressure, lipids, type 2 diabetes, insulin, and glucose. Second, these genetic variants were used as proxies for the above-mentioned risk factors and evaluated in relation RCC risk using GWAS data from 10,000 RCC patients and 20,000 control participants.
- Based on these genetic data, we found that multiple measures of obesity, as well as diastolic blood pressure (DBP) and fasting insulin, are associated with RCC risk. In contrast, we found little evidence for an association with RCC risk for systolic blood pressure (SBP), circulating lipids, overall diabetes, or fasting glucose.

**What do these findings mean?:**

- This study provided robust and confirmatory evidence of an important role of obesity as an important risk factor of RCC.
- Further confirmatory evidence was found for elevated DBP as a risk factor of RCC, but it is not clear why DBP rather than SBP is important in RCC.
- The study, to our knowledge, provided novel evidence of an important role of circulating insulin in RCC etiology.
- This study provided some novel insights into the pathways involved in mediating the risk increase in RCC that is caused by obesity, most notably through insulin and DBP, but further research is needed to fully elucidate the important relationship between obesity and RCC.

## Introduction

The etiology of renal cell carcinoma (RCC) is only partly understood [1]. An increased risk of RCC has been observed for individuals with high body mass index (BMI), elevated blood pressure, and triglycerides [2]. However, these obesity-related exposures are inherently interrelated, and traditional epidemiological studies have not been able to untangle which individual factors directly influence RCC risk and which are merely correlated with the underlying causal factor.

Mendelian randomization (MR) is an analytical approach whereby germline genetic markers are used as proxies—or instrumental variables—for putative risk factors. These genetic markers cannot be influenced by reverse causation (i.e., the disease affecting the exposure) [3], and assuming an absence of pleiotropy (i.e., genetic variants associated with the disease through alternative pathways) can provide unconfounded estimates of disease risk [4]. MR-based studies can therefore circumvent many of the inherent limitations of traditional observational studies, and an association between genetic proxies and the disease of interest would indicate that the risk factor being proxied influences risk in a causal manner [5].

We evaluated the role of obesity-related factors in RCC etiology using a two-sample MR framework wherein genetic variants associated with 13 relevant risk factors were identified from genome-wide association studies (GWASs). Subsequently, we evaluated the association of these genetic variants with RCC risk in a large RCC GWAS comprising 10,784 RCC patients and 20,406 control participants.

## Materials and methods

### Analytical strategy

The goal of our analytical strategy was to clarify the role of obesity and obesity-related risk factors in RCC etiology using a two-sample MR framework. This involved reviewing the GWAS literature to identify obesity-related risk factors for which valid proxy single nucleotide polymorphisms (SNPs) could be identified or by carrying out de novo GWAS analyses (e.g., for risk factors measured in UK Biobank) (first sample). This led to assembling SNP-based instrumental variables for obesity-related factors that were evaluated in relation to risk in the largest RCC GWAS published to date (second sample) [6]. This MR-based risk analysis involved using the likelihood-based approach to estimate the RCC odds ratio (OR) associated with a standard deviation (SD) increment in each risk factor of interest, with several complementary MR methods being applied to evaluate consistency in association estimates and between-study heterogeneity. No changes to the analytical strategy were done following the initial analysis. Further details on the specific methods used are indicated in the Statistical analysis section below.

### Identification of genetic markers as instrumental variables for obesity-related factors (first sample)

Genetic markers for various obesity-related risk factors comprised SNPs that were associated with the risk factor of interest (*P* < 5 × 10^−8^) based on study participants with European ancestry. Correlated SNPs were excluded based on measures of linkage disequilibrium (LD) R^2^ < 0.1. Instruments for BMI and waist-to-hip ratio (WHR) were identified from meta-analyses of GWASs in approximately 700,000 individuals of European ancestry. GWAS data from the Genetic Investigation of ANthropometric Traits (GIANT) consortium (approximately 325,000 participants) [7,8] were combined with GWAS data from UK Biobank (approximately 375,000 individuals). SNPs associated with body fat percentage (%) were also identified using UK Biobank GWAS data. UK Biobank released genetic data for 488,363 individuals, from which we used data on participants of European descent with valid BMI, WHR, and body fat percentage measurements (374,237, 374,722, and 368,690 individuals, respectively). Genetic instruments for systolic blood pressure (SBP) and diastolic blood pressure (DBP), as well as pulse pressure (PP), were obtained from a GWAS in 375,091 European UK Biobank samples. These GWAS analyses were performed using Plink software [9], adjusted for age, sex, genotyping array, and principal components for population stratification as well as manual or automated blood pressure measurements. Instruments for circulating high-density lipoprotein cholesterol (HDL) and low-density lipoprotein cholesterol (LDL), total cholesterol, and triglycerides were identified from the Global Lipids Genetics Consortium (GLGC) study [10]. Instruments for circulating factors related to hyperglycemia and hyperinsulinemia, including fasting glucose and fasting insulin, were identified from the Meta-Analysis of Glucose and Insulin-Related Traits Consortium (MAGIC) [11,12]. Finally, instruments for type 2 diabetes were identified from a genetic fine-mapping study by Gaulton and colleagues [13]. Because SNPs associated with risk of type 2 diabetes may act by increasing insulin resistance or affecting pancreatic beta-cell function, we also evaluated subgroups of type 2 diabetes SNPs by stratifying for these two pathophysiologic mechanisms [14].

SNPs with ambiguous strand codification (A/T or C/G) were replaced by SNPs in genetic linkage (R^2^ > 0.8) using the *proxysnps* R package (European populations) (R Project) or were removed from the analyses if the minor allele frequency was higher than 0.4. For each SNP included in the different instrumental variables, the genetic effect estimate on exposure expressed in SDs of the trait per allele (β_GE_) was retrieved from each respective GWAS, along with the corresponding standard errors (SE_GE_). Table 1 provides details on the number of SNPs that constituted the instrumental variable for each potential risk factor, the proportion of variance explained by the instrument (or cumulative SNP liability in the case of type 2 diabetes), and the mean and SD of the respective risk factors in the original discovery study. Effect estimates for each individual SNP regarding their association with risk factor (β_GE_) are presented in S1 Table.

**Table 1:** Description of SNPs used as instrumental variables for obesity-related factors.

Risk factor (source)	Mean (SD)^a^	Units	*n* SNP^b^	Variance (%)^c^
BMI [7,8] (UK Biobank)	27.2 (4.7)	kg/m^2^	709	9.5
WHR [7,8] (UK Biobank)	1.0 (0.1)	cm/cm	355	2.9
Body fat percentage (UK Biobank)	31.8 (6.6)	(%)	398	3.5
SBP (UK Biobank)	140.3 (19.6)	mmHg	199	3.1
DBP (UK Biobank)	82.2 (10.9)	mmHg	233	3.7
PP (UK Biobank)	59.1 (14.5)	mmHg	260	4.6
HDL [10]	53.3 (15.5)	mg/dL	69	13.7
LDL [10]	133.6 (38.0)	mg/dL	52	14.6
Total cholesterol [10]	213.28 (42.6)	mg/dL	70	15.0
Triglycerides [10]	140.85 (87.8)	mg/dL	41	11.7
Fasting glucose [11,12]	5.2 (0.8)	mmol/L	37	4.8
Fasting insulin [11,12]	56.9 (44.4)	pmol/L	17	1.2
Type 2 diabetes [13]	-	-	39	5.7

^a^Mean and SD for each risk factor in the original discovery study for the SNPs used as instrumental variables.

^b^Number of SNPs used in the instrument variable when evaluating each risk factor of interest with risk.

^c^Variance explained by the instrumental variable for each risk factor as indicated in the discovery GWAS (see marker publication).

Abbreviations: BMI, body mass index; DBP, diastolic blood pressure; GWAS, genome-wide association study; HDL, high-density lipoprotein cholesterol; LDL, low-density lipoprotein cholesterol; PP, pulse pressure; SBP, systolic blood pressure; SD, standard deviation; SNP, single nucleotide polymorphism; WHR, waist-to-hip ratio.

### RCC association results (second sample)

To evaluate the association of each SNP used in the respective instrumental variables with RCC risk, we used summary genetic effect estimates on RCC risk (β_GD_) with the corresponding standard errors (SE_GD_) from the most recent GWAS on RCC [15]. This study encompassed data from 31,190 study participants, including 5,586 RCC patients and 13,075 control participants from previous GWASs [16–20], as well as an additional 5,198 RCC patients and 7,331 control participants with new GWA data, resulting in a total of 10,784 RCC patients and 20,406 control participants. These samples comprised prospective and retrospective case-control studies coordinated by four institutes, including the International Agency for Research on Cancer (IARC), the United States National Cancer Institute (NCI), MD Anderson Cancer Center (MDA), and United Kingdom cancer research centers (UK) (S2 Table). Sex-stratified analyses were performed for study participants from the IARC study sample (3,227 male RCC patients and 4,915 male control participants; 1,992 female RCC patients and 3,095 female control participants). SNPs with an imputation quality score (R^2^ or info score) lower than 0.7 were not used in the MR analysis. Effect estimates for association with disease risk for each SNP (β_GD_) can be observed in S1 Table.

### Statistical analysis

A priori power calculations for the MR analysis to detect an association of nominal statistical significance (*P* < 0.05) were performed for instrumental variables explaining a range in variance of a causal risk factor, using the method proposed by Burgess [21]. Fig A in S1 Text depicts the statistical power of instrumental variables for different levels of explained variance for the risk factor of interest.

OR_SD_ were calculated as effect estimates on RCC risk for an SD increment for each risk factor of interest using the corresponding instrumental variable. The primary MR analysis and the instrumental SNP heterogeneity analysis were conducted using the likelihood-based approach described by Burgess and colleagues [6]. Heterogeneity of initial effect estimates between the four data sources were investigated by estimating the percentage of variance that is attributable to study heterogeneity (I^2^ statistic). The *P* value for heterogeneity (*P*_Study-Heterogeneity_) assumed a fixed-effect model with 3 degrees of freedom. For the sex-stratified risk analysis, the *P* value for heterogeneity (*P*_Sex-Heterogeneity_) was obtained from a fixed-effect model with 1 degree of freedom.

As a sensitivity test, the presence of pleiotropy (i.e., genetic contribution to disease risk through a separate pathway) and potential outlier SNPs among genetic instruments were assessed using a novel approach labeled “MR pleiotropy residual sum and outlier” (MR-PRESSO) [22]. SNPs behaving as outliers were excluded from the instrument, and the effect estimate for the relevant risk factor of interest was reassessed. We also provided OR estimates using the complementary weighted-median method, wherein the effect estimate is weighted toward the median of the distribution of SNPs used in the instrumental variable [23]. This approach is less sensitive to individual SNPs strongly influencing the overall effect estimate. Furthermore, to evaluate the extent to which directional pleiotropy (nonbalanced horizontal pleiotropy in the MR risk estimates) may affect the OR_SD_ estimates, we used an Egger regression approach (SIMEX version that does not assume the absence of measurement error on the β_GE_ estimate) [24]. As a visual evaluation of pleiotropy, we also provided funnel plots depicting the weight exerted on the effect estimate along the y-axis (β_GE_/SE_GD_) and estimates of the effect on RCC along the x-axis (exp[β_GD_/β_GE_]) for each SNP used in the corresponding instrumental variable. Finally, we removed one SNP at a time from the instrument and reestimated the risk estimate to evaluate whether individual SNPs dominated the overall effect estimate. Statistical analyses were performed using R (R Project) and Plink [9].

## Results

### MR results of RCC for obesity-related risk factors

#### BMI, WHR, and body fat percentage

**Fig 1:**
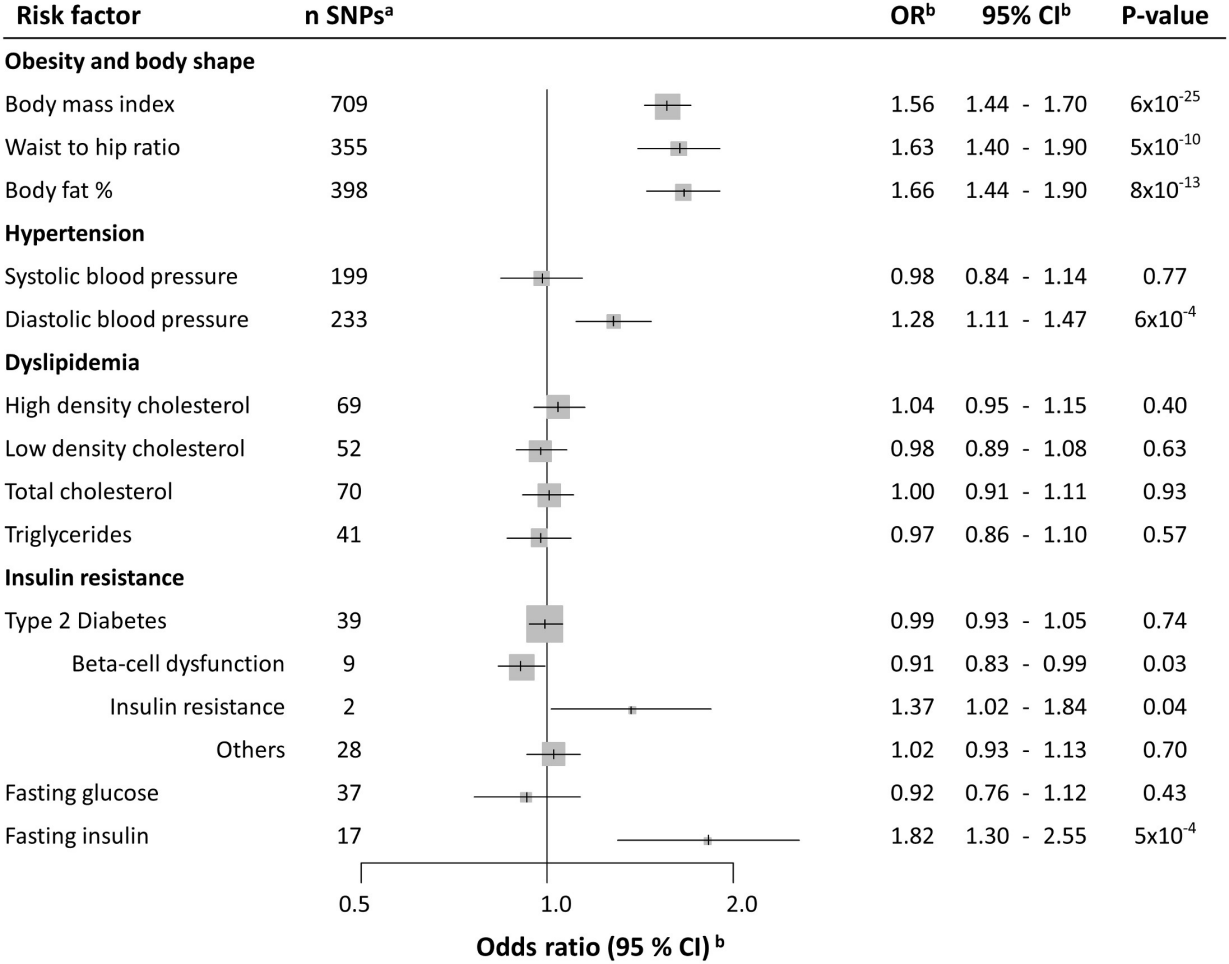
Forest plot depicts OR estimates of RCC for the instrumental variables defined by genetic markers of obesity-related risk factors. ^a^Number of SNPs used in each instrumental variable. ^b^OR of RCC associated with one SD increment for each risk factor, as estimated using the instrumental variable. These ORs were estimated using the likelihood method. CI, confidence interval; OR, odds ratio; RCC, renal cell carcinoma; SD, standard deviation; SNP, single nucleotide polymorphism.

**Fig 2:**
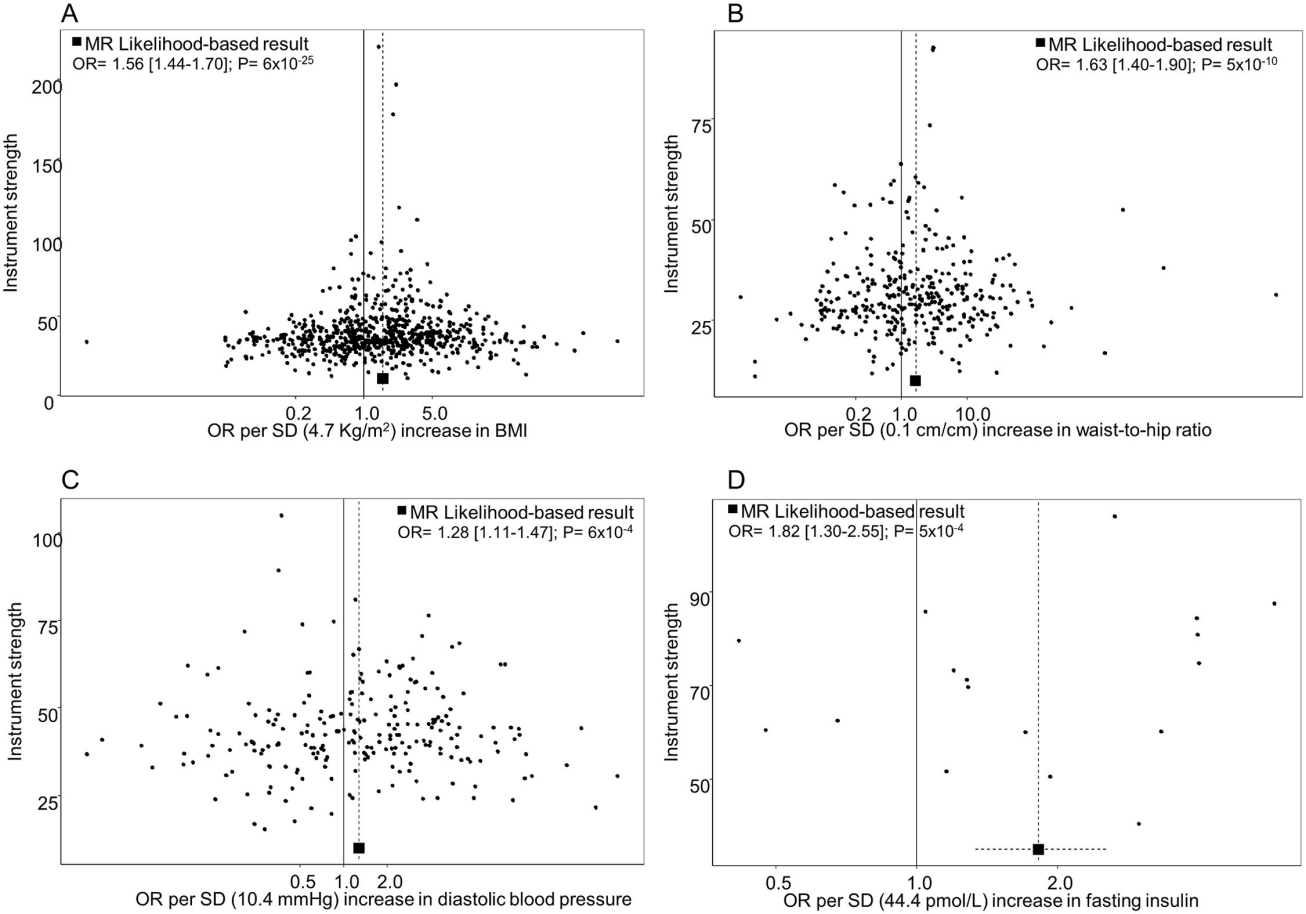
Funnel plots depict the weight exerted for each SNP used in the genetic instruments, along the y-axis (β_GP_/SE_GD_), into the estimated effect on RCC odds, along the x-axis (exp[β_GD_/β_GP_]), for (A) BMI, (B) waist-to-hip ratio, (C) diastolic blood pressure, and (D) fasting insulin. Funnel plot x-axis is in logarithmic scale. BMI, body mass index; MR, mendelian randomization; OR, odds ratio; RCC, renal cell carcinoma; SD, standard deviation; SNP, single nucleotide polymorphism.

We estimated that each SD increment in BMI (1 SD: 4.7 kg/m^*2*^) increased the risk of RCC by 56% (OR_*SD*_: 1.56, 95% confidence interval [CI] 1.44–1.70) (Fig 1; Fig B in S1 Text for study stratification). A similar increase in risk was also seen for WHR (1 SD: 0.1 cm/cm) (OR_*SD*_: 1.63, 95% CI 1.40–1.90) (Fig 1; Fig C in S1 Text for study stratification) and for body fat percentage (1 SD: 6.6%) (OR_*SD*_: 1.66, 95% CI 1.44–1.90) (Fig 1; Fig D in S1 Text for study stratification). SNP heterogeneity (*P*_*SNP_heterogeneity*_ = 9 × 10^*−8*^) and horizontal pleiotropy (*P*_*MR_PRESSO*_ < 1 × 10^*−4*^) were observed for each obesity instrument (S3 Table), but there was little evidence of outlier SNPs or directional pleiotropy (*P*_*MR-Egger intercept*_ > 0.12, S3 Table). The funnel plot for the BMI instruments (Fig 2A) indicated a symmetric distribution of effect estimates, and the leave-one-out histogram (Fig E in S1 Text) did not indicate that any individual SNPs were driving the overall association with risk, with similar results for WHR (Fig 2B and Fig F in S1 Text) and body fat percentage (Fig G in S1 Text). Accordingly, the complementary weighted-median MR method provided similar results (OR_*SD*_: 1.75, 95% CI 1.50–2.03, for BMI; OR_*SD*_: 1.45, 95% CI 1.14–1.86, for WHR; OR_*SD*_: 1.63, 95% CI 1.31–2.03, for body fat %; S3 Table). Sex heterogeneity was not observed in the IARC study sample (*P* > 0.41; Fig B–D in S1 Text).

#### Blood pressure

The MR analysis indicated that DBP (1 SD of DBP: 10.9 mmHg) (OR_*SD*_: 1.28, 95% CI 1.11–1.47) (Fig 1; Fig H in S1 Text for study stratification), but not SBP (OR_*SD*_: 0.98, 95% CI 0.84–1.14) (Fig 1; Fig I in S1 Text for study stratification), influences RCC risk (*P* for difference in OR: 0.01). In accordance with the risk association of DBP, we also observed an inverse association for PP with RCC risk (OR_*SD*_: 0.77, 95% CI 0.68–0.88) (Fig J in S1 Text for study stratification). Sensitivity analyses did not detect any nonbalanced pleiotropic effect biasing the DBP risk estimates (S3 Table), and the funnel plot demonstrated a symmetric distribution of effect estimates from DBP SNPs (Fig 2C). The leave-one-out histogram for DBP can be seen in Fig K in S1 Text. Funnel plots and leave-one-out histograms for SBP and PP can be observed in Fig L and Fig M in S1 Text, respectively. In the IARC study samples, sex heterogeneity was not observed for DBP and SBP. However, the inverse association for PP with RCC risk was observed in men (OR_*SD*_: 0.74, 95% CI 0.58–0.94) but not in women (OR_*SD*_: 1.25, 95% CI 0.93–1.69) (*P*_*Sex-heterogeneity*_ = 7 × 10^*−3*^) (Fig J in S1 Text).

#### Blood lipids

For blood lipid levels—including HDL, LDL, total cholesterol, and triglycerides—the MR analysis indicated little evidence for an influence on RCC risk, with the overall OR_*SD*_ estimates ranging from 0.97 to 1.04 (P≥ 0.40, Fig 1, Fig N-Q in S1 Text for stratified analyses, Fig R–U in S1 Text for sensitivity analyses).

#### Type 2 diabetes, insulin, and glucose

Overall type 2 diabetes was not associated with RCC risk (OR_*SD*_: 0.99, 95% CI 0.93–1.05), but when analyzing subgroups related to type 2 diabetes in a secondary analysis, SNPs related to beta-cell dysfunction were nominally inversely associated with risk (OR_*SD*_: 0.91, 95% CI 0.83–0.99), and SNPs related to insulin resistance were nominally positively associated with risk (OR_*SD*_: 1.37, 95% CI 1.02–1.84) (Fig 1). Notably, fasting insulin was positively associated with RCC risk, with each SD increment (44.4 pmol/L) increasing RCC risk by 82% (OR_*SD*_: 1.82, 95% CI 1.30–2.55) whereas little evidence was seen for a role of fasting glucose in RCC (*P* = 0.43) (Fig 1). No clear evidence of heterogeneity by study or sex was observed for any of the diabetes-related risk factors (Fig V–X in S1 Text), nor did the Egger regression or other sensitivity analyses indicate the presence of pleiotropy (S3 Table and Fig Y–AA in S1 Text).

## Discussion

This MR analysis confirmed a role for higher BMI and DBP in RCC etiology and provided novel evidence for a role of fasting insulin. In contrast, we found little evidence for a role of other obesity-related risk factors RCC etiology, with evaluated risk factors including diagnosis of type 2 diabetes, SBP, and blood lipid levels.

There is an abundance of observational studies implicating obesity in RCC development, and several reviews have concluded that there is convincing evidence that being overweight or obese increases RCC risk [25,26]. Although a previous MR analysis on obesity and RCC did not provide unequivocal support for such a relation, the statistical power of that study was limited by a small sample size [27]. The current study leveraged results of several large-scale GWAS initiatives on both the metabolic risk factors of interest and RCC risk. We identified 709 SNPs as instrumental variables for BMI through meta-analyses of the existing GIANT GWAS and the novel UK Biobank GWAS, as well as results from our recent RCC GWAS initiative of over 10,000 RCC patients and 20,000 control participants. This enabled a well-powered two-sample MR analysis that provided strong support for a role of obesity and being overweight in RCC etiology (Fig 1 and Fig A in S1 Text). In particular, we estimated that an SD increment in BMI—approximately equivalent to 5 kg/m^2^—increases the risk of RCC by 56% (Fig 1). Considering that most observational studies have estimated risk increases of approximately 30% per 5 units of BMI [25,26], these results are notable, as they suggest that the impact of higher BMI and obesity in RCC etiology may be even more important than previously thought. This difference in effect estimates may reflect an inherent weakness in observational studies that use single direct measures of the risk factor of interest, whereas the genetic variants used to proxy the risk factor in an MR study would be expected to reflect the life-course exposure, thus better capturing the cumulative exposure.

Several additional obesity-related factors have been consistently implicated in RCC—in particular, factors traditionally associated with the metabolic syndrome, which is defined as a cluster of factors that increase the risk of type 2 diabetes and cardiovascular disease [28]. In addition to obesity, these risk factors typically include impaired glucose tolerance or diabetes, hypertension, and dyslipidemia [29,30]. The Metabolic Syndrome and Cancer (Me-Can) consortium pooled individual-level prospective data on over half a million study participants and estimated that men with high BMI, blood pressure, and blood triglycerides have up to a 3-fold risk increase of RCC compared to men without these conditions, and some have suggested that these risk factors exert independent effects on RCC risk [2,29]. When we used genetic markers for these risk factors, we observed a positive association between DBP and RCC, whereas no association with risk was seen for SBP nor for blood lipids, including HDL, LDL, total cholesterol, and triglycerides. Based on the fraction of variance explained by the instrumental variables of these factors (Table 1), we estimated that there was sufficient statistical power (>80%) to detect even modest log-linear relative risks of 1.2 per SD increment in a risk factor and close to complete statistical power (100%) to detect relative risks of 1.5 (Fig A in S1 Text). As such, this MR-based analysis did not support a role of SBP and blood lipids in RCC [2]. However, the positive association between the instrumental variable of DBP with risk is in line with previous epidemiological evidence. We also note that the difference in risk effect estimates between the DBP and SBP was significant, suggesting that it was not due to chance and lack of statistical power [2,31]. The mechanisms by which elevated blood pressure might influence RCC development are not established, but several plausible mechanisms have been suggested, including by influencing angiogenesis, growth factors, and renal function, thereby making the kidney more susceptible to carcinogens [31]. We are not aware of any hypothesis for why DBP rather than SBP would be important in RCC, but note that some epidemiological studies have indicated a stronger association with risk for DBP than for SBP [31].

We further observed a strong association between the instrumental variables of fasting insulin and RCC risk (Fig 1). In particular, we estimated that one SD increment in fasting insulin results in 82% increased risk of RCC. Whereas no association with RCC risk was seen for type 2 diabetes, we found an inverse association with risk for SNPs related to beta-cell dysfunction and a positive association with risk for SNPs related to insulin resistance, even though these subanalyses were conducted with limited statistical power. Insulin resistance may lead to compensatory hyperinsulinemia when pancreatic beta cells increase insulin secretion to maintain normal blood glucose. The contrasting associations with risk for the beta-cell dysfunction and insulin-resistance SNPs would therefore lend further support for a role of insulin in RCC etiology, as well as explain the lack of association with RCC risk for overall type 2 diabetes. Based on previously published data on the relationship between fasting insulin and BMI [32], we estimated that approximately one-fifth of the effect of BMI on RCC risk would be mediated by fasting insulin. Whereas the experimental data on the role of insulin in RCC tumorigenesis are still limited [33], there is ample in vivo and in vitro data describing pro-proliferative and antiapoptotic properties of insulin together with insulin-like growth factor 1 (IGF1) [33–35]. Prospective studies evaluating the association between directly measured fasting insulin (prediagnostically) and RCC risk are further warranted, and improving our understanding of insulin and IGF1 signaling in RCC development and progression may also offer therapeutic opportunities [36].

An overarching observation was that little evidence was found for sex heterogeneity in the relation between any of the risk-associated factors and RCC. This result is interesting, as it contrasts with some traditional observational studies that have reported stronger associations for obesity-related factors with RCC risk among women [37,38].

In conclusion, this study confirmed the important role of being overweight and having elevated DBP in affecting RCC risk and provided novel evidence for an etiological role of elevated insulin. The study gave little support for SBP or blood lipids and glucose being important in RCC. Taken together, these results advance our understanding of RCC etiology but highlight the need for further research focused on understanding how DBP and insulin-related pathways affect RCC risk, as well as complementary research aiming to identify additional pathways explaining the mechanisms by which obesity influences RCC development.

## Supporting information

S1 Table Association parameters of instrumental SNPs of obesity-related risk factors for RCC. ^a^Beta-cell dysfunction SNPs within type 2 diabetes. ^b^Insulin resistance SNPs within type 2 diabetes. Β, beta estimate; BMI, body mass index; BP, base position; CHR, chromosome; DBP, diastolic blood pressure; EffAl, effect allele; GD, genotype-to-disease; GE, genotype-to-exposure; HDL, high-density lipoprotein cholesterol; LDL, low-density lipoprotein cholesterol; OthAl, other allele; PP, pulse pressure; SBP, systolic blood pressure; SE, standard error.
(PDF)

S2 Table Description of the studies participating in the RCC GWAS meta-analysis. GWAS, genome-wide association study; RCC, renal cell carcinoma.
(PDF)

S3 Table Pleiotropy assessment and risk increase of obesity-related factors on RCC provided by sensitivity tests. LCI, lower confidence interval; *n* SNPs, number of SNPs; OR, odds ratio; P, *P* value; PSNP-Heterogeneity, heterogeneity *P* value between instrumental SNP causal estimates (β_GD_/β_GE_) from genetic effects in S1 Table; UCI, upper confidence interval.
(PDF)

S1 Text Supplementary figures. (PDF)

## Abbreviations

BMI: body mass index
CI: confidence interval
DBP: diastolic blood pressure
GIANT: Genetic Investigation of ANthropometric Traits
GLGC: Global Lipids Genetics Consortium
GWAS: genome-wide association study
HDL: high-density lipoprotein cholesterol
IARC: International Agency for Research on Cancer
IGF1: insulin-like growth factor 1
LD: linkage disequilibrium
LDL: low-density lipoprotein cholesterol
MAGIC: Meta-Analysis of Glucose and Insulin-Related Traits Consortium
MDA: MD Anderson Cancer Center
Me-Can: Metabolic Syndrome and Cancer
MR: mendelian randomization
MR-PRESSO: MR pleiotropy residual sum and outlier
NCI: National Cancer Institute
OR: odds ratio
PP: pulse pressure
RCC: renal cell carcinoma
SBP: systolic blood pressure
SD: standard deviation
SNP: single nucleotide polymorphism
WHR: waist-to-hip ratio
