# Supplementary material for: The influence of obesity-related factors in the etiology of renal cell carcinoma—A mendelian randomization study
Source: PLoS Med. 2019 Jan 3;16(1):e1002724. doi: 10.1371/journal.pmed.1002724 (PMC6317776; doi:10.1371/journal.pmed.1002724)
Supplement: S1 Table — aBeta-cell dysfunction SNPs within type 2 diabetes. bInsulin resistance SNPs within type 2 diabetes. Β, beta estimate; BMI, body mass index; BP, base position; CHR, chromosome; DBP, diastolic blood pressure; EffAl, effect allele; GD, genotype-to-disease; GE, genotype-to-exposure; HDL, high-density lipoprotein cholesterol; LDL, low-density lipoprotein cholesterol; OthAl, other allele; PP, pulse pressure; SBP, systolic blood pressure; SE, standard error. (PDF) [file pmed.1002724.s001.pdf]

S1 Table- Association parameters of instrumental SNPs of obesity-related risk factors for RCC.

SNP	CHR	BP	Quality	EffAl	OthAl	β_{GE} (SE_{GE})	β_{GD} (SE_{GD})	IARC β_{GD} (SE_{GD})	NCI β_{GD} (SE_{GD})	MDA β_{GD} (SE_{GD})	UK β_{GD} (SE_{GD})	Phenotype
rs12044597	1	1708801	0,94	G	A	0.015 (0.002)	-0.017 (0.019)	-0.028 (0.029)	-0.01 (0.03)	-0.092 (0.079)	0.025 (0.051)	BMI
rs4648360	1	2723345	0,96	C	T	0.014 (0.002)	0.03 (0.019)	0.011 (0.028)	0.043 (0.031)	0.054 (0.078)	0.052 (0.052)	BMI
rs11583755	1	6672729	1,00	C	A	0.014 (0.002)	0.015 (0.019)	-0.003 (0.028)	0.044 (0.031)	0.036 (0.079)	-0.016 (0.053)	BMI
rs3011926	1	7737799	0,97	A	G	0.013 (0.002)	-0.016 (0.018)	-0.024 (0.027)	-0.007 (0.03)	0.115 (0.08)	-0.071 (0.052)	BMI
rs1381928	1	8690933	0,99	A	G	0.012 (0.002)	-0.034 (0.019)	-0.009 (0.028)	-0.072 (0.031)	0.092 (0.078)	-0.075 (0.053)	BMI
rs2791653	1	11129848	0,99	A	G	0.014 (0.002)	0.066 (0.021)	0.054 (0.031)	0.096 (0.035)	-0.072 (0.09)	0.078 (0.061)	BMI
rs10917502	1	19961679	0,97	A	G	0.016 (0.003)	-0.007 (0.022)	-0.021 (0.033)	-0.02 (0.035)	0.118 (0.089)	0.025 (0.061)	BMI
rs681648	1	23342362	1,00	A	G	0.018 (0.003)	0.03 (0.024)	0.002 (0.036)	0.002 (0.04)	0.214 (0.103)	0.13 (0.068)	BMI
rs2228552	1	32165495	1,00	T	G	0.017 (0.002)	-0.001 (0.019)	-0.025 (0.028)	0.035 (0.031)	0.009 (0.08)	-0.022 (0.054)	BMI
rs4295917	1	32173360	0,99	A	G	0.017 (0.002)	-0.005 (0.019)	-0.026 (0.028)	0.031 (0.031)	-0.016 (0.08)	-0.032 (0.055)	BMI
rs10798918	1	33275981	1,00	T	C	0.016 (0.003)	-0.031 (0.023)	-0.062 (0.033)	0.03 (0.038)	0.032 (0.101)	-0.112 (0.068)	BMI
rs4653017	1	33776728	0,99	T	C	0.013 (0.002)	0.001 (0.019)	-0.015 (0.029)	0.009 (0.032)	0.041 (0.082)	0.02 (0.055)	BMI
rs11577094	1	38026600	1,00	T	C	0.02 (0.003)	0.014 (0.034)	0.002 (0.052)	0.061 (0.056)	0.206 (0.142)	-0.157 (0.092)	BMI
rs112566467	1	39562627	0,98	T	C	0.022 (0.003)	0.041 (0.022)	0.033 (0.032)	0.052 (0.036)	0.067 (0.096)	0.029 (0.062)	BMI
rs61743745	1	40036944	0,92	G	A	0.042 (0.007)	-0.032 (0.058)	-0.051 (0.092)	0.034 (0.092)	-0.275 (0.25)	-0.07 (0.145)	BMI
rs61232586	1	40086357	0,99	T	G	0.017 (0.003)	-0.027 (0.022)	-0.03 (0.032)	-0.003 (0.036)	-0.17 (0.094)	-0.024 (0.062)	BMI
rs1707322	1	46505147	1,00	G	A	0.013 (0.002)	0.036 (0.02)	0.06 (0.029)	0.029 (0.033)	-0.05 (0.088)	0.006 (0.057)	BMI
rs2984618	1	47690438	1,00	T	G	0.016 (0.002)	-0.008 (0.018)	-0.003 (0.027)	-0.032 (0.03)	0.076 (0.078)	0.01 (0.052)	BMI
rs657452	1	49589847	1,00	A	G	0.019 (0.002)	-0.006 (0.018)	-0.023 (0.027)	0.025 (0.03)	-0.033 (0.081)	-0.022 (0.052)	BMI
rs4926542	1	50263773	1,00	C	T	0.019 (0.002)	-0.02 (0.019)	-0.029 (0.028)	0 (0.032)	-0.071 (0.084)	-0.023 (0.055)	BMI
rs3766430	1	54730651	0,98	C	T	0.011 (0.002)	-0.016 (0.018)	-0.022 (0.028)	-0.029 (0.03)	0.027 (0.079)	0.026 (0.051)	BMI
rs2481665	1	62594677	0,99	T	C	0.016 (0.002)	-0.002 (0.018)	-0.003 (0.027)	-0.007 (0.03)	-0.011 (0.079)	0.019 (0.052)	BMI
rs7519259	1	66434743	1,00	A	G	0.013 (0.002)	0.011 (0.018)	0.031 (0.027)	-0.002 (0.03)	-0.12 (0.078)	0.033 (0.051)	BMI
rs3101336	1	72751185	1,00	C	T	0.027 (0.002)	0 (0.019)	-0.031 (0.028)	0.069 (0.031)	-0.038 (0.078)	-0.078 (0.052)	BMI
rs990871	1	72823713	1,00	T	C	0.026 (0.002)	0.003 (0.019)	-0.023 (0.028)	0.066 (0.031)	-0.057 (0.078)	-0.067 (0.052)	BMI
rs12566985	1	75002193	0,94	G	A	0.019 (0.002)	0.013 (0.019)	-0.011 (0.028)	0.022 (0.03)	0.066 (0.079)	0.043 (0.051)	BMI
rs12049202	1	77967523	0,99	T	C	0.023 (0.002)	0.036 (0.023)	0.027 (0.035)	0.034 (0.038)	0.113 (0.096)	0.033 (0.063)	BMI
rs17391694	1	78623626	0,83	T	C	0.032 (0.003)	0.057 (0.031)	0.085 (0.051)	0.039 (0.05)	0.16 (0.112)	-0.023 (0.083)	BMI
rs12142416	1	78830045	0,82	T	C	0.028 (0.004)	0.04 (0.038)	0.178 (0.064)	-0.048 (0.058)	0.045 (0.144)	-0.031 (0.091)	BMI
rs12088284	1	80798635	1,00	T	C	0.016 (0.002)	-0.004 (0.02)	0 (0.03)	0.003 (0.033)	0.014 (0.086)	-0.043 (0.056)	BMI
rs284227	1	82379446	1,00	C	T	0.015 (0.002)	0.007 (0.021)	-0.018 (0.031)	0.02 (0.034)	0.064 (0.091)	0.035 (0.06)	BMI
rs6690764	1	92976590	1,00	G	A	0.015 (0.003)	0.009 (0.022)	0.027 (0.033)	-0.016 (0.037)	-0.055 (0.098)	0.042 (0.063)	BMI
rs6686243	1	96280265	1,00	A	C	0.016 (0.002)	0.001 (0.019)	0.001 (0.028)	0.021 (0.032)	-0.048 (0.082)	-0.039 (0.054)	BMI
rs11165643	1	96924097	1,00	T	C	0.019 (0.002)	0.03 (0.018)	0.034 (0.027)	0.028 (0.03)	-0.001 (0.079)	0.037 (0.052)	BMI
rs1925834	1	97091507	0,92	A	T	0.02 (0.002)	0.001 (0.02)	0.019 (0.031)	-0.03 (0.033)	-0.191 (0.088)	0.107 (0.057)	BMI
rs995258	1	97431052	0,98	A	C	0.015 (0.002)	-0.021 (0.018)	-0.023 (0.028)	-0.038 (0.03)	-0.164 (0.078)	0.096 (0.051)	BMI
rs12062845	1	98342685	1,00	A	C	0.015 (0.002)	-0.034 (0.021)	-0.03 (0.031)	-0.032 (0.036)	0.027 (0.096)	-0.077 (0.062)	BMI
rs17496332	1	107546375	1,00	G	A	0.012 (0.002)	0.003 (0.019)	0.006 (0.028)	0.018 (0.032)	-0.045 (0.083)	-0.032 (0.055)	BMI
rs11802147	1	108001307	0,99	A	G	0.014 (0.002)	-0.017 (0.02)	0.014 (0.029)	-0.05 (0.033)	-0.137 (0.086)	0.012 (0.056)	BMI
rs17024393	1	110154688	0,99	C	T	0.068 (0.006)	0.061 (0.048)	0.033 (0.065)	0.048 (0.084)	0.624 (0.266)	0.07 (0.16)	BMI
rs1546924	1	112273485	0,99	T	C	0.014 (0.002)	0.034 (0.018)	0.021 (0.027)	0.025 (0.03)	0.156 (0.078)	0.053 (0.052)	BMI
rs17544384	1	115295160	1,00	C	T	0.016 (0.003)	-0.016 (0.022)	-0.031 (0.033)	-0.008 (0.037)	-0.044 (0.096)	0.033 (0.062)	BMI
rs7534091	1	118864616	1,00	G	A	0.012 (0.002)	-0.025 (0.021)	-0.067 (0.031)	-0.002 (0.034)	-0.014 (0.088)	0.053 (0.058)	BMI
rs10923724	1	119546842	1,00	C	T	0.011 (0.002)	0.008 (0.018)	0.036 (0.027)	-0.016 (0.03)	-0.11 (0.077)	0.028 (0.052)	BMI
rs4357530	1	151103153	0,96	G	A	0.015 (0.002)	0.02 (0.02)	-0.029 (0.029)	0.045 (0.032)	-0.009 (0.084)	0.132 (0.056)	BMI

rs905938	1	154991389	0,99	C	T	0.016 (0.002)	-0.041 (0.021)	-0.016 (0.031)	-0.066 (0.034)	0.018 (0.087)	-0.084 (0.058)	BMI
rs61813324	1	156049877	0,73	T	C	0.03 (0.003)	-0.015 (0.031)	-0.023 (0.045)	0.025 (0.051)	-0.1 (0.124)	-0.06 (0.09)	BMI
rs11264489	1	156480831	0,99	G	A	0.014 (0.002)	0.021 (0.019)	0.006 (0.028)	0.026 (0.031)	0.034 (0.081)	0.056 (0.053)	BMI
rs10733051	1	167280354	1,00	A	G	0.011 (0.002)	-0.019 (0.018)	-0.015 (0.027)	-0.009 (0.03)	-0.121 (0.076)	-0.019 (0.051)	BMI
rs34720381	1	171455322	1,00	T	C	0.023 (0.004)	-0.074 (0.033)	-0.028 (0.05)	-0.091 (0.053)	0.151 (0.152)	-0.241 (0.087)	BMI
rs61826771	1	173936487	1,00	C	T	0.022 (0.004)	-0.042 (0.028)	-0.064 (0.043)	-0.027 (0.046)	-0.131 (0.123)	0.028 (0.081)	BMI
rs12564992	1	174478100	0,99	G	A	0.02 (0.003)	-0.026 (0.029)	-0.038 (0.045)	-0.015 (0.047)	-0.137 (0.124)	0.025 (0.081)	BMI
rs543874	1	177889480	1,00	G	A	0.048 (0.002)	0.007 (0.023)	0.058 (0.036)	-0.027 (0.037)	0.058 (0.097)	-0.07 (0.062)	BMI
rs684227	1	177901741	1,00	G	A	0.022 (0.002)	0.028 (0.018)	0.021 (0.027)	0.037 (0.03)	0.176 (0.078)	-0.037 (0.052)	BMI
rs10920678	1	190239907	1,00	A	G	0.017 (0.002)	0.017 (0.018)	0.007 (0.027)	0.002 (0.03)	0.191 (0.077)	0.017 (0.051)	BMI
rs7527427	1	195006827	0,99	T	C	0.016 (0.002)	0.041 (0.022)	0.069 (0.032)	0.032 (0.036)	-0.11 (0.092)	0.03 (0.063)	BMI
rs2172935	1	201826340	1,00	T	C	0.024 (0.002)	-0.005 (0.019)	-0.005 (0.028)	-0.027 (0.032)	0.052 (0.082)	0.032 (0.054)	BMI
rs9077	1	202116238	0,95	G	A	0.014 (0.002)	0.01 (0.02)	0.043 (0.03)	-0.009 (0.031)	0 (0.084)	-0.035 (0.055)	BMI
rs11119208	1	209211968	1,00	A	G	0.012 (0.002)	0.017 (0.018)	-0.002 (0.028)	0.023 (0.03)	-0.11 (0.078)	0.121 (0.052)	BMI
rs56265166	1	209517989	0,99	G	A	0.017 (0.003)	0.033 (0.022)	0.069 (0.032)	-0.004 (0.036)	0.096 (0.094)	-0.023 (0.064)	BMI
rs227179	1	210216731	1,00	A	G	0.013 (0.002)	0.015 (0.018)	-0.005 (0.027)	0.044 (0.03)	0.023 (0.079)	-0.005 (0.052)	BMI
rs17015701	1	210337691	1,00	A	G	0.016 (0.002)	-0.018 (0.023)	0.002 (0.035)	-0.052 (0.038)	0.011 (0.093)	-0.004 (0.066)	BMI
rs11118308	1	219633869	0,97	A	G	0.011 (0.002)	0.006 (0.018)	0.043 (0.027)	-0.024 (0.03)	-0.082 (0.078)	0 (0.052)	BMI
rs2491864	1	242986063	0,99	A	G	0.014 (0.002)	-0.005 (0.022)	-0.029 (0.032)	0.016 (0.036)	-0.083 (0.093)	0.06 (0.062)	BMI
rs3753549	1	243722892	0,99	C	T	0.02 (0.003)	-0.001 (0.026)	-0.006 (0.037)	0.016 (0.043)	-0.111 (0.106)	0.027 (0.075)	BMI
rs4658403	1	243832560	0,99	C	T	0.019 (0.003)	0.007 (0.024)	0.01 (0.035)	0.009 (0.04)	-0.117 (0.101)	0.047 (0.069)	BMI
rs4854306	2	433274	0,95	G	A	0.019 (0.002)	0.022 (0.022)	0.036 (0.031)	0.004 (0.036)	-0.008 (0.092)	0.031 (0.061)	BMI
rs12996547	2	602036	1,00	T	C	0.025 (0.002)	0.017 (0.019)	-0.011 (0.028)	0.034 (0.031)	0.024 (0.084)	0.068 (0.054)	BMI
rs12714415	2	651430	0,98	T	C	0.059 (0.002)	-0.011 (0.024)	-0.045 (0.036)	-0.013 (0.04)	0.12 (0.104)	0.061 (0.069)	BMI
rs10929925	2	6155557	1,00	C	A	0.014 (0.002)	0.02 (0.018)	0.014 (0.027)	0.015 (0.03)	0.055 (0.081)	0.04 (0.052)	BMI
rs74639823	2	25041007	1,00	G	T	0.032 (0.004)	0.027 (0.034)	-0.036 (0.053)	0.134 (0.056)	0.059 (0.147)	-0.079 (0.093)	BMI
rs10182181	2	25150296	1,00	G	A	0.032 (0.002)	-0.01 (0.018)	-0.014 (0.027)	-0.015 (0.03)	-0.009 (0.077)	0.017 (0.052)	BMI
rs12468863	2	26940294	0,99	C	T	0.016 (0.002)	-0.019 (0.018)	-0.05 (0.027)	0.007 (0.03)	-0.041 (0.078)	0.024 (0.051)	BMI
rs1260326	2	27730940	1,00	C	T	0.011 (0.002)	0.004 (0.018)	-0.01 (0.027)	0.016 (0.03)	0.117 (0.08)	-0.03 (0.053)	BMI
rs4372836	2	28973883	1,00	T	C	0.013 (0.002)	0.002 (0.02)	0.014 (0.029)	-0.007 (0.032)	0.03 (0.084)	-0.028 (0.056)	BMI
rs2888172	2	35439812	1,00	G	A	0.014 (0.002)	0.039 (0.018)	0.027 (0.027)	0.025 (0.03)	0.05 (0.08)	0.121 (0.051)	BMI
rs4670626	2	37046657	0,98	C	T	0.012 (0.002)	0.015 (0.019)	0.041 (0.028)	-0.007 (0.031)	0.049 (0.082)	-0.03 (0.054)	BMI
rs10185199	2	40282202	0,88	G	A	0.016 (0.002)	-0.044 (0.021)	-0.065 (0.031)	-0.003 (0.035)	0.034 (0.084)	-0.125 (0.061)	BMI
rs10169594	2	41637688	0,97	C	T	0.013 (0.002)	-0.033 (0.019)	-0.045 (0.028)	-0.025 (0.032)	0.029 (0.082)	-0.036 (0.055)	BMI
rs1011797	2	44757217	0,99	G	A	0.012 (0.002)	0.02 (0.019)	-0.002 (0.028)	0.042 (0.032)	0.01 (0.081)	0.035 (0.055)	BMI
rs35722922	2	47002226	0,99	A	G	0.016 (0.002)	0.02 (0.019)	0.036 (0.028)	-0.005 (0.031)	-0.114 (0.079)	0.099 (0.053)	BMI
rs35809007	2	47019521	0,98	G	A	0.016 (0.002)	0.02 (0.019)	0.025 (0.029)	0.008 (0.031)	-0.118 (0.08)	0.102 (0.054)	BMI
rs7561278	2	48954905	0,92	T	C	0.015 (0.002)	-0.012 (0.023)	0.012 (0.034)	-0.045 (0.036)	-0.151 (0.096)	0.067 (0.066)	BMI
rs1369295	2	50232266	1,00	A	C	0.019 (0.003)	0.039 (0.025)	0.033 (0.036)	0.053 (0.041)	0.07 (0.112)	0.014 (0.067)	BMI
rs13406839	2	50735433	0,99	A	G	0.015 (0.002)	-0.01 (0.018)	-0.011 (0.027)	-0.027 (0.031)	0.054 (0.082)	0.021 (0.053)	BMI
rs3198123	2	55207693	1,00	C	T	0.014 (0.002)	0.021 (0.02)	0.039 (0.03)	0.012 (0.033)	0.064 (0.083)	-0.033 (0.056)	BMI
rs13432055	2	56603985	0,96	C	T	0.013 (0.002)	0.009 (0.02)	-0.013 (0.03)	0.008 (0.034)	0.13 (0.084)	0.04 (0.06)	BMI
rs1559556	2	57332617	0,99	G	A	0.013 (0.002)	0.055 (0.019)	0.029 (0.028)	0.09 (0.031)	0.083 (0.08)	0.034 (0.053)	BMI
rs1106090	2	58068741	1,00	G	A	0.012 (0.002)	0.041 (0.018)	0.032 (0.028)	0.061 (0.031)	0.069 (0.079)	0.005 (0.053)	BMI
rs4671328	2	58935282	0,98	T	G	0.022 (0.002)	0.011 (0.018)	-0.033 (0.027)	0.05 (0.03)	-0.08 (0.078)	0.086 (0.051)	BMI
rs11125768	2	59306564	0,99	T	C	0.02 (0.002)	0.027 (0.018)	-0.006 (0.027)	0.041 (0.03)	0.137 (0.08)	0.061 (0.052)	BMI
rs12053103	2	59540594	1,00	A	G	0.013 (0.002)	0.009 (0.018)	0.027 (0.027)	-0.001 (0.031)	0.056 (0.079)	-0.05 (0.052)	BMI
rs11688816	2	63053048	0,99	G	A	0.013 (0.002)	-0.002 (0.018)	-0.019 (0.027)	-0.001 (0.03)	0.022 (0.077)	0.045 (0.052)	BMI
rs2861685	2	67837553	1,00	T	C	0.018 (0.002)	0.012 (0.018)	0.011 (0.027)	0.014 (0.03)	-0.063 (0.079)	0.045 (0.053)	BMI

rs12622280	2	79515954	0,80	T	G	0.017 (0.003)	0.004 (0.027)	-0.02 (0.04)	0.002 (0.045)	0.132 (0.119)	0.039 (0.076)	BMI
rs6712609	2	81847998	0,99	C	T	0.013 (0.002)	0.02 (0.019)	0.043 (0.029)	-0.001 (0.032)	0.063 (0.082)	-0.019 (0.054)	BMI
rs7557796	2	86766153	1,00	T	C	0.016 (0.002)	-0.016 (0.019)	-0.048 (0.028)	-0.032 (0.031)	0.138 (0.081)	0.084 (0.054)	BMI
rs4303732	2	100830040	0,99	T	C	0.016 (0.002)	-0.007 (0.018)	0.021 (0.027)	-0.035 (0.03)	0.047 (0.078)	-0.055 (0.054)	BMI
rs6707445	2	104420858	0,99	A	G	0.014 (0.002)	0.032 (0.018)	-0.014 (0.027)	0.076 (0.03)	0.089 (0.08)	0.044 (0.052)	BMI
rs10197031	2	105454590	0,99	C	T	0.016 (0.002)	-0.009 (0.02)	-0.035 (0.03)	0 (0.033)	-0.049 (0.086)	0.077 (0.056)	BMI
rs6730157	2	135907088	0,95	A	G	0.014 (0.002)	0.021 (0.02)	0.043 (0.03)	0.007 (0.033)	0.036 (0.087)	-0.017 (0.056)	BMI
rs4988235	2	136608646	0,92	A	G	0.015 (0.002)	0.005 (0.02)	0.018 (0.031)	-0.01 (0.033)	0.048 (0.088)	-0.01 (0.057)	BMI
rs4954638	2	137435455	1,00	A	C	0.014 (0.002)	-0.015 (0.02)	-0.025 (0.029)	0.022 (0.034)	-0.036 (0.088)	-0.082 (0.06)	BMI
rs17551974	2	142293146	1,00	C	A	0.015 (0.002)	0.022 (0.024)	0.007 (0.035)	0.057 (0.038)	0.028 (0.101)	-0.035 (0.066)	BMI
rs2890652	2	142959931	0,99	C	T	0.019 (0.003)	0.019 (0.024)	0.038 (0.036)	0 (0.04)	-0.067 (0.108)	0.044 (0.071)	BMI
rs6710871	2	143960593	1,00	A	G	0.018 (0.003)	-0.027 (0.026)	-0.029 (0.04)	-0.029 (0.043)	-0.108 (0.111)	0.02 (0.074)	BMI
rs7560871	2	145616899	1,00	A	G	0.024 (0.004)	0.084 (0.032)	0.103 (0.046)	0.016 (0.056)	0.114 (0.151)	0.186 (0.095)	BMI
rs1445305	2	146047105	0,99	G	A	0.019 (0.003)	-0.004 (0.03)	0.033 (0.047)	-0.019 (0.048)	-0.032 (0.133)	-0.063 (0.084)	BMI
rs497418	2	147842671	1,00	A	C	0.017 (0.002)	0.029 (0.018)	0.058 (0.027)	0.053 (0.031)	-0.125 (0.079)	-0.076 (0.052)	BMI
rs11679163	2	159372601	0,99	G	A	0.016 (0.003)	-0.04 (0.025)	-0.075 (0.037)	0.016 (0.042)	-0.065 (0.113)	-0.065 (0.072)	BMI
rs12692596	2	161265910	1,00	T	C	0.012 (0.002)	0.014 (0.019)	0.063 (0.028)	-0.063 (0.031)	0.073 (0.078)	0.034 (0.053)	BMI
rs10192119	2	164581241	1,00	G	T	0.016 (0.002)	0.007 (0.024)	0.007 (0.035)	-0.005 (0.04)	0.119 (0.108)	0 (0.07)	BMI
rs1128249	2	165528624	0,98	T	G	0.013 (0.002)	-0.042 (0.019)	-0.009 (0.028)	-0.061 (0.031)	-0.137 (0.079)	-0.061 (0.053)	BMI
rs12477385	2	166144850	0,94	G	T	0.014 (0.002)	0.038 (0.023)	0.099 (0.033)	-0.033 (0.038)	-0.059 (0.097)	0.066 (0.064)	BMI
rs6738445	2	172599615	0,99	C	T	0.013 (0.002)	-0.034 (0.02)	-0.031 (0.03)	-0.055 (0.033)	0.096 (0.087)	-0.042 (0.056)	BMI
rs2044469	2	174961488	0,99	G	A	0.013 (0.002)	0.007 (0.019)	-0.005 (0.028)	0 (0.031)	0.034 (0.081)	0.057 (0.054)	BMI
rs7588437	2	181575281	0,99	G	A	0.018 (0.002)	0.027 (0.019)	-0.004 (0.028)	0.046 (0.031)	0.157 (0.08)	0.028 (0.053)	BMI
rs6716898	2	198944271	0,99	A	G	0.016 (0.002)	0.047 (0.018)	0.028 (0.027)	0.069 (0.03)	-0.107 (0.077)	0.123 (0.051)	BMI
rs7593917	2	203931012	0,99	G	A	0.013 (0.002)	-0.024 (0.018)	0.006 (0.027)	-0.05 (0.03)	0.063 (0.078)	-0.1 (0.052)	BMI
rs10804139	2	205377705	0,95	A	G	0.033 (0.004)	0.098 (0.036)	0.059 (0.056)	0.171 (0.057)	0.095 (0.145)	-0.004 (0.102)	BMI
rs972540	2	207244783	1,00	G	A	0.012 (0.002)	-0.009 (0.02)	0.02 (0.031)	-0.029 (0.034)	0.049 (0.086)	-0.075 (0.058)	BMI
rs1263627	2	207977253	0,99	T	A	0.014 (0.002)	0.03 (0.021)	-0.01 (0.032)	0.091 (0.035)	-0.052 (0.093)	0.025 (0.06)	BMI
rs17203016	2	208255518	0,99	G	A	0.017 (0.002)	0.042 (0.022)	0.006 (0.033)	0.079 (0.037)	0.263 (0.099)	-0.024 (0.065)	BMI
rs4673553	2	211608379	1,00	G	T	0.015 (0.002)	0.012 (0.018)	-0.023 (0.027)	0.067 (0.03)	0.026 (0.077)	-0.028 (0.051)	BMI
rs7421089	2	211988412	0,99	T	C	0.015 (0.002)	0.012 (0.02)	0.011 (0.03)	0.009 (0.033)	0.064 (0.087)	0.007 (0.057)	BMI
rs16825005	2	212304565	1,00	A	G	0.014 (0.002)	0.012 (0.02)	0.048 (0.03)	0.011 (0.033)	-0.084 (0.084)	-0.068 (0.056)	BMI
rs7599312	2	213413231	1,00	G	A	0.018 (0.002)	0.016 (0.02)	0.035 (0.03)	0.017 (0.034)	0.144 (0.088)	-0.111 (0.058)	BMI
rs7607369	2	219279097	0,99	A	G	0.013 (0.002)	0.003 (0.018)	-0.013 (0.027)	0.018 (0.03)	0.051 (0.079)	-0.002 (0.053)	BMI
rs2276638	2	220145286	0,99	C	T	0.017 (0.002)	-0.026 (0.024)	-0.052 (0.036)	0.033 (0.04)	-0.054 (0.101)	-0.093 (0.067)	BMI
rs4500930	2	228985505	0,98	T	C	0.016 (0.002)	0.042 (0.019)	0.041 (0.029)	0 (0.031)	0.084 (0.082)	0.151 (0.055)	BMI
rs36191868	2	228999054	0,96	C	T	0.015 (0.002)	0.023 (0.018)	0.016 (0.028)	0.005 (0.03)	0.103 (0.08)	0.071 (0.052)	BMI
rs2433733	2	230816703	0,99	G	A	0.015 (0.002)	0.002 (0.019)	0.017 (0.028)	-0.034 (0.032)	0.099 (0.083)	0.01 (0.056)	BMI
rs112380819	3	9498519	0,99	A	G	0.024 (0.004)	0.014 (0.029)	0.078 (0.042)	-0.064 (0.048)	0.008 (0.122)	-0.01 (0.083)	BMI
rs6766610	3	11664859	0,99	C	A	0.011 (0.002)	-0.026 (0.018)	-0.006 (0.027)	-0.056 (0.03)	-0.009 (0.076)	-0.02 (0.051)	BMI
rs11709077	3	12336507	0,99	A	G	0.02 (0.003)	-0.026 (0.027)	-0.027 (0.039)	-0.008 (0.044)	-0.047 (0.119)	-0.076 (0.08)	BMI
rs7649970	3	12392272	1,00	T	C	0.02 (0.003)	-0.03 (0.026)	-0.033 (0.039)	-0.01 (0.044)	-0.034 (0.119)	-0.083 (0.079)	BMI
rs1801282	3	12393125	1,00	G	C	0.02 (0.003)	-0.028 (0.027)	-0.03 (0.039)	-0.011 (0.044)	-0.027 (0.119)	-0.076 (0.079)	BMI
rs765248	3	13001931	0,99	G	A	0.012 (0.002)	0.025 (0.018)	0.035 (0.027)	0.008 (0.03)	-0.016 (0.077)	0.06 (0.051)	BMI
rs2174172	3	15768579	1,00	C	A	0.012 (0.002)	-0.013 (0.018)	-0.013 (0.028)	-0.004 (0.03)	0.022 (0.079)	-0.052 (0.052)	BMI
rs4858193	3	20441050	0,97	T	C	0.014 (0.002)	0.037 (0.021)	0.012 (0.031)	0.04 (0.034)	0.054 (0.09)	0.108 (0.058)	BMI
rs6804842	3	25106437	0,98	G	A	0.015 (0.002)	0.003 (0.018)	0.012 (0.028)	0.007 (0.03)	0.001 (0.077)	-0.041 (0.052)	BMI
rs11921432	3	35117776	0,98	C	T	0.019 (0.003)	0.025 (0.029)	0.022 (0.045)	0.061 (0.047)	-0.119 (0.124)	-0.007 (0.08)	BMI
rs11129662	3	35696096	0,99	G	A	0.014 (0.002)	0.025 (0.019)	0.002 (0.028)	0.031 (0.032)	-0.012 (0.082)	0.106 (0.055)	BMI

rs10460960	3	42308735	0,98	A	G	0.02 (0.003)	-0.009 (0.028)	-0.009 (0.042)	0.029 (0.047)	-0.081 (0.121)	-0.088 (0.082)	BMI
rs33485	3	42417982	0,96	C	T	0.014 (0.002)	0.018 (0.021)	0.004 (0.031)	0.033 (0.034)	0.12 (0.091)	-0.022 (0.058)	BMI
rs6781618	3	44001933	1,00	C	T	0.016 (0.002)	0.03 (0.021)	0.005 (0.03)	0.068 (0.034)	-0.07 (0.092)	0.053 (0.06)	BMI
rs55676934	3	45222958	0,97	G	A	0.014 (0.002)	0.02 (0.019)	0.048 (0.029)	-0.011 (0.032)	-0.027 (0.083)	0.033 (0.055)	BMI
rs4683096	3	45374496	0,99	T	G	0.012 (0.002)	0 (0.018)	-0.023 (0.027)	0.011 (0.03)	-0.005 (0.077)	0.053 (0.052)	BMI
rs7429588	3	46835250	0,76	C	T	0.016 (0.003)	-0.012 (0.023)	NA (NA)	-0.028 (0.036)	-0.078 (0.094)	-0.016 (0.059)	BMI
rs72906474	3	47817007	0,91	G	T	0.017 (0.002)	-0.029 (0.019)	-0.029 (0.029)	-0.063 (0.032)	0.047 (0.081)	0.039 (0.054)	BMI
rs73078357	3	48695834	0,96	T	C	0.025 (0.004)	0.026 (0.028)	0.008 (0.041)	0.049 (0.045)	0.101 (0.123)	-0.012 (0.08)	BMI
rs12635454	3	48763656	0,75	A	G	0.026 (0.004)	0.028 (0.037)	0.029 (0.055)	0.065 (0.06)	0.026 (0.148)	-0.107 (0.112)	BMI
rs7623659	3	49414791	0,97	C	T	0.019 (0.002)	0.004 (0.02)	-0.006 (0.03)	0.006 (0.032)	0.041 (0.085)	0.015 (0.056)	BMI
rs9843653	3	49920571	0,99	C	T	0.03 (0.002)	0.026 (0.018)	0.005 (0.027)	0.015 (0.03)	0.126 (0.078)	0.097 (0.052)	BMI
rs7428430	3	50174184	0,99	C	T	0.025 (0.002)	0.012 (0.018)	-0.008 (0.027)	0.007 (0.03)	0.12 (0.078)	0.057 (0.052)	BMI
rs62260811	3	50435832	0,96	C	T	0.024 (0.004)	-0.036 (0.03)	-0.048 (0.046)	-0.046 (0.05)	0.184 (0.13)	-0.057 (0.083)	BMI
rs10433609	3	51311574	0,88	A	T	0.016 (0.003)	-0.026 (0.025)	-0.009 (0.036)	-0.028 (0.042)	0.017 (0.107)	-0.118 (0.076)	BMI
rs353547	3	52268866	0,99	T	C	0.011 (0.002)	0.011 (0.018)	-0.008 (0.027)	0.024 (0.03)	0.126 (0.08)	-0.009 (0.053)	BMI
rs2710323	3	52815905	1,00	C	T	0.013 (0.002)	0.012 (0.018)	0.012 (0.027)	0.009 (0.03)	-0.034 (0.077)	0.04 (0.052)	BMI
rs6798941	3	52893465	0,95	T	C	0.018 (0.002)	0.011 (0.02)	0 (0.03)	0.003 (0.033)	0.069 (0.087)	0.046 (0.057)	BMI
rs2680648	3	53777176	0,99	T	C	0.015 (0.002)	-0.006 (0.021)	0.004 (0.031)	-0.019 (0.035)	-0.004 (0.091)	-0.01 (0.06)	BMI
rs1586314	3	56196416	1,00	G	T	0.018 (0.003)	0.014 (0.031)	-0.013 (0.049)	0.039 (0.05)	0.226 (0.127)	-0.075 (0.085)	BMI
rs2365389	3	61236462	0,99	C	T	0.018 (0.002)	-0.008 (0.018)	-0.004 (0.027)	-0.012 (0.03)	-0.046 (0.079)	0.007 (0.053)	BMI
rs9968060	3	62471282	1,00	T	C	0.013 (0.002)	0.008 (0.019)	0.008 (0.027)	0.025 (0.031)	0.006 (0.079)	-0.036 (0.053)	BMI
rs475978	3	62714077	1,00	T	C	0.014 (0.002)	0.002 (0.022)	0.036 (0.032)	-0.028 (0.036)	-0.053 (0.094)	-0.018 (0.061)	BMI
rs56038322	3	69925128	0,89	A	G	0.014 (0.002)	-0.02 (0.021)	-0.047 (0.032)	0.022 (0.033)	-0.06 (0.087)	-0.042 (0.056)	BMI
rs11915371	3	70539559	1,00	C	A	0.016 (0.002)	0.032 (0.022)	0.04 (0.033)	0.017 (0.036)	0.136 (0.096)	0.006 (0.063)	BMI
rs1523768	3	77667044	1,00	G	A	0.013 (0.002)	0.021 (0.019)	0.007 (0.029)	0.026 (0.032)	0.039 (0.08)	0.048 (0.054)	BMI
rs876675	3	77675638	1,00	C	T	0.013 (0.002)	0.009 (0.018)	-0.025 (0.027)	0.034 (0.03)	0.067 (0.076)	0.03 (0.051)	BMI
rs6419734	3	78458928	0,95	T	C	0.017 (0.003)	0.003 (0.025)	0.053 (0.037)	-0.051 (0.042)	-0.044 (0.107)	-0.002 (0.076)	BMI
rs3773192	3	78660056	0,98	C	T	0.013 (0.002)	0.024 (0.02)	-0.004 (0.03)	0.074 (0.034)	0.006 (0.088)	-0.007 (0.058)	BMI
rs3849570	3	81792112	0,98	A	C	0.012 (0.002)	-0.038 (0.019)	-0.06 (0.029)	-0.032 (0.032)	0.033 (0.082)	-0.012 (0.054)	BMI
rs6794598	3	82570006	1,00	C	T	0.02 (0.003)	0.019 (0.024)	0.061 (0.036)	0.025 (0.04)	-0.139 (0.104)	-0.085 (0.069)	BMI
rs76162423	3	83445572	0,80	G	T	0.026 (0.004)	-0.031 (0.036)	-0.062 (0.054)	0.05 (0.059)	-0.34 (0.148)	-0.022 (0.099)	BMI
rs114593013	3	84113491	0,95	A	G	0.036 (0.005)	-0.004 (0.037)	-0.007 (0.055)	0.026 (0.062)	-0.098 (0.166)	-0.034 (0.102)	BMI
rs116032709	3	84628711	0,90	A	G	0.034 (0.005)	-0.023 (0.041)	-0.032 (0.062)	0.019 (0.069)	-0.191 (0.178)	-0.038 (0.114)	BMI
rs13095644	3	85141564	0,99	A	G	0.014 (0.002)	0 (0.018)	-0.005 (0.027)	0.035 (0.03)	-0.05 (0.077)	-0.064 (0.051)	BMI
rs7431895	3	85870501	1,00	T	C	0.02 (0.002)	0.008 (0.019)	0.055 (0.028)	-0.018 (0.031)	0.025 (0.081)	-0.096 (0.054)	BMI
rs12495178	3	85886077	1,00	T	C	0.02 (0.002)	0.01 (0.019)	0.057 (0.028)	-0.016 (0.031)	0.032 (0.081)	-0.098 (0.054)	BMI
rs17025214	3	88023541	0,95	C	T	0.015 (0.002)	-0.004 (0.02)	-0.002 (0.03)	-0.014 (0.033)	0.088 (0.084)	-0.025 (0.056)	BMI
rs1006896	3	88104411	0,95	A	C	0.023 (0.003)	0.045 (0.031)	0.021 (0.048)	0.064 (0.05)	0.117 (0.125)	0.036 (0.08)	BMI
rs62276243	3	90121534	0,98	C	T	0.015 (0.002)	0.056 (0.018)	0.079 (0.028)	0.049 (0.03)	-0.009 (0.082)	0.021 (0.052)	BMI
rs71324802	3	93517667	0,77	T	C	0.016 (0.003)	0.078 (0.024)	NA (NA)	0.064 (0.037)	-0.01 (0.105)	0.064 (0.062)	BMI
rs4857329	3	94036952	1,00	A	G	0.02 (0.002)	0.036 (0.018)	0.077 (0.027)	0.016 (0.03)	0.014 (0.079)	-0.048 (0.052)	BMI
rs1454687	3	94038085	1,00	C	G	0.02 (0.002)	0.036 (0.018)	0.077 (0.027)	0.015 (0.03)	0.012 (0.08)	-0.049 (0.052)	BMI
rs1436351	3	104617973	1,00	T	G	0.016 (0.002)	0.038 (0.021)	0.026 (0.032)	0.064 (0.034)	-0.084 (0.09)	0.059 (0.059)	BMI
rs35772758	3	107297351	0,99	C	T	0.018 (0.003)	-0.056 (0.024)	-0.053 (0.037)	0.001 (0.04)	-0.173 (0.099)	-0.168 (0.066)	BMI
rs7640424	3	107820063	0,97	C	T	0.014 (0.002)	0.021 (0.02)	0.004 (0.03)	0.036 (0.033)	-0.093 (0.086)	0.083 (0.056)	BMI
rs326358	3	107822310	0,97	T	C	0.013 (0.002)	0.013 (0.02)	-0.008 (0.029)	0.04 (0.032)	-0.078 (0.084)	0.048 (0.055)	BMI
rs17619973	3	114417675	0,97	A	G	0.024 (0.004)	-0.006 (0.035)	0.021 (0.051)	-0.007 (0.06)	-0.15 (0.148)	-0.044 (0.101)	BMI
rs2124499	3	123093541	0,99	G	C	0.013 (0.002)	0.025 (0.019)	0.032 (0.027)	0.019 (0.031)	0.029 (0.08)	0.015 (0.053)	BMI
rs9848399	3	125205106	1,00	A	G	0.018 (0.003)	0.046 (0.027)	0.101 (0.04)	0.002 (0.045)	0.027 (0.115)	-0.026 (0.078)	BMI

rs9864995	3	131602528	1,00	C	T	0.015 (0.002)	0.036 (0.019)	0.001 (0.028)	0.071 (0.031)	0.141 (0.081)	0.017 (0.052)	BMI
rs1320903	3	131758077	1,00	A	G	0.022 (0.002)	-0.002 (0.019)	-0.014 (0.029)	0.014 (0.032)	0.092 (0.083)	-0.047 (0.054)	BMI
rs10935143	3	134665159	0,99	G	A	0.012 (0.002)	-0.038 (0.018)	-0.031 (0.027)	-0.027 (0.03)	-0.107 (0.076)	-0.061 (0.052)	BMI
rs6775778	3	135726679	1,00	G	A	0.016 (0.002)	-0.023 (0.021)	-0.036 (0.031)	0.003 (0.035)	0.006 (0.09)	-0.063 (0.059)	BMI
rs9880211	3	136107549	1,00	A	G	0.019 (0.002)	-0.02 (0.021)	-0.036 (0.031)	-0.003 (0.034)	0.03 (0.089)	-0.035 (0.059)	BMI
rs7621025	3	136272246	1,00	C	T	0.02 (0.002)	0.002 (0.021)	-0.034 (0.032)	0.07 (0.035)	0.013 (0.088)	-0.07 (0.059)	BMI
rs1199334	3	138091140	1,00	A	G	0.015 (0.002)	0.014 (0.023)	0.005 (0.033)	0.005 (0.037)	0.073 (0.099)	0.056 (0.067)	BMI
rs16851483	3	141275436	1,00	T	G	0.036 (0.004)	0.024 (0.037)	-0.003 (0.053)	0.085 (0.063)	-0.072 (0.158)	0.003 (0.106)	BMI
rs171390	3	154038412	0,99	C	T	0.015 (0.002)	-0.002 (0.018)	-0.019 (0.027)	0.031 (0.03)	-0.052 (0.079)	-0.013 (0.052)	BMI
rs17367831	3	156297117	0,99	C	T	0.017 (0.003)	0.004 (0.026)	-0.018 (0.038)	0.015 (0.043)	0.074 (0.108)	0.02 (0.072)	BMI
rs6805114	3	156897572	0,99	G	A	0.013 (0.002)	0.048 (0.019)	0.039 (0.029)	0.057 (0.031)	0.039 (0.081)	0.052 (0.054)	BMI
rs9857883	3	157887272	1,00	A	C	0.012 (0.002)	0.011 (0.018)	0.023 (0.027)	0.034 (0.03)	-0.083 (0.079)	-0.059 (0.052)	BMI
rs541222	3	161478669	0,99	T	C	0.013 (0.002)	0.008 (0.018)	0.016 (0.027)	0.004 (0.03)	0.078 (0.078)	-0.037 (0.052)	BMI
rs8192675	3	170724883	1,00	C	T	0.018 (0.002)	0.017 (0.02)	0.029 (0.029)	0.025 (0.032)	-0.012 (0.083)	-0.033 (0.056)	BMI
rs583514	3	173114167	0,99	C	T	0.016 (0.002)	0.04 (0.018)	0.041 (0.027)	0.028 (0.03)	0.084 (0.077)	0.052 (0.052)	BMI
rs9872156	3	176337348	0,98	A	G	0.013 (0.002)	-0.007 (0.021)	-0.048 (0.031)	0.002 (0.034)	0.06 (0.089)	0.082 (0.06)	BMI
rs6443750	3	181329682	0,90	C	T	0.016 (0.002)	0.012 (0.024)	0.026 (0.038)	-0.012 (0.039)	0.006 (0.115)	0.04 (0.065)	BMI
rs263041	3	183522231	1,00	A	G	0.014 (0.002)	0.008 (0.019)	0.028 (0.028)	-0.012 (0.031)	0.007 (0.081)	-0.006 (0.052)	BMI
rs7620457	3	183747266	0,92	G	A	0.014 (0.002)	0.016 (0.023)	0.015 (0.034)	0.012 (0.038)	0.06 (0.101)	0.012 (0.064)	BMI
rs1516725	3	185824004	1,00	C	T	0.037 (0.003)	-0.046 (0.027)	-0.067 (0.04)	-0.057 (0.044)	0.082 (0.115)	0.003 (0.075)	BMI
rs11721261	3	194876993	0,94	C	T	0.015 (0.002)	0.038 (0.02)	0.064 (0.031)	0.022 (0.034)	0.071 (0.087)	-0.022 (0.058)	BMI
rs13124829	4	18531148	0,99	G	A	0.014 (0.002)	0.02 (0.019)	0.037 (0.028)	0.014 (0.032)	-0.09 (0.083)	0.021 (0.056)	BMI
rs1385639	4	20097334	0,98	G	A	0.028 (0.004)	0.033 (0.028)	0.036 (0.042)	0.083 (0.046)	-0.112 (0.123)	-0.068 (0.081)	BMI
rs28602597	4	20112947	0,97	A	G	0.027 (0.004)	0.017 (0.027)	0.024 (0.041)	0.067 (0.044)	-0.098 (0.117)	-0.106 (0.077)	BMI
rs73213501	4	28514830	0,98	A	C	0.022 (0.003)	0.026 (0.023)	0.057 (0.033)	-0.023 (0.037)	0.063 (0.097)	0.048 (0.068)	BMI
rs6448733	4	31028190	0,95	G	A	0.015 (0.002)	0.002 (0.019)	-0.014 (0.028)	0.021 (0.032)	-0.005 (0.081)	0.006 (0.056)	BMI
rs13132853	4	38680015	0,98	A	G	0.015 (0.002)	0.01 (0.02)	-0.013 (0.029)	0.025 (0.032)	0.006 (0.084)	0.048 (0.054)	BMI
rs10938397	4	45182527	0,98	G	A	0.032 (0.002)	0.013 (0.018)	0.042 (0.027)	0.009 (0.03)	-0.138 (0.08)	-0.011 (0.052)	BMI
rs2768950	4	49064487	0,99	A	G	0.012 (0.002)	0.009 (0.021)	0.042 (0.031)	0.007 (0.034)	-0.148 (0.088)	-0.03 (0.056)	BMI
rs784944	4	52927229	0,99	A	G	0.013 (0.002)	0.012 (0.021)	0.027 (0.031)	0.036 (0.034)	-0.102 (0.088)	-0.053 (0.057)	BMI
rs7655709	4	55499386	0,99	C	G	0.014 (0.002)	-0.023 (0.018)	-0.015 (0.027)	-0.028 (0.03)	-0.05 (0.077)	-0.025 (0.051)	BMI
rs17085463	4	65740387	0,98	G	A	0.015 (0.002)	-0.018 (0.019)	-0.011 (0.029)	-0.061 (0.032)	-0.033 (0.083)	0.091 (0.055)	BMI
rs2318543	4	67803263	1,00	A	G	0.018 (0.003)	0.011 (0.021)	0.026 (0.031)	0.051 (0.036)	-0.176 (0.092)	-0.078 (0.061)	BMI
rs6835324	4	77117309	0,98	A	G	0.02 (0.003)	0.081 (0.026)	0.03 (0.039)	0.163 (0.041)	-0.039 (0.107)	0.049 (0.074)	BMI
rs6534626	4	80810069	1,00	T	C	0.014 (0.002)	-0.023 (0.018)	-0.033 (0.027)	-0.012 (0.03)	-0.212 (0.08)	0.062 (0.052)	BMI
rs4148155	4	89054667	1,00	A	G	0.018 (0.003)	0 (0.029)	0.022 (0.043)	-0.026 (0.048)	-0.211 (0.122)	0.085 (0.078)	BMI
rs7685048	4	95027784	0,99	C	T	0.012 (0.002)	0.014 (0.018)	0.018 (0.027)	0.051 (0.03)	-0.02 (0.077)	-0.098 (0.052)	BMI
rs1229984	4	100239319	0,79	C	T	0.045 (0.008)	0.025 (0.047)	-0.001 (0.076)	0.072 (0.072)	0.076 (0.197)	-0.095 (0.14)	BMI
rs17199964	4	102707791	0,85	A	G	0.037 (0.004)	-0.025 (0.038)	-0.068 (0.061)	0.057 (0.059)	0.014 (0.144)	-0.165 (0.1)	BMI
rs13107325	4	103188709	0,97	T	C	0.051 (0.004)	-0.032 (0.035)	-0.048 (0.052)	-0.016 (0.058)	0.047 (0.146)	-0.053 (0.097)	BMI
rs10516497	4	103942714	1,00	A	C	0.014 (0.002)	0.038 (0.022)	0.067 (0.033)	0.02 (0.037)	0.137 (0.096)	-0.069 (0.064)	BMI
rs326889	4	112713436	0,99	C	T	0.012 (0.002)	0.02 (0.019)	0.071 (0.028)	-0.034 (0.03)	0.072 (0.078)	-0.024 (0.053)	BMI
rs4864201	4	130731284	0,99	T	C	0.015 (0.002)	0.009 (0.019)	0.022 (0.028)	0.034 (0.031)	-0.07 (0.08)	-0.08 (0.054)	BMI
rs1296328	4	137083193	0,95	A	C	0.017 (0.002)	0.009 (0.019)	-0.025 (0.028)	0.015 (0.03)	0.059 (0.081)	0.092 (0.053)	BMI
rs57800857	4	140863365	0,97	A	C	0.017 (0.002)	0.016 (0.019)	0.025 (0.028)	0.034 (0.032)	-0.084 (0.082)	-0.031 (0.055)	BMI
rs9997448	4	140870515	0,98	C	T	0.017 (0.002)	0.018 (0.019)	0.029 (0.028)	0.035 (0.031)	-0.068 (0.081)	-0.036 (0.055)	BMI
rs35107973	4	143125948	0,98	T	C	0.021 (0.004)	0.014 (0.028)	0.046 (0.041)	0 (0.047)	-0.239 (0.123)	0.038 (0.079)	BMI
rs6852570	4	143700358	1,00	T	G	0.013 (0.002)	-0.011 (0.019)	-0.031 (0.028)	0 (0.031)	-0.025 (0.079)	0.036 (0.053)	BMI
rs17019408	4	145433452	0,99	C	A	0.013 (0.002)	-0.017 (0.02)	-0.019 (0.03)	-0.005 (0.033)	-0.038 (0.086)	-0.03 (0.057)	BMI

rs1455137	4	145986668	1,00	C	A	0.012 (0.002)	-0.011 (0.018)	-0.015 (0.027)	-0.03 (0.031)	-0.112 (0.08)	0.109 (0.053)	BMI
rs11944523	4	147374979	1,00	A	G	0.013 (0.002)	-0.002 (0.02)	-0.003 (0.03)	-0.014 (0.034)	0.153 (0.09)	-0.025 (0.058)	BMI
rs750090	4	152931436	1,00	T	C	0.013 (0.002)	0.018 (0.019)	0.049 (0.028)	-0.019 (0.031)	0.094 (0.08)	-0.021 (0.053)	BMI
rs6536583	4	162145663	0,99	C	T	0.013 (0.002)	-0.025 (0.019)	0.011 (0.028)	-0.069 (0.03)	-0.046 (0.08)	-0.017 (0.053)	BMI
rs10032421	4	171052156	0,99	G	A	0.013 (0.002)	0.021 (0.019)	0.04 (0.028)	0.006 (0.031)	0.05 (0.081)	-0.016 (0.053)	BMI
rs1522569	4	171632637	1,00	T	G	0.015 (0.002)	-0.034 (0.023)	-0.017 (0.034)	-0.049 (0.039)	-0.147 (0.098)	0.002 (0.067)	BMI
rs6867471	5	3574564	0,86	C	T	0.013 (0.002)	0.013 (0.02)	0.01 (0.03)	0.024 (0.033)	-0.089 (0.088)	0.037 (0.058)	BMI
rs12109333	5	27166660	1,00	T	C	0.013 (0.002)	0.024 (0.02)	0.034 (0.029)	0.027 (0.033)	0.051 (0.085)	-0.032 (0.057)	BMI
rs782978	5	43146329	0,99	C	T	0.015 (0.002)	0.023 (0.02)	0.08 (0.031)	-0.02 (0.034)	-0.064 (0.088)	-0.021 (0.058)	BMI
rs12189178	5	50914726	0,97	T	C	0.035 (0.005)	-0.046 (0.05)	-0.076 (0.076)	-0.042 (0.081)	0.054 (0.189)	-0.011 (0.137)	BMI
rs4865796	5	53272664	0,99	G	A	0.012 (0.002)	-0.007 (0.02)	-0.013 (0.029)	0.008 (0.032)	0.007 (0.084)	-0.035 (0.056)	BMI
rs1503526	5	63020706	1,00	C	T	0.015 (0.002)	0.008 (0.018)	-0.007 (0.027)	0.022 (0.03)	0 (0.076)	0.03 (0.051)	BMI
rs6864168	5	63962299	0,99	T	C	0.014 (0.002)	-0.032 (0.018)	-0.017 (0.027)	-0.04 (0.03)	0.019 (0.078)	-0.084 (0.052)	BMI
rs27218	5	66207261	1,00	C	T	0.013 (0.002)	0.022 (0.021)	0.022 (0.031)	0 (0.034)	0.023 (0.087)	0.081 (0.057)	BMI
rs1600075	5	74418304	1,00	G	A	0.022 (0.002)	-0.005 (0.019)	-0.013 (0.028)	-0.011 (0.031)	-0.018 (0.079)	0.05 (0.054)	BMI
rs3843480	5	74624482	0,99	T	C	0.021 (0.002)	0.006 (0.019)	0.009 (0.028)	0.037 (0.031)	-0.131 (0.078)	-0.034 (0.053)	BMI
rs42854	5	74963277	1,00	C	G	0.026 (0.002)	0.023 (0.02)	0.025 (0.029)	0.038 (0.032)	-0.015 (0.084)	-0.01 (0.056)	BMI
rs2307111	5	75003678	1,00	T	C	0.026 (0.002)	0.022 (0.018)	0.021 (0.027)	0.037 (0.03)	-0.046 (0.079)	0.013 (0.053)	BMI
rs252748	5	77390494	0,97	C	T	0.014 (0.002)	-0.037 (0.019)	-0.04 (0.028)	-0.034 (0.03)	-0.018 (0.08)	-0.044 (0.053)	BMI
rs12514473	5	80818639	0,99	T	C	0.016 (0.002)	-0.026 (0.022)	-0.02 (0.033)	-0.017 (0.036)	-0.063 (0.094)	-0.056 (0.061)	BMI
rs323794	5	86796296	0,96	A	G	0.046 (0.007)	0.005 (0.056)	-0.013 (0.079)	-0.011 (0.096)	-0.068 (0.242)	0.197 (0.183)	BMI
rs6870983	5	87697533	0,99	C	T	0.024 (0.002)	0.004 (0.022)	0.005 (0.032)	0.01 (0.036)	0.064 (0.093)	-0.048 (0.064)	BMI
rs1501673	5	87963600	1,00	A	G	0.03 (0.003)	0.083 (0.026)	0.07 (0.038)	0.125 (0.044)	0.125 (0.112)	-0.006 (0.076)	BMI
rs16867703	5	88799143	1,00	G	T	0.016 (0.002)	0.016 (0.019)	0.013 (0.028)	0 (0.031)	-0.01 (0.08)	0.089 (0.053)	BMI
rs4869417	5	92544460	1,00	C	T	0.013 (0.002)	-0.002 (0.02)	-0.016 (0.029)	0.042 (0.034)	0.03 (0.085)	-0.091 (0.057)	BMI
rs7713317	5	95716722	1,00	G	A	0.014 (0.002)	-0.003 (0.02)	-0.024 (0.029)	0.014 (0.033)	-0.07 (0.084)	0.054 (0.057)	BMI
rs1837269	5	95859144	1,00	C	T	0.017 (0.002)	-0.003 (0.018)	-0.032 (0.027)	0.032 (0.03)	0.007 (0.078)	0.003 (0.052)	BMI
rs2161097	5	103945178	1,00	T	C	0.012 (0.002)	-0.004 (0.018)	-0.021 (0.027)	0.025 (0.03)	-0.041 (0.076)	-0.01 (0.052)	BMI
rs11742930	5	105774098	0,99	T	C	0.012 (0.002)	0.015 (0.018)	0.006 (0.027)	0.01 (0.03)	-0.011 (0.078)	0.077 (0.052)	BMI
rs11739877	5	105876806	1,00	T	C	0.012 (0.002)	0.015 (0.018)	0.046 (0.027)	0.002 (0.03)	-0.008 (0.079)	-0.052 (0.053)	BMI
rs252818	5	106725691	1,00	T	C	0.015 (0.002)	0.02 (0.023)	0.039 (0.033)	0.013 (0.038)	0.062 (0.098)	-0.054 (0.067)	BMI
rs40067	5	107439012	1,00	G	A	0.027 (0.003)	0.038 (0.024)	0.034 (0.036)	0.052 (0.039)	0.091 (0.105)	-0.014 (0.069)	BMI
rs459552	5	112176756	0,94	T	A	0.013 (0.002)	0.029 (0.022)	-0.002 (0.033)	0.052 (0.035)	0.054 (0.092)	0.054 (0.061)	BMI
rs351114	5	119368666	0,98	T	C	0.011 (0.002)	-0.001 (0.018)	0.007 (0.027)	0.005 (0.03)	-0.132 (0.078)	0.014 (0.053)	BMI
rs1582931	5	122657199	0,98	G	A	0.014 (0.002)	-0.019 (0.018)	-0.007 (0.028)	-0.048 (0.03)	-0.004 (0.078)	0.017 (0.052)	BMI
rs6864049	5	124330522	0,99	G	A	0.014 (0.002)	-0.002 (0.018)	-0.035 (0.027)	0.041 (0.03)	-0.064 (0.077)	0.018 (0.051)	BMI
rs329120	5	133861756	1,00	C	T	0.016 (0.002)	0.013 (0.018)	0.033 (0.027)	0.003 (0.03)	0.016 (0.08)	-0.03 (0.052)	BMI
rs13163306	5	136571959	1,00	G	A	0.012 (0.002)	-0.015 (0.018)	0.003 (0.027)	-0.025 (0.03)	-0.006 (0.077)	-0.057 (0.051)	BMI
rs13174863	5	139080745	0,92	G	A	0.02 (0.003)	-0.005 (0.026)	-0.029 (0.039)	0.032 (0.043)	-0.032 (0.111)	-0.014 (0.074)	BMI
rs2190788	5	144484261	0,98	T	G	0.014 (0.002)	-0.004 (0.02)	-0.019 (0.029)	0.008 (0.032)	0.102 (0.084)	-0.036 (0.056)	BMI
rs2964009	5	153211441	1,00	A	G	0.012 (0.002)	-0.024 (0.018)	-0.027 (0.027)	-0.021 (0.03)	0.104 (0.077)	-0.077 (0.052)	BMI
rs7715256	5	153537893	1,00	G	T	0.016 (0.002)	0.004 (0.018)	-0.008 (0.027)	0 (0.03)	-0.016 (0.078)	0.07 (0.051)	BMI
rs7730898	5	170459675	0,98	A	G	0.016 (0.002)	-0.004 (0.02)	-0.024 (0.03)	-0.011 (0.033)	-0.013 (0.087)	0.096 (0.058)	BMI
rs9463175	6	9510030	0,99	C	T	0.012 (0.002)	-0.004 (0.019)	0.02 (0.028)	-0.054 (0.031)	-0.049 (0.081)	0.08 (0.055)	BMI
rs2228213	6	12124855	1,00	G	A	0.014 (0.002)	0.011 (0.019)	0.005 (0.028)	-0.006 (0.031)	-0.038 (0.08)	0.099 (0.053)	BMI
rs11753081	6	20705590	1,00	T	G	0.014 (0.002)	0.013 (0.023)	0.043 (0.034)	-0.022 (0.039)	-0.103 (0.096)	0.06 (0.067)	BMI
rs7760082	6	21919387	1,00	G	A	0.013 (0.002)	0.043 (0.019)	0.038 (0.028)	0.067 (0.032)	0.137 (0.083)	-0.049 (0.054)	BMI
rs9466947	6	23868205	1,00	T	C	0.014 (0.002)	0.026 (0.019)	0.05 (0.028)	0.007 (0.032)	0.036 (0.083)	-0.016 (0.055)	BMI
rs2237228	6	26104630	0,99	T	C	0.022 (0.004)	-0.059 (0.03)	-0.044 (0.047)	-0.024 (0.049)	-0.118 (0.13)	-0.17 (0.078)	BMI

rs75499503	6	26145217	0,95	C	T	0.021 (0.003)	-0.006 (0.022)	-0.019 (0.031)	0.085 (0.036)	-0.241 (0.097)	-0.138 (0.065)	BMI
rs17739727	6	27329788	1,00	A	C	0.018 (0.003)	-0.002 (0.022)	-0.001 (0.033)	-0.004 (0.037)	0.124 (0.097)	-0.053 (0.065)	BMI
rs853681	6	28296650	1,00	A	C	0.016 (0.003)	-0.05 (0.026)	-0.043 (0.04)	-0.02 (0.043)	0.048 (0.113)	-0.186 (0.07)	BMI
rs150254595	6	29278406	0,99	A	G	0.018 (0.003)	-0.049 (0.026)	-0.015 (0.041)	-0.056 (0.043)	0.041 (0.113)	-0.158 (0.068)	BMI
rs4495304	6	31080718	1,00	T	C	0.023 (0.004)	-0.042 (0.033)	0.013 (0.047)	-0.044 (0.057)	-0.377 (0.141)	-0.123 (0.106)	BMI
rs62395827	6	31786730	0,97	C	T	0.031 (0.004)	-0.014 (0.033)	-0.058 (0.05)	-0.008 (0.054)	0.237 (0.141)	0.004 (0.085)	BMI
rs9267677	6	31892641	0,99	C	T	0.025 (0.003)	-0.02 (0.03)	-0.038 (0.043)	0.028 (0.051)	0.037 (0.129)	-0.112 (0.086)	BMI
rs7761464	6	33519417	1,00	A	C	0.017 (0.002)	-0.006 (0.021)	-0.009 (0.031)	-0.031 (0.034)	-0.003 (0.088)	0.074 (0.057)	BMI
rs210130	6	33533404	1,00	G	T	0.014 (0.002)	-0.004 (0.019)	0.014 (0.027)	-0.013 (0.031)	0.044 (0.081)	-0.064 (0.054)	BMI
rs2499714	6	34072215	0,94	T	C	0.021 (0.003)	0.023 (0.033)	-0.016 (0.05)	0.098 (0.053)	0.09 (0.143)	-0.098 (0.09)	BMI
rs2744974	6	34579431	0,99	T	C	0.026 (0.002)	0.028 (0.019)	-0.022 (0.029)	0.075 (0.031)	0.077 (0.082)	0.046 (0.054)	BMI
rs16894959	6	34825662	0,99	C	T	0.027 (0.003)	0.067 (0.026)	-0.029 (0.039)	0.149 (0.041)	0.166 (0.105)	0.108 (0.073)	BMI
rs2091074	6	35006409	1,00	T	C	0.017 (0.003)	0.04 (0.021)	0.038 (0.032)	0.045 (0.035)	0.016 (0.09)	0.042 (0.059)	BMI
rs12203818	6	35251317	0,93	G	A	0.02 (0.002)	0.038 (0.023)	0.016 (0.037)	0.07 (0.037)	0.041 (0.097)	0.009 (0.061)	BMI
rs2033529	6	40348653	1,00	G	A	0.022 (0.002)	0.008 (0.02)	0.021 (0.029)	-0.003 (0.033)	-0.049 (0.085)	0.016 (0.056)	BMI
rs9471333	6	40362023	1,00	C	T	0.024 (0.002)	-0.003 (0.018)	0.003 (0.027)	-0.008 (0.03)	-0.004 (0.078)	-0.012 (0.051)	BMI
rs7748777	6	41133806	1,00	A	G	0.012 (0.002)	0.02 (0.018)	0.012 (0.027)	0.045 (0.03)	-0.083 (0.078)	0.016 (0.052)	BMI
rs9357402	6	42674983	0,98	G	T	0.014 (0.002)	0.007 (0.018)	0.029 (0.027)	-0.018 (0.029)	-0.068 (0.078)	0.033 (0.052)	BMI
rs1358980	6	43764551	0,93	C	T	0.013 (0.002)	-0.027 (0.019)	-0.052 (0.029)	0.004 (0.03)	-0.001 (0.078)	-0.053 (0.052)	BMI
rs6903387	6	46348834	0,99	A	G	0.012 (0.002)	-0.031 (0.018)	-0.065 (0.027)	0.004 (0.03)	-0.034 (0.077)	-0.012 (0.051)	BMI
rs17665162	6	50275258	1,00	C	T	0.032 (0.004)	0.05 (0.037)	0.076 (0.052)	0.061 (0.066)	-0.155 (0.166)	-0.001 (0.11)	BMI
rs2207139	6	50845490	1,00	G	A	0.042 (0.002)	0.047 (0.023)	0.045 (0.033)	0.09 (0.039)	-0.126 (0.101)	-0.007 (0.068)	BMI
rs9473932	6	50857995	1,00	A	G	0.031 (0.002)	0.016 (0.02)	0.007 (0.029)	0.058 (0.033)	-0.099 (0.085)	-0.026 (0.056)	BMI
rs283579	6	50954071	1,00	G	A	0.019 (0.002)	-0.014 (0.02)	-0.022 (0.031)	0.018 (0.033)	0.011 (0.086)	-0.087 (0.056)	BMI
rs1775255	6	51243035	0,99	T	G	0.014 (0.002)	0.018 (0.018)	0.016 (0.027)	0.036 (0.03)	0.089 (0.078)	-0.062 (0.051)	BMI
rs9395747	6	51799902	0,99	G	T	0.016 (0.002)	0.032 (0.02)	0.043 (0.029)	0.039 (0.033)	-0.047 (0.087)	0 (0.057)	BMI
rs9688431	6	73922654	0,98	T	C	0.023 (0.004)	-0.037 (0.038)	-0.01 (0.056)	-0.08 (0.061)	-0.183 (0.163)	0.068 (0.112)	BMI
rs9294260	6	83433228	0,98	A	G	0.014 (0.002)	0.034 (0.018)	0.043 (0.028)	0.023 (0.03)	-0.058 (0.076)	0.077 (0.052)	BMI
rs9362662	6	90296588	0,99	A	G	0.013 (0.002)	0.02 (0.018)	-0.023 (0.027)	0.084 (0.03)	0.136 (0.076)	-0.067 (0.051)	BMI
rs9320518	6	97763117	0,98	G	A	0.017 (0.002)	-0.015 (0.02)	-0.019 (0.03)	-0.01 (0.033)	0.096 (0.085)	-0.068 (0.059)	BMI
rs9320823	6	98429337	0,99	C	T	0.018 (0.002)	0.015 (0.019)	0.006 (0.028)	0.001 (0.03)	-0.011 (0.078)	0.099 (0.053)	BMI
rs901630	6	98539519	0,97	C	T	0.017 (0.002)	0.01 (0.019)	0.007 (0.029)	-0.006 (0.03)	-0.034 (0.08)	0.087 (0.053)	BMI
rs156151	6	104799007	1,00	G	C	0.019 (0.002)	-0.037 (0.022)	-0.011 (0.033)	-0.031 (0.037)	-0.045 (0.098)	-0.156 (0.065)	BMI
rs768023	6	108876002	0,99	A	G	0.016 (0.002)	0.007 (0.018)	-0.002 (0.027)	0.011 (0.031)	0.113 (0.08)	-0.021 (0.052)	BMI
rs9387640	6	119508871	1,00	C	T	0.012 (0.002)	-0.018 (0.019)	-0.039 (0.028)	0.006 (0.031)	-0.085 (0.08)	0.021 (0.054)	BMI
rs2357760	6	120213880	1,00	A	G	0.014 (0.002)	0.017 (0.019)	0.017 (0.028)	0.023 (0.032)	0.073 (0.082)	-0.021 (0.055)	BMI
rs9388347	6	124934981	0,99	G	T	0.016 (0.003)	-0.009 (0.025)	0.022 (0.038)	-0.069 (0.041)	-0.08 (0.101)	0.089 (0.069)	BMI
rs111743285	6	127048230	0,98	T	C	0.016 (0.003)	0.005 (0.022)	0.003 (0.032)	0.012 (0.037)	-0.012 (0.094)	0.005 (0.062)	BMI
rs4538727	6	129408564	0,93	G	A	0.013 (0.002)	-0.025 (0.019)	-0.054 (0.028)	0.016 (0.031)	-0.022 (0.08)	-0.045 (0.054)	BMI
rs2246012	6	131898208	1,00	C	T	0.016 (0.002)	0.022 (0.024)	0.041 (0.036)	-0.027 (0.04)	0.07 (0.105)	0.08 (0.07)	BMI
rs11754747	6	141494602	0,87	T	C	0.013 (0.002)	0.039 (0.022)	0.057 (0.033)	0.006 (0.036)	0.144 (0.092)	0.024 (0.062)	BMI
rs12194370	6	143178146	0,97	A	G	0.016 (0.002)	-0.002 (0.023)	-0.005 (0.034)	0.046 (0.038)	-0.203 (0.096)	-0.034 (0.064)	BMI
rs2256216	6	147471730	0,98	G	A	0.012 (0.002)	0.009 (0.018)	0.019 (0.027)	0.028 (0.03)	-0.06 (0.077)	-0.054 (0.052)	BMI
rs2185027	6	153381622	1,00	C	A	0.015 (0.002)	0.019 (0.02)	0.007 (0.029)	0.037 (0.032)	-0.135 (0.085)	0.077 (0.057)	BMI
rs10499276	6	154309808	1,00	T	C	0.018 (0.003)	-0.016 (0.027)	-0.031 (0.039)	-0.03 (0.044)	0.067 (0.112)	0.045 (0.078)	BMI
rs13191362	6	163033350	0,99	A	G	0.024 (0.003)	0.015 (0.029)	0.046 (0.044)	-0.002 (0.047)	-0.032 (0.122)	-0.016 (0.077)	BMI
rs9356132	6	164102214	1,00	C	T	0.012 (0.002)	-0.002 (0.02)	0.034 (0.029)	-0.025 (0.032)	-0.085 (0.084)	-0.034 (0.057)	BMI
rs60426889	7	24471610	0,93	T	G	0.014 (0.002)	-0.018 (0.02)	-0.045 (0.029)	0.014 (0.032)	-0.057 (0.082)	0.003 (0.056)	BMI
rs10259620	7	27202289	1,00	A	G	0.016 (0.002)	-0.015 (0.022)	-0.008 (0.031)	-0.048 (0.036)	-0.034 (0.094)	0.063 (0.062)	BMI

rs849135	7	28196413	0,99	A	G	0.012 (0.002)	-0.032 (0.018)	-0.036 (0.027)	-0.044 (0.03)	0.035 (0.076)	-0.012 (0.051)	BMI
rs215632	7	32368524	1,00	A	G	0.014 (0.002)	0.028 (0.019)	-0.004 (0.028)	0.068 (0.031)	0.09 (0.08)	-0.001 (0.053)	BMI
rs12537044	7	39357892	1,00	T	G	0.017 (0.003)	0.015 (0.025)	0.008 (0.037)	0.047 (0.041)	-0.012 (0.107)	-0.044 (0.07)	BMI
rs799449	7	44784697	1,00	T	C	0.016 (0.002)	0.004 (0.018)	0.015 (0.027)	-0.036 (0.03)	0.085 (0.076)	0.049 (0.052)	BMI
rs10269783	7	49616203	0,99	A	G	0.012 (0.002)	-0.002 (0.018)	0.015 (0.027)	-0.023 (0.031)	0.128 (0.079)	-0.061 (0.052)	BMI
rs10499694	7	50614173	1,00	A	G	0.013 (0.002)	0.017 (0.018)	0.024 (0.027)	0.022 (0.03)	0.062 (0.074)	-0.044 (0.051)	BMI
rs6969375	7	69721267	0,99	C	T	0.014 (0.002)	0.006 (0.02)	-0.046 (0.03)	0.038 (0.034)	0.261 (0.091)	-0.003 (0.058)	BMI
rs10237317	7	70045941	0,93	G	A	0.013 (0.002)	-0.012 (0.019)	-0.018 (0.028)	-0.002 (0.032)	-0.019 (0.082)	-0.011 (0.054)	BMI
rs10807744	7	71641264	1,00	G	T	0.015 (0.002)	-0.021 (0.023)	-0.017 (0.033)	0.006 (0.038)	-0.104 (0.103)	-0.085 (0.067)	BMI
rs17207196	7	75101065	0,90	C	T	0.023 (0.002)	0.008 (0.019)	-0.032 (0.028)	0.028 (0.032)	0.01 (0.077)	0.1 (0.056)	BMI
rs1167800	7	75176196	0,99	A	G	0.021 (0.002)	0.001 (0.018)	-0.011 (0.027)	-0.015 (0.03)	0.031 (0.077)	0.081 (0.052)	BMI
rs11505821	7	76818677	0,89	T	A	0.03 (0.004)	-0.099 (0.04)	-0.127 (0.063)	-0.14 (0.062)	-0.025 (0.166)	0.102 (0.116)	BMI
rs740157	7	77055885	1,00	A	G	0.013 (0.002)	0.039 (0.018)	0.052 (0.027)	0.037 (0.03)	0.016 (0.077)	0.01 (0.051)	BMI
rs1852006	7	77829768	0,99	G	A	0.014 (0.002)	0.026 (0.019)	0.01 (0.028)	0.033 (0.031)	-0.009 (0.08)	0.081 (0.053)	BMI
rs7805441	7	78121458	1,00	T	C	0.012 (0.002)	0.014 (0.018)	0.01 (0.027)	0.035 (0.03)	-0.029 (0.076)	-0.015 (0.051)	BMI
rs2528531	7	93236510	0,96	C	A	0.014 (0.002)	-0.002 (0.019)	0.004 (0.029)	-0.014 (0.032)	0.162 (0.082)	-0.056 (0.055)	BMI
rs13240600	7	99064466	0,98	A	G	0.021 (0.003)	0.033 (0.025)	0.071 (0.037)	0.012 (0.041)	-0.203 (0.114)	0.047 (0.071)	BMI
rs2299383	7	103418846	1,00	T	C	0.016 (0.002)	0.015 (0.018)	0.03 (0.027)	0.004 (0.03)	-0.095 (0.077)	0.038 (0.051)	BMI
rs7788008	7	112972483	0,99	G	A	0.016 (0.002)	0.007 (0.018)	0.015 (0.027)	-0.004 (0.03)	-0.038 (0.078)	0.03 (0.052)	BMI
rs10953744	7	113516403	0,99	G	T	0.013 (0.002)	-0.018 (0.018)	-0.027 (0.028)	-0.016 (0.03)	0.013 (0.076)	-0.01 (0.051)	BMI
rs6950442	7	114412664	0,99	G	A	0.017 (0.002)	0.004 (0.022)	0.013 (0.032)	0.004 (0.036)	0.062 (0.095)	-0.051 (0.063)	BMI
rs1899689	7	121964349	1,00	T	C	0.013 (0.002)	-0.009 (0.018)	-0.001 (0.027)	-0.035 (0.03)	0.18 (0.08)	-0.046 (0.054)	BMI
rs896183	7	127831580	0,97	A	G	0.012 (0.002)	-0.017 (0.018)	-0.027 (0.027)	-0.005 (0.03)	0.002 (0.083)	-0.022 (0.052)	BMI
rs7810870	7	133581473	0,98	C	G	0.02 (0.003)	0.003 (0.027)	-0.023 (0.041)	0.012 (0.044)	0.111 (0.115)	0.02 (0.077)	BMI
rs1547958	7	150640285	1,00	C	T	0.015 (0.002)	0.014 (0.022)	0.006 (0.032)	0.033 (0.036)	-0.051 (0.092)	0.015 (0.059)	BMI
rs6601703	8	8380224	1,00	G	A	0.02 (0.002)	0.019 (0.018)	0.066 (0.027)	-0.035 (0.03)	-0.089 (0.078)	0.054 (0.051)	BMI
rs1825100	8	9146270	0,95	C	T	0.019 (0.002)	0.012 (0.019)	0.055 (0.028)	-0.03 (0.03)	-0.059 (0.081)	0.014 (0.053)	BMI
rs9657509	8	9620359	0,96	G	A	0.018 (0.002)	0.018 (0.02)	0.049 (0.03)	-0.021 (0.032)	0.055 (0.084)	0.014 (0.054)	BMI
rs615632	8	9796321	0,95	C	T	0.017 (0.002)	0.01 (0.018)	0.059 (0.028)	-0.054 (0.03)	0.009 (0.079)	0.026 (0.052)	BMI
rs6601527	8	10665444	0,97	C	A	0.021 (0.002)	0.001 (0.019)	0.037 (0.028)	-0.035 (0.031)	-0.059 (0.081)	0.002 (0.053)	BMI
rs11250162	8	11593222	0,99	C	T	0.019 (0.002)	0.015 (0.018)	0.057 (0.027)	-0.027 (0.03)	-0.084 (0.078)	0.032 (0.051)	BMI
rs13269000	8	14095199	1,00	C	T	0.015 (0.002)	-0.004 (0.019)	0.02 (0.028)	-0.042 (0.031)	0.016 (0.081)	0.007 (0.054)	BMI
rs13263601	8	14095900	1,00	C	A	0.015 (0.002)	-0.006 (0.019)	0.019 (0.028)	-0.042 (0.031)	0.011 (0.081)	0 (0.054)	BMI
rs11781222	8	23389571	0,98	T	C	0.018 (0.003)	0.011 (0.027)	0.023 (0.041)	-0.016 (0.045)	0.016 (0.108)	0.048 (0.076)	BMI
rs1425717	8	25657921	1,00	G	A	0.013 (0.002)	-0.006 (0.02)	0.014 (0.029)	0.016 (0.032)	-0.086 (0.084)	-0.106 (0.055)	BMI
rs2725371	8	30854033	1,00	A	G	0.018 (0.002)	0.013 (0.02)	0.002 (0.029)	-0.003 (0.033)	0.186 (0.085)	0.025 (0.056)	BMI
rs1362910	8	30856464	1,00	A	G	0.014 (0.002)	0.009 (0.018)	0.002 (0.027)	-0.013 (0.03)	0.147 (0.079)	0.037 (0.051)	BMI
rs7826312	8	32400115	0,99	C	T	0.012 (0.002)	-0.013 (0.018)	-0.037 (0.027)	0.01 (0.03)	0.045 (0.078)	-0.019 (0.051)	BMI
rs7844647	8	34503776	1,00	T	C	0.013 (0.002)	-0.001 (0.021)	-0.006 (0.031)	0.014 (0.034)	-0.024 (0.087)	-0.015 (0.057)	BMI
rs11784406	8	60856330	1,00	C	T	0.014 (0.002)	0.016 (0.019)	0.003 (0.028)	0.031 (0.031)	-0.001 (0.081)	0.025 (0.053)	BMI
rs6471941	8	62117973	1,00	A	G	0.017 (0.002)	0.042 (0.023)	0.046 (0.034)	0.019 (0.039)	0.112 (0.1)	0.067 (0.067)	BMI
rs11780222	8	67205241	1,00	A	C	0.015 (0.002)	0.024 (0.02)	0.022 (0.03)	0.01 (0.034)	0.105 (0.088)	0.034 (0.058)	BMI
rs35957544	8	73440371	0,90	G	T	0.021 (0.002)	0.005 (0.019)	-0.002 (0.028)	0.028 (0.032)	-0.096 (0.083)	0.009 (0.054)	BMI
rs2170382	8	74689288	0,98	T	C	0.019 (0.003)	0 (0.028)	-0.027 (0.042)	-0.039 (0.048)	0.166 (0.12)	0.137 (0.08)	BMI
rs2926614	8	76301610	1,00	C	T	0.019 (0.002)	0.044 (0.023)	0.043 (0.034)	0.055 (0.039)	-0.029 (0.102)	0.045 (0.067)	BMI
rs17405819	8	76806584	1,00	T	C	0.021 (0.002)	0.051 (0.02)	0.044 (0.029)	0.055 (0.033)	0.036 (0.086)	0.071 (0.056)	BMI
rs4326365	8	77340403	0,98	A	G	0.014 (0.002)	0.003 (0.02)	0.032 (0.03)	-0.029 (0.033)	0.077 (0.086)	-0.037 (0.058)	BMI
rs16907751	8	81375457	0,97	C	T	0.022 (0.003)	-0.04 (0.03)	-0.089 (0.042)	-0.007 (0.049)	0.039 (0.13)	0.03 (0.092)	BMI
rs733594	8	85077686	0,98	T	C	0.015 (0.002)	0.018 (0.02)	-0.003 (0.03)	0.034 (0.033)	0.027 (0.086)	0.048 (0.06)	BMI

rs7014897	8	85615059	0,99	A	G	0.014 (0.002)	-0.004 (0.022)	-0.015 (0.032)	0.005 (0.036)	0.058 (0.093)	-0.017 (0.061)	BMI
rs12546331	8	87505968	1,00	T	C	0.011 (0.002)	0.068 (0.018)	0.066 (0.027)	0.07 (0.03)	0.152 (0.077)	0.034 (0.052)	BMI
rs2174367	8	89408196	1,00	G	T	0.013 (0.002)	0.019 (0.019)	0.045 (0.028)	-0.02 (0.032)	-0.079 (0.084)	0.08 (0.056)	BMI
rs12680842	8	95582606	0,99	A	G	0.017 (0.002)	-0.001 (0.019)	0.013 (0.028)	-0.012 (0.032)	0.026 (0.082)	-0.031 (0.054)	BMI
rs1470764	8	101950038	0,98	A	G	0.012 (0.002)	0.001 (0.019)	0.006 (0.028)	-0.008 (0.03)	-0.045 (0.079)	0.034 (0.053)	BMI
rs2737250	8	116661874	1,00	A	G	0.015 (0.002)	-0.006 (0.019)	-0.029 (0.029)	0.022 (0.031)	-0.078 (0.081)	0.024 (0.054)	BMI
rs2721965	8	116662038	1,00	A	C	0.015 (0.002)	-0.007 (0.019)	-0.042 (0.029)	0.029 (0.031)	-0.057 (0.082)	0.032 (0.054)	BMI
rs2954021	8	126482077	1,00	G	A	0.011 (0.002)	0.001 (0.018)	0.017 (0.027)	-0.004 (0.03)	0.028 (0.08)	-0.057 (0.051)	BMI
rs4072917	8	143300279	0,97	A	G	0.012 (0.002)	-0.004 (0.018)	0.01 (0.027)	-0.043 (0.03)	0.105 (0.078)	0.008 (0.052)	BMI
rs7042372	9	6959840	0,98	A	G	0.012 (0.002)	0.04 (0.019)	0.029 (0.029)	0.048 (0.031)	0.112 (0.083)	0.024 (0.055)	BMI
rs10959687	9	11280652	0,98	A	G	0.012 (0.002)	0.038 (0.018)	0.026 (0.027)	0.037 (0.03)	0.076 (0.077)	0.067 (0.052)	BMI
rs17820822	9	11831420	1,00	T	G	0.014 (0.002)	0.074 (0.019)	0.056 (0.028)	0.071 (0.031)	0.067 (0.08)	0.146 (0.053)	BMI
rs4740619	9	15634326	0,99	T	C	0.019 (0.002)	0.029 (0.018)	0.005 (0.027)	0.066 (0.03)	0.157 (0.08)	-0.054 (0.052)	BMI
rs10481558	9	16715657	0,99	C	T	0.016 (0.002)	-0.011 (0.019)	0.003 (0.028)	0.009 (0.031)	-0.094 (0.081)	-0.09 (0.054)	BMI
rs10968104	9	27782517	0,99	T	C	0.013 (0.002)	0.029 (0.018)	0.012 (0.027)	0.024 (0.03)	0.143 (0.077)	0.051 (0.051)	BMI
rs10968576	9	28414339	1,00	G	A	0.025 (0.002)	-0.006 (0.019)	-0.001 (0.028)	-0.01 (0.032)	-0.015 (0.083)	-0.005 (0.054)	BMI
rs12378755	9	29318695	1,00	G	A	0.019 (0.003)	-0.008 (0.028)	0.015 (0.043)	-0.02 (0.047)	0.069 (0.123)	-0.088 (0.078)	BMI
rs10969334	9	29717279	0,99	C	A	0.012 (0.002)	0.012 (0.018)	0.014 (0.027)	-0.001 (0.03)	0.105 (0.08)	0.003 (0.053)	BMI
rs7848702	9	31210051	0,99	T	C	0.013 (0.002)	0.018 (0.021)	-0.002 (0.031)	-0.001 (0.034)	0.162 (0.088)	0.085 (0.058)	BMI
rs10971721	9	33827694	0,99	C	T	0.021 (0.003)	-0.036 (0.028)	-0.045 (0.041)	-0.027 (0.047)	-0.006 (0.121)	-0.039 (0.085)	BMI
rs13290794	9	37183628	1,00	G	A	0.014 (0.002)	0.04 (0.019)	0.023 (0.028)	0.086 (0.031)	-0.094 (0.08)	0.024 (0.053)	BMI
rs12339822	9	92187178	0,98	G	A	0.015 (0.002)	0.048 (0.018)	0.071 (0.027)	0 (0.03)	0.053 (0.078)	0.111 (0.052)	BMI
rs10992854	9	96437565	1,00	T	C	0.018 (0.002)	-0.025 (0.019)	-0.013 (0.029)	-0.03 (0.032)	-0.08 (0.08)	-0.025 (0.054)	BMI
rs9650755	9	96484342	0,99	G	A	0.02 (0.002)	-0.004 (0.02)	-0.024 (0.03)	0.006 (0.034)	0.004 (0.086)	0.037 (0.059)	BMI
rs420158	9	101477500	0,95	C	T	0.013 (0.002)	-0.022 (0.021)	-0.017 (0.031)	-0.015 (0.035)	-0.155 (0.092)	-0.002 (0.06)	BMI
rs12551906	9	102119090	0,98	G	A	0.012 (0.002)	0.016 (0.02)	0.007 (0.031)	0.032 (0.033)	0.062 (0.082)	-0.018 (0.056)	BMI
rs959893	9	103096666	1,00	G	A	0.015 (0.002)	-0.023 (0.018)	-0.051 (0.027)	0.002 (0.03)	-0.095 (0.078)	0.042 (0.051)	BMI
rs7024334	9	109072075	1,00	T	G	0.016 (0.002)	0.009 (0.022)	-0.028 (0.031)	0.021 (0.036)	-0.013 (0.096)	0.13 (0.062)	BMI
rs6477694	9	111932342	0,99	C	T	0.014 (0.002)	0.013 (0.019)	0.013 (0.028)	0.015 (0.031)	-0.01 (0.081)	0.017 (0.054)	BMI
rs1928295	9	120378483	1,00	T	C	0.014 (0.002)	0.034 (0.018)	0.027 (0.027)	0.054 (0.03)	-0.019 (0.077)	0.02 (0.052)	BMI
rs10760280	9	126112812	0,98	T	C	0.013 (0.002)	0.019 (0.018)	0.003 (0.027)	0.05 (0.03)	0.005 (0.077)	-0.008 (0.052)	BMI
rs682929	9	126625509	0,99	C	T	0.016 (0.003)	0.002 (0.024)	0.021 (0.033)	-0.061 (0.04)	0.132 (0.112)	0.061 (0.076)	BMI
rs10733682	9	129460914	1,00	A	G	0.015 (0.002)	0.009 (0.018)	0.015 (0.026)	-0.007 (0.03)	0.072 (0.078)	0.003 (0.051)	BMI
rs4734	9	131018639	0,95	G	T	0.02 (0.003)	0.016 (0.025)	-0.003 (0.038)	0.054 (0.041)	-0.098 (0.106)	0.024 (0.072)	BMI
rs4740383	9	133783566	0,99	A	G	0.014 (0.002)	-0.018 (0.018)	-0.026 (0.027)	-0.006 (0.03)	0.053 (0.079)	-0.05 (0.052)	BMI
rs6602411	10	10264200	0,91	T	C	0.016 (0.003)	-0.011 (0.025)	0.01 (0.04)	-0.014 (0.038)	-0.01 (0.098)	-0.062 (0.066)	BMI
rs7893571	10	16750129	0,98	T	G	0.013 (0.002)	0.005 (0.019)	-0.021 (0.029)	0.021 (0.032)	-0.014 (0.083)	0.054 (0.055)	BMI
rs11012732	10	21830104	0,98	G	A	0.024 (0.002)	0.046 (0.019)	0.045 (0.028)	0.054 (0.032)	0.162 (0.083)	-0.02 (0.054)	BMI
rs12762056	10	33969962	1,00	C	T	0.016 (0.003)	-0.018 (0.025)	-0.03 (0.036)	0.012 (0.042)	-0.065 (0.107)	-0.043 (0.07)	BMI
rs12765914	10	34013507	0,99	T	C	0.023 (0.004)	-0.012 (0.032)	0.001 (0.048)	-0.029 (0.054)	-0.085 (0.143)	0.018 (0.091)	BMI
rs10763953	10	34072703	0,99	A	T	0.012 (0.002)	0.004 (0.019)	0.012 (0.029)	0.024 (0.032)	-0.052 (0.082)	-0.061 (0.055)	BMI
rs1937684	10	53680085	1,00	A	T	0.012 (0.002)	0.008 (0.019)	-0.009 (0.028)	0.008 (0.031)	0.078 (0.082)	0.038 (0.054)	BMI
rs10761687	10	64789407	0,99	T	C	0.013 (0.002)	-0.002 (0.019)	-0.064 (0.029)	0.043 (0.032)	0.001 (0.083)	0.089 (0.055)	BMI
rs2163188	10	65314711	0,99	C	G	0.015 (0.002)	0.011 (0.018)	-0.021 (0.027)	0.022 (0.03)	0.02 (0.077)	0.09 (0.051)	BMI
rs12098284	10	76047464	0,97	T	C	0.019 (0.003)	-0.053 (0.028)	-0.04 (0.043)	-0.063 (0.046)	-0.093 (0.119)	-0.046 (0.081)	BMI
rs2002023	10	76848524	0,99	T	C	0.012 (0.002)	-0.01 (0.018)	-0.005 (0.027)	0.002 (0.03)	-0.126 (0.079)	-0.016 (0.052)	BMI
rs11001963	10	78760959	0,99	T	C	0.012 (0.002)	-0.01 (0.018)	-0.004 (0.027)	-0.044 (0.03)	0.04 (0.079)	0.048 (0.052)	BMI
rs7899106	10	87410904	0,99	G	A	0.033 (0.004)	0.067 (0.044)	-0.012 (0.069)	0.085 (0.07)	0.08 (0.18)	0.234 (0.116)	BMI
rs2114824	10	88119015	1,00	G	A	0.014 (0.002)	0.01 (0.018)	0.003 (0.027)	0.035 (0.03)	0.004 (0.077)	-0.037 (0.052)	BMI

rs2450444	10	93010383	0,99	G	A	0.012 (0.002)	-0.011 (0.019)	-0.002 (0.028)	-0.002 (0.031)	-0.087 (0.08)	-0.041 (0.054)	BMI
rs2439823	10	99778226	0,98	G	A	0.018 (0.002)	0.036 (0.018)	0.075 (0.027)	-0.022 (0.03)	-0.024 (0.079)	0.088 (0.052)	BMI
rs1867073	10	99793012	0,96	G	A	0.017 (0.002)	0.012 (0.02)	0.018 (0.03)	0.018 (0.032)	-0.05 (0.086)	-0.002 (0.055)	BMI
rs17094222	10	102395440	0,97	C	T	0.019 (0.002)	0.064 (0.022)	0.03 (0.034)	0.068 (0.037)	0.036 (0.096)	0.171 (0.062)	BMI
rs11190643	10	102432710	0,96	G	A	0.016 (0.002)	0.063 (0.022)	0.042 (0.032)	0.069 (0.036)	0.008 (0.092)	0.148 (0.062)	BMI
rs41310284	10	102447647	0,91	C	A	0.027 (0.004)	0.01 (0.033)	0.072 (0.048)	-0.022 (0.054)	-0.231 (0.14)	-0.034 (0.104)	BMI
rs7083450	10	103984060	1,00	T	C	0.017 (0.002)	-0.012 (0.025)	-0.001 (0.038)	-0.012 (0.041)	-0.029 (0.101)	-0.039 (0.071)	BMI
rs12411886	10	104685299	1,00	A	C	0.027 (0.003)	-0.013 (0.031)	-0.012 (0.046)	-0.018 (0.052)	0.276 (0.136)	-0.138 (0.094)	BMI
rs4132670	10	114767771	0,99	G	A	0.02 (0.002)	0.042 (0.02)	0.057 (0.03)	0.05 (0.032)	-0.102 (0.086)	0.022 (0.056)	BMI
rs740600	10	118651882	1,00	T	C	0.015 (0.002)	-0.004 (0.021)	-0.002 (0.03)	-0.018 (0.034)	0.027 (0.09)	0.015 (0.06)	BMI
rs845084	10	125220036	0,99	A	G	0.016 (0.002)	0.01 (0.02)	0.027 (0.03)	-0.033 (0.033)	0.175 (0.085)	0.001 (0.058)	BMI
rs17636031	10	126594078	0,84	C	T	0.015 (0.002)	0.04 (0.022)	0.041 (0.034)	0.064 (0.036)	0.03 (0.092)	-0.03 (0.061)	BMI
rs3781445	10	126688370	0,98	G	A	0.012 (0.002)	0.027 (0.018)	0.033 (0.027)	0.029 (0.03)	-0.053 (0.078)	0.037 (0.053)	BMI
rs7096307	10	133983427	0,95	C	T	0.015 (0.002)	0.011 (0.019)	-0.001 (0.028)	0.042 (0.03)	-0.082 (0.079)	-0.001 (0.052)	BMI
rs4963120	11	825777	0,97	T	C	0.013 (0.002)	0.008 (0.019)	0.01 (0.028)	0.026 (0.031)	-0.044 (0.08)	-0.022 (0.052)	BMI
rs2316901	11	8679016	1,00	G	A	0.018 (0.002)	0.049 (0.019)	0.094 (0.028)	0.051 (0.031)	0.052 (0.081)	-0.137 (0.055)	BMI
rs10766077	11	13349781	0,99	G	A	0.017 (0.002)	0.048 (0.019)	0.035 (0.028)	0.029 (0.03)	0.092 (0.08)	0.132 (0.053)	BMI
rs11023948	11	16481965	0,97	C	T	0.015 (0.002)	-0.013 (0.023)	-0.038 (0.034)	0.062 (0.038)	-0.156 (0.1)	-0.087 (0.063)	BMI
rs1557765	11	17403639	1,00	C	T	0.014 (0.002)	-0.014 (0.018)	-0.026 (0.027)	-0.022 (0.031)	0.011 (0.08)	0.042 (0.054)	BMI
rs17309874	11	27667236	0,97	A	G	0.031 (0.002)	-0.018 (0.021)	-0.061 (0.032)	0.022 (0.035)	-0.099 (0.089)	0.041 (0.059)	BMI
rs6265	11	27679916	1,00	C	T	0.042 (0.002)	0.042 (0.023)	0.013 (0.035)	0.076 (0.038)	-0.169 (0.1)	0.132 (0.064)	BMI
rs570463	11	28739318	0,99	C	A	0.013 (0.002)	0.001 (0.02)	-0.024 (0.029)	0.021 (0.033)	0.062 (0.082)	0.002 (0.055)	BMI
rs2211018	11	30330079	1,00	T	C	0.015 (0.002)	-0.011 (0.022)	0.034 (0.033)	-0.037 (0.037)	-0.152 (0.095)	-0.038 (0.062)	BMI
rs2065418	11	30422068	0,99	T	G	0.014 (0.002)	0.021 (0.019)	0.044 (0.028)	0.009 (0.031)	-0.054 (0.082)	0.002 (0.053)	BMI
rs13642	11	30432220	0,99	A	T	0.014 (0.002)	0.023 (0.019)	0.045 (0.028)	0.013 (0.031)	-0.063 (0.082)	0.008 (0.053)	BMI
rs7116641	11	43696917	0,99	G	T	0.021 (0.002)	0.016 (0.02)	-0.015 (0.029)	0.03 (0.032)	0.084 (0.084)	0.051 (0.054)	BMI
rs10742752	11	45438374	1,00	C	T	0.012 (0.002)	-0.018 (0.018)	-0.034 (0.027)	-0.02 (0.03)	0.119 (0.078)	-0.013 (0.052)	BMI
rs7124681	11	47529947	0,99	A	C	0.025 (0.002)	-0.007 (0.018)	-0.006 (0.028)	-0.012 (0.03)	0.001 (0.078)	0 (0.053)	BMI
rs7942074	11	47720509	1,00	T	C	0.022 (0.002)	-0.021 (0.018)	-0.025 (0.027)	-0.035 (0.03)	0.102 (0.078)	-0.019 (0.051)	BMI
rs1483121	11	48333360	1,00	G	A	0.015 (0.003)	-0.008 (0.025)	-0.034 (0.037)	0.007 (0.042)	0.121 (0.111)	-0.006 (0.073)	BMI
rs893006	11	64365796	0,99	A	C	0.014 (0.002)	-0.024 (0.02)	-0.056 (0.029)	-0.001 (0.032)	0.039 (0.084)	-0.001 (0.057)	BMI
rs506338	11	64440920	1,00	C	T	0.014 (0.002)	-0.035 (0.02)	-0.063 (0.029)	-0.014 (0.032)	0.016 (0.083)	-0.014 (0.056)	BMI
rs61754785	11	65632507	0,84	G	A	0.047 (0.008)	0.064 (0.075)	NA (NA)	0.197 (0.111)	-0.109 (0.301)	0.234 (0.176)	BMI
rs2234458	11	65639374	0,98	C	T	0.021 (0.002)	0.052 (0.019)	0.062 (0.029)	0.039 (0.031)	0.035 (0.082)	0.06 (0.054)	BMI
rs10908197	11	69439730	0,98	G	A	0.015 (0.002)	0.03 (0.018)	0.027 (0.027)	0.055 (0.03)	-0.071 (0.078)	0.01 (0.052)	BMI
rs592483	11	69445173	0,97	C	T	0.015 (0.002)	0.02 (0.018)	0.027 (0.027)	0.036 (0.03)	-0.11 (0.079)	0.005 (0.052)	BMI
rs667515	11	69449076	0,87	G	C	0.016 (0.002)	0.027 (0.02)	0.051 (0.03)	0.044 (0.032)	-0.231 (0.09)	-0.006 (0.055)	BMI
rs10898319	11	84613804	0,90	C	T	0.014 (0.002)	0.02 (0.019)	0.038 (0.028)	0.024 (0.032)	0.039 (0.084)	-0.066 (0.054)	BMI
rs61903695	11	89922417	1,00	G	A	0.017 (0.003)	0.034 (0.021)	0.029 (0.032)	0.063 (0.035)	-0.068 (0.092)	0.012 (0.058)	BMI
rs11214503	11	112986754	1,00	T	C	0.013 (0.002)	0.043 (0.018)	0.072 (0.027)	0.021 (0.03)	0 (0.079)	0.019 (0.052)	BMI
rs1048932	11	115044850	1,00	C	A	0.018 (0.002)	0.014 (0.018)	-0.006 (0.027)	0.03 (0.03)	-0.042 (0.078)	0.065 (0.052)	BMI
rs74881320	11	116765925	0,99	C	A	0.03 (0.005)	0.072 (0.037)	0.121 (0.055)	0.003 (0.062)	0.255 (0.168)	0.017 (0.107)	BMI
rs1786141	11	118938315	0,98	T	C	0.014 (0.002)	-0.006 (0.018)	-0.007 (0.027)	0.006 (0.03)	-0.086 (0.08)	-0.001 (0.052)	BMI
rs693701	11	119804461	1,00	T	C	0.013 (0.002)	0.001 (0.021)	0.036 (0.03)	0.007 (0.035)	-0.199 (0.091)	-0.068 (0.059)	BMI
rs579682	11	122014110	1,00	C	T	0.013 (0.002)	-0.002 (0.02)	-0.029 (0.03)	0.036 (0.033)	-0.03 (0.086)	-0.008 (0.056)	BMI
rs7941030	11	122522375	1,00	C	T	0.011 (0.002)	0.009 (0.019)	0.027 (0.027)	-0.023 (0.031)	0.021 (0.081)	0.031 (0.053)	BMI
rs7944782	11	130795698	0,97	G	T	0.014 (0.002)	-0.005 (0.018)	0.008 (0.027)	-0.018 (0.03)	0.135 (0.077)	-0.081 (0.052)	BMI
rs2512885	11	131467794	0,97	C	T	0.011 (0.002)	0.017 (0.018)	0.003 (0.028)	0.005 (0.03)	0.076 (0.078)	0.074 (0.052)	BMI
rs11222940	11	131985721	1,00	C	A	0.013 (0.002)	0.01 (0.02)	-0.008 (0.029)	0.017 (0.032)	0.089 (0.084)	0.016 (0.054)	BMI

rs2187449	11	133712682	0,97	A	G	0.017 (0.002)	0.03 (0.022)	0.022 (0.035)	0.02 (0.036)	-0.062 (0.096)	0.121 (0.06)	BMI
rs12806052	11	134518868	0,99	C	T	0.02 (0.003)	0.017 (0.025)	0.031 (0.037)	0.007 (0.041)	-0.006 (0.107)	0.011 (0.07)	BMI
rs12364470	11	134601012	0,96	G	T	0.019 (0.002)	-0.012 (0.024)	-0.019 (0.036)	0.032 (0.041)	-0.159 (0.101)	-0.05 (0.07)	BMI
rs11611246	12	939480	0,92	T	G	0.021 (0.002)	0.012 (0.023)	-0.033 (0.036)	-0.012 (0.037)	0.044 (0.099)	0.207 (0.063)	BMI
rs765123	12	2155997	0,95	G	A	0.018 (0.003)	0.039 (0.022)	0.01 (0.033)	0.069 (0.038)	-0.052 (0.097)	0.111 (0.066)	BMI
rs12422552	12	14413931	0,97	G	C	0.015 (0.002)	-0.004 (0.021)	0.005 (0.032)	0.002 (0.034)	-0.198 (0.088)	0.035 (0.059)	BMI
rs1983303	12	19257284	0,97	C	T	0.013 (0.002)	0.03 (0.02)	0.055 (0.03)	0.009 (0.033)	0.035 (0.087)	-0.007 (0.057)	BMI
rs11046979	12	23724688	1,00	T	C	0.016 (0.003)	0.026 (0.02)	-0.011 (0.029)	0.059 (0.033)	0.177 (0.089)	0.004 (0.059)	BMI
rs7970953	12	24075508	1,00	A	G	0.013 (0.002)	0.001 (0.02)	-0.009 (0.029)	0.002 (0.033)	0.054 (0.086)	0.015 (0.056)	BMI
rs11170468	12	39430048	1,00	A	C	0.015 (0.002)	0.012 (0.021)	0.027 (0.031)	-0.016 (0.035)	0.049 (0.091)	0.014 (0.06)	BMI
rs2733287	12	41880909	1,00	C	A	0.016 (0.002)	-0.028 (0.018)	-0.022 (0.027)	-0.014 (0.03)	-0.058 (0.076)	-0.075 (0.051)	BMI
rs7138803	12	50247468	1,00	A	G	0.03 (0.002)	-0.01 (0.018)	-0.02 (0.027)	0.021 (0.03)	-0.129 (0.078)	-0.009 (0.053)	BMI
rs4759075	12	54667285	1,00	T	C	0.011 (0.002)	-0.002 (0.018)	-0.025 (0.027)	0.005 (0.03)	-0.038 (0.08)	0.077 (0.052)	BMI
rs10783779	12	56491880	1,00	T	G	0.016 (0.002)	0.032 (0.018)	0.028 (0.027)	0.05 (0.03)	0.018 (0.079)	0 (0.052)	BMI
rs2292238	12	56493822	0,99	A	C	0.019 (0.002)	0.034 (0.018)	0.033 (0.027)	0.05 (0.031)	0.013 (0.08)	-0.002 (0.052)	BMI
rs1819844	12	68205604	1,00	A	G	0.014 (0.002)	0.03 (0.023)	0.022 (0.034)	-0.006 (0.038)	0.035 (0.102)	0.167 (0.067)	BMI
rs650198	12	69674595	1,00	C	T	0.013 (0.002)	0.041 (0.02)	0.048 (0.03)	0.036 (0.032)	0.06 (0.084)	0.022 (0.058)	BMI
rs2279574	12	89745477	0,98	C	A	0.015 (0.002)	-0.002 (0.018)	0.006 (0.028)	-0.004 (0.03)	0.083 (0.077)	-0.06 (0.051)	BMI
rs67524827	12	90136912	0,98	G	A	0.017 (0.003)	0.011 (0.021)	0.018 (0.031)	-0.018 (0.034)	0.061 (0.089)	0.051 (0.058)	BMI
rs2731251	12	90621658	0,99	T	C	0.013 (0.002)	-0.005 (0.021)	-0.011 (0.031)	0 (0.034)	-0.002 (0.087)	0 (0.059)	BMI
rs11105839	12	91237920	0,99	T	A	0.011 (0.002)	-0.011 (0.019)	-0.001 (0.027)	-0.011 (0.031)	-0.062 (0.08)	-0.028 (0.053)	BMI
rs1420341	12	97913785	0,97	C	T	0.016 (0.003)	-0.003 (0.024)	0.006 (0.037)	0.029 (0.039)	-0.069 (0.106)	-0.107 (0.068)	BMI
rs481902	12	99547717	0,99	G	A	0.014 (0.002)	0.009 (0.019)	0.006 (0.027)	0.025 (0.031)	0.107 (0.08)	-0.067 (0.053)	BMI
rs7488867	12	103699685	1,00	C	T	0.022 (0.002)	0.022 (0.021)	0.026 (0.031)	0.028 (0.034)	-0.025 (0.089)	0.012 (0.06)	BMI
rs10861861	12	108436396	1,00	T	G	0.017 (0.002)	-0.003 (0.022)	-0.052 (0.032)	0.01 (0.036)	0.077 (0.095)	0.11 (0.061)	BMI
rs1502337	12	111062852	0,99	T	C	0.013 (0.002)	-0.026 (0.019)	-0.033 (0.029)	-0.045 (0.031)	0.078 (0.079)	0.006 (0.053)	BMI
rs11065987	12	112072424	0,96	A	G	0.014 (0.002)	0.008 (0.019)	0.03 (0.028)	-0.038 (0.03)	0.07 (0.08)	0.05 (0.052)	BMI
rs11066188	12	112610714	0,90	G	A	0.013 (0.002)	0.003 (0.02)	0.024 (0.031)	-0.045 (0.03)	0.065 (0.079)	0.062 (0.054)	BMI
rs2891403	12	113137572	0,89	G	A	0.014 (0.002)	0.005 (0.021)	0.036 (0.032)	-0.041 (0.034)	-0.029 (0.091)	0.051 (0.059)	BMI
rs11065502	12	121677314	0,98	C	G	0.016 (0.003)	0.049 (0.026)	0.048 (0.04)	0.028 (0.043)	0.161 (0.107)	0.057 (0.072)	BMI
rs11057405	12	122781897	0,97	G	A	0.031 (0.003)	-0.044 (0.029)	-0.018 (0.043)	-0.046 (0.049)	-0.116 (0.134)	-0.109 (0.082)	BMI
rs7301515	12	122949673	0,99	C	T	0.019 (0.002)	-0.023 (0.021)	-0.016 (0.03)	-0.005 (0.034)	-0.108 (0.088)	-0.067 (0.059)	BMI
rs11060344	12	123329101	0,70	A	G	0.025 (0.003)	0.018 (0.031)	NA (NA)	0.029 (0.048)	-0.054 (0.12)	-0.098 (0.085)	BMI
rs7133378	12	124409502	0,98	A	G	0.014 (0.002)	-0.017 (0.02)	-0.019 (0.029)	-0.008 (0.032)	0.001 (0.084)	-0.044 (0.055)	BMI
rs10773049	12	124506631	1,00	C	T	0.013 (0.002)	-0.01 (0.018)	-0.016 (0.027)	-0.014 (0.03)	-0.016 (0.078)	0.03 (0.052)	BMI
rs10902511	12	132689436	0,99	T	C	0.016 (0.003)	-0.008 (0.023)	0.002 (0.034)	0.002 (0.037)	-0.004 (0.097)	-0.077 (0.065)	BMI
rs11614340	12	133426483	1,00	C	T	0.014 (0.002)	0.012 (0.02)	0.044 (0.029)	-0.047 (0.032)	-0.027 (0.085)	0.088 (0.056)	BMI
rs7323	13	28009031	0,97	G	C	0.017 (0.002)	-0.004 (0.02)	0.014 (0.031)	0.002 (0.034)	0.084 (0.088)	-0.123 (0.057)	BMI
rs1218822	13	28011963	0,98	A	G	0.016 (0.002)	-0.002 (0.019)	-0.004 (0.029)	0.04 (0.031)	0.05 (0.081)	-0.144 (0.054)	BMI
rs1933437	13	28624294	1,00	G	A	0.015 (0.002)	0.026 (0.018)	-0.002 (0.027)	0.057 (0.03)	0.031 (0.079)	0.041 (0.053)	BMI
rs12856169	13	31017268	0,98	G	A	0.014 (0.002)	0.019 (0.021)	0.034 (0.031)	-0.005 (0.034)	0.122 (0.092)	-0.011 (0.058)	BMI
rs2239748	13	33101817	1,00	C	T	0.016 (0.002)	0.039 (0.019)	0.039 (0.028)	0.05 (0.031)	0.107 (0.08)	-0.029 (0.054)	BMI
rs9603697	13	40783323	1,00	T	C	0.015 (0.002)	0.023 (0.019)	0.044 (0.029)	0.007 (0.032)	0.057 (0.084)	-0.019 (0.054)	BMI
rs12429545	13	54102206	1,00	A	G	0.03 (0.003)	0.049 (0.027)	0.062 (0.041)	0.055 (0.043)	-0.107 (0.116)	0.054 (0.076)	BMI
rs7989022	13	54104672	1,00	G	A	0.013 (0.002)	-0.009 (0.018)	-0.012 (0.027)	0.002 (0.03)	-0.068 (0.078)	-0.003 (0.052)	BMI
rs12431244	13	58630651	1,00	G	T	0.023 (0.002)	-0.039 (0.021)	-0.025 (0.031)	-0.08 (0.035)	-0.032 (0.093)	0.027 (0.062)	BMI
rs4886100	13	59261225	1,00	A	G	0.015 (0.002)	-0.005 (0.018)	0.007 (0.027)	-0.033 (0.03)	0.006 (0.079)	0.032 (0.051)	BMI
rs9538162	13	59265043	1,00	T	C	0.016 (0.002)	-0.009 (0.018)	-0.001 (0.027)	-0.035 (0.03)	0.018 (0.079)	0.026 (0.051)	BMI
rs9538190	13	59340640	0,99	C	G	0.019 (0.003)	-0.009 (0.024)	0.03 (0.037)	-0.054 (0.04)	0.048 (0.1)	-0.032 (0.067)	BMI

rs2322622	13	60497331	0,99	C	T	0.011 (0.002)	-0.003 (0.019)	0.026 (0.028)	-0.044 (0.031)	-0.044 (0.081)	0.034 (0.053)	BMI
rs1304070	13	65479449	1,00	A	G	0.014 (0.002)	0.004 (0.021)	-0.006 (0.031)	-0.018 (0.035)	0.14 (0.092)	0.04 (0.06)	BMI
rs9540493	13	66205704	0,99	A	G	0.015 (0.002)	0.034 (0.018)	0.04 (0.027)	0.041 (0.03)	0.059 (0.078)	-0.018 (0.052)	BMI
rs9571687	13	67472713	0,99	C	A	0.015 (0.002)	-0.04 (0.019)	-0.063 (0.029)	-0.034 (0.032)	-0.048 (0.082)	0.032 (0.055)	BMI
rs1441264	13	79580919	1,00	A	G	0.018 (0.002)	-0.01 (0.018)	-0.013 (0.027)	-0.008 (0.03)	-0.11 (0.08)	0.035 (0.052)	BMI
rs61969510	13	86484025	0,99	C	T	0.017 (0.003)	-0.002 (0.02)	-0.019 (0.03)	0.01 (0.034)	0.026 (0.088)	0.009 (0.056)	BMI
rs1927790	13	96922191	1,00	C	T	0.016 (0.002)	0.015 (0.018)	0.015 (0.027)	0.011 (0.03)	-0.004 (0.078)	0.032 (0.052)	BMI
rs9584870	13	99245866	0,96	T	C	0.012 (0.002)	0.014 (0.019)	0.019 (0.029)	-0.005 (0.031)	0.128 (0.084)	0.01 (0.053)	BMI
rs2528787	13	112188236	0,94	C	T	0.018 (0.002)	0.014 (0.019)	-0.007 (0.029)	0.023 (0.032)	-0.062 (0.082)	0.09 (0.054)	BMI
rs9522285	13	112230701	1,00	A	G	0.015 (0.002)	0.024 (0.018)	0.03 (0.027)	0.031 (0.03)	-0.128 (0.078)	0.051 (0.051)	BMI
rs10132280	14	25928179	0,97	C	A	0.022 (0.002)	-0.006 (0.02)	-0.007 (0.03)	-0.006 (0.032)	-0.007 (0.086)	0.002 (0.058)	BMI
rs8010984	14	25946443	1,00	T	C	0.02 (0.002)	-0.008 (0.019)	-0.018 (0.029)	-0.011 (0.032)	-0.004 (0.083)	0.031 (0.055)	BMI
rs4981693	14	29680331	0,99	A	G	0.019 (0.002)	0.037 (0.021)	0.07 (0.032)	0.02 (0.035)	0.091 (0.093)	-0.065 (0.062)	BMI
rs12885454	14	29736838	0,98	C	A	0.017 (0.002)	0.036 (0.019)	0.057 (0.029)	0.048 (0.032)	-0.084 (0.083)	-0.022 (0.054)	BMI
rs10483389	14	30495719	1,00	T	C	0.035 (0.005)	0.057 (0.047)	0.102 (0.071)	-0.07 (0.076)	-0.022 (0.204)	0.31 (0.132)	BMI
rs17522122	14	33302882	0,98	T	G	0.018 (0.002)	0.017 (0.018)	0.025 (0.027)	0.011 (0.03)	0.054 (0.076)	-0.008 (0.052)	BMI
rs2150527	14	40875382	0,99	G	A	0.015 (0.002)	0.013 (0.022)	0.011 (0.032)	0.08 (0.036)	0.083 (0.093)	-0.22 (0.064)	BMI
rs4900714	14	47302219	0,99	T	G	0.014 (0.002)	-0.02 (0.018)	-0.039 (0.027)	0.01 (0.03)	-0.004 (0.078)	-0.048 (0.052)	BMI
rs724623	14	47303577	0,99	A	C	0.015 (0.002)	-0.021 (0.018)	-0.041 (0.027)	0.012 (0.03)	-0.011 (0.078)	-0.051 (0.052)	BMI
rs7160227	14	60139839	0,97	A	G	0.012 (0.002)	0.013 (0.019)	0.039 (0.029)	0.014 (0.032)	-0.098 (0.08)	-0.031 (0.054)	BMI
rs217672	14	62361021	0,99	C	A	0.016 (0.003)	0.014 (0.021)	-0.021 (0.031)	0.042 (0.034)	0.075 (0.088)	0.032 (0.058)	BMI
rs3902951	14	69789755	0,97	G	T	0.014 (0.002)	0.025 (0.021)	-0.017 (0.03)	0.066 (0.035)	0.222 (0.092)	-0.02 (0.061)	BMI
rs1007934	14	73463479	1,00	G	A	0.012 (0.002)	0.043 (0.018)	0.066 (0.028)	0.038 (0.03)	-0.046 (0.078)	0.014 (0.052)	BMI
rs2221298	14	79510587	0,89	T	C	0.012 (0.002)	0.006 (0.019)	0.024 (0.029)	-0.028 (0.032)	-0.054 (0.083)	0.066 (0.055)	BMI
rs7144011	14	79940383	1,00	T	G	0.027 (0.002)	0.026 (0.022)	0.019 (0.032)	0.02 (0.036)	0.094 (0.095)	0.046 (0.062)	BMI
rs9646138	14	88292265	1,00	A	G	0.014 (0.002)	0.007 (0.021)	-0.015 (0.031)	0.056 (0.035)	0 (0.089)	-0.055 (0.061)	BMI
rs1286146	14	91474448	0,99	A	G	0.015 (0.002)	0.013 (0.02)	0.02 (0.031)	0.025 (0.034)	0.055 (0.085)	-0.061 (0.057)	BMI
rs1951455	14	91512339	0,91	C	T	0.016 (0.002)	0.01 (0.021)	0.012 (0.031)	0.025 (0.034)	0.07 (0.086)	-0.062 (0.058)	BMI
rs9989178	14	94008080	0,99	A	G	0.023 (0.002)	0.013 (0.019)	0.031 (0.028)	0.018 (0.031)	-0.059 (0.079)	-0.036 (0.054)	BMI
rs3850422	14	99671788	1,00	G	A	0.012 (0.002)	0.003 (0.018)	-0.005 (0.027)	-0.002 (0.03)	-0.002 (0.077)	0.05 (0.052)	BMI
rs12147845	14	101144596	0,96	T	C	0.019 (0.003)	0.005 (0.03)	0.04 (0.046)	0.017 (0.047)	-0.115 (0.12)	-0.088 (0.083)	BMI
rs7161194	14	101529005	0,70	A	G	0.018 (0.002)	0.001 (0.023)	NA (NA)	0.009 (0.037)	NA (NA)	0.018 (0.065)	BMI
rs7143963	14	103304425	1,00	T	C	0.022 (0.002)	-0.005 (0.024)	-0.083 (0.035)	0.08 (0.039)	-0.009 (0.1)	0.024 (0.068)	BMI
rs2010281	14	103862322	1,00	G	A	0.017 (0.002)	0.013 (0.019)	-0.042 (0.028)	0.064 (0.031)	0.028 (0.079)	0.067 (0.054)	BMI
rs709400	14	104149475	1,00	A	G	0.016 (0.002)	0.005 (0.019)	-0.04 (0.028)	0.033 (0.031)	0.14 (0.078)	0.026 (0.053)	BMI
rs7172627	15	31877690	1,00	G	A	0.013 (0.002)	-0.008 (0.018)	-0.014 (0.027)	-0.001 (0.03)	0.011 (0.079)	-0.018 (0.052)	BMI
rs9944219	15	46500612	1,00	G	A	0.012 (0.002)	-0.019 (0.018)	-0.034 (0.027)	-0.018 (0.03)	0.103 (0.078)	-0.022 (0.052)	BMI
rs1559677	15	47738063	1,00	G	A	0.011 (0.002)	0 (0.018)	-0.001 (0.028)	-0.019 (0.03)	0.023 (0.082)	0.054 (0.052)	BMI
rs6493498	15	51754451	0,99	T	C	0.014 (0.002)	0.011 (0.018)	-0.009 (0.028)	0.041 (0.03)	0.087 (0.077)	-0.044 (0.052)	BMI
rs16965225	15	53143170	0,98	T	G	0.024 (0.004)	-0.019 (0.038)	-0.04 (0.056)	0.023 (0.062)	-0.199 (0.157)	0.008 (0.104)	BMI
rs11070956	15	53442930	1,00	G	A	0.023 (0.004)	0.062 (0.036)	0.03 (0.054)	0.105 (0.058)	0.047 (0.15)	0.05 (0.102)	BMI
rs17236194	15	59002755	0,95	C	T	0.015 (0.003)	-0.013 (0.027)	-0.036 (0.04)	0.034 (0.044)	-0.197 (0.113)	0.019 (0.074)	BMI
rs340025	15	60908307	1,00	C	T	0.011 (0.002)	-0.011 (0.018)	-0.028 (0.027)	0.02 (0.03)	0.009 (0.079)	-0.051 (0.052)	BMI
rs11635675	15	63793238	0,99	T	G	0.012 (0.002)	-0.021 (0.019)	-0.021 (0.028)	-0.021 (0.031)	-0.074 (0.081)	0 (0.053)	BMI
rs17200912	15	66730307	0,99	C	T	0.016 (0.002)	-0.102 (0.021)	-0.107 (0.032)	-0.099 (0.035)	-0.181 (0.093)	-0.059 (0.061)	BMI
rs16951275	15	68077168	1,00	T	C	0.03 (0.002)	0.022 (0.022)	0.021 (0.033)	0.046 (0.036)	-0.056 (0.088)	-0.007 (0.061)	BMI
rs8030477	15	73085815	0,99	T	C	0.018 (0.002)	0.013 (0.019)	0.009 (0.029)	0.031 (0.032)	-0.029 (0.082)	-0.011 (0.055)	BMI
rs7164727	15	73093991	0,99	T	C	0.018 (0.002)	0.012 (0.019)	0.011 (0.029)	0.029 (0.032)	-0.027 (0.082)	-0.015 (0.055)	BMI
rs35364449	15	74278126	0,95	T	C	0.027 (0.004)	-0.014 (0.029)	-0.058 (0.042)	0.016 (0.048)	-0.001 (0.126)	0.052 (0.082)	BMI

rs2290573	15	75129594	0,95	A	G	0.012 (0.002)	0.016 (0.018)	0.03 (0.028)	0.021 (0.03)	0.017 (0.078)	-0.05 (0.053)	BMI
rs7177533	15	77387923	0,98	T	C	0.014 (0.002)	0.012 (0.021)	-0.002 (0.031)	0.004 (0.035)	-0.016 (0.089)	0.1 (0.059)	BMI
rs4886869	15	77799657	0,98	G	A	0.013 (0.002)	0.011 (0.019)	0.019 (0.028)	-0.026 (0.031)	0.09 (0.079)	0.058 (0.053)	BMI
rs11855853	15	78012618	0,97	C	T	0.016 (0.002)	-0.007 (0.021)	-0.006 (0.032)	-0.002 (0.034)	-0.057 (0.09)	-0.007 (0.061)	BMI
rs1443658	15	79386366	0,98	A	G	0.014 (0.002)	0.012 (0.018)	0.016 (0.027)	0.004 (0.03)	-0.022 (0.078)	0.034 (0.053)	BMI
rs12900485	15	81022087	1,00	G	A	0.014 (0.002)	-0.036 (0.02)	-0.05 (0.03)	-0.026 (0.034)	-0.023 (0.087)	-0.017 (0.058)	BMI
rs7181498	15	95271404	1,00	T	C	0.017 (0.002)	-0.009 (0.019)	0.028 (0.027)	-0.066 (0.031)	0.095 (0.081)	-0.023 (0.053)	BMI
rs11866815	16	387867	0,99	C	T	0.018 (0.002)	0.03 (0.021)	0.048 (0.031)	0.001 (0.035)	0.042 (0.088)	0.038 (0.06)	BMI
rs2516739	16	2097158	0,89	G	A	0.016 (0.002)	-0.022 (0.023)	-0.101 (0.035)	0.08 (0.037)	0.051 (0.101)	-0.09 (0.061)	BMI
rs12448257	16	3599655	0,96	A	G	0.016 (0.002)	0.007 (0.023)	-0.002 (0.034)	0.024 (0.037)	0.01 (0.096)	-0.017 (0.064)	BMI
rs879620	16	4015729	0,99	T	C	0.026 (0.002)	0.006 (0.018)	-0.021 (0.027)	0.033 (0.03)	0.149 (0.081)	-0.04 (0.054)	BMI
rs1876359	16	4930100	1,00	T	C	0.012 (0.002)	-0.023 (0.019)	0.009 (0.028)	-0.036 (0.031)	0.052 (0.081)	-0.134 (0.054)	BMI
rs977540	16	9724750	0,99	A	G	0.014 (0.002)	0 (0.021)	-0.015 (0.031)	0.011 (0.036)	0.039 (0.094)	0.006 (0.061)	BMI
rs12446632	16	19935389	1,00	G	A	0.033 (0.003)	0.009 (0.026)	0.014 (0.038)	0.035 (0.043)	-0.115 (0.115)	-0.033 (0.074)	BMI
rs11648621	16	19973008	0,92	A	G	0.019 (0.002)	0.038 (0.023)	0.024 (0.034)	0.066 (0.038)	-0.05 (0.097)	0.045 (0.064)	BMI
rs2107118	16	24310282	0,93	A	G	0.013 (0.002)	0.009 (0.019)	0.002 (0.029)	0.02 (0.03)	-0.045 (0.076)	0.025 (0.051)	BMI
rs7195386	16	24578458	1,00	T	C	0.014 (0.002)	0.008 (0.018)	0.003 (0.026)	0.002 (0.03)	-0.022 (0.076)	0.06 (0.051)	BMI
rs7186893	16	24806420	1,00	G	T	0.016 (0.002)	0.013 (0.02)	-0.01 (0.03)	0.044 (0.034)	0.051 (0.086)	-0.007 (0.058)	BMI
rs205149	16	25645124	0,99	C	T	0.012 (0.002)	0.006 (0.018)	-0.026 (0.028)	0.02 (0.03)	-0.029 (0.079)	0.089 (0.051)	BMI
rs2726036	16	28347140	0,76	C	A	0.027 (0.002)	0.03 (0.021)	NA (NA)	0.01 (0.033)	0.06 (0.084)	0.046 (0.056)	BMI
rs7498665	16	28883241	1,00	G	A	0.028 (0.002)	0.042 (0.018)	0.061 (0.027)	0.014 (0.03)	0.141 (0.078)	0.008 (0.052)	BMI
rs4609871	16	29932064	1,00	T	C	0.024 (0.002)	0.026 (0.018)	0.028 (0.027)	0.022 (0.03)	0.038 (0.078)	0.024 (0.052)	BMI
rs3814883	16	29994922	0,94	T	C	0.025 (0.002)	0.018 (0.018)	0.023 (0.027)	0.015 (0.031)	0.009 (0.081)	0.014 (0.053)	BMI
rs2285459	16	30495412	1,00	T	C	0.012 (0.002)	-0.007 (0.018)	0.018 (0.027)	-0.052 (0.03)	0.069 (0.079)	0 (0.052)	BMI
rs4889606	16	31011183	0,94	A	G	0.023 (0.002)	-0.007 (0.019)	-0.016 (0.03)	0 (0.031)	0.013 (0.083)	-0.007 (0.052)	BMI
rs2080454	16	49062590	1,00	C	A	0.012 (0.002)	0.032 (0.018)	0.064 (0.027)	0.019 (0.03)	0.05 (0.08)	-0.058 (0.053)	BMI
rs11075985	16	53805207	0,98	A	C	0.075 (0.002)	0.026 (0.018)	0.019 (0.028)	0.058 (0.03)	-0.04 (0.077)	-0.014 (0.052)	BMI
rs2058908	16	53806145	0,89	C	T	0.054 (0.002)	0.067 (0.022)	0.044 (0.034)	0.078 (0.035)	0.024 (0.093)	0.126 (0.06)	BMI
rs17817288	16	53807764	0,98	G	A	0.065 (0.002)	0.049 (0.018)	0.016 (0.028)	0.105 (0.03)	0.01 (0.076)	0.008 (0.051)	BMI
rs11642841	16	53845487	0,91	A	C	0.066 (0.002)	0.046 (0.019)	0.028 (0.029)	0.082 (0.03)	0.015 (0.082)	0.004 (0.054)	BMI
rs12444921	16	56503416	0,99	G	T	0.018 (0.003)	0.056 (0.026)	0.033 (0.039)	0.075 (0.044)	0.127 (0.117)	0.062 (0.076)	BMI
rs11075489	16	62803841	0,99	C	T	0.012 (0.002)	-0.001 (0.018)	-0.032 (0.027)	0.028 (0.03)	0.063 (0.079)	0.002 (0.051)	BMI
rs78801969	16	64707204	0,92	C	T	0.02 (0.003)	-0.005 (0.026)	-0.047 (0.039)	0.014 (0.042)	0.001 (0.11)	0.085 (0.075)	BMI
rs2307022	16	68381978	1,00	A	G	0.012 (0.002)	0.044 (0.019)	0.063 (0.028)	0.017 (0.032)	-0.039 (0.084)	0.082 (0.054)	BMI
rs889398	16	69556715	0,99	C	T	0.02 (0.002)	0.023 (0.018)	0.009 (0.028)	0.04 (0.03)	0.178 (0.077)	-0.051 (0.052)	BMI
rs244415	16	69666683	0,99	G	A	0.021 (0.002)	0.023 (0.018)	0.018 (0.027)	0.035 (0.03)	0.167 (0.076)	-0.064 (0.052)	BMI
rs4985407	16	70285901	0,90	G	A	0.016 (0.002)	0.001 (0.02)	0.02 (0.033)	-0.019 (0.03)	0.029 (0.078)	0.006 (0.051)	BMI
rs891124	16	71440756	0,99	T	C	0.012 (0.002)	0.014 (0.02)	-0.004 (0.03)	0.037 (0.034)	-0.03 (0.086)	0.031 (0.057)	BMI
rs929866	16	72038363	0,99	T	C	0.015 (0.002)	0.026 (0.019)	0.01 (0.029)	0.057 (0.032)	0.058 (0.083)	-0.025 (0.055)	BMI
rs811054	16	72251132	0,95	T	C	0.014 (0.002)	0.013 (0.019)	0.016 (0.028)	-0.017 (0.03)	0.033 (0.08)	0.083 (0.053)	BMI
rs756717	16	72996162	0,96	G	A	0.013 (0.002)	-0.031 (0.019)	-0.031 (0.028)	-0.045 (0.031)	0.113 (0.084)	-0.049 (0.052)	BMI
rs4888536	16	76761728	0,99	C	A	0.015 (0.002)	0.042 (0.023)	0.09 (0.033)	0.008 (0.038)	0.002 (0.096)	-0.024 (0.064)	BMI
rs2012502	16	81728081	0,98	A	C	0.013 (0.002)	0.004 (0.019)	-0.038 (0.028)	0.028 (0.031)	0.203 (0.082)	0.01 (0.053)	BMI
rs7206608	16	82872628	0,99	G	C	0.013 (0.002)	0.008 (0.02)	-0.007 (0.03)	0.018 (0.032)	0.106 (0.084)	-0.01 (0.055)	BMI
rs3923783	17	1843189	0,97	C	A	0.024 (0.003)	0.015 (0.024)	-0.028 (0.036)	0.058 (0.039)	0.032 (0.099)	0.024 (0.066)	BMI
rs7217226	17	2136065	0,98	G	T	0.013 (0.002)	0.006 (0.019)	0.029 (0.029)	-0.042 (0.031)	-0.03 (0.081)	0.088 (0.054)	BMI
rs3966782	17	4807098	0,98	C	T	0.015 (0.002)	-0.005 (0.019)	0.042 (0.029)	-0.045 (0.031)	-0.077 (0.082)	-0.022 (0.055)	BMI
rs1000940	17	5283252	1,00	G	A	0.016 (0.002)	0.016 (0.019)	0.034 (0.029)	0.002 (0.032)	0.081 (0.085)	-0.043 (0.055)	BMI
rs178810	17	16097430	0,99	T	C	0.012 (0.002)	-0.003 (0.018)	-0.003 (0.027)	0.004 (0.03)	-0.059 (0.078)	0.002 (0.052)	BMI

rs4986044	17	21261560	0,99	C	T	0.018 (0.002)	-0.049 (0.018)	-0.055 (0.027)	-0.056 (0.03)	-0.114 (0.079)	0.02 (0.051)	BMI
rs1038088	17	28074563	0,98	G	T	0.012 (0.002)	-0.034 (0.018)	-0.059 (0.027)	-0.053 (0.03)	0.038 (0.078)	0.081 (0.052)	BMI
rs8066490	17	31460281	0,98	G	A	0.014 (0.002)	0.039 (0.021)	0.037 (0.031)	0.055 (0.035)	-0.039 (0.09)	0.028 (0.062)	BMI
rs9898582	17	32221181	0,99	G	T	0.014 (0.002)	0.001 (0.018)	0.016 (0.027)	-0.002 (0.03)	-0.046 (0.08)	-0.029 (0.052)	BMI
rs12150665	17	34914787	0,99	T	C	0.019 (0.002)	0.034 (0.018)	0.012 (0.027)	0.097 (0.03)	-0.038 (0.079)	-0.038 (0.052)	BMI
rs7218387	17	35055251	0,99	A	G	0.013 (0.002)	-0.009 (0.02)	-0.011 (0.029)	0.024 (0.033)	-0.06 (0.085)	-0.071 (0.056)	BMI
rs2670854	17	41085683	0,91	A	G	0.012 (0.002)	0.022 (0.022)	-0.007 (0.034)	0.056 (0.034)	0.05 (0.088)	-0.01 (0.058)	BMI
rs16969990	17	46230891	0,92	C	T	0.027 (0.004)	-0.012 (0.036)	-0.069 (0.052)	0.051 (0.062)	0.062 (0.168)	0.008 (0.106)	BMI
rs208015	17	46252346	0,99	T	C	0.034 (0.004)	0.004 (0.033)	-0.036 (0.048)	0.054 (0.055)	0.057 (0.148)	-0.017 (0.102)	BMI
rs11079849	17	47090785	0,96	C	T	0.018 (0.002)	-0.006 (0.02)	-0.009 (0.03)	-0.008 (0.032)	0.093 (0.083)	-0.037 (0.056)	BMI
rs11655587	17	47140794	0,98	C	T	0.02 (0.002)	0.004 (0.019)	0.012 (0.029)	-0.001 (0.031)	0.017 (0.082)	-0.011 (0.053)	BMI
rs8075273	17	61728881	1,00	C	A	0.012 (0.002)	-0.007 (0.02)	-0.039 (0.03)	0.025 (0.033)	-0.11 (0.087)	0.057 (0.057)	BMI
rs12602912	17	65870073	1,00	T	C	0.019 (0.002)	-0.004 (0.023)	-0.001 (0.034)	0.004 (0.037)	-0.059 (0.098)	-0.02 (0.064)	BMI
rs12949279	17	78558411	0,99	T	C	0.019 (0.002)	-0.005 (0.018)	0.036 (0.027)	-0.041 (0.03)	-0.145 (0.077)	0.014 (0.051)	BMI
rs11658335	17	80084821	0,98	C	T	0.012 (0.002)	0.014 (0.018)	0.013 (0.027)	-0.001 (0.03)	0.039 (0.078)	0.054 (0.053)	BMI
rs7226371	18	1850771	0,97	G	A	0.02 (0.003)	0.005 (0.025)	0.005 (0.038)	0.043 (0.041)	0.028 (0.102)	-0.124 (0.071)	BMI
rs12953970	18	13186928	0,98	A	G	0.015 (0.003)	-0.014 (0.023)	-0.031 (0.035)	0.004 (0.038)	-0.027 (0.097)	-0.002 (0.067)	BMI
rs891387	18	21103909	1,00	T	C	0.02 (0.002)	0.017 (0.018)	0.004 (0.027)	0.03 (0.03)	0.14 (0.078)	-0.026 (0.051)	BMI
rs16940823	18	22137319	0,98	C	A	0.018 (0.002)	0.018 (0.023)	0.013 (0.034)	0.051 (0.038)	0.029 (0.1)	-0.062 (0.066)	BMI
rs1941697	18	31251276	1,00	A	G	0.012 (0.002)	0.001 (0.018)	-0.008 (0.027)	0.011 (0.03)	0.022 (0.077)	-0.003 (0.052)	BMI
rs8087875	18	39630347	1,00	G	A	0.021 (0.003)	-0.021 (0.027)	-0.055 (0.039)	0.034 (0.044)	-0.144 (0.116)	0.001 (0.08)	BMI
rs7239883	18	40147671	1,00	G	A	0.012 (0.002)	0.009 (0.018)	-0.003 (0.027)	0.007 (0.03)	0.055 (0.078)	0.04 (0.052)	BMI
rs2052608	18	40759678	1,00	A	G	0.015 (0.002)	-0.032 (0.02)	-0.024 (0.03)	-0.048 (0.034)	-0.066 (0.088)	0.001 (0.058)	BMI
rs555267	18	40992698	1,00	T	G	0.014 (0.002)	-0.036 (0.019)	-0.024 (0.029)	-0.058 (0.032)	-0.029 (0.081)	-0.014 (0.055)	BMI
rs954018	18	42598463	1,00	G	A	0.014 (0.002)	-0.012 (0.02)	0 (0.029)	-0.004 (0.032)	0.004 (0.084)	-0.085 (0.056)	BMI
rs7239114	18	45921214	0,98	A	G	0.013 (0.002)	-0.024 (0.018)	-0.024 (0.027)	-0.046 (0.03)	-0.15 (0.079)	0.096 (0.052)	BMI
rs8092503	18	52479487	1,00	G	A	0.015 (0.002)	-0.044 (0.021)	-0.025 (0.031)	-0.051 (0.035)	-0.12 (0.094)	-0.068 (0.06)	BMI
rs784257	18	53397199	0,98	C	T	0.015 (0.002)	-0.006 (0.024)	0.001 (0.036)	-0.013 (0.039)	-0.063 (0.104)	0.014 (0.066)	BMI
rs7243357	18	56883319	0,99	T	G	0.02 (0.002)	0.008 (0.024)	-0.042 (0.036)	0.057 (0.038)	0.048 (0.104)	0.021 (0.067)	BMI
rs1573399	18	57720337	1,00	A	G	0.017 (0.002)	-0.006 (0.018)	-0.006 (0.027)	0.009 (0.03)	-0.11 (0.08)	-0.01 (0.053)	BMI
rs6567160	18	57829135	0,99	C	T	0.055 (0.002)	0.045 (0.021)	0.064 (0.032)	0.008 (0.035)	0.097 (0.093)	0.062 (0.06)	BMI
rs1598786	18	57990470	0,98	T	G	0.032 (0.002)	-0.024 (0.02)	-0.016 (0.03)	-0.048 (0.033)	0.121 (0.086)	-0.042 (0.057)	BMI
rs113728099	18	58157767	0,98	G	A	0.067 (0.007)	0.062 (0.057)	0.055 (0.085)	0.18 (0.093)	-0.008 (0.243)	-0.251 (0.166)	BMI
rs193239997	18	58639547	0,94	T	C	0.072 (0.009)	0.126 (0.07)	0.089 (0.112)	0.179 (0.108)	0.292 (0.296)	-0.002 (0.19)	BMI
rs12454712	18	60845884	0,88	C	T	0.015 (0.002)	-0.012 (0.02)	-0.019 (0.032)	-0.016 (0.031)	-0.027 (0.08)	0.027 (0.052)	BMI
rs7235205	18	63373762	1,00	G	A	0.013 (0.002)	0.035 (0.02)	0.036 (0.029)	0.058 (0.033)	-0.079 (0.085)	0.016 (0.059)	BMI
rs11150911	18	73498528	1,00	A	C	0.013 (0.002)	0.002 (0.02)	-0.03 (0.029)	0.016 (0.033)	0.103 (0.087)	0.039 (0.058)	BMI
rs11668832	19	1812682	0,96	G	A	0.014 (0.002)	-0.017 (0.019)	-0.018 (0.028)	-0.015 (0.03)	0.04 (0.078)	-0.04 (0.052)	BMI
rs12981256	19	1865901	0,92	A	G	0.015 (0.002)	-0.022 (0.019)	-0.026 (0.029)	-0.013 (0.03)	0.079 (0.079)	-0.084 (0.052)	BMI
rs4807179	19	1956035	0,95	A	G	0.016 (0.002)	-0.01 (0.019)	0.003 (0.029)	-0.016 (0.031)	0.065 (0.081)	-0.07 (0.054)	BMI
rs350832	19	4069426	0,98	A	G	0.02 (0.002)	0.044 (0.022)	0.048 (0.033)	0.02 (0.036)	0.19 (0.092)	0.032 (0.063)	BMI
rs273504	19	18215247	0,98	G	A	0.014 (0.002)	-0.001 (0.018)	-0.027 (0.028)	0.019 (0.03)	-0.055 (0.08)	0.051 (0.051)	BMI
rs17724992	19	18454825	0,99	A	G	0.018 (0.002)	0.032 (0.021)	0.023 (0.031)	0.019 (0.034)	0.18 (0.086)	0.033 (0.058)	BMI
rs757318	19	18820308	1,00	C	A	0.018 (0.002)	0.018 (0.018)	0.032 (0.027)	-0.006 (0.03)	0.03 (0.077)	0.03 (0.051)	BMI
rs6511027	19	19391851	0,99	C	T	0.017 (0.002)	-0.006 (0.025)	0.011 (0.037)	0.021 (0.041)	-0.084 (0.105)	-0.107 (0.069)	BMI
rs17513613	19	30286822	0,99	C	T	0.018 (0.002)	0.026 (0.019)	0.054 (0.029)	-0.003 (0.031)	-0.093 (0.084)	0.061 (0.055)	BMI
rs11084553	19	31019780	0,99	A	G	0.018 (0.003)	0.021 (0.026)	0.014 (0.041)	0.022 (0.043)	0.072 (0.111)	0.017 (0.071)	BMI
rs29944	19	34306898	0,99	G	A	0.016 (0.002)	-0.018 (0.019)	-0.041 (0.029)	-0.042 (0.032)	0.215 (0.083)	0.042 (0.055)	BMI
rs29941	19	34309532	1,00	G	A	0.016 (0.002)	-0.02 (0.019)	-0.042 (0.028)	-0.044 (0.032)	0.201 (0.083)	0.038 (0.055)	BMI

rs2075650	19	45395619	1,00	A	G	0.021 (0.003)	0.032 (0.025)	0.049 (0.037)	0.021 (0.042)	-0.026 (0.108)	0.031 (0.073)	BMI
rs11672660	19	46180184	0,98	C	T	0.035 (0.002)	0.055 (0.022)	0.033 (0.033)	0.081 (0.036)	0.112 (0.096)	0.032 (0.064)	BMI
rs3810291	19	47569003	0,93	A	G	0.028 (0.002)	-0.023 (0.02)	-0.04 (0.031)	0.022 (0.032)	-0.04 (0.087)	-0.094 (0.055)	BMI
rs1884897	20	6612832	1,00	G	A	0.02 (0.002)	0.03 (0.019)	0.022 (0.028)	0.053 (0.031)	-0.025 (0.08)	0.019 (0.053)	BMI
rs8123881	20	15819495	0,98	G	A	0.019 (0.003)	-0.013 (0.027)	-0.028 (0.04)	-0.01 (0.044)	0.087 (0.116)	-0.014 (0.076)	BMI
rs852056	20	17102860	1,00	T	C	0.014 (0.002)	0.004 (0.021)	0.049 (0.031)	-0.062 (0.035)	-0.038 (0.089)	0.056 (0.06)	BMI
rs12625687	20	21411237	1,00	C	A	0.02 (0.003)	0.008 (0.03)	-0.024 (0.045)	0.029 (0.051)	0.047 (0.132)	0.05 (0.085)	BMI
rs6138482	20	25059442	1,00	T	C	0.014 (0.002)	-0.022 (0.023)	-0.025 (0.034)	-0.038 (0.038)	0.021 (0.096)	0.014 (0.063)	BMI
rs4456769	20	25190777	1,00	T	C	0.013 (0.002)	0.002 (0.019)	-0.021 (0.028)	0.032 (0.032)	0.064 (0.081)	-0.032 (0.054)	BMI
rs6050446	20	25195509	0,90	G	A	0.034 (0.005)	0.035 (0.055)	-0.133 (0.087)	0.208 (0.09)	-0.031 (0.215)	0.077 (0.145)	BMI
rs6142096	20	32686658	0,99	A	G	0.013 (0.002)	0.009 (0.018)	0.006 (0.027)	-0.002 (0.03)	0.087 (0.079)	0.017 (0.051)	BMI
rs17122844	20	33452600	0,99	C	T	0.014 (0.002)	0.049 (0.022)	0.063 (0.033)	0.028 (0.036)	0.132 (0.092)	0.027 (0.059)	BMI
rs2143253	20	41987392	0,98	G	A	0.018 (0.003)	-0.017 (0.028)	-0.017 (0.043)	-0.038 (0.045)	-0.043 (0.114)	0.056 (0.078)	BMI
rs2425840	20	44904838	0,99	C	A	0.012 (0.002)	0.002 (0.018)	-0.036 (0.028)	0.024 (0.03)	0.042 (0.077)	0.058 (0.052)	BMI
rs6019482	20	47495560	0,97	C	T	0.019 (0.003)	0.079 (0.025)	0.087 (0.037)	0.076 (0.041)	0.142 (0.107)	0.031 (0.069)	BMI
rs13037630	20	51143319	1,00	C	T	0.025 (0.002)	0.005 (0.024)	0.021 (0.036)	0.015 (0.04)	-0.234 (0.107)	0.016 (0.066)	BMI
rs6010784	20	61540319	0,99	T	C	0.012 (0.002)	0.004 (0.018)	0.039 (0.027)	-0.023 (0.03)	0.027 (0.077)	-0.051 (0.051)	BMI
rs73149487	20	62481351	0,87	G	T	0.032 (0.005)	0.031 (0.048)	0.044 (0.077)	0.072 (0.075)	0.103 (0.204)	-0.121 (0.118)	BMI
rs2836754	21	40291740	0,98	C	T	0.014 (0.002)	-0.004 (0.019)	0 (0.028)	-0.031 (0.031)	-0.045 (0.082)	0.08 (0.053)	BMI
rs2838006	21	42653567	1,00	C	T	0.013 (0.002)	-0.031 (0.019)	-0.031 (0.027)	-0.025 (0.031)	-0.112 (0.08)	-0.008 (0.053)	BMI
rs36221780	21	46439910	0,89	G	A	0.03 (0.004)	0.11 (0.039)	0.057 (0.059)	0.186 (0.063)	-0.13 (0.156)	0.172 (0.106)	BMI
rs427943	21	46570896	0,97	C	A	0.018 (0.002)	0.021 (0.018)	0.013 (0.027)	0.028 (0.03)	-0.002 (0.078)	0.039 (0.052)	BMI
rs395379	22	18212923	0,97	G	A	0.015 (0.003)	0.058 (0.034)	NA (NA)	0.083 (0.041)	-0.147 (0.104)	0.076 (0.071)	BMI
rs12628891	22	38317137	0,99	C	T	0.014 (0.002)	0.039 (0.02)	0.033 (0.029)	0.061 (0.032)	-0.041 (0.085)	0.028 (0.055)	BMI
rs5995843	22	40697377	1,00	A	G	0.017 (0.002)	-0.008 (0.019)	-0.024 (0.028)	0.005 (0.031)	0.045 (0.081)	-0.009 (0.053)	BMI
rs738140	22	41884954	0,99	A	G	0.012 (0.002)	0.016 (0.019)	0.034 (0.028)	0.013 (0.032)	-0.032 (0.081)	-0.02 (0.056)	BMI
rs13053342	22	48871624	0,96	A	G	0.012 (0.002)	-0.004 (0.019)	0.033 (0.028)	-0.061 (0.031)	0.016 (0.081)	0.03 (0.052)	BMI
rs7525092	1	1810090	1,00	C	T	0.011 (0.002)	0.008 (0.02)	0.035 (0.03)	-0.006 (0.034)	-0.14 (0.088)	0.011 (0.058)	Wasit-to-hip ratio
rs1109251	1	2973576	0,98	A	G	0.014 (0.002)	-0.005 (0.025)	-0.022 (0.038)	-0.003 (0.041)	0.082 (0.101)	0.005 (0.073)	Wasit-to-hip ratio
rs11583755	1	6672729	1,00	C	A	0.01 (0.002)	0.015 (0.019)	-0.003 (0.028)	0.044 (0.031)	0.036 (0.079)	-0.016 (0.053)	Wasit-to-hip ratio
rs6688233	1	9335745	0,96	T	C	0.014 (0.002)	0.009 (0.022)	0.019 (0.033)	0.032 (0.036)	-0.021 (0.095)	-0.08 (0.059)	Wasit-to-hip ratio
rs2179072	1	9349150	0,99	C	T	0.012 (0.002)	-0.021 (0.02)	-0.024 (0.029)	-0.016 (0.033)	-0.054 (0.085)	-0.011 (0.059)	Wasit-to-hip ratio
rs12024554	1	19925759	0,99	C	T	0.012 (0.002)	-0.004 (0.021)	-0.02 (0.032)	-0.011 (0.035)	0.108 (0.089)	0.023 (0.061)	Wasit-to-hip ratio
rs213010	1	21639167	1,00	A	G	0.01 (0.002)	0.006 (0.019)	0.035 (0.028)	-0.017 (0.031)	-0.074 (0.083)	-0.002 (0.054)	Wasit-to-hip ratio
rs2235529	1	22450487	0,98	C	T	0.013 (0.002)	-0.043 (0.025)	-0.048 (0.037)	-0.031 (0.041)	-0.191 (0.11)	-0.002 (0.07)	Wasit-to-hip ratio
rs10737472	1	23708919	0,98	C	A	0.017 (0.002)	0.055 (0.026)	0.017 (0.038)	0.104 (0.042)	0.272 (0.11)	-0.051 (0.075)	Wasit-to-hip ratio
rs213637	1	26188625	0,99	T	G	0.01 (0.002)	0.006 (0.018)	-0.005 (0.027)	-0.002 (0.03)	-0.046 (0.077)	0.092 (0.051)	Wasit-to-hip ratio
rs7531656	1	49828663	1,00	A	G	0.013 (0.002)	-0.017 (0.019)	-0.023 (0.028)	-0.001 (0.032)	-0.07 (0.084)	-0.024 (0.054)	Wasit-to-hip ratio
rs7544728	1	50339649	1,00	A	G	0.012 (0.002)	-0.018 (0.019)	-0.026 (0.028)	0 (0.032)	-0.069 (0.084)	-0.019 (0.055)	Wasit-to-hip ratio
rs12140153	1	62579891	0,84	G	T	0.018 (0.003)	-0.018 (0.035)	-0.051 (0.051)	0.058 (0.06)	-0.085 (0.148)	-0.065 (0.098)	Wasit-to-hip ratio
rs11208659	1	65979280	1,00	C	T	0.019 (0.003)	-0.003 (0.031)	0.009 (0.044)	-0.045 (0.052)	0.234 (0.144)	-0.017 (0.097)	Wasit-to-hip ratio
rs2815749	1	72814783	1,00	G	A	0.013 (0.002)	0.015 (0.024)	-0.033 (0.037)	0.082 (0.039)	0.081 (0.101)	-0.043 (0.064)	Wasit-to-hip ratio
rs12726975	1	86359419	1,00	T	C	0.013 (0.002)	0.004 (0.021)	0.002 (0.031)	0.018 (0.034)	0.092 (0.09)	-0.064 (0.06)	Wasit-to-hip ratio
rs6699397	1	91212216	0,99	G	A	0.01 (0.002)	0.033 (0.018)	0.039 (0.027)	0.023 (0.031)	0.103 (0.08)	0.012 (0.053)	Wasit-to-hip ratio
rs10747492	1	98342417	1,00	G	A	0.012 (0.002)	-0.035 (0.021)	-0.031 (0.031)	-0.031 (0.036)	0.01 (0.096)	-0.08 (0.062)	Wasit-to-hip ratio
rs1415361	1	103562263	0,99	T	C	0.012 (0.002)	-0.001 (0.018)	0.002 (0.027)	0.009 (0.03)	0.009 (0.079)	-0.046 (0.052)	Wasit-to-hip ratio
rs3108680	1	107572997	0,99	C	T	0.009 (0.002)	0.01 (0.019)	0.016 (0.028)	0.034 (0.031)	-0.054 (0.083)	-0.052 (0.054)	Wasit-to-hip ratio
rs7550711	1	110082886	0,98	T	C	0.029 (0.005)	0.059 (0.048)	0.032 (0.065)	0.068 (0.085)	0.63 (0.264)	-0.017 (0.163)	Wasit-to-hip ratio
rs12033257	1	112318484	0,95	A	G	0.01 (0.002)	0.029 (0.019)	0.007 (0.029)	0.033 (0.031)	0.131 (0.08)	0.05 (0.054)	Wasit-to-hip ratio

rs3789615	1	114941326	0,96	C	T	0.011 (0.002)	0.005 (0.019)	0.017 (0.029)	0.002 (0.03)	-0.14 (0.079)	0.034 (0.052)	Wasit-to-hip ratio
rs10923724	1	119546842	1,00	T	C	0.02 (0.002)	-0.008 (0.018)	-0.036 (0.027)	0.016 (0.03)	0.11 (0.077)	-0.028 (0.052)	Wasit-to-hip ratio
rs1325933	1	119555759	1,00	T	C	0.018 (0.002)	-0.004 (0.021)	-0.017 (0.032)	-0.019 (0.034)	0.135 (0.086)	0.015 (0.059)	Wasit-to-hip ratio
rs61813324	1	156049877	0,73	T	C	0.015 (0.003)	-0.015 (0.031)	-0.023 (0.045)	0.025 (0.051)	-0.1 (0.124)	-0.06 (0.09)	Wasit-to-hip ratio
rs2104912	1	170308387	1,00	G	T	0.014 (0.002)	-0.018 (0.018)	-0.018 (0.027)	-0.026 (0.03)	-0.008 (0.079)	0.007 (0.054)	Wasit-to-hip ratio
rs4471313	1	170367466	0,99	T	G	0.022 (0.002)	-0.008 (0.02)	-0.003 (0.03)	-0.019 (0.033)	-0.044 (0.087)	0.024 (0.058)	Wasit-to-hip ratio
rs11589142	1	172172467	1,00	C	A	0.01 (0.002)	-0.016 (0.019)	-0.006 (0.028)	-0.01 (0.032)	0.009 (0.083)	-0.083 (0.056)	Wasit-to-hip ratio
rs2001129	1	172333248	0,99	T	G	0.017 (0.002)	0.021 (0.018)	0.053 (0.027)	0.002 (0.03)	0.169 (0.079)	-0.101 (0.052)	Wasit-to-hip ratio
rs28680958	1	173848808	0,99	G	A	0.012 (0.002)	0.02 (0.022)	0 (0.033)	0.058 (0.036)	-0.005 (0.094)	-0.006 (0.062)	Wasit-to-hip ratio
rs543874	1	177889480	1,00	G	A	0.015 (0.002)	0.007 (0.023)	0.058 (0.036)	-0.027 (0.037)	0.058 (0.097)	-0.07 (0.062)	Wasit-to-hip ratio
rs11240358	1	205070573	1,00	A	G	0.01 (0.002)	0.044 (0.018)	0.095 (0.027)	0 (0.031)	-0.07 (0.08)	0.034 (0.052)	Wasit-to-hip ratio
rs2605107	1	219650741	0,99	A	C	0.022 (0.002)	0.005 (0.02)	-0.014 (0.031)	0.031 (0.033)	0.019 (0.085)	-0.01 (0.056)	Wasit-to-hip ratio
rs2791550	1	219655369	1,00	G	T	0.023 (0.002)	0.013 (0.02)	-0.004 (0.03)	0.036 (0.032)	0.079 (0.083)	-0.021 (0.055)	Wasit-to-hip ratio
rs12042959	1	243533273	0,99	A	G	0.014 (0.002)	-0.007 (0.026)	0.03 (0.038)	-0.043 (0.042)	0.081 (0.109)	-0.084 (0.074)	Wasit-to-hip ratio
rs6743060	2	629510	0,99	A	C	0.019 (0.002)	-0.01 (0.024)	-0.038 (0.035)	-0.024 (0.039)	0.148 (0.104)	0.071 (0.068)	Wasit-to-hip ratio
rs12714415	2	651430	0,98	T	C	0.02 (0.002)	-0.011 (0.024)	-0.045 (0.036)	-0.013 (0.04)	0.12 (0.104)	0.061 (0.069)	Wasit-to-hip ratio
rs10495563	2	9662210	0,99	A	G	0.01 (0.002)	0.023 (0.019)	0.002 (0.029)	0.04 (0.032)	-0.072 (0.085)	0.089 (0.055)	Wasit-to-hip ratio
rs711869	2	13073967	0,97	G	A	0.014 (0.002)	0.035 (0.018)	0.044 (0.027)	0.034 (0.03)	-0.048 (0.078)	0.039 (0.052)	Wasit-to-hip ratio
rs934778	2	25389224	1,00	G	A	0.014 (0.002)	-0.014 (0.02)	-0.031 (0.03)	0.008 (0.032)	0.037 (0.085)	-0.048 (0.056)	Wasit-to-hip ratio
rs10084238	2	25458379	0,99	G	A	0.013 (0.002)	0.024 (0.018)	0.021 (0.027)	0.038 (0.03)	0.043 (0.077)	-0.019 (0.053)	Wasit-to-hip ratio
rs1275941	2	26957396	0,99	A	C	0.01 (0.002)	-0.022 (0.018)	-0.046 (0.027)	0.003 (0.03)	-0.081 (0.078)	0.013 (0.052)	Wasit-to-hip ratio
rs13406302	2	37874850	0,98	C	A	0.01 (0.002)	0.025 (0.021)	0.074 (0.031)	0.011 (0.034)	-0.087 (0.086)	-0.063 (0.059)	Wasit-to-hip ratio
rs17326656	2	48962291	0,99	T	G	0.012 (0.002)	0.024 (0.022)	0.048 (0.033)	0.027 (0.036)	-0.066 (0.092)	-0.026 (0.06)	Wasit-to-hip ratio
rs11894830	2	58713430	0,99	C	T	0.013 (0.002)	-0.003 (0.024)	-0.063 (0.035)	0.04 (0.039)	0.003 (0.1)	0.097 (0.069)	Wasit-to-hip ratio
rs17049712	2	58961136	1,00	T	C	0.011 (0.002)	0.006 (0.02)	0.005 (0.03)	0.02 (0.032)	-0.132 (0.086)	0.026 (0.056)	Wasit-to-hip ratio
rs6545714	2	59307725	1,00	G	A	0.012 (0.002)	0.026 (0.018)	-0.008 (0.027)	0.043 (0.03)	0.144 (0.08)	0.053 (0.052)	Wasit-to-hip ratio
rs13028903	2	59951465	0,97	T	C	0.009 (0.002)	-0.022 (0.018)	-0.032 (0.028)	-0.015 (0.03)	0.051 (0.079)	-0.043 (0.053)	Wasit-to-hip ratio
rs13391573	2	66237388	1,00	T	C	0.016 (0.002)	-0.01 (0.024)	0.006 (0.035)	-0.014 (0.039)	-0.018 (0.098)	-0.058 (0.069)	Wasit-to-hip ratio
rs11897119	2	66772000	1,00	C	T	0.011 (0.002)	0.028 (0.018)	0.022 (0.027)	0.038 (0.03)	0.117 (0.079)	-0.02 (0.052)	Wasit-to-hip ratio
rs13413974	2	67762906	0,99	T	G	0.018 (0.002)	-0.022 (0.024)	-0.012 (0.036)	-0.029 (0.041)	-0.062 (0.103)	-0.023 (0.07)	Wasit-to-hip ratio
rs2861643	2	67797924	0,99	C	T	0.016 (0.002)	0.013 (0.02)	-0.009 (0.029)	0.034 (0.032)	-0.104 (0.087)	0.076 (0.056)	Wasit-to-hip ratio
rs7598674	2	67852689	1,00	G	A	0.016 (0.002)	0.014 (0.019)	-0.012 (0.028)	0.035 (0.031)	0.019 (0.083)	0.043 (0.054)	Wasit-to-hip ratio
rs6542924	2	100893113	0,96	C	A	0.012 (0.002)	-0.036 (0.02)	-0.066 (0.03)	0.003 (0.033)	0.001 (0.084)	-0.058 (0.057)	Wasit-to-hip ratio
rs4851286	2	100915629	1,00	T	C	0.012 (0.002)	-0.034 (0.019)	-0.043 (0.028)	-0.023 (0.031)	0.034 (0.079)	-0.061 (0.054)	Wasit-to-hip ratio
rs4849294	2	114619997	1,00	T	C	0.011 (0.002)	0.014 (0.019)	-0.007 (0.028)	0.035 (0.031)	0.03 (0.082)	0.022 (0.052)	Wasit-to-hip ratio
rs332105	2	119444229	0,99	G	A	0.011 (0.002)	-0.001 (0.018)	-0.01 (0.027)	-0.002 (0.03)	-0.086 (0.079)	0.075 (0.052)	Wasit-to-hip ratio
rs1020731	2	161144055	0,99	A	G	0.012 (0.002)	0.011 (0.02)	0.032 (0.03)	-0.008 (0.032)	-0.093 (0.085)	0.041 (0.056)	Wasit-to-hip ratio
rs10195252	2	165513091	0,99	T	C	0.019 (0.002)	0.04 (0.018)	0.011 (0.027)	0.05 (0.03)	0.167 (0.079)	0.06 (0.053)	Wasit-to-hip ratio
rs355838	2	165619163	0,99	T	G	0.014 (0.002)	0.011 (0.018)	0.023 (0.027)	-0.003 (0.03)	0.147 (0.08)	-0.05 (0.053)	Wasit-to-hip ratio
rs10184230	2	171602134	0,99	C	T	0.011 (0.002)	-0.03 (0.019)	-0.01 (0.029)	-0.056 (0.032)	-0.046 (0.082)	-0.019 (0.054)	Wasit-to-hip ratio
rs7588437	2	181575281	0,99	G	A	0.01 (0.002)	0.027 (0.019)	-0.004 (0.028)	0.046 (0.031)	0.157 (0.08)	0.028 (0.053)	Wasit-to-hip ratio
rs1569135	2	188115398	0,99	A	G	0.019 (0.002)	-0.038 (0.018)	-0.032 (0.027)	-0.043 (0.03)	-0.005 (0.079)	-0.064 (0.052)	Wasit-to-hip ratio
rs10498240	2	230734531	1,00	A	C	0.011 (0.002)	0.009 (0.019)	0.025 (0.028)	-0.024 (0.032)	0.093 (0.083)	0.009 (0.055)	Wasit-to-hip ratio
rs4686340	3	9345218	0,96	A	C	0.012 (0.002)	-0.032 (0.021)	-0.008 (0.031)	-0.052 (0.035)	-0.156 (0.088)	-0.005 (0.064)	Wasit-to-hip ratio
rs6442253	3	11474238	0,99	T	C	0.013 (0.002)	0.024 (0.024)	0.052 (0.036)	-0.03 (0.04)	0.159 (0.1)	0.023 (0.066)	Wasit-to-hip ratio
rs796313	3	12449528	1,00	G	T	0.011 (0.002)	-0.001 (0.018)	-0.013 (0.027)	0.001 (0.03)	-0.04 (0.078)	0.055 (0.051)	Wasit-to-hip ratio
rs17819328	3	12489342	0,97	G	T	0.016 (0.002)	-0.017 (0.018)	-0.018 (0.028)	-0.01 (0.03)	-0.015 (0.08)	-0.035 (0.053)	Wasit-to-hip ratio
rs1447649	3	15837784	1,00	C	T	0.011 (0.002)	-0.007 (0.02)	-0.01 (0.029)	0.02 (0.032)	-0.049 (0.082)	-0.056 (0.055)	Wasit-to-hip ratio
rs11129563	3	33900651	1,00	G	A	0.012 (0.002)	-0.025 (0.021)	-0.01 (0.031)	-0.079 (0.035)	0.055 (0.09)	0.047 (0.06)	Wasit-to-hip ratio

rs7612999	3	35678337	0,99	A	G	0.016 (0.002)	0.026 (0.021)	0.043 (0.03)	0.008 (0.035)	-0.049 (0.088)	0.047 (0.059)	Wasit-to-hip ratio
rs3016003	3	37587444	1,00	T	C	0.01 (0.002)	0.013 (0.018)	-0.001 (0.027)	0.018 (0.03)	0.146 (0.079)	-0.01 (0.052)	Wasit-to-hip ratio
rs13092573	3	46988561	0,91	T	C	0.011 (0.002)	-0.019 (0.02)	0.004 (0.031)	-0.049 (0.032)	-0.018 (0.089)	-0.006 (0.054)	Wasit-to-hip ratio
rs13098603	3	47534109	0,76	A	G	0.01 (0.002)	-0.016 (0.022)	0.008 (0.034)	-0.026 (0.034)	-0.083 (0.093)	-0.035 (0.061)	Wasit-to-hip ratio
rs1903061	3	48231919	0,83	G	T	0.019 (0.003)	0 (0.032)	-0.036 (0.05)	0.023 (0.051)	0.142 (0.134)	-0.017 (0.091)	Wasit-to-hip ratio
rs6446204	3	49034879	1,00	C	T	0.011 (0.002)	-0.038 (0.021)	-0.049 (0.031)	-0.035 (0.035)	-0.027 (0.091)	-0.009 (0.06)	Wasit-to-hip ratio
rs2291542	3	49751585	1,00	T	C	0.014 (0.002)	0.01 (0.02)	0.009 (0.029)	0.01 (0.033)	-0.075 (0.084)	0.053 (0.054)	Wasit-to-hip ratio
rs2280406	3	49941436	0,99	A	G	0.013 (0.002)	0.029 (0.018)	0.008 (0.027)	0.022 (0.03)	0.101 (0.078)	0.092 (0.052)	Wasit-to-hip ratio
rs12637870	3	50506073	0,95	C	A	0.014 (0.002)	0.011 (0.024)	0.024 (0.036)	-0.046 (0.038)	0.061 (0.098)	0.116 (0.067)	Wasit-to-hip ratio
rs11235	3	52745087	0,94	C	T	0.012 (0.002)	-0.012 (0.019)	-0.019 (0.029)	0.004 (0.031)	0.055 (0.08)	-0.061 (0.053)	Wasit-to-hip ratio
rs7641212	3	53251807	0,91	C	T	0.011 (0.002)	0 (0.02)	-0.04 (0.03)	0.01 (0.033)	0.162 (0.084)	0.036 (0.056)	Wasit-to-hip ratio
rs9860730	3	64701146	0,99	A	G	0.022 (0.002)	-0.053 (0.02)	0.005 (0.029)	-0.106 (0.032)	-0.073 (0.085)	-0.093 (0.056)	Wasit-to-hip ratio
rs4324463	3	64708628	0,99	C	T	0.023 (0.002)	-0.038 (0.021)	0.02 (0.031)	-0.091 (0.034)	-0.068 (0.09)	-0.085 (0.061)	Wasit-to-hip ratio
rs1007414	3	82727509	1,00	T	C	0.011 (0.002)	0.003 (0.019)	0.013 (0.028)	0.035 (0.031)	-0.02 (0.079)	-0.112 (0.053)	Wasit-to-hip ratio
rs7629375	3	85895494	1,00	C	A	0.011 (0.002)	0.01 (0.019)	0.056 (0.028)	-0.021 (0.031)	0.046 (0.081)	-0.084 (0.054)	Wasit-to-hip ratio
rs9942009	3	89121921	1,00	T	C	0.011 (0.002)	0.023 (0.018)	-0.021 (0.027)	0.08 (0.03)	-0.002 (0.079)	0.022 (0.052)	Wasit-to-hip ratio
rs1609906	3	94033599	1,00	G	A	0.011 (0.002)	0.036 (0.018)	0.077 (0.027)	0.016 (0.03)	0.014 (0.079)	-0.048 (0.052)	Wasit-to-hip ratio
rs9861425	3	123072883	0,99	C	A	0.009 (0.002)	0.014 (0.018)	0.005 (0.027)	0.018 (0.03)	0.073 (0.08)	0.007 (0.051)	Wasit-to-hip ratio
rs11718692	3	128195814	1,00	G	T	0.012 (0.002)	-0.025 (0.023)	-0.016 (0.035)	-0.024 (0.038)	-0.098 (0.098)	-0.027 (0.064)	Wasit-to-hip ratio
rs7610060	3	129300291	1,00	C	A	0.012 (0.002)	0.01 (0.019)	0.038 (0.029)	0.008 (0.032)	-0.124 (0.082)	-0.024 (0.055)	Wasit-to-hip ratio
rs9837325	3	129315831	1,00	C	A	0.022 (0.002)	-0.006 (0.023)	0.029 (0.035)	-0.015 (0.038)	-0.223 (0.098)	-0.013 (0.065)	Wasit-to-hip ratio
rs10804591	3	129334233	1,00	A	C	0.02 (0.002)	0 (0.022)	0.031 (0.033)	0 (0.036)	-0.227 (0.092)	-0.003 (0.063)	Wasit-to-hip ratio
rs6795831	3	129341403	0,97	A	C	0.022 (0.002)	-0.001 (0.024)	0.038 (0.035)	-0.016 (0.038)	-0.156 (0.099)	-0.021 (0.066)	Wasit-to-hip ratio
rs13063979	3	131564741	1,00	G	T	0.011 (0.002)	0.036 (0.02)	0.004 (0.031)	0.08 (0.034)	0.17 (0.087)	-0.034 (0.057)	Wasit-to-hip ratio
rs645040	3	135926622	1,00	T	G	0.011 (0.002)	0.014 (0.022)	-0.011 (0.034)	0.07 (0.036)	0.062 (0.092)	-0.084 (0.061)	Wasit-to-hip ratio
rs13075615	3	136505832	0,97	C	T	0.013 (0.002)	0.015 (0.026)	-0.028 (0.039)	0.07 (0.043)	0.142 (0.109)	-0.056 (0.075)	Wasit-to-hip ratio
rs9844972	3	150097635	0,85	C	G	0.021 (0.003)	0.038 (0.037)	0.034 (0.059)	0.032 (0.059)	0.07 (0.149)	0.05 (0.1)	Wasit-to-hip ratio
rs9820091	3	156267397	1,00	T	C	0.013 (0.002)	0.001 (0.024)	0.008 (0.037)	-0.001 (0.04)	-0.024 (0.106)	-0.008 (0.069)	Wasit-to-hip ratio
rs17451107	3	156797609	0,97	T	C	0.022 (0.002)	-0.001 (0.019)	-0.001 (0.027)	0.005 (0.031)	0.003 (0.081)	-0.018 (0.056)	Wasit-to-hip ratio
rs998749	3	168972802	1,00	A	G	0.01 (0.002)	0.022 (0.018)	0.045 (0.027)	0.022 (0.03)	-0.148 (0.076)	0.017 (0.051)	Wasit-to-hip ratio
rs4894803	3	171800256	1,00	A	G	0.011 (0.002)	0.04 (0.018)	0.05 (0.027)	0.033 (0.03)	0.012 (0.079)	0.033 (0.052)	Wasit-to-hip ratio
rs291927	3	173728170	0,99	C	A	0.011 (0.002)	0.031 (0.021)	0.023 (0.031)	0.03 (0.034)	0.111 (0.092)	0.029 (0.059)	Wasit-to-hip ratio
rs6784112	3	184899231	0,99	G	A	0.014 (0.002)	0.022 (0.024)	0.03 (0.036)	0.02 (0.041)	0.069 (0.104)	-0.018 (0.068)	Wasit-to-hip ratio
rs7647305	3	185834290	0,99	C	T	0.011 (0.002)	-0.026 (0.022)	-0.031 (0.033)	-0.057 (0.037)	0.048 (0.094)	0.049 (0.064)	Wasit-to-hip ratio
rs28602597	4	20112947	0,97	A	G	0.016 (0.003)	0.017 (0.027)	0.024 (0.041)	0.067 (0.044)	-0.098 (0.117)	-0.106 (0.077)	Wasit-to-hip ratio
rs2137234	4	26080549	0,98	C	T	0.015 (0.002)	-0.012 (0.023)	-0.039 (0.034)	-0.007 (0.038)	0.075 (0.098)	0.034 (0.065)	Wasit-to-hip ratio
rs17644283	4	26308792	0,98	A	G	0.012 (0.002)	0.013 (0.019)	0 (0.028)	0.041 (0.031)	0.006 (0.08)	-0.023 (0.053)	Wasit-to-hip ratio
rs13130484	4	45175691	0,98	T	C	0.012 (0.002)	0.012 (0.018)	0.041 (0.027)	0.006 (0.03)	-0.142 (0.079)	-0.01 (0.052)	Wasit-to-hip ratio
rs476184	4	56246864	1,00	G	A	0.012 (0.002)	0.013 (0.02)	0.015 (0.029)	0.019 (0.032)	-0.035 (0.084)	0.006 (0.057)	Wasit-to-hip ratio
rs9991328	4	89713121	1,00	T	C	0.015 (0.002)	0.024 (0.018)	0.035 (0.027)	-0.02 (0.03)	0.058 (0.077)	0.103 (0.052)	Wasit-to-hip ratio
rs2213041	4	100247351	1,00	A	C	0.013 (0.002)	-0.012 (0.024)	-0.002 (0.035)	-0.023 (0.04)	0.066 (0.107)	-0.048 (0.068)	Wasit-to-hip ratio
rs797090	4	140867514	0,98	A	G	0.01 (0.002)	0.019 (0.019)	0.027 (0.027)	0.037 (0.031)	-0.068 (0.081)	-0.033 (0.055)	Wasit-to-hip ratio
rs13146972	4	145569692	1,00	C	T	0.01 (0.002)	0.031 (0.018)	0.056 (0.027)	0.054 (0.03)	0 (0.078)	-0.115 (0.052)	Wasit-to-hip ratio
rs7727202	5	4007914	0,98	C	A	0.012 (0.002)	-0.006 (0.018)	0.02 (0.027)	-0.019 (0.03)	-0.143 (0.079)	0.003 (0.052)	Wasit-to-hip ratio
rs2448	5	53302354	1,00	T	C	0.011 (0.002)	-0.011 (0.02)	0.002 (0.031)	-0.006 (0.034)	-0.114 (0.087)	-0.033 (0.058)	Wasit-to-hip ratio
rs30000	5	55803533	0,98	G	A	0.021 (0.002)	0.018 (0.021)	0.039 (0.031)	0.029 (0.034)	-0.091 (0.088)	-0.046 (0.059)	Wasit-to-hip ratio
rs459193	5	55806751	0,98	A	G	0.021 (0.002)	0.02 (0.021)	0.038 (0.031)	0.036 (0.034)	-0.096 (0.089)	-0.048 (0.06)	Wasit-to-hip ratio
rs16885714	5	55841824	0,96	G	A	0.025 (0.003)	0.054 (0.031)	0.043 (0.046)	0.062 (0.051)	-0.001 (0.122)	0.1 (0.089)	Wasit-to-hip ratio
rs2307111	5	75003678	1,00	T	C	0.012 (0.002)	0.022 (0.018)	0.021 (0.027)	0.037 (0.03)	-0.046 (0.079)	0.013 (0.053)	Wasit-to-hip ratio

rs1382894	5	76599022	1,00	A	T	0.01 (0.002)	-0.038 (0.019)	-0.036 (0.028)	-0.039 (0.032)	-0.047 (0.082)	-0.037 (0.054)	Wasit-to-hip ratio
rs6870983	5	87697533	0,99	C	T	0.014 (0.002)	0.004 (0.022)	0.005 (0.032)	0.01 (0.036)	0.064 (0.093)	-0.048 (0.064)	Wasit-to-hip ratio
rs2161097	5	103945178	1,00	T	C	0.009 (0.002)	-0.004 (0.018)	-0.021 (0.027)	0.025 (0.03)	-0.041 (0.076)	-0.01 (0.052)	Wasit-to-hip ratio
rs11956399	5	112888676	0,99	T	G	0.012 (0.002)	0.041 (0.023)	0.05 (0.033)	0.031 (0.038)	0.099 (0.096)	0.009 (0.067)	Wasit-to-hip ratio
rs3813309	5	118690998	0,99	A	G	0.01 (0.002)	0.03 (0.02)	-0.021 (0.03)	0.086 (0.032)	0.063 (0.084)	0.029 (0.056)	Wasit-to-hip ratio
rs11747001	5	132412299	0,99	A	G	0.014 (0.002)	0.055 (0.021)	0.054 (0.032)	0.07 (0.035)	-0.026 (0.092)	0.045 (0.061)	Wasit-to-hip ratio
rs10477191	5	142077715	0,74	G	A	0.028 (0.004)	0.092 (0.048)	NA (NA)	0.062 (0.073)	0.115 (0.203)	0.174 (0.145)	Wasit-to-hip ratio
rs6861681	5	173362458	1,00	A	G	0.014 (0.002)	0.058 (0.019)	0.103 (0.029)	0.035 (0.032)	-0.007 (0.084)	-0.017 (0.056)	Wasit-to-hip ratio
rs244723	5	176534886	0,94	A	G	0.013 (0.002)	-0.02 (0.018)	-0.027 (0.028)	0.028 (0.03)	-0.149 (0.079)	-0.083 (0.052)	Wasit-to-hip ratio
rs1294410	6	6738752	0,99	C	T	0.021 (0.002)	-0.006 (0.019)	0.01 (0.028)	-0.024 (0.031)	0.047 (0.08)	-0.034 (0.052)	Wasit-to-hip ratio
rs1294437	6	6749789	0,98	C	T	0.02 (0.002)	-0.008 (0.019)	0.002 (0.028)	-0.021 (0.032)	0.048 (0.082)	-0.034 (0.053)	Wasit-to-hip ratio
rs11759008	6	7258523	0,96	T	C	0.011 (0.002)	-0.021 (0.019)	-0.053 (0.028)	0.007 (0.031)	0.045 (0.081)	-0.016 (0.053)	Wasit-to-hip ratio
rs9296938	6	14573063	1,00	G	A	0.012 (0.002)	0.012 (0.021)	-0.007 (0.031)	0.051 (0.034)	0.03 (0.092)	-0.042 (0.059)	Wasit-to-hip ratio
rs151302046	6	30775131	0,96	A	G	0.056 (0.008)	0.056 (0.076)	-0.077 (0.116)	0.271 (0.119)	0.39 (0.329)	-0.417 (0.238)	Wasit-to-hip ratio
rs2523582	6	31328092	0,96	G	A	0.019 (0.002)	0.073 (0.022)	0.057 (0.034)	0.079 (0.035)	-0.045 (0.092)	0.141 (0.056)	Wasit-to-hip ratio
rs2844498	6	31476854	1,00	T	C	0.01 (0.002)	0.012 (0.018)	-0.044 (0.027)	0.05 (0.03)	-0.042 (0.079)	0.13 (0.051)	Wasit-to-hip ratio
rs605203	6	31847012	1,00	A	C	0.016 (0.002)	0.056 (0.02)	0.036 (0.03)	0.043 (0.032)	0.03 (0.082)	0.164 (0.053)	Wasit-to-hip ratio
rs17207951	6	32050653	1,00	T	C	0.011 (0.002)	0.025 (0.019)	-0.003 (0.027)	0.017 (0.032)	0.123 (0.085)	0.125 (0.056)	Wasit-to-hip ratio
rs556025	6	32570880	0,86	T	C	0.016 (0.002)	-0.024 (0.021)	NA (NA)	0.004 (0.032)	-0.19 (0.085)	-0.003 (0.053)	Wasit-to-hip ratio
rs2858864	6	32578229	0,79	C	T	0.013 (0.002)	0.042 (0.023)	NA (NA)	0.056 (0.033)	-0.252 (0.091)	0.125 (0.06)	Wasit-to-hip ratio
rs3130100	6	33283766	1,00	C	T	0.01 (0.002)	0.026 (0.018)	0.002 (0.027)	0.028 (0.03)	0.033 (0.08)	0.11 (0.051)	Wasit-to-hip ratio
rs76376137	6	34173330	0,93	G	T	0.05 (0.004)	-0.012 (0.044)	-0.138 (0.068)	0.128 (0.072)	0.069 (0.202)	-0.03 (0.121)	Wasit-to-hip ratio
rs2033529	6	40348653	1,00	G	A	0.015 (0.002)	0.008 (0.02)	0.021 (0.029)	-0.003 (0.033)	-0.049 (0.085)	0.016 (0.056)	Wasit-to-hip ratio
rs3828755	6	43115220	0,99	G	A	0.01 (0.002)	-0.024 (0.018)	-0.076 (0.027)	0.011 (0.03)	-0.041 (0.075)	0.069 (0.052)	Wasit-to-hip ratio
rs998584	6	43757896	0,84	A	C	0.029 (0.002)	0.028 (0.02)	0.051 (0.03)	-0.002 (0.032)	0.007 (0.083)	0.054 (0.055)	Wasit-to-hip ratio
rs881858	6	43806609	0,95	A	G	0.014 (0.002)	0.002 (0.02)	0.01 (0.03)	-0.003 (0.033)	0.079 (0.088)	-0.05 (0.057)	Wasit-to-hip ratio
rs9463594	6	50318587	1,00	G	A	0.02 (0.003)	0.04 (0.037)	0.07 (0.052)	0.043 (0.065)	-0.181 (0.162)	-0.004 (0.109)	Wasit-to-hip ratio
rs4715208	6	50829471	1,00	G	A	0.012 (0.002)	-0.003 (0.021)	-0.01 (0.031)	0.029 (0.034)	0.053 (0.092)	-0.095 (0.059)	Wasit-to-hip ratio
rs2207139	6	50845490	1,00	G	A	0.016 (0.002)	0.047 (0.023)	0.045 (0.033)	0.09 (0.039)	-0.126 (0.101)	-0.007 (0.068)	Wasit-to-hip ratio
rs16891545	6	80927751	1,00	T	G	0.021 (0.003)	0.063 (0.031)	0.057 (0.045)	0.087 (0.054)	0.074 (0.135)	0.008 (0.096)	Wasit-to-hip ratio
rs1339382	6	81436013	1,00	A	G	0.01 (0.002)	0.035 (0.018)	0.043 (0.027)	0.073 (0.03)	0.003 (0.079)	-0.096 (0.052)	Wasit-to-hip ratio
rs1322863	6	85720325	1,00	A	G	0.019 (0.003)	0.138 (0.034)	0.095 (0.048)	0.159 (0.058)	0.256 (0.159)	0.216 (0.101)	Wasit-to-hip ratio
rs10499013	6	97946396	0,94	G	A	0.011 (0.002)	0.005 (0.021)	0.015 (0.031)	-0.005 (0.034)	0.035 (0.086)	-0.014 (0.059)	Wasit-to-hip ratio
rs901630	6	98539519	0,97	C	T	0.01 (0.002)	0.01 (0.019)	0.007 (0.029)	-0.006 (0.03)	-0.034 (0.08)	0.087 (0.053)	Wasit-to-hip ratio
rs2503099	6	100610101	1,00	G	A	0.019 (0.002)	-0.022 (0.024)	-0.04 (0.036)	0.017 (0.04)	0.086 (0.104)	-0.114 (0.067)	Wasit-to-hip ratio
rs768023	6	108876002	0,99	A	G	0.011 (0.002)	0.007 (0.018)	-0.002 (0.027)	0.011 (0.031)	0.113 (0.08)	-0.021 (0.052)	Wasit-to-hip ratio
rs2184968	6	126760994	0,99	T	C	0.011 (0.002)	0.004 (0.018)	-0.009 (0.027)	0.007 (0.03)	0.01 (0.079)	0.044 (0.051)	Wasit-to-hip ratio
rs143474978	6	126814933	0,87	C	T	0.054 (0.005)	-0.063 (0.056)	-0.007 (0.089)	-0.17 (0.088)	-0.174 (0.228)	0.125 (0.145)	Wasit-to-hip ratio
rs987763	6	127281547	0,95	C	T	0.016 (0.002)	0.019 (0.019)	0.029 (0.028)	0.02 (0.031)	-0.022 (0.081)	0.001 (0.052)	Wasit-to-hip ratio
rs9375486	6	127388186	1,00	C	T	0.019 (0.002)	0.004 (0.018)	-0.014 (0.027)	0.016 (0.031)	-0.038 (0.079)	0.061 (0.053)	Wasit-to-hip ratio
rs72961007	6	127524398	0,73	A	C	0.068 (0.004)	0.01 (0.046)	NA (NA)	-0.055 (0.072)	-0.086 (0.179)	0.189 (0.122)	Wasit-to-hip ratio
rs4534007	6	129373689	0,99	A	G	0.009 (0.002)	-0.025 (0.018)	-0.052 (0.027)	0.016 (0.03)	-0.02 (0.078)	-0.053 (0.052)	Wasit-to-hip ratio
rs605066	6	139829666	0,98	C	T	0.016 (0.002)	-0.031 (0.018)	0.008 (0.028)	-0.062 (0.03)	-0.054 (0.079)	-0.072 (0.053)	Wasit-to-hip ratio
rs639286	6	153459796	0,99	T	G	0.01 (0.002)	0.036 (0.018)	-0.013 (0.027)	0.085 (0.03)	-0.005 (0.078)	0.082 (0.052)	Wasit-to-hip ratio
rs668871	6	160769811	0,99	C	T	0.01 (0.002)	-0.027 (0.018)	-0.039 (0.027)	0.01 (0.03)	-0.065 (0.077)	-0.076 (0.052)	Wasit-to-hip ratio
rs1055144	7	25871109	1,00	T	C	0.02 (0.002)	-0.001 (0.023)	0.002 (0.034)	-0.018 (0.038)	0.087 (0.099)	0.002 (0.065)	Wasit-to-hip ratio
rs1406754	7	26396198	0,98	G	T	0.014 (0.002)	-0.007 (0.019)	0 (0.028)	-0.034 (0.03)	0.116 (0.08)	-0.004 (0.052)	Wasit-to-hip ratio
rs1534696	7	26397239	0,92	C	A	0.017 (0.002)	0.006 (0.019)	0.02 (0.028)	-0.023 (0.031)	0.088 (0.082)	0.006 (0.053)	Wasit-to-hip ratio
rs7801581	7	27223771	0,97	T	C	0.013 (0.002)	-0.01 (0.021)	-0.02 (0.032)	0.014 (0.035)	-0.039 (0.093)	-0.032 (0.059)	Wasit-to-hip ratio

rs2189239	7	27237453	1,00	C	T	0.02 (0.003)	0.009 (0.032)	0.069 (0.047)	-0.066 (0.053)	0.149 (0.138)	-0.058 (0.092)	Wasit-to-hip ratio
rs1178979	7	72856430	0,95	T	C	0.011 (0.002)	0.055 (0.024)	0.105 (0.039)	0.032 (0.038)	0.099 (0.098)	-0.037 (0.065)	Wasit-to-hip ratio
rs17145717	7	72929608	0,84	A	G	0.019 (0.003)	-0.099 (0.041)	NA (NA)	-0.093 (0.064)	0.012 (0.182)	-0.047 (0.102)	Wasit-to-hip ratio
rs3843540	7	99126640	1,00	T	C	0.014 (0.002)	0.045 (0.025)	0.083 (0.037)	0.02 (0.042)	-0.209 (0.118)	0.07 (0.073)	Wasit-to-hip ratio
rs411844	7	101692403	0,95	G	A	0.014 (0.002)	0.072 (0.024)	0.048 (0.035)	0.097 (0.039)	0.237 (0.099)	0.015 (0.068)	Wasit-to-hip ratio
rs1142	7	104756326	0,99	T	C	0.011 (0.002)	-0.005 (0.019)	-0.058 (0.029)	0.053 (0.031)	0.151 (0.082)	-0.056 (0.054)	Wasit-to-hip ratio
rs4727695	7	107614003	1,00	A	G	0.02 (0.003)	0.005 (0.031)	0.098 (0.047)	-0.046 (0.051)	-0.27 (0.129)	-0.046 (0.089)	Wasit-to-hip ratio
rs6966838	7	117083580	0,99	C	T	0.012 (0.002)	0.018 (0.018)	0.003 (0.027)	0.041 (0.03)	-0.068 (0.08)	0.04 (0.054)	Wasit-to-hip ratio
rs10500083	7	120883602	0,99	T	C	0.01 (0.002)	-0.029 (0.019)	-0.001 (0.028)	-0.068 (0.031)	0.024 (0.08)	-0.04 (0.053)	Wasit-to-hip ratio
rs9969455	8	12617155	0,92	A	G	0.01 (0.002)	0 (0.019)	-0.049 (0.029)	0.035 (0.032)	0.121 (0.084)	0.015 (0.053)	Wasit-to-hip ratio
rs6983481	8	23609009	1,00	G	T	0.014 (0.002)	-0.031 (0.021)	-0.032 (0.032)	-0.058 (0.035)	-0.047 (0.09)	0.06 (0.06)	Wasit-to-hip ratio
rs4872376	8	25759022	0,99	T	C	0.012 (0.002)	-0.023 (0.018)	-0.018 (0.027)	-0.024 (0.03)	-0.083 (0.078)	-0.015 (0.051)	Wasit-to-hip ratio
rs2725371	8	30854033	1,00	A	G	0.014 (0.002)	0.013 (0.02)	0.002 (0.029)	-0.003 (0.033)	0.186 (0.085)	0.025 (0.056)	Wasit-to-hip ratio
rs881301	8	38332318	0,99	C	T	0.011 (0.002)	0.022 (0.018)	0.056 (0.027)	0.031 (0.03)	-0.054 (0.078)	-0.102 (0.052)	Wasit-to-hip ratio
rs7825457	8	68271955	0,97	G	A	0.011 (0.002)	-0.015 (0.02)	-0.01 (0.03)	0.013 (0.034)	-0.235 (0.09)	-0.022 (0.058)	Wasit-to-hip ratio
rs10504486	8	71843581	1,00	A	G	0.018 (0.003)	0.024 (0.032)	0.012 (0.048)	0.018 (0.052)	-0.057 (0.137)	0.127 (0.092)	Wasit-to-hip ratio
rs10096191	8	72419498	0,99	G	T	0.025 (0.003)	0.052 (0.037)	0.024 (0.057)	0.105 (0.06)	-0.208 (0.156)	0.092 (0.096)	Wasit-to-hip ratio
rs4738141	8	72469742	0,99	G	A	0.016 (0.002)	0.035 (0.021)	0.022 (0.031)	0.056 (0.034)	-0.067 (0.09)	0.064 (0.059)	Wasit-to-hip ratio
rs1431659	8	73439070	1,00	A	G	0.011 (0.002)	-0.006 (0.02)	-0.015 (0.03)	0.006 (0.034)	-0.102 (0.088)	0.031 (0.057)	Wasit-to-hip ratio
rs13256367	8	128334900	0,98	A	C	0.01 (0.002)	-0.028 (0.019)	-0.047 (0.028)	-0.019 (0.031)	0.009 (0.081)	-0.005 (0.055)	Wasit-to-hip ratio
rs12676719	8	135728516	1,00	G	A	0.01 (0.002)	-0.019 (0.019)	-0.012 (0.028)	-0.01 (0.03)	-0.086 (0.079)	-0.041 (0.053)	Wasit-to-hip ratio
rs7043405	9	11442765	0,99	C	T	0.009 (0.002)	0.055 (0.018)	0.038 (0.027)	0.063 (0.03)	0.026 (0.078)	0.11 (0.052)	Wasit-to-hip ratio
rs7867635	9	20241069	0,93	C	T	0.01 (0.002)	0.005 (0.019)	0.018 (0.029)	-0.019 (0.031)	0.081 (0.08)	0.001 (0.052)	Wasit-to-hip ratio
rs10968576	9	28414339	1,00	G	A	0.013 (0.002)	-0.006 (0.019)	-0.001 (0.028)	-0.01 (0.032)	-0.015 (0.083)	-0.005 (0.054)	Wasit-to-hip ratio
rs1475536	9	92212674	0,97	C	T	0.01 (0.002)	0.045 (0.018)	0.075 (0.027)	0.005 (0.03)	0.056 (0.078)	0.055 (0.052)	Wasit-to-hip ratio
rs10992380	9	95354524	1,00	T	C	0.013 (0.002)	0.011 (0.024)	0.036 (0.036)	-0.016 (0.04)	-0.015 (0.105)	0.012 (0.068)	Wasit-to-hip ratio
rs2841682	9	96160820	0,97	C	A	0.012 (0.002)	-0.03 (0.023)	-0.023 (0.035)	-0.01 (0.038)	-0.032 (0.098)	-0.113 (0.067)	Wasit-to-hip ratio
rs2398893	9	96758342	1,00	A	G	0.014 (0.002)	0.015 (0.02)	0 (0.03)	0.024 (0.033)	0.003 (0.087)	0.048 (0.057)	Wasit-to-hip ratio
rs2777795	9	107672365	0,93	G	A	0.017 (0.003)	0.015 (0.03)	-0.018 (0.045)	0.043 (0.048)	-0.045 (0.121)	0.074 (0.084)	Wasit-to-hip ratio
rs4742929	9	107689352	0,93	G	A	0.013 (0.002)	-0.009 (0.019)	-0.038 (0.028)	-0.02 (0.031)	0.096 (0.08)	0.085 (0.053)	Wasit-to-hip ratio
rs9792666	9	107887738	0,94	A	G	0.033 (0.004)	-0.01 (0.049)	-0.004 (0.075)	-0.002 (0.077)	0.028 (0.196)	-0.075 (0.135)	Wasit-to-hip ratio
rs10116353	9	111950088	1,00	G	T	0.012 (0.002)	0.027 (0.02)	0.026 (0.03)	0.027 (0.032)	-0.076 (0.085)	0.073 (0.056)	Wasit-to-hip ratio
rs7040419	9	111962817	0,99	A	G	0.012 (0.002)	0.023 (0.019)	0.02 (0.029)	0.045 (0.031)	-0.109 (0.083)	0.026 (0.054)	Wasit-to-hip ratio
rs2058432	9	126703738	0,97	C	T	0.012 (0.002)	0.015 (0.021)	0.032 (0.031)	-0.002 (0.036)	0.029 (0.094)	-0.016 (0.064)	Wasit-to-hip ratio
rs9328536	9	134914385	1,00	C	T	0.01 (0.002)	0.022 (0.019)	0.018 (0.029)	0.055 (0.032)	-0.124 (0.084)	-0.003 (0.055)	Wasit-to-hip ratio
rs12774134	10	4963327	1,00	C	T	0.015 (0.002)	0.035 (0.027)	0.041 (0.039)	0.021 (0.045)	0.029 (0.12)	0.054 (0.08)	Wasit-to-hip ratio
rs11012737	10	21849769	0,99	A	G	0.013 (0.002)	0.048 (0.019)	0.044 (0.029)	0.054 (0.032)	0.169 (0.084)	-0.004 (0.055)	Wasit-to-hip ratio
rs2778665	10	32057106	0,99	A	G	0.01 (0.002)	-0.027 (0.018)	-0.02 (0.027)	-0.054 (0.031)	0.093 (0.079)	-0.032 (0.052)	Wasit-to-hip ratio
rs1757471	10	34168090	0,98	T	C	0.01 (0.002)	-0.001 (0.018)	0.013 (0.027)	0.006 (0.03)	0.031 (0.08)	-0.08 (0.051)	Wasit-to-hip ratio
rs2804852	10	36237535	0,96	C	T	0.01 (0.002)	0.016 (0.019)	0.026 (0.028)	0.01 (0.031)	-0.013 (0.081)	0.008 (0.054)	Wasit-to-hip ratio
rs7919055	10	63808475	0,98	C	T	0.029 (0.004)	-0.015 (0.047)	-0.058 (0.068)	0.081 (0.079)	-0.19 (0.234)	-0.067 (0.136)	Wasit-to-hip ratio
rs7923609	10	65133822	1,00	A	G	0.012 (0.002)	0.011 (0.018)	-0.022 (0.027)	0.017 (0.03)	0.109 (0.077)	0.07 (0.051)	Wasit-to-hip ratio
rs703974	10	80948593	1,00	A	G	0.012 (0.002)	-0.003 (0.018)	0.006 (0.027)	0.022 (0.03)	-0.115 (0.08)	-0.06 (0.052)	Wasit-to-hip ratio
rs1234212	10	89608892	1,00	T	C	0.01 (0.002)	-0.027 (0.019)	-0.074 (0.029)	0.004 (0.032)	-0.009 (0.084)	0.042 (0.055)	Wasit-to-hip ratio
rs1437	10	93790523	1,00	A	G	0.011 (0.002)	0.012 (0.019)	0.026 (0.028)	-0.021 (0.031)	0.018 (0.082)	0.06 (0.054)	Wasit-to-hip ratio
rs2254069	10	122875589	0,98	A	G	0.018 (0.002)	-0.025 (0.028)	-0.003 (0.042)	-0.042 (0.045)	0.039 (0.118)	-0.071 (0.076)	Wasit-to-hip ratio
rs4929927	11	8658485	1,00	G	A	0.011 (0.002)	0.05 (0.019)	0.096 (0.028)	0.052 (0.031)	0.052 (0.082)	-0.134 (0.055)	Wasit-to-hip ratio
rs10840349	11	10076430	0,99	G	A	0.011 (0.002)	0.002 (0.018)	0 (0.027)	0.022 (0.03)	0.018 (0.079)	-0.058 (0.052)	Wasit-to-hip ratio
rs7932891	11	10921512	0,96	A	G	0.01 (0.002)	-0.02 (0.02)	0.013 (0.031)	-0.048 (0.032)	0.046 (0.082)	-0.078 (0.056)	Wasit-to-hip ratio

rs925946	11	27667202	1,00	T	G	0.014 (0.002)	-0.032 (0.02)	-0.086 (0.03)	0 (0.032)	-0.031 (0.083)	0.065 (0.055)	Wasit-to-hip ratio
rs11030107	11	27694835	1,00	G	A	0.015 (0.002)	-0.016 (0.021)	-0.055 (0.032)	0.007 (0.034)	-0.053 (0.088)	0.065 (0.058)	Wasit-to-hip ratio
rs4755720	11	43628749	0,99	C	T	0.01 (0.002)	0.001 (0.019)	0 (0.028)	-0.004 (0.03)	0.045 (0.08)	-0.003 (0.052)	Wasit-to-hip ratio
rs12361415	11	47474146	0,98	T	G	0.015 (0.002)	-0.039 (0.02)	-0.053 (0.03)	-0.025 (0.033)	-0.031 (0.087)	-0.036 (0.056)	Wasit-to-hip ratio
rs2509963	11	62192931	0,98	C	T	0.013 (0.002)	-0.003 (0.02)	-0.012 (0.031)	0.015 (0.033)	0.067 (0.084)	-0.059 (0.058)	Wasit-to-hip ratio
rs11231144	11	62328038	0,99	C	T	0.013 (0.002)	0.016 (0.019)	0 (0.028)	0.046 (0.031)	0.121 (0.08)	-0.06 (0.052)	Wasit-to-hip ratio
rs11231698	11	63877163	0,96	T	C	0.031 (0.004)	0.033 (0.04)	-0.033 (0.06)	0.059 (0.065)	-0.017 (0.165)	0.206 (0.111)	Wasit-to-hip ratio
rs35169799	11	64031241	0,94	T	C	0.032 (0.004)	0.034 (0.038)	0.01 (0.055)	0.05 (0.063)	0.077 (0.172)	0.065 (0.114)	Wasit-to-hip ratio
rs10896012	11	65278461	0,99	C	T	0.013 (0.002)	0.018 (0.023)	0.01 (0.035)	0.001 (0.037)	0.215 (0.094)	0.002 (0.062)	Wasit-to-hip ratio
rs2276106	11	66105817	0,93	T	C	0.012 (0.002)	0.019 (0.021)	0.016 (0.032)	0.035 (0.034)	0.038 (0.086)	-0.024 (0.058)	Wasit-to-hip ratio
rs72932183	11	69150932	0,77	G	A	0.016 (0.002)	0.027 (0.026)	0.023 (0.041)	0.06 (0.042)	-0.046 (0.108)	-0.027 (0.076)	Wasit-to-hip ratio
rs4980786	11	69239611	0,99	T	C	0.011 (0.002)	0.139 (0.018)	0.1 (0.027)	0.135 (0.03)	0.225 (0.08)	0.258 (0.052)	Wasit-to-hip ratio
rs536665	11	85322400	0,99	G	A	0.013 (0.002)	-0.002 (0.023)	-0.048 (0.035)	0.04 (0.038)	-0.004 (0.095)	0.032 (0.064)	Wasit-to-hip ratio
rs2276390	11	111895254	0,95	G	T	0.015 (0.002)	-0.001 (0.019)	-0.014 (0.029)	0.015 (0.032)	0.071 (0.082)	-0.034 (0.054)	Wasit-to-hip ratio
rs11216183	11	116781545	0,99	A	C	0.019 (0.003)	0.043 (0.03)	0.046 (0.043)	0.025 (0.05)	0.227 (0.129)	0.003 (0.088)	Wasit-to-hip ratio
rs1177563	11	118949083	0,98	A	G	0.012 (0.002)	-0.003 (0.018)	-0.006 (0.027)	0.009 (0.03)	-0.088 (0.08)	0.006 (0.052)	Wasit-to-hip ratio
rs579682	11	122014110	1,00	C	T	0.011 (0.002)	-0.002 (0.02)	-0.029 (0.03)	0.036 (0.033)	-0.03 (0.086)	-0.008 (0.056)	Wasit-to-hip ratio
rs6590683	11	133110361	0,95	T	C	0.011 (0.002)	-0.001 (0.019)	-0.008 (0.028)	0.029 (0.03)	-0.024 (0.08)	-0.057 (0.052)	Wasit-to-hip ratio
rs7222	12	2055266	0,92	T	C	0.009 (0.002)	-0.008 (0.019)	-0.02 (0.028)	0.012 (0.031)	0.004 (0.08)	-0.032 (0.054)	Wasit-to-hip ratio
rs12580347	12	3388932	0,99	C	T	0.01 (0.002)	-0.001 (0.018)	-0.035 (0.027)	0.032 (0.03)	-0.002 (0.079)	0.024 (0.052)	Wasit-to-hip ratio
rs1805741	12	9074259	0,98	C	T	0.011 (0.002)	-0.023 (0.02)	-0.025 (0.029)	-0.004 (0.033)	-0.029 (0.085)	-0.07 (0.057)	Wasit-to-hip ratio
rs718314	12	26453283	1,00	G	A	0.021 (0.002)	0.166 (0.02)	0.187 (0.029)	0.137 (0.034)	0.234 (0.087)	0.144 (0.059)	Wasit-to-hip ratio
rs4654	12	26490155	1,00	C	T	0.013 (0.002)	0.12 (0.018)	0.177 (0.028)	0.09 (0.031)	0.09 (0.08)	0.01 (0.052)	Wasit-to-hip ratio
rs11051005	12	30783475	1,00	A	G	0.01 (0.002)	-0.022 (0.021)	-0.03 (0.031)	-0.035 (0.034)	0 (0.092)	0.033 (0.059)	Wasit-to-hip ratio
rs10844642	12	33730368	1,00	A	C	0.012 (0.002)	0.001 (0.019)	-0.011 (0.028)	0.018 (0.031)	0.004 (0.08)	-0.004 (0.052)	Wasit-to-hip ratio
rs10844828	12	34303109	1,00	C	T	0.01 (0.002)	-0.01 (0.018)	-0.022 (0.027)	0.005 (0.031)	0.035 (0.078)	-0.03 (0.052)	Wasit-to-hip ratio
rs8189549	12	38245752	0,98	A	G	0.009 (0.002)	0.015 (0.019)	0.01 (0.029)	0.042 (0.031)	0.029 (0.078)	-0.05 (0.053)	Wasit-to-hip ratio
rs1026462	12	41821630	1,00	G	A	0.012 (0.002)	-0.022 (0.018)	-0.024 (0.027)	-0.002 (0.03)	-0.054 (0.076)	-0.063 (0.051)	Wasit-to-hip ratio
rs11169170	12	50223013	0,98	T	C	0.009 (0.002)	-0.016 (0.018)	-0.013 (0.027)	0.004 (0.03)	-0.111 (0.078)	-0.043 (0.051)	Wasit-to-hip ratio
rs1443512	12	54342684	1,00	A	C	0.023 (0.002)	0.004 (0.021)	0.002 (0.031)	0.032 (0.035)	-0.065 (0.091)	-0.045 (0.061)	Wasit-to-hip ratio
rs11614913	12	54385599	0,99	T	C	0.019 (0.002)	-0.021 (0.019)	-0.016 (0.028)	-0.02 (0.031)	-0.037 (0.078)	-0.034 (0.052)	Wasit-to-hip ratio
rs11176015	12	66441684	0,95	T	C	0.012 (0.002)	0.035 (0.02)	0.043 (0.03)	0.004 (0.033)	0.083 (0.084)	0.073 (0.058)	Wasit-to-hip ratio
rs10506965	12	89775029	0,99	T	G	0.011 (0.002)	-0.005 (0.018)	0.005 (0.027)	-0.006 (0.03)	0.071 (0.077)	-0.071 (0.052)	Wasit-to-hip ratio
rs11107169	12	94126855	0,98	A	G	0.01 (0.002)	0.012 (0.018)	0.029 (0.027)	-0.003 (0.03)	-0.108 (0.079)	0.044 (0.052)	Wasit-to-hip ratio
rs7973678	12	108459102	0,98	A	G	0.011 (0.002)	0.001 (0.022)	-0.05 (0.033)	0.016 (0.036)	0.068 (0.096)	0.106 (0.061)	Wasit-to-hip ratio
rs3764002	12	108618630	1,00	C	T	0.014 (0.002)	-0.006 (0.02)	-0.015 (0.029)	-0.007 (0.034)	-0.055 (0.087)	0.055 (0.058)	Wasit-to-hip ratio
rs2071272	12	121670792	0,99	A	G	0.02 (0.003)	-0.021 (0.041)	0.028 (0.061)	-0.088 (0.067)	0.136 (0.168)	-0.068 (0.116)	Wasit-to-hip ratio
rs580063	12	123206340	0,96	T	C	0.016 (0.002)	-0.008 (0.022)	0.023 (0.033)	-0.04 (0.036)	0.115 (0.095)	-0.082 (0.064)	Wasit-to-hip ratio
rs1568427	12	123738678	0,99	G	A	0.017 (0.002)	-0.041 (0.021)	-0.027 (0.031)	-0.106 (0.034)	0.117 (0.088)	0.032 (0.06)	Wasit-to-hip ratio
rs7131750	12	124258887	0,98	T	C	0.011 (0.002)	0.017 (0.018)	-0.002 (0.027)	0.013 (0.03)	0.089 (0.079)	0.07 (0.052)	Wasit-to-hip ratio
rs7133378	12	124409502	0,98	G	A	0.022 (0.002)	0.017 (0.02)	0.019 (0.029)	0.008 (0.032)	-0.001 (0.084)	0.044 (0.055)	Wasit-to-hip ratio
rs10773049	12	124506631	1,00	T	C	0.02 (0.002)	0.01 (0.018)	0.016 (0.027)	0.014 (0.03)	0.016 (0.078)	-0.03 (0.052)	Wasit-to-hip ratio
rs1360485	13	31031884	1,00	T	C	0.013 (0.002)	0.013 (0.02)	0.034 (0.029)	-0.005 (0.033)	0.093 (0.088)	-0.044 (0.055)	Wasit-to-hip ratio
rs6561490	13	49540727	0,99	C	A	0.009 (0.002)	0 (0.018)	-0.009 (0.027)	0.013 (0.03)	-0.03 (0.08)	0.003 (0.051)	Wasit-to-hip ratio
rs558003	13	51172963	0,96	G	A	0.027 (0.003)	0.014 (0.032)	0.059 (0.048)	0.016 (0.052)	0.041 (0.135)	-0.169 (0.091)	Wasit-to-hip ratio
rs797486	13	51221618	0,98	A	C	0.027 (0.002)	0.01 (0.028)	0.048 (0.043)	-0.006 (0.046)	-0.001 (0.118)	-0.069 (0.078)	Wasit-to-hip ratio
rs1379828	13	54396602	0,99	C	T	0.012 (0.002)	-0.003 (0.022)	-0.031 (0.034)	0.042 (0.037)	-0.02 (0.096)	-0.032 (0.063)	Wasit-to-hip ratio
rs9596914	13	54690617	0,95	G	T	0.017 (0.003)	0.063 (0.03)	0.056 (0.045)	0.049 (0.048)	0.107 (0.126)	0.114 (0.085)	Wasit-to-hip ratio
rs1441264	13	79580919	1,00	A	G	0.01 (0.002)	-0.01 (0.018)	-0.013 (0.027)	-0.008 (0.03)	-0.11 (0.08)	0.035 (0.052)	Wasit-to-hip ratio

rs9556979	13	99241507	0,94	G	T	0.01 (0.002)	0.025 (0.02)	0.027 (0.031)	0.034 (0.032)	0.106 (0.086)	-0.039 (0.055)	Wasit-to-hip ratio
rs9515201	13	111040798	0,98	C	A	0.01 (0.002)	-0.005 (0.02)	-0.013 (0.03)	0.017 (0.032)	-0.032 (0.086)	-0.028 (0.056)	Wasit-to-hip ratio
rs1163627	13	112225701	0,99	A	C	0.01 (0.002)	0.016 (0.018)	0.008 (0.027)	0.026 (0.03)	-0.076 (0.078)	0.054 (0.051)	Wasit-to-hip ratio
rs61986154	14	53937418	0,99	A	G	0.012 (0.002)	0.017 (0.021)	0 (0.031)	0.011 (0.035)	0.009 (0.088)	0.102 (0.061)	Wasit-to-hip ratio
rs1190982	14	58815839	0,98	T	C	0.013 (0.002)	0.035 (0.02)	0.038 (0.03)	0.038 (0.032)	-0.002 (0.086)	0.034 (0.056)	Wasit-to-hip ratio
rs2412107	14	65426216	0,98	T	G	0.012 (0.002)	-0.037 (0.022)	-0.08 (0.033)	0.039 (0.036)	-0.117 (0.092)	-0.075 (0.062)	Wasit-to-hip ratio
rs2526886	14	71359064	0,99	T	G	0.011 (0.002)	-0.018 (0.02)	-0.025 (0.029)	0.004 (0.032)	-0.079 (0.084)	-0.029 (0.056)	Wasit-to-hip ratio
rs17109256	14	79939993	1,00	A	G	0.012 (0.002)	0.027 (0.022)	0.019 (0.032)	0.02 (0.036)	0.1 (0.095)	0.046 (0.062)	Wasit-to-hip ratio
rs7143963	14	103304425	1,00	T	C	0.014 (0.002)	-0.005 (0.024)	-0.083 (0.035)	0.08 (0.039)	-0.009 (0.1)	0.024 (0.068)	Wasit-to-hip ratio
rs6493498	15	51754451	0,99	T	C	0.01 (0.002)	0.011 (0.018)	-0.009 (0.028)	0.041 (0.03)	0.087 (0.077)	-0.044 (0.052)	Wasit-to-hip ratio
rs2440376	15	53091870	1,00	A	G	0.013 (0.002)	0.003 (0.026)	0.008 (0.04)	0.003 (0.043)	-0.131 (0.112)	0.043 (0.072)	Wasit-to-hip ratio
rs16976932	15	56781255	0,95	A	G	0.015 (0.002)	-0.001 (0.03)	-0.016 (0.047)	0.039 (0.048)	-0.099 (0.124)	-0.023 (0.082)	Wasit-to-hip ratio
rs3784634	15	62259637	0,99	C	T	0.009 (0.002)	-0.015 (0.018)	-0.004 (0.027)	0 (0.03)	0.015 (0.077)	-0.113 (0.052)	Wasit-to-hip ratio
rs1440372	15	67033151	0,99	C	T	0.01 (0.002)	0.028 (0.02)	0.022 (0.029)	0.024 (0.033)	0.083 (0.085)	0.037 (0.058)	Wasit-to-hip ratio
rs8043060	15	67661784	0,99	G	A	0.014 (0.002)	0.031 (0.022)	-0.018 (0.033)	0.043 (0.036)	0.133 (0.092)	0.118 (0.06)	Wasit-to-hip ratio
rs4776964	15	68016955	1,00	T	G	0.013 (0.002)	0.009 (0.021)	0.014 (0.032)	0.028 (0.034)	-0.068 (0.085)	-0.023 (0.059)	Wasit-to-hip ratio
rs7164727	15	73093991	0,99	T	C	0.01 (0.002)	0.012 (0.019)	0.011 (0.029)	0.029 (0.032)	-0.027 (0.082)	-0.015 (0.055)	Wasit-to-hip ratio
rs9479	15	74328576	0,99	G	A	0.011 (0.002)	0.015 (0.018)	-0.002 (0.027)	0.05 (0.03)	0.054 (0.077)	-0.045 (0.052)	Wasit-to-hip ratio
rs876383	15	74333413	1,00	G	A	0.01 (0.002)	0.001 (0.018)	-0.033 (0.027)	0.036 (0.03)	0.073 (0.077)	-0.014 (0.052)	Wasit-to-hip ratio
rs72744599	15	77972842	1,00	G	C	0.015 (0.003)	-0.014 (0.027)	0.005 (0.041)	-0.057 (0.045)	-0.036 (0.118)	0.06 (0.08)	Wasit-to-hip ratio
rs12593088	15	81058640	1,00	G	A	0.01 (0.002)	-0.015 (0.02)	-0.033 (0.029)	0.003 (0.032)	-0.043 (0.084)	0.013 (0.055)	Wasit-to-hip ratio
rs12101386	15	92571283	0,99	G	T	0.012 (0.002)	-0.054 (0.022)	-0.039 (0.033)	-0.097 (0.036)	0.076 (0.097)	-0.034 (0.062)	Wasit-to-hip ratio
rs8024294	15	94023132	0,95	A	G	0.017 (0.003)	0.002 (0.031)	0.03 (0.048)	0.024 (0.05)	-0.178 (0.135)	-0.07 (0.081)	Wasit-to-hip ratio
rs36232	16	2199788	0,90	G	T	0.014 (0.002)	-0.042 (0.025)	-0.147 (0.038)	0.078 (0.041)	0.103 (0.102)	-0.096 (0.065)	Wasit-to-hip ratio
rs3747579	16	4445327	1,00	C	T	0.011 (0.002)	-0.032 (0.02)	-0.001 (0.031)	-0.049 (0.033)	0.036 (0.087)	-0.117 (0.056)	Wasit-to-hip ratio
rs7186893	16	24806420	1,00	G	T	0.013 (0.002)	0.013 (0.02)	-0.01 (0.03)	0.044 (0.034)	0.051 (0.086)	-0.007 (0.058)	Wasit-to-hip ratio
rs2008514	16	28825605	0,96	A	G	0.013 (0.002)	0.042 (0.019)	0.061 (0.028)	0.015 (0.03)	0.15 (0.079)	0.006 (0.052)	Wasit-to-hip ratio
rs4788196	16	29967434	0,99	G	A	0.014 (0.002)	0.02 (0.018)	0.032 (0.027)	0.012 (0.03)	0.012 (0.078)	-0.002 (0.051)	Wasit-to-hip ratio
rs3814883	16	29994922	0,94	T	C	0.015 (0.002)	0.018 (0.018)	0.023 (0.027)	0.015 (0.031)	0.009 (0.081)	0.014 (0.053)	Wasit-to-hip ratio
rs1421085	16	53800954	0,99	C	T	0.032 (0.002)	0.035 (0.018)	0.021 (0.028)	0.079 (0.03)	-0.032 (0.077)	-0.012 (0.052)	Wasit-to-hip ratio
rs9936385	16	53819169	1,00	C	T	0.03 (0.002)	0.033 (0.018)	0.008 (0.027)	0.093 (0.03)	-0.034 (0.077)	-0.023 (0.052)	Wasit-to-hip ratio
rs889398	16	69556715	0,99	C	T	0.014 (0.002)	0.023 (0.018)	0.009 (0.028)	0.04 (0.03)	0.178 (0.077)	-0.051 (0.052)	Wasit-to-hip ratio
rs2925979	16	81534790	1,00	T	C	0.016 (0.002)	-0.004 (0.02)	0.004 (0.03)	-0.016 (0.032)	-0.031 (0.087)	0.016 (0.055)	Wasit-to-hip ratio
rs7217226	17	2136065	0,98	G	T	0.012 (0.002)	0.006 (0.019)	0.029 (0.029)	-0.042 (0.031)	-0.03 (0.081)	0.088 (0.054)	Wasit-to-hip ratio
rs8070737	17	3981066	0,99	T	G	0.013 (0.002)	0.033 (0.024)	0.011 (0.035)	0.072 (0.04)	0.153 (0.102)	-0.048 (0.066)	Wasit-to-hip ratio
rs9900673	17	17548633	0,97	C	T	0.012 (0.002)	0.045 (0.019)	0.013 (0.028)	0.039 (0.031)	0.076 (0.08)	0.156 (0.052)	Wasit-to-hip ratio
rs7213608	17	21279289	0,95	C	T	0.013 (0.002)	-0.037 (0.02)	-0.054 (0.031)	-0.058 (0.032)	0.004 (0.087)	0.061 (0.055)	Wasit-to-hip ratio
rs2306589	17	34848874	0,98	T	C	0.013 (0.002)	0.045 (0.018)	0.038 (0.028)	0.109 (0.03)	-0.027 (0.077)	-0.092 (0.051)	Wasit-to-hip ratio
rs16967620	17	40422341	1,00	T	G	0.012 (0.002)	-0.004 (0.02)	-0.027 (0.029)	0.01 (0.033)	-0.009 (0.085)	0.038 (0.055)	Wasit-to-hip ratio
rs113382777	17	41058634	0,81	T	C	0.036 (0.006)	0.044 (0.066)	-0.079 (0.093)	0.057 (0.117)	0.516 (0.301)	0.328 (0.185)	Wasit-to-hip ratio
rs2071167	17	42287519	0,98	T	C	0.011 (0.002)	0 (0.021)	-0.013 (0.031)	0.021 (0.034)	0.017 (0.097)	-0.015 (0.061)	Wasit-to-hip ratio
rs1876829	17	43911443	1,00	C	T	0.014 (0.002)	0.035 (0.022)	0.06 (0.034)	0.029 (0.036)	-0.012 (0.092)	-0.003 (0.059)	Wasit-to-hip ratio
rs113564729	17	44179095	0,91	G	A	0.015 (0.002)	0.004 (0.02)	-0.022 (0.032)	0.033 (0.031)	-0.028 (0.08)	0.01 (0.053)	Wasit-to-hip ratio
rs62074125	17	44852612	0,73	A	C	0.016 (0.002)	0.029 (0.025)	NA (NA)	0.054 (0.038)	-0.053 (0.097)	-0.006 (0.066)	Wasit-to-hip ratio
rs916888	17	44863133	0,93	C	T	0.012 (0.002)	0.029 (0.022)	0.042 (0.034)	0.042 (0.036)	-0.052 (0.094)	-0.015 (0.059)	Wasit-to-hip ratio
rs11079810	17	46227846	0,99	C	T	0.016 (0.002)	-0.001 (0.029)	-0.049 (0.042)	0.041 (0.049)	0.044 (0.133)	0.056 (0.086)	Wasit-to-hip ratio
rs757608	17	59497277	1,00	A	G	0.01 (0.002)	-0.012 (0.019)	-0.019 (0.029)	-0.018 (0.032)	0.001 (0.083)	0.026 (0.054)	Wasit-to-hip ratio
rs2854152	17	61986027	0,94	A	G	0.012 (0.002)	-0.016 (0.02)	-0.046 (0.03)	-0.011 (0.032)	0.022 (0.086)	0.058 (0.056)	Wasit-to-hip ratio
rs12602912	17	65870073	1,00	T	C	0.016 (0.002)	-0.004 (0.023)	-0.001 (0.034)	0.004 (0.037)	-0.059 (0.098)	-0.02 (0.064)	Wasit-to-hip ratio

rs6501392	17	68434200	1,00	A	G	0.012 (0.002)	-0.014 (0.018)	-0.039 (0.027)	0.017 (0.03)	-0.014 (0.077)	-0.015 (0.052)	Wasit-to-hip ratio
rs2242231	17	73242935	1,00	G	A	0.014 (0.002)	-0.018 (0.024)	-0.04 (0.035)	0.006 (0.039)	-0.066 (0.1)	0.018 (0.068)	Wasit-to-hip ratio
rs8079062	17	74255029	0,99	G	A	0.018 (0.003)	-0.093 (0.035)	-0.099 (0.053)	-0.111 (0.058)	-0.096 (0.139)	-0.024 (0.095)	Wasit-to-hip ratio
rs4077074	17	79979642	0,85	T	C	0.012 (0.002)	-0.014 (0.021)	-0.044 (0.033)	0 (0.034)	-0.004 (0.093)	0.032 (0.056)	Wasit-to-hip ratio
rs3810068	18	2846499	0,86	T	C	0.013 (0.002)	-0.016 (0.02)	-0.032 (0.027)	0.02 (0.035)	-0.05 (0.078)	NA (NA)	Wasit-to-hip ratio
rs1787013	18	13072979	1,00	C	T	0.009 (0.002)	-0.011 (0.018)	-0.003 (0.027)	0.014 (0.03)	-0.016 (0.077)	-0.118 (0.052)	Wasit-to-hip ratio
rs11663645	18	21067076	1,00	A	G	0.01 (0.002)	0.037 (0.019)	0.034 (0.028)	0.032 (0.031)	0.131 (0.083)	0.022 (0.054)	Wasit-to-hip ratio
rs7231852	18	40761976	1,00	G	A	0.011 (0.002)	-0.032 (0.02)	-0.024 (0.03)	-0.048 (0.034)	-0.065 (0.088)	0.001 (0.058)	Wasit-to-hip ratio
rs7239114	18	45921214	0,98	A	G	0.009 (0.002)	-0.024 (0.018)	-0.024 (0.027)	-0.046 (0.03)	-0.15 (0.079)	0.096 (0.052)	Wasit-to-hip ratio
rs9951872	18	46678832	1,00	A	G	0.016 (0.002)	-0.026 (0.024)	-0.038 (0.035)	0.026 (0.04)	0.081 (0.105)	-0.181 (0.07)	Wasit-to-hip ratio
rs530550	18	46851638	1,00	T	G	0.013 (0.002)	0.031 (0.019)	0.021 (0.028)	0.07 (0.032)	0.119 (0.081)	-0.08 (0.053)	Wasit-to-hip ratio
rs7235891	18	53454774	1,00	C	T	0.01 (0.002)	0.018 (0.018)	0.021 (0.027)	-0.01 (0.03)	0.049 (0.078)	0.077 (0.052)	Wasit-to-hip ratio
rs663129	18	57838401	1,00	A	G	0.021 (0.002)	0.045 (0.021)	0.063 (0.032)	0.014 (0.035)	0.086 (0.092)	0.058 (0.06)	Wasit-to-hip ratio
rs17773412	18	57960769	0,99	C	T	0.015 (0.002)	-0.016 (0.02)	-0.004 (0.029)	-0.04 (0.032)	0.129 (0.085)	-0.05 (0.055)	Wasit-to-hip ratio
rs112297194	18	58056958	0,97	G	A	0.044 (0.006)	0.018 (0.066)	-0.049 (0.1)	0.17 (0.108)	0.225 (0.28)	-0.265 (0.177)	Wasit-to-hip ratio
rs12459350	19	2176586	1,00	A	G	0.01 (0.002)	-0.057 (0.018)	-0.041 (0.027)	-0.084 (0.03)	-0.061 (0.077)	-0.03 (0.051)	Wasit-to-hip ratio
rs73504817	19	17167723	1,00	T	C	0.012 (0.002)	0.008 (0.02)	0.006 (0.029)	0.027 (0.033)	-0.095 (0.084)	0.011 (0.057)	Wasit-to-hip ratio
rs273507	19	18221964	0,97	C	A	0.018 (0.002)	0.001 (0.02)	-0.016 (0.03)	0.024 (0.033)	-0.112 (0.085)	0.04 (0.057)	Wasit-to-hip ratio
rs12608504	19	18389135	1,00	A	G	0.019 (0.002)	0.005 (0.019)	0.008 (0.028)	0.004 (0.031)	0.009 (0.083)	-0.005 (0.053)	Wasit-to-hip ratio
rs6511027	19	19391851	0,99	C	T	0.014 (0.002)	-0.006 (0.025)	0.011 (0.037)	0.021 (0.041)	-0.084 (0.105)	-0.107 (0.069)	Wasit-to-hip ratio
rs17513613	19	30286822	0,99	C	T	0.012 (0.002)	0.026 (0.019)	0.054 (0.029)	-0.003 (0.031)	-0.093 (0.084)	0.061 (0.055)	Wasit-to-hip ratio
rs10403561	19	33790502	0,88	A	G	0.017 (0.002)	-0.043 (0.02)	-0.02 (0.03)	-0.071 (0.031)	0.014 (0.086)	-0.053 (0.051)	Wasit-to-hip ratio
rs3786897	19	33893008	0,98	G	A	0.019 (0.002)	-0.012 (0.018)	-0.012 (0.028)	-0.039 (0.03)	0.092 (0.081)	0.02 (0.052)	Wasit-to-hip ratio
rs4420638	19	45422946	0,83	A	G	0.023 (0.002)	0.05 (0.026)	0.073 (0.041)	0.013 (0.043)	-0.082 (0.11)	0.119 (0.064)	Wasit-to-hip ratio
rs11672660	19	46180184	0,98	C	T	0.017 (0.002)	0.055 (0.022)	0.033 (0.033)	0.081 (0.036)	0.112 (0.096)	0.032 (0.064)	Wasit-to-hip ratio
rs3810291	19	47569003	0,93	A	G	0.01 (0.002)	-0.023 (0.02)	-0.04 (0.031)	0.022 (0.032)	-0.04 (0.087)	-0.094 (0.055)	Wasit-to-hip ratio
rs8103017	19	55999142	0,87	G	C	0.013 (0.002)	0.017 (0.022)	-0.013 (0.033)	0.058 (0.035)	0.07 (0.091)	-0.031 (0.06)	Wasit-to-hip ratio
rs805770	20	5668714	1,00	T	C	0.015 (0.002)	0.026 (0.018)	0.009 (0.027)	0.003 (0.03)	0.136 (0.079)	0.111 (0.053)	Wasit-to-hip ratio
rs143384	20	34025756	0,92	A	G	0.013 (0.002)	0.013 (0.019)	0.006 (0.029)	0.055 (0.031)	0.01 (0.083)	-0.09 (0.055)	Wasit-to-hip ratio
rs4812492	20	39938122	0,98	C	T	0.01 (0.002)	0.014 (0.018)	-0.015 (0.027)	0.051 (0.03)	-0.025 (0.077)	0.025 (0.054)	Wasit-to-hip ratio
rs2236519	20	45529571	0,99	A	G	0.016 (0.002)	0.019 (0.019)	0.024 (0.028)	0.014 (0.031)	0.092 (0.079)	-0.014 (0.053)	Wasit-to-hip ratio
rs2073170	20	45790289	1,00	G	A	0.011 (0.002)	0.008 (0.018)	-0.009 (0.027)	0.012 (0.03)	-0.048 (0.078)	0.081 (0.052)	Wasit-to-hip ratio
rs6021889	20	50982870	0,99	A	G	0.016 (0.002)	0.039 (0.02)	0.047 (0.03)	0.064 (0.034)	-0.12 (0.09)	0.007 (0.057)	Wasit-to-hip ratio
rs910382	20	51699189	1,00	G	A	0.013 (0.002)	-0.014 (0.018)	-0.003 (0.027)	-0.048 (0.03)	0.055 (0.077)	0.013 (0.051)	Wasit-to-hip ratio
rs6122049	20	62692789	0,95	T	C	0.012 (0.002)	-0.034 (0.022)	-0.058 (0.032)	0.001 (0.036)	0.009 (0.093)	-0.065 (0.062)	Wasit-to-hip ratio
rs2211854	21	39486320	0,98	A	C	0.011 (0.002)	0.002 (0.018)	-0.005 (0.028)	0.033 (0.03)	-0.018 (0.078)	-0.059 (0.052)	Wasit-to-hip ratio
rs510197	22	27584316	1,00	G	A	0.013 (0.002)	-0.004 (0.023)	-0.036 (0.034)	0.004 (0.038)	0.249 (0.098)	-0.022 (0.064)	Wasit-to-hip ratio
rs2294239	22	29449477	1,00	A	G	0.017 (0.002)	0.004 (0.018)	0.01 (0.027)	-0.002 (0.03)	0.034 (0.079)	-0.018 (0.052)	Wasit-to-hip ratio
rs733381	22	40669648	0,99	A	G	0.011 (0.002)	0.018 (0.022)	0.026 (0.033)	0.044 (0.036)	-0.048 (0.094)	-0.06 (0.063)	Wasit-to-hip ratio
rs12124126	1	6660349	1,00	A	G	0.012 (0.002)	-0.008 (0.019)	0.012 (0.028)	-0.045 (0.031)	0.059 (0.081)	0.003 (0.054)	Body fat %
rs4908677	1	7738180	0,95	T	C	0.011 (0.002)	-0.017 (0.018)	-0.024 (0.028)	0.002 (0.03)	0.073 (0.081)	-0.085 (0.052)	Body fat %
rs159961	1	8484228	0,99	T	C	0.012 (0.002)	-0.029 (0.02)	-0.061 (0.029)	0.004 (0.032)	0.044 (0.082)	-0.052 (0.054)	Body fat %
rs1318408	1	11925781	0,98	G	A	0.016 (0.003)	0.019 (0.029)	-0.035 (0.045)	0.079 (0.046)	0.116 (0.118)	-0.033 (0.079)	Body fat %
rs3766823	1	32197257	0,95	A	G	0.017 (0.002)	0.012 (0.024)	0.002 (0.037)	0.074 (0.038)	-0.162 (0.104)	-0.074 (0.068)	Body fat %
rs2050256	1	32204683	0,94	G	A	0.016 (0.002)	0.01 (0.024)	-0.002 (0.037)	0.073 (0.039)	-0.182 (0.105)	-0.063 (0.068)	Body fat %
rs6686901	1	42436054	1,00	C	T	0.012 (0.002)	0.016 (0.018)	-0.003 (0.027)	0.039 (0.03)	0 (0.077)	0.023 (0.052)	Body fat %
rs138556772	1	46174888	0,84	G	A	0.027 (0.004)	0.012 (0.052)	0.05 (0.084)	0.037 (0.082)	0.065 (0.234)	-0.164 (0.133)	Body fat %
rs2224976	1	49486981	0,89	A	G	0.012 (0.002)	-0.003 (0.02)	0.009 (0.03)	0.006 (0.032)	-0.089 (0.085)	-0.036 (0.056)	Body fat %
rs1167309	1	49997674	1,00	C	T	0.014 (0.002)	-0.018 (0.019)	-0.025 (0.028)	0.001 (0.032)	-0.072 (0.084)	-0.026 (0.054)	Body fat %

rs12566626	1	50499636	1,00	T	C	0.013 (0.002)	-0.018 (0.019)	-0.027 (0.029)	0.008 (0.032)	-0.072 (0.084)	-0.034 (0.055)	Body fat %
rs1013293	1	62570321	0,99	G	A	0.014 (0.002)	-0.004 (0.018)	-0.01 (0.027)	-0.006 (0.03)	-0.012 (0.079)	0.021 (0.052)	Body fat %
rs12140153	1	62579891	0,84	G	T	0.025 (0.003)	-0.018 (0.035)	-0.051 (0.051)	0.058 (0.06)	-0.085 (0.148)	-0.065 (0.098)	Body fat %
rs2186120	1	66453163	1,00	A	G	0.01 (0.002)	0.01 (0.018)	0.032 (0.027)	-0.005 (0.03)	-0.122 (0.078)	0.03 (0.051)	Body fat %
rs2815764	1	72754123	0,99	G	A	0.02 (0.002)	0.009 (0.024)	-0.043 (0.037)	0.084 (0.04)	0.074 (0.102)	-0.059 (0.064)	Body fat %
rs3104464	1	77966230	0,99	A	G	0.015 (0.002)	0.032 (0.022)	0.016 (0.032)	0.047 (0.035)	0.094 (0.089)	0.014 (0.06)	Body fat %
rs71658797	1	77967507	0,96	A	T	0.023 (0.003)	0.078 (0.03)	0.097 (0.047)	0.078 (0.049)	0.166 (0.12)	-0.012 (0.077)	Body fat %
rs17391694	1	78623626	0,83	T	C	0.019 (0.003)	0.057 (0.031)	0.085 (0.051)	0.039 (0.05)	0.16 (0.112)	-0.023 (0.083)	Body fat %
rs6688826	1	80812329	0,99	C	T	0.011 (0.002)	-0.006 (0.02)	-0.007 (0.031)	-0.003 (0.033)	0.034 (0.086)	-0.026 (0.056)	Body fat %
rs2181375	1	96940119	1,00	G	A	0.015 (0.002)	0.032 (0.018)	0.04 (0.027)	0.028 (0.03)	0.012 (0.078)	0.02 (0.052)	Body fat %
rs12072739	1	98315893	1,00	G	A	0.014 (0.002)	-0.04 (0.021)	-0.042 (0.031)	-0.031 (0.035)	0.035 (0.095)	-0.089 (0.061)	Body fat %
rs2077569	1	103350876	1,00	G	A	0.011 (0.002)	-0.004 (0.018)	-0.009 (0.027)	0.015 (0.03)	-0.068 (0.078)	-0.009 (0.052)	Body fat %
rs1730858	1	107619244	0,94	T	C	0.011 (0.002)	-0.001 (0.019)	-0.008 (0.029)	0.036 (0.032)	-0.04 (0.084)	-0.07 (0.055)	Body fat %
rs17024393	1	110154688	0,99	C	T	0.047 (0.006)	0.061 (0.048)	0.033 (0.065)	0.048 (0.084)	0.624 (0.266)	0.07 (0.16)	Body fat %
rs2306937	1	113246506	0,98	C	T	0.015 (0.002)	-0.002 (0.022)	-0.027 (0.033)	-0.027 (0.037)	0.091 (0.098)	0.126 (0.064)	Body fat %
rs11205303	1	149906413	0,95	C	T	0.017 (0.002)	-0.019 (0.019)	-0.014 (0.029)	-0.033 (0.031)	-0.092 (0.081)	0.04 (0.052)	Body fat %
rs143453062	1	150340182	0,71	A	G	0.031 (0.004)	-0.044 (0.053)	NA (NA)	0.003 (0.076)	-0.022 (0.192)	-0.006 (0.133)	Body fat %
rs9659073	1	150527354	1,00	A	G	0.012 (0.002)	0.019 (0.018)	0.048 (0.027)	-0.006 (0.03)	-0.002 (0.079)	-0.001 (0.05)	Body fat %
rs146468719	1	151000790	0,88	A	C	0.013 (0.002)	0.032 (0.024)	0.038 (0.036)	0.027 (0.039)	0.06 (0.104)	0.01 (0.069)	Body fat %
rs35154152	1	155172725	0,99	T	C	0.02 (0.003)	-0.002 (0.03)	-0.011 (0.045)	0.026 (0.049)	0.07 (0.132)	-0.077 (0.084)	Body fat %
rs61813324	1	156049877	0,73	T	C	0.017 (0.003)	-0.015 (0.031)	-0.023 (0.045)	0.025 (0.051)	-0.1 (0.124)	-0.06 (0.09)	Body fat %
rs148137538	1	173399677	0,82	A	G	0.034 (0.006)	-0.074 (0.069)	-0.031 (0.103)	-0.072 (0.116)	-0.307 (0.284)	-0.12 (0.188)	Body fat %
rs77560793	1	175001179	0,93	G	A	0.031 (0.005)	-0.121 (0.057)	-0.129 (0.088)	-0.095 (0.094)	-0.414 (0.234)	-0.044 (0.148)	Body fat %
rs543874	1	177889480	1,00	G	A	0.028 (0.002)	0.007 (0.023)	0.058 (0.036)	-0.027 (0.037)	0.058 (0.097)	-0.07 (0.062)	Body fat %
rs9425633	1	184657251	1,00	C	T	0.012 (0.002)	0.012 (0.018)	0.028 (0.027)	-0.011 (0.03)	0.015 (0.079)	0.022 (0.051)	Body fat %
rs672313	1	195142845	1,00	G	A	0.014 (0.002)	0.016 (0.022)	0.063 (0.032)	-0.026 (0.036)	-0.159 (0.094)	0.035 (0.064)	Body fat %
rs2678204	1	201800511	1,00	G	T	0.014 (0.002)	-0.006 (0.019)	-0.005 (0.028)	-0.03 (0.032)	0.057 (0.082)	0.033 (0.054)	Body fat %
rs2644135	1	201856256	0,93	C	G	0.014 (0.002)	0.003 (0.02)	0.004 (0.029)	-0.016 (0.032)	0.064 (0.083)	0.029 (0.055)	Body fat %
rs11119208	1	209211968	1,00	A	G	0.011 (0.002)	0.017 (0.018)	-0.002 (0.028)	0.023 (0.03)	-0.11 (0.078)	0.121 (0.052)	Body fat %
rs78508049	1	210344884	1,00	C	T	0.014 (0.002)	-0.019 (0.024)	-0.005 (0.036)	-0.056 (0.039)	0.021 (0.095)	0.021 (0.067)	Body fat %
rs2494196	1	219762581	0,99	A	C	0.022 (0.002)	-0.005 (0.02)	0.028 (0.03)	-0.037 (0.033)	-0.111 (0.086)	0.021 (0.056)	Body fat %
rs12133169	1	219792380	0,96	A	G	0.018 (0.002)	-0.006 (0.022)	-0.009 (0.033)	-0.027 (0.036)	-0.028 (0.094)	0.076 (0.06)	Body fat %
rs12042959	1	243533273	0,99	A	G	0.016 (0.002)	-0.007 (0.026)	0.03 (0.038)	-0.043 (0.042)	0.081 (0.109)	-0.084 (0.074)	Body fat %
rs13393304	2	637830	1,00	G	A	0.028 (0.002)	-0.011 (0.024)	-0.041 (0.035)	-0.019 (0.039)	0.119 (0.103)	0.07 (0.068)	Body fat %
rs11096549	2	16607101	0,96	T	C	0.012 (0.002)	-0.004 (0.02)	-0.024 (0.029)	0.016 (0.033)	-0.037 (0.084)	0.028 (0.058)	Body fat %
rs141240885	2	24449850	0,95	T	G	0.024 (0.004)	-0.05 (0.04)	-0.081 (0.059)	-0.037 (0.065)	0.042 (0.162)	-0.026 (0.111)	Body fat %
rs78265103	2	24468191	0,91	A	T	0.024 (0.004)	-0.049 (0.044)	-0.1 (0.066)	-0.009 (0.072)	0.204 (0.183)	-0.102 (0.119)	Body fat %
rs6752378	2	25150116	1,00	A	C	0.022 (0.002)	-0.011 (0.018)	-0.014 (0.027)	-0.016 (0.03)	-0.009 (0.077)	0.016 (0.052)	Body fat %
rs76286777	2	25195577	0,98	C	T	0.023 (0.002)	0.005 (0.023)	-0.009 (0.036)	0.028 (0.038)	0.022 (0.097)	-0.027 (0.063)	Body fat %
rs1731260	2	26953354	0,99	T	G	0.012 (0.002)	-0.022 (0.018)	-0.046 (0.027)	0.001 (0.03)	-0.066 (0.077)	0.014 (0.052)	Body fat %
rs11678385	2	30492847	0,98	G	A	0.01 (0.002)	0.028 (0.018)	0.002 (0.027)	0.03 (0.03)	0.167 (0.078)	0.056 (0.052)	Body fat %
rs10172196	2	36780549	0,99	A	G	0.013 (0.002)	0.031 (0.02)	0.009 (0.03)	0.058 (0.032)	0.018 (0.088)	0.032 (0.056)	Body fat %
rs10169594	2	41637688	0,97	C	T	0.013 (0.002)	-0.033 (0.019)	-0.045 (0.028)	-0.025 (0.032)	0.029 (0.082)	-0.036 (0.055)	Body fat %
rs113019802	2	46884824	0,99	G	A	0.014 (0.002)	-0.013 (0.022)	-0.011 (0.032)	-0.031 (0.036)	-0.052 (0.092)	0.051 (0.062)	Body fat %
rs2436772	2	47283557	1,00	G	A	0.015 (0.002)	0.021 (0.022)	0.037 (0.034)	-0.042 (0.037)	0.131 (0.096)	0.106 (0.063)	Body fat %
rs13406839	2	50735433	0,99	A	G	0.011 (0.002)	-0.01 (0.018)	-0.011 (0.027)	-0.027 (0.031)	0.054 (0.082)	0.021 (0.053)	Body fat %
rs13387836	2	55278559	0,98	T	C	0.012 (0.002)	0.02 (0.02)	0.033 (0.031)	0.019 (0.033)	0.03 (0.084)	-0.024 (0.056)	Body fat %
rs7601895	2	55281901	0,98	C	G	0.012 (0.002)	0.024 (0.02)	0.032 (0.031)	0.028 (0.033)	0.04 (0.084)	-0.022 (0.056)	Body fat %
rs1559556	2	57332617	0,99	G	A	0.011 (0.002)	0.055 (0.019)	0.029 (0.028)	0.09 (0.031)	0.083 (0.08)	0.034 (0.053)	Body fat %

rs7608397	2	58769042	0,99	G	T	0.011 (0.002)	0.037 (0.018)	0.021 (0.027)	0.059 (0.03)	-0.001 (0.077)	0.05 (0.052)	Body fat %
rs11125768	2	59306564	0,99	T	C	0.015 (0.002)	0.027 (0.018)	-0.006 (0.027)	0.041 (0.03)	0.137 (0.08)	0.061 (0.052)	Body fat %
rs6739755	2	59330227	0,99	A	G	0.015 (0.002)	0.027 (0.018)	-0.009 (0.028)	0.045 (0.03)	0.148 (0.08)	0.049 (0.052)	Body fat %
rs12477088	2	67841326	1,00	T	C	0.012 (0.002)	0.012 (0.018)	0.01 (0.027)	0.012 (0.03)	-0.056 (0.079)	0.052 (0.053)	Body fat %
rs3552	2	69698158	0,98	A	G	0.013 (0.002)	-0.015 (0.018)	-0.021 (0.027)	-0.004 (0.03)	0.048 (0.08)	-0.055 (0.052)	Body fat %
rs12619178	2	100838157	0,98	C	T	0.014 (0.002)	-0.006 (0.019)	0.024 (0.027)	-0.036 (0.031)	0.05 (0.078)	-0.057 (0.054)	Body fat %
rs6730157	2	135907088	0,95	A	G	0.013 (0.002)	0.021 (0.02)	0.043 (0.03)	0.007 (0.033)	0.036 (0.087)	-0.017 (0.056)	Body fat %
rs1446585	2	136407479	0,95	A	G	0.015 (0.002)	0.006 (0.02)	0.015 (0.03)	-0.003 (0.033)	0.004 (0.087)	0.002 (0.06)	Body fat %
rs10181181	2	161087411	0,98	C	T	0.012 (0.002)	0.01 (0.02)	0.029 (0.03)	-0.006 (0.033)	-0.101 (0.087)	0.041 (0.056)	Body fat %
rs6717858	2	165539661	0,98	C	T	0.018 (0.002)	-0.038 (0.018)	-0.01 (0.028)	-0.055 (0.03)	-0.136 (0.079)	-0.05 (0.053)	Body fat %
rs12477385	2	166144850	0,94	G	T	0.012 (0.002)	0.038 (0.023)	0.099 (0.033)	-0.033 (0.038)	-0.059 (0.097)	0.066 (0.064)	Body fat %
rs4668314	2	171631258	0,99	G	T	0.01 (0.002)	-0.023 (0.019)	0.002 (0.028)	-0.07 (0.031)	-0.008 (0.08)	0.017 (0.053)	Body fat %
rs2129475	2	172916772	0,95	G	A	0.011 (0.002)	-0.027 (0.019)	-0.026 (0.029)	-0.042 (0.031)	-0.024 (0.081)	0.01 (0.053)	Body fat %
rs79869125	2	176422238	0,96	G	T	0.019 (0.003)	0.024 (0.03)	0.075 (0.044)	-0.045 (0.049)	-0.06 (0.126)	0.072 (0.086)	Body fat %
rs12622267	2	181568007	0,98	A	G	0.013 (0.002)	0.029 (0.019)	-0.001 (0.028)	0.047 (0.032)	0.141 (0.08)	0.035 (0.053)	Body fat %
rs7570258	2	193791720	0,99	C	T	0.01 (0.002)	0.019 (0.018)	0.017 (0.027)	0.029 (0.03)	0.078 (0.077)	-0.029 (0.051)	Body fat %
rs2043016	2	198146381	0,97	T	C	0.011 (0.002)	-0.005 (0.019)	-0.038 (0.028)	0.033 (0.031)	0.112 (0.082)	-0.047 (0.054)	Body fat %
rs4482463	2	205375909	0,92	C	A	0.022 (0.003)	0.097 (0.034)	0.088 (0.053)	0.158 (0.054)	0.031 (0.138)	-0.04 (0.098)	Body fat %
rs2712169	2	217671349	0,99	G	A	0.011 (0.002)	0.011 (0.018)	0.01 (0.028)	0.019 (0.03)	0.022 (0.079)	-0.014 (0.052)	Body fat %
rs2943650	2	227105921	1,00	C	T	0.015 (0.002)	-0.038 (0.019)	-0.014 (0.028)	-0.064 (0.031)	-0.097 (0.082)	-0.02 (0.053)	Body fat %
rs4321353	2	229003882	0,99	T	G	0.014 (0.002)	0.039 (0.019)	0.04 (0.028)	-0.004 (0.031)	0.087 (0.081)	0.139 (0.054)	Body fat %
rs10498240	2	230734531	1,00	A	C	0.016 (0.002)	0.009 (0.019)	0.025 (0.028)	-0.024 (0.032)	0.093 (0.083)	0.009 (0.055)	Body fat %
rs62246314	3	9504099	0,99	A	G	0.017 (0.003)	0.014 (0.028)	0.078 (0.041)	-0.064 (0.048)	0.006 (0.122)	-0.008 (0.083)	Body fat %
rs4684847	3	12386337	1,00	T	C	0.03 (0.003)	-0.029 (0.026)	-0.031 (0.039)	-0.012 (0.044)	-0.028 (0.119)	-0.078 (0.079)	Body fat %
rs7649970	3	12392272	1,00	T	C	0.031 (0.003)	-0.03 (0.026)	-0.033 (0.039)	-0.01 (0.044)	-0.034 (0.119)	-0.083 (0.079)	Body fat %
rs4684848	3	12395645	1,00	A	G	0.03 (0.003)	-0.025 (0.026)	-0.032 (0.039)	0.002 (0.044)	-0.029 (0.118)	-0.082 (0.079)	Body fat %
rs4619804	3	18674644	0,98	C	A	0.013 (0.002)	0.048 (0.02)	0.007 (0.03)	0.06 (0.033)	0.183 (0.087)	0.107 (0.059)	Body fat %
rs13062093	3	35667057	1,00	G	T	0.012 (0.002)	0.011 (0.019)	0.005 (0.027)	-0.002 (0.031)	-0.01 (0.08)	0.083 (0.053)	Body fat %
rs1348252	3	42418752	0,98	C	T	0.012 (0.002)	0.02 (0.021)	0.008 (0.031)	0.032 (0.034)	0.131 (0.092)	-0.02 (0.06)	Body fat %
rs7637852	3	44041777	0,98	A	G	0.013 (0.002)	0.009 (0.02)	0.021 (0.029)	0.008 (0.033)	-0.137 (0.084)	0.032 (0.057)	Body fat %
rs62259939	3	49386047	0,97	A	G	0.013 (0.002)	-0.011 (0.019)	-0.012 (0.028)	-0.005 (0.031)	-0.026 (0.078)	-0.021 (0.051)	Body fat %
rs73079014	3	49863483	0,90	C	T	0.015 (0.003)	0.032 (0.03)	0.032 (0.044)	0.007 (0.048)	0.036 (0.13)	0.106 (0.083)	Body fat %
rs9843653	3	49920571	0,99	C	T	0.018 (0.002)	0.026 (0.018)	0.005 (0.027)	0.015 (0.03)	0.126 (0.078)	0.097 (0.052)	Body fat %
rs3774581	3	53802748	1,00	A	G	0.011 (0.002)	-0.011 (0.02)	-0.001 (0.029)	-0.01 (0.033)	-0.047 (0.085)	-0.037 (0.057)	Body fat %
rs17639546	3	61251635	1,00	G	A	0.014 (0.002)	-0.007 (0.026)	-0.005 (0.039)	-0.03 (0.044)	0.142 (0.114)	-0.014 (0.074)	Body fat %
rs9968060	3	62471282	1,00	T	C	0.012 (0.002)	0.008 (0.019)	0.008 (0.027)	0.025 (0.031)	0.006 (0.079)	-0.036 (0.053)	Body fat %
rs66815886	3	64703394	1,00	T	G	0.012 (0.002)	0.046 (0.02)	-0.014 (0.029)	0.102 (0.033)	0.06 (0.086)	0.097 (0.058)	Body fat %
rs2371767	3	64718258	0,99	C	G	0.013 (0.002)	0.048 (0.02)	-0.011 (0.03)	0.105 (0.033)	0.06 (0.086)	0.094 (0.058)	Body fat %
rs7630228	3	71681487	0,96	T	C	0.011 (0.002)	0.005 (0.018)	0.031 (0.027)	-0.007 (0.031)	0.001 (0.08)	-0.059 (0.054)	Body fat %
rs9856109	3	82563862	1,00	T	C	0.011 (0.002)	0.025 (0.019)	0.034 (0.029)	0.033 (0.031)	-0.008 (0.08)	-0.02 (0.054)	Body fat %
rs114712833	3	84139974	0,95	C	T	0.021 (0.004)	-0.009 (0.036)	0 (0.053)	-0.001 (0.06)	-0.101 (0.16)	-0.026 (0.098)	Body fat %
rs3911063	3	85906928	0,99	T	C	0.013 (0.002)	0.014 (0.02)	0.067 (0.029)	-0.029 (0.032)	0.073 (0.084)	-0.074 (0.055)	Body fat %
rs13062221	3	89778009	0,97	T	C	0.013 (0.002)	0.056 (0.019)	0.076 (0.029)	0.039 (0.03)	0.069 (0.08)	0.036 (0.052)	Body fat %
rs2198955	3	90345475	0,83	C	T	0.013 (0.002)	0.071 (0.021)	0.11 (0.032)	0.045 (0.032)	0.052 (0.089)	0.041 (0.057)	Body fat %
rs35714284	3	93529866	0,90	G	A	0.012 (0.002)	0.069 (0.02)	0.104 (0.031)	0.039 (0.032)	0.038 (0.086)	0.066 (0.054)	Body fat %
rs1609906	3	94033599	1,00	G	A	0.016 (0.002)	0.036 (0.018)	0.077 (0.027)	0.016 (0.03)	0.014 (0.079)	-0.048 (0.052)	Body fat %
rs10934646	3	123084541	0,99	A	G	0.013 (0.002)	0.025 (0.019)	0.029 (0.028)	0.022 (0.031)	0.024 (0.081)	0.017 (0.053)	Body fat %
rs9820766	3	123264017	0,99	C	T	0.012 (0.002)	0.039 (0.02)	0.038 (0.03)	0.06 (0.032)	0.086 (0.083)	-0.039 (0.056)	Body fat %
rs9816797	3	131614595	1,00	G	A	0.014 (0.002)	0.036 (0.02)	0.007 (0.031)	0.073 (0.034)	0.169 (0.087)	-0.027 (0.057)	Body fat %

rs7635592	3	131718029	1,00	T	C	0.016 (0.002)	-0.012 (0.022)	-0.014 (0.034)	0 (0.037)	-0.05 (0.096)	-0.027 (0.063)	Body fat %
rs2042864	3	141178979	0,98	C	T	0.012 (0.002)	0.038 (0.019)	0.036 (0.028)	0.024 (0.031)	-0.018 (0.078)	0.113 (0.053)	Body fat %
rs62271373	3	150066540	0,84	T	A	0.022 (0.004)	-0.064 (0.041)	-0.05 (0.065)	-0.074 (0.065)	-0.094 (0.158)	-0.059 (0.11)	Body fat %
rs1568489	3	153673681	1,00	G	A	0.012 (0.002)	-0.024 (0.018)	-0.041 (0.027)	0.003 (0.03)	-0.041 (0.078)	-0.041 (0.053)	Body fat %
rs8192675	3	170724883	1,00	C	T	0.011 (0.002)	0.017 (0.02)	0.029 (0.029)	0.025 (0.032)	-0.012 (0.083)	-0.033 (0.056)	Body fat %
rs12635614	3	173113041	0,99	A	G	0.01 (0.002)	0.039 (0.018)	0.037 (0.027)	0.029 (0.03)	0.082 (0.077)	0.053 (0.052)	Body fat %
rs2606228	3	183537759	0,97	A	C	0.012 (0.002)	0.024 (0.019)	0.037 (0.029)	0.001 (0.031)	0.102 (0.081)	0.013 (0.053)	Body fat %
rs9867130	3	185729753	0,79	G	A	0.016 (0.003)	-0.047 (0.028)	-0.05 (0.044)	-0.081 (0.043)	0.034 (0.114)	0.031 (0.076)	Body fat %
rs2192527	4	18329824	0,98	G	A	0.014 (0.002)	-0.018 (0.018)	-0.042 (0.027)	-0.015 (0.03)	0.092 (0.078)	0.008 (0.053)	Body fat %
rs28602597	4	20112947	0,97	A	G	0.019 (0.003)	0.017 (0.027)	0.024 (0.041)	0.067 (0.044)	-0.098 (0.117)	-0.106 (0.077)	Body fat %
rs73213501	4	28514830	0,98	A	C	0.015 (0.002)	0.026 (0.023)	0.057 (0.033)	-0.023 (0.037)	0.063 (0.097)	0.048 (0.068)	Body fat %
rs10938398	4	45186139	0,97	A	G	0.019 (0.002)	0.014 (0.018)	0.044 (0.028)	0.007 (0.03)	-0.132 (0.08)	-0.007 (0.052)	Body fat %
rs3761728	4	48990885	0,94	T	G	0.011 (0.002)	0.007 (0.021)	0.031 (0.032)	0.011 (0.033)	-0.104 (0.085)	-0.027 (0.056)	Body fat %
rs2102278	4	52818664	0,95	G	A	0.011 (0.002)	-0.003 (0.02)	0.035 (0.031)	-0.012 (0.033)	-0.126 (0.087)	-0.049 (0.056)	Body fat %
rs6840236	4	56289785	1,00	C	T	0.012 (0.002)	-0.005 (0.018)	0.024 (0.027)	-0.063 (0.03)	0.133 (0.077)	0.001 (0.052)	Body fat %
rs2318543	4	67803263	1,00	A	G	0.012 (0.002)	0.011 (0.021)	0.026 (0.031)	0.051 (0.036)	-0.176 (0.092)	-0.078 (0.061)	Body fat %
rs58125425	4	73550383	0,94	T	C	0.023 (0.004)	-0.006 (0.036)	-0.005 (0.051)	-0.036 (0.064)	0.018 (0.164)	0.064 (0.106)	Body fat %
rs12503232	4	80911468	0,98	A	C	0.013 (0.002)	-0.038 (0.02)	-0.066 (0.031)	-0.003 (0.033)	-0.094 (0.088)	-0.021 (0.058)	Body fat %
rs7692359	4	83209346	1,00	T	C	0.013 (0.002)	-0.01 (0.021)	-0.062 (0.031)	0.068 (0.036)	0.145 (0.096)	-0.104 (0.062)	Body fat %
rs3796658	4	89708241	1,00	G	A	0.012 (0.002)	-0.021 (0.018)	-0.032 (0.027)	0.024 (0.03)	-0.054 (0.077)	-0.104 (0.052)	Body fat %
rs1229984	4	100239319	0,79	C	T	0.035 (0.006)	0.025 (0.047)	-0.001 (0.076)	0.072 (0.072)	0.076 (0.197)	-0.095 (0.14)	Body fat %
rs13126505	4	102865304	0,76	A	G	0.03 (0.003)	-0.05 (0.041)	NA (NA)	0.061 (0.064)	-0.078 (0.161)	-0.13 (0.102)	Body fat %
rs13107325	4	103188709	0,97	T	C	0.037 (0.003)	-0.032 (0.035)	-0.048 (0.052)	-0.016 (0.058)	0.047 (0.146)	-0.053 (0.097)	Body fat %
rs11099020	4	130724902	0,98	C	T	0.011 (0.002)	0.002 (0.019)	0.016 (0.028)	0.028 (0.031)	-0.064 (0.08)	-0.101 (0.054)	Body fat %
rs1296328	4	137083193	0,95	A	C	0.01 (0.002)	0.009 (0.019)	-0.025 (0.028)	0.015 (0.03)	0.059 (0.081)	0.092 (0.053)	Body fat %
rs57800857	4	140863365	0,97	A	C	0.015 (0.002)	0.016 (0.019)	0.025 (0.028)	0.034 (0.032)	-0.084 (0.082)	-0.031 (0.055)	Body fat %
rs7720791	5	50361133	0,99	G	A	0.011 (0.002)	-0.03 (0.02)	-0.004 (0.03)	-0.073 (0.032)	-0.008 (0.085)	0.002 (0.056)	Body fat %
rs6861649	5	50864788	0,99	C	T	0.011 (0.002)	-0.034 (0.018)	-0.007 (0.027)	-0.038 (0.031)	-0.05 (0.08)	-0.112 (0.053)	Body fat %
rs157845	5	55796639	0,96	T	C	0.012 (0.002)	0.02 (0.021)	0.044 (0.031)	0.032 (0.034)	-0.095 (0.089)	-0.05 (0.06)	Body fat %
rs7736910	5	63040773	0,97	G	A	0.012 (0.002)	0.007 (0.018)	-0.008 (0.027)	0.022 (0.03)	-0.013 (0.077)	0.029 (0.052)	Body fat %
rs6893495	5	64409378	1,00	T	C	0.013 (0.002)	-0.002 (0.022)	-0.05 (0.033)	0.041 (0.037)	0.094 (0.098)	0.01 (0.062)	Body fat %
rs249612	5	66200783	1,00	T	C	0.012 (0.002)	0.023 (0.02)	0.024 (0.031)	0 (0.034)	0.033 (0.087)	0.082 (0.057)	Body fat %
rs4976033	5	67714246	0,91	A	G	0.012 (0.002)	0.014 (0.019)	0.038 (0.029)	0.031 (0.031)	-0.148 (0.084)	-0.055 (0.052)	Body fat %
rs4704187	5	74480288	0,92	T	C	0.014 (0.002)	0.004 (0.019)	0.003 (0.029)	0.011 (0.03)	-0.056 (0.08)	0.013 (0.052)	Body fat %
rs13356670	5	74673707	0,89	A	G	0.018 (0.002)	0.031 (0.022)	-0.018 (0.034)	0.061 (0.036)	0.018 (0.096)	0.102 (0.061)	Body fat %
rs2307111	5	75003678	1,00	T	C	0.017 (0.002)	0.022 (0.018)	0.021 (0.027)	0.037 (0.03)	-0.046 (0.079)	0.013 (0.053)	Body fat %
rs59893724	5	80830788	0,98	A	G	0.013 (0.002)	-0.022 (0.022)	-0.024 (0.032)	-0.012 (0.036)	-0.026 (0.094)	-0.04 (0.06)	Body fat %
rs34580448	5	82810884	0,90	T	C	0.032 (0.004)	-0.016 (0.046)	-0.03 (0.068)	-0.058 (0.075)	-0.146 (0.198)	0.228 (0.133)	Body fat %
rs11951885	5	86727566	0,97	C	T	0.036 (0.006)	0.014 (0.055)	0.033 (0.079)	-0.065 (0.092)	0.044 (0.231)	0.194 (0.175)	Body fat %
rs6870983	5	87697533	0,99	C	T	0.014 (0.002)	0.004 (0.022)	0.005 (0.032)	0.01 (0.036)	0.064 (0.093)	-0.048 (0.064)	Body fat %
rs34483452	5	87986314	0,99	A	C	0.024 (0.003)	0.074 (0.026)	0.052 (0.037)	0.12 (0.043)	0.125 (0.112)	-0.005 (0.075)	Body fat %
rs59399491	5	92560816	0,99	G	A	0.013 (0.002)	0.013 (0.022)	0.019 (0.033)	0.042 (0.037)	-0.012 (0.094)	-0.088 (0.064)	Body fat %
rs11135450	5	95554016	0,99	G	A	0.011 (0.002)	0.013 (0.019)	-0.006 (0.028)	0.016 (0.031)	-0.034 (0.085)	0.096 (0.055)	Body fat %
rs254024	5	103944020	1,00	T	G	0.01 (0.002)	-0.004 (0.018)	-0.021 (0.027)	0.025 (0.03)	-0.039 (0.076)	-0.01 (0.052)	Body fat %
rs40067	5	107439012	1,00	G	A	0.017 (0.002)	0.038 (0.024)	0.034 (0.036)	0.052 (0.039)	0.091 (0.105)	-0.014 (0.069)	Body fat %
rs4502882	5	153093998	1,00	C	T	0.012 (0.002)	0.004 (0.019)	-0.01 (0.028)	0.024 (0.032)	0.068 (0.082)	-0.033 (0.055)	Body fat %
rs2964481	5	157892530	0,99	T	C	0.015 (0.002)	0.004 (0.02)	-0.013 (0.03)	0.016 (0.034)	-0.114 (0.088)	0.081 (0.058)	Body fat %
rs245775	5	170532105	0,98	G	A	0.013 (0.002)	-0.006 (0.02)	-0.021 (0.03)	-0.015 (0.033)	-0.022 (0.088)	0.084 (0.059)	Body fat %
rs10947793	6	12142817	0,95	A	G	0.012 (0.002)	0.007 (0.019)	-0.003 (0.028)	0.003 (0.032)	-0.064 (0.08)	0.088 (0.054)	Body fat %

rs1042317	6	20492995	0,98	T	C	0.011 (0.002)	-0.017 (0.02)	-0.05 (0.03)	0.002 (0.033)	-0.061 (0.085)	0.059 (0.055)	Body fat %
rs75499503	6	26145217	0,95	C	T	0.025 (0.002)	-0.006 (0.022)	-0.019 (0.031)	0.085 (0.036)	-0.241 (0.097)	-0.138 (0.065)	Body fat %
rs7766641	6	26184102	0,99	G	A	0.024 (0.002)	-0.008 (0.02)	0.021 (0.029)	0.008 (0.033)	-0.026 (0.088)	-0.179 (0.06)	Body fat %
rs12202849	6	26704972	0,89	G	A	0.017 (0.002)	0.013 (0.022)	0.062 (0.034)	0.006 (0.034)	0.026 (0.09)	-0.125 (0.06)	Body fat %
rs72843644	6	27211601	0,99	A	C	0.021 (0.003)	0.018 (0.031)	0.051 (0.047)	0.05 (0.051)	0.019 (0.136)	-0.191 (0.088)	Body fat %
rs6902687	6	28413491	1,00	C	T	0.01 (0.002)	-0.018 (0.02)	0 (0.031)	0.004 (0.032)	0.071 (0.082)	-0.17 (0.053)	Body fat %
rs62395827	6	31786730	0,97	C	T	0.023 (0.003)	-0.014 (0.033)	-0.058 (0.05)	-0.008 (0.054)	0.237 (0.141)	0.004 (0.085)	Body fat %
rs1061801	6	33282338	1,00	A	G	0.014 (0.002)	-0.008 (0.023)	-0.055 (0.034)	0.033 (0.038)	-0.044 (0.099)	0.061 (0.065)	Body fat %
rs59137082	6	33732365	1,00	C	T	0.012 (0.002)	0.034 (0.021)	0.016 (0.03)	0.033 (0.034)	0.312 (0.09)	-0.014 (0.06)	Body fat %
rs2744956	6	34618937	1,00	C	T	0.025 (0.003)	0.065 (0.025)	-0.025 (0.038)	0.146 (0.041)	0.158 (0.106)	0.107 (0.073)	Body fat %
rs9469887	6	34758940	0,99	T	C	0.021 (0.002)	0.028 (0.019)	-0.019 (0.028)	0.075 (0.032)	0.064 (0.082)	0.043 (0.055)	Body fat %
rs1051952	6	35465786	1,00	A	C	0.013 (0.002)	0.028 (0.018)	0.031 (0.027)	0.002 (0.03)	0.14 (0.077)	0.04 (0.051)	Body fat %
rs2064317	6	35477032	0,99	A	G	0.015 (0.002)	0.02 (0.019)	0.027 (0.028)	-0.01 (0.031)	0.156 (0.081)	0.022 (0.053)	Body fat %
rs9471333	6	40362023	1,00	C	T	0.016 (0.002)	-0.003 (0.018)	0.003 (0.027)	-0.008 (0.03)	-0.004 (0.078)	-0.012 (0.051)	Body fat %
rs73737608	6	50376763	0,98	G	A	0.023 (0.004)	0.061 (0.038)	0.084 (0.053)	0.079 (0.066)	-0.247 (0.17)	0.04 (0.114)	Body fat %
rs4715208	6	50829471	1,00	G	A	0.017 (0.002)	-0.003 (0.021)	-0.01 (0.031)	0.029 (0.034)	0.053 (0.092)	-0.095 (0.059)	Body fat %
rs1928185	6	50935513	0,98	C	T	0.023 (0.002)	0.048 (0.023)	0.048 (0.034)	0.088 (0.039)	-0.117 (0.101)	-0.004 (0.069)	Body fat %
rs1414506	6	51487416	0,99	T	C	0.011 (0.002)	-0.016 (0.019)	-0.047 (0.028)	0.039 (0.031)	-0.018 (0.08)	-0.065 (0.054)	Body fat %
rs1884953	6	51798760	0,82	A	T	0.014 (0.002)	0.051 (0.026)	0.06 (0.037)	0.066 (0.044)	-0.201 (0.115)	0.081 (0.076)	Body fat %
rs13191298	6	70010225	1,00	G	A	0.016 (0.003)	0.027 (0.027)	-0.008 (0.041)	0.075 (0.045)	0.154 (0.114)	-0.054 (0.079)	Body fat %
rs62422090	6	97944199	0,96	A	G	0.012 (0.002)	0.001 (0.021)	0.008 (0.031)	-0.002 (0.034)	0.058 (0.086)	-0.037 (0.059)	Body fat %
rs10499014	6	97947755	0,95	C	G	0.012 (0.002)	0.002 (0.021)	0.011 (0.032)	-0.002 (0.034)	0.055 (0.087)	-0.04 (0.059)	Body fat %
rs9320823	6	98429337	0,99	C	T	0.017 (0.002)	0.015 (0.019)	0.006 (0.028)	0.001 (0.03)	-0.011 (0.078)	0.099 (0.053)	Body fat %
rs9375188	6	98555272	1,00	C	T	0.017 (0.002)	0.003 (0.018)	-0.001 (0.027)	0.001 (0.03)	-0.002 (0.078)	0.031 (0.051)	Body fat %
rs314279	6	105402083	0,95	C	A	0.016 (0.003)	-0.026 (0.028)	-0.034 (0.041)	-0.036 (0.046)	0.232 (0.123)	-0.073 (0.079)	Body fat %
rs6927268	6	108865663	0,99	T	G	0.014 (0.002)	0.015 (0.022)	0.002 (0.031)	0.066 (0.036)	0.05 (0.093)	-0.105 (0.064)	Body fat %
rs9400479	6	111826959	0,99	G	T	0.014 (0.002)	0.015 (0.023)	-0.014 (0.034)	0.025 (0.038)	-0.066 (0.1)	0.135 (0.067)	Body fat %
rs9481206	6	112233412	1,00	A	T	0.018 (0.003)	-0.019 (0.03)	0.006 (0.046)	-0.028 (0.048)	0.001 (0.121)	-0.083 (0.082)	Body fat %
rs111743285	6	127048230	0,98	T	C	0.016 (0.002)	0.005 (0.022)	0.003 (0.032)	0.012 (0.037)	-0.012 (0.094)	0.005 (0.062)	Body fat %
rs72959041	6	127454893	0,72	G	A	0.025 (0.004)	0.062 (0.047)	NA (NA)	0.19 (0.074)	-0.051 (0.188)	-0.142 (0.128)	Body fat %
rs9321191	6	130165691	1,00	T	C	0.013 (0.002)	0.013 (0.022)	-0.025 (0.033)	0.068 (0.037)	-0.161 (0.099)	0.065 (0.064)	Body fat %
rs72995085	6	143193971	0,99	T	C	0.014 (0.002)	-0.017 (0.023)	-0.037 (0.035)	0.02 (0.039)	-0.157 (0.1)	0.012 (0.066)	Body fat %
rs4709745	6	164105984	1,00	C	T	0.011 (0.002)	0 (0.02)	0.036 (0.029)	-0.022 (0.032)	-0.086 (0.084)	-0.034 (0.056)	Body fat %
rs2529050	7	24595823	0,99	C	T	0.011 (0.002)	0.056 (0.02)	0.051 (0.03)	0.085 (0.033)	0.06 (0.087)	-0.011 (0.057)	Body fat %
rs10259620	7	27202289	1,00	A	G	0.016 (0.002)	-0.015 (0.022)	-0.008 (0.031)	-0.048 (0.036)	-0.034 (0.094)	0.063 (0.062)	Body fat %
rs10264581	7	27255417	0,99	G	A	0.025 (0.004)	-0.047 (0.036)	-0.019 (0.052)	-0.056 (0.061)	-0.053 (0.156)	-0.138 (0.108)	Body fat %
rs215669	7	32378979	0,99	G	A	0.011 (0.002)	0.034 (0.019)	0.007 (0.028)	0.066 (0.03)	0.106 (0.079)	0.005 (0.053)	Body fat %
rs4549685	7	39326478	1,00	C	T	0.013 (0.002)	0 (0.019)	0.008 (0.029)	0.01 (0.031)	-0.107 (0.082)	-0.016 (0.055)	Body fat %
rs2289379	7	44804225	0,97	C	T	0.012 (0.002)	0.011 (0.019)	0.011 (0.028)	-0.01 (0.031)	0.07 (0.079)	0.046 (0.052)	Body fat %
rs6948959	7	50697051	0,99	G	A	0.012 (0.002)	-0.038 (0.021)	0.001 (0.032)	-0.103 (0.035)	-0.065 (0.088)	0.03 (0.058)	Body fat %
rs4718964	7	70038969	0,99	T	G	0.012 (0.002)	-0.01 (0.018)	-0.02 (0.027)	0.009 (0.03)	-0.026 (0.078)	-0.02 (0.052)	Body fat %
rs62477684	7	75101427	0,89	C	T	0.013 (0.002)	0.008 (0.019)	-0.032 (0.028)	0.03 (0.032)	0.006 (0.078)	0.102 (0.056)	Body fat %
rs17704028	7	95140031	0,99	C	T	0.015 (0.002)	-0.01 (0.026)	-0.053 (0.039)	0.041 (0.041)	-0.065 (0.116)	0.006 (0.074)	Body fat %
rs3901286	7	99107727	0,98	C	A	0.016 (0.002)	0.033 (0.025)	0.076 (0.036)	0.011 (0.041)	-0.224 (0.115)	0.039 (0.072)	Body fat %
rs6946860	7	112953475	0,98	A	G	0.013 (0.002)	0.013 (0.019)	0.041 (0.028)	-0.02 (0.031)	-0.061 (0.08)	0.045 (0.054)	Body fat %
rs80172389	7	114432036	0,90	G	T	0.018 (0.003)	-0.005 (0.032)	-0.045 (0.049)	0.018 (0.051)	-0.044 (0.13)	0.076 (0.09)	Body fat %
rs972283	7	130466854	0,98	A	G	0.013 (0.002)	-0.006 (0.018)	0.008 (0.028)	-0.03 (0.03)	0.016 (0.078)	0.006 (0.052)	Body fat %
rs6977416	7	150542711	0,99	G	A	0.012 (0.002)	-0.034 (0.019)	-0.022 (0.028)	-0.028 (0.031)	-0.069 (0.081)	-0.086 (0.054)	Body fat %
rs10245306	7	158029340	0,90	C	G	0.011 (0.002)	-0.028 (0.021)	0.011 (0.033)	-0.059 (0.033)	-0.061 (0.082)	-0.035 (0.056)	Body fat %

rs11782341	8	4813459	1,00	G	A	0.014 (0.002)	0.02 (0.023)	0.017 (0.034)	0.02 (0.038)	0.017 (0.098)	0.036 (0.067)	Body fat %
rs13268133	8	9731470	0,99	T	C	0.012 (0.002)	0.038 (0.02)	0.088 (0.03)	-0.02 (0.031)	0.098 (0.083)	0.021 (0.055)	Body fat %
rs11786089	8	21975521	0,98	G	A	0.012 (0.002)	0.028 (0.018)	0.026 (0.027)	0.022 (0.03)	0.089 (0.078)	0.028 (0.051)	Body fat %
rs11781222	8	23389571	0,98	T	C	0.017 (0.003)	0.011 (0.027)	0.023 (0.041)	-0.016 (0.045)	0.016 (0.108)	0.048 (0.076)	Body fat %
rs59104534	8	25666169	0,97	T	C	0.011 (0.002)	-0.006 (0.02)	0.013 (0.03)	0.02 (0.033)	-0.076 (0.086)	-0.121 (0.057)	Body fat %
rs2725370	8	30852826	1,00	T	C	0.013 (0.002)	0.013 (0.02)	0.003 (0.029)	-0.004 (0.033)	0.176 (0.085)	0.025 (0.056)	Body fat %
rs1808629	8	73435964	1,00	G	A	0.016 (0.002)	0.013 (0.019)	0.016 (0.029)	0.019 (0.032)	-0.059 (0.084)	0.012 (0.055)	Body fat %
rs2977345	8	76725867	1,00	C	T	0.015 (0.002)	0.05 (0.02)	0.039 (0.029)	0.054 (0.033)	0.06 (0.085)	0.073 (0.056)	Body fat %
rs10100245	8	77226919	1,00	A	G	0.015 (0.002)	0.049 (0.018)	0.089 (0.027)	-0.004 (0.03)	-0.01 (0.081)	0.08 (0.052)	Body fat %
rs879256	8	78893942	0,99	A	G	0.011 (0.002)	0.018 (0.019)	0.008 (0.028)	0.011 (0.031)	0.088 (0.081)	0.048 (0.054)	Body fat %
rs2721963	8	116661174	1,00	A	C	0.014 (0.002)	-0.008 (0.019)	-0.038 (0.029)	0.019 (0.031)	-0.045 (0.082)	0.034 (0.054)	Body fat %
rs4876611	8	116671848	1,00	G	A	0.016 (0.002)	-0.01 (0.02)	-0.059 (0.031)	0.036 (0.033)	-0.066 (0.086)	0.041 (0.057)	Body fat %
rs4466418	8	126323787	0,99	A	G	0.011 (0.002)	0.014 (0.018)	0.04 (0.027)	-0.012 (0.03)	0.019 (0.077)	-0.011 (0.051)	Body fat %
rs2954033	8	126493746	0,99	G	A	0.011 (0.002)	-0.006 (0.02)	-0.003 (0.029)	-0.004 (0.032)	-0.124 (0.084)	0.03 (0.055)	Body fat %
rs10959841	9	11469190	1,00	T	C	0.011 (0.002)	0.065 (0.018)	0.063 (0.028)	0.058 (0.03)	-0.017 (0.078)	0.129 (0.052)	Body fat %
rs7046483	9	14777395	1,00	G	A	0.01 (0.002)	0.036 (0.018)	0.017 (0.027)	0.051 (0.031)	0.077 (0.08)	0.044 (0.052)	Body fat %
rs10962016	9	15389970	0,96	G	A	0.024 (0.004)	-0.016 (0.041)	-0.083 (0.063)	0.087 (0.066)	-0.026 (0.172)	-0.098 (0.118)	Body fat %
rs13292699	9	15910044	0,99	A	C	0.021 (0.002)	0.023 (0.018)	-0.015 (0.028)	0.08 (0.03)	0.205 (0.079)	-0.09 (0.052)	Body fat %
rs1415475	9	16591502	0,98	T	C	0.024 (0.004)	0.04 (0.037)	0.003 (0.055)	0.117 (0.061)	0.115 (0.154)	-0.089 (0.106)	Body fat %
rs10756798	9	16739763	0,97	C	T	0.014 (0.002)	-0.016 (0.019)	-0.001 (0.028)	0.003 (0.031)	-0.077 (0.08)	-0.102 (0.055)	Body fat %
rs17770336	9	28414625	1,00	T	C	0.015 (0.002)	-0.005 (0.019)	-0.001 (0.028)	-0.008 (0.032)	-0.015 (0.083)	-0.006 (0.054)	Body fat %
rs7848702	9	31210051	0,99	T	C	0.012 (0.002)	0.018 (0.021)	-0.002 (0.031)	-0.001 (0.034)	0.162 (0.088)	0.085 (0.058)	Body fat %
rs10973160	9	36994969	0,92	C	T	0.011 (0.002)	0.023 (0.021)	0 (0.031)	0.044 (0.034)	0.066 (0.089)	0.019 (0.058)	Body fat %
rs12339822	9	92187178	0,98	G	A	0.014 (0.002)	0.048 (0.018)	0.071 (0.027)	0 (0.03)	0.053 (0.078)	0.111 (0.052)	Body fat %
rs10820739	9	99263821	1,00	A	G	0.013 (0.002)	0.002 (0.023)	-0.024 (0.035)	0.033 (0.038)	0.039 (0.098)	-0.016 (0.063)	Body fat %
rs11790018	9	129702842	0,94	C	G	0.01 (0.002)	-0.01 (0.019)	-0.028 (0.028)	0.014 (0.031)	0.004 (0.081)	-0.023 (0.056)	Body fat %
rs11012732	10	21830104	0,98	G	A	0.018 (0.002)	0.046 (0.019)	0.045 (0.028)	0.054 (0.032)	0.162 (0.083)	-0.02 (0.054)	Body fat %
rs7078183	10	70367452	0,95	C	A	0.01 (0.002)	0.03 (0.019)	0.046 (0.029)	0.032 (0.031)	-0.059 (0.081)	0.011 (0.052)	Body fat %
rs10999456	10	72413827	1,00	T	C	0.016 (0.002)	-0.011 (0.021)	-0.033 (0.031)	0.004 (0.034)	-0.003 (0.09)	0.016 (0.057)	Body fat %
rs10999460	10	72428283	1,00	T	C	0.016 (0.002)	-0.004 (0.021)	-0.028 (0.031)	0.013 (0.034)	0.006 (0.09)	0.024 (0.057)	Body fat %
rs2002023	10	76848524	0,99	T	C	0.012 (0.002)	-0.01 (0.018)	-0.005 (0.027)	0.002 (0.03)	-0.126 (0.079)	-0.016 (0.052)	Body fat %
rs11594905	10	77659733	0,75	A	G	0.015 (0.003)	0.001 (0.032)	NA (NA)	0.018 (0.049)	-0.003 (0.122)	-0.026 (0.081)	Body fat %
rs10887582	10	88110792	0,99	C	T	0.01 (0.002)	0.01 (0.018)	0.006 (0.027)	0.035 (0.03)	0.006 (0.077)	-0.046 (0.052)	Body fat %
rs11187838	10	96038686	0,99	G	A	0.014 (0.002)	0.053 (0.018)	0.039 (0.027)	0.071 (0.03)	0.024 (0.078)	0.064 (0.052)	Body fat %
rs4110517	10	96650328	0,99	G	A	0.012 (0.002)	0.03 (0.022)	0.022 (0.033)	0.091 (0.036)	-0.095 (0.094)	-0.072 (0.064)	Body fat %
rs577525	10	99769388	1,00	C	T	0.014 (0.002)	0.035 (0.018)	0.074 (0.027)	-0.029 (0.03)	-0.035 (0.078)	0.113 (0.052)	Body fat %
rs41310284	10	102447647	0,91	C	A	0.018 (0.003)	0.01 (0.033)	0.072 (0.048)	-0.022 (0.054)	-0.231 (0.14)	-0.034 (0.104)	Body fat %
rs6585201	10	114768783	0,99	G	A	0.011 (0.002)	0.02 (0.018)	0.014 (0.027)	0.034 (0.03)	-0.03 (0.078)	0.024 (0.052)	Body fat %
rs1225404	10	114914665	0,99	C	T	0.011 (0.002)	0.016 (0.019)	0.022 (0.028)	0.026 (0.031)	0.026 (0.081)	-0.038 (0.053)	Body fat %
rs10510025	10	118650996	1,00	T	C	0.013 (0.002)	-0.005 (0.021)	0.001 (0.03)	-0.028 (0.034)	0.057 (0.09)	0.018 (0.06)	Body fat %
rs4752182	10	120397131	0,99	A	G	0.01 (0.002)	0.006 (0.018)	0.006 (0.027)	0.021 (0.03)	0.009 (0.08)	-0.04 (0.052)	Body fat %
rs12218858	10	126474200	0,99	T	C	0.01 (0.002)	-0.011 (0.018)	-0.005 (0.027)	-0.036 (0.03)	0.075 (0.079)	0.009 (0.051)	Body fat %
rs2172131	10	133978962	0,94	T	C	0.011 (0.002)	0.009 (0.019)	-0.001 (0.028)	0.039 (0.03)	-0.083 (0.078)	-0.003 (0.052)	Body fat %
rs11042030	11	8690718	0,99	T	C	0.013 (0.002)	0.056 (0.02)	0.098 (0.029)	0.055 (0.033)	0.037 (0.087)	-0.101 (0.058)	Body fat %
rs11042725	11	10325325	0,99	A	C	0.011 (0.002)	-0.003 (0.018)	-0.006 (0.027)	0.016 (0.03)	0.042 (0.08)	-0.072 (0.052)	Body fat %
rs10766077	11	13349781	0,99	G	A	0.012 (0.002)	0.048 (0.019)	0.035 (0.028)	0.029 (0.03)	0.092 (0.08)	0.132 (0.053)	Body fat %
rs10767659	11	27686196	0,99	G	T	0.02 (0.002)	-0.024 (0.02)	-0.077 (0.029)	0 (0.032)	0.007 (0.082)	0.075 (0.054)	Body fat %
rs11030108	11	27695464	1,00	A	G	0.021 (0.002)	-0.031 (0.02)	-0.081 (0.03)	-0.013 (0.032)	-0.01 (0.083)	0.078 (0.055)	Body fat %
rs59227842	11	43692423	0,97	G	A	0.017 (0.002)	0.014 (0.02)	-0.023 (0.03)	0.032 (0.032)	0.097 (0.085)	0.054 (0.056)	Body fat %

rs71474196	11	46977160	0,93	C	T	0.016 (0.003)	-0.003 (0.029)	-0.029 (0.043)	0.027 (0.048)	0.098 (0.124)	-0.036 (0.079)	Body fat %
rs7124681	11	47529947	0,99	A	C	0.022 (0.002)	-0.007 (0.018)	-0.006 (0.028)	-0.012 (0.03)	0.001 (0.078)	0 (0.053)	Body fat %
rs12146571	11	48314077	0,74	C	T	0.014 (0.002)	0.001 (0.026)	NA (NA)	0.011 (0.041)	0.084 (0.109)	0.032 (0.068)	Body fat %
rs477895	11	64048912	1,00	T	C	0.015 (0.002)	0.001 (0.025)	0.024 (0.038)	0.012 (0.041)	-0.081 (0.107)	-0.068 (0.069)	Body fat %
rs801742	11	65914766	0,99	C	A	0.015 (0.002)	0.033 (0.019)	0.033 (0.029)	-0.006 (0.031)	0.142 (0.083)	0.103 (0.054)	Body fat %
rs1213257	11	85220773	0,99	C	T	0.014 (0.002)	-0.026 (0.024)	-0.035 (0.036)	-0.046 (0.04)	-0.017 (0.106)	0.065 (0.069)	Body fat %
rs61903695	11	89922417	1,00	G	A	0.012 (0.002)	0.034 (0.021)	0.029 (0.032)	0.063 (0.035)	-0.068 (0.092)	0.012 (0.058)	Body fat %
rs3802851	11	112912550	1,00	T	C	0.012 (0.002)	0.017 (0.02)	0.021 (0.029)	0.02 (0.034)	-0.149 (0.088)	0.064 (0.059)	Body fat %
rs719802	11	113234679	1,00	T	C	0.011 (0.002)	-0.021 (0.019)	-0.062 (0.028)	0.019 (0.03)	0.146 (0.081)	-0.067 (0.052)	Body fat %
rs4545564	11	118937518	0,98	C	T	0.011 (0.002)	-0.006 (0.018)	-0.007 (0.027)	0.006 (0.03)	-0.086 (0.08)	-0.001 (0.052)	Body fat %
rs10791109	11	130850377	0,99	G	T	0.011 (0.002)	-0.007 (0.018)	0.011 (0.027)	-0.006 (0.03)	0.123 (0.077)	-0.132 (0.051)	Body fat %
rs73041988	11	134517234	1,00	T	G	0.017 (0.002)	0.017 (0.025)	0.03 (0.037)	0.004 (0.041)	0.006 (0.107)	0.017 (0.07)	Body fat %
rs55726687	12	991306	0,94	A	G	0.014 (0.002)	0.014 (0.023)	-0.017 (0.034)	-0.01 (0.037)	0.059 (0.101)	0.181 (0.064)	Body fat %
rs765123	12	2155997	0,95	G	A	0.012 (0.002)	0.039 (0.022)	0.01 (0.033)	0.069 (0.038)	-0.052 (0.097)	0.111 (0.066)	Body fat %
rs7296615	12	3350679	0,99	A	G	0.021 (0.003)	-0.01 (0.031)	-0.026 (0.046)	0.031 (0.051)	0.046 (0.136)	-0.105 (0.093)	Body fat %
rs12367809	12	50256063	0,97	T	C	0.019 (0.002)	-0.009 (0.019)	-0.021 (0.028)	0.019 (0.031)	-0.108 (0.08)	-0.002 (0.054)	Body fat %
rs7132908	12	50263148	0,99	A	G	0.019 (0.002)	-0.008 (0.018)	-0.027 (0.027)	0.032 (0.03)	-0.091 (0.079)	-0.02 (0.052)	Body fat %
rs4759318	12	54420098	0,99	T	C	0.011 (0.002)	-0.031 (0.019)	-0.033 (0.028)	-0.038 (0.031)	-0.053 (0.081)	0.004 (0.054)	Body fat %
rs704061	12	89771903	1,00	C	T	0.014 (0.002)	-0.008 (0.018)	0.002 (0.027)	-0.009 (0.03)	0.072 (0.076)	-0.079 (0.051)	Body fat %
rs12813149	12	90142637	0,98	G	A	0.015 (0.002)	0.011 (0.021)	0.017 (0.031)	-0.018 (0.034)	0.062 (0.089)	0.051 (0.058)	Body fat %
rs7975788	12	90273927	1,00	G	T	0.012 (0.002)	-0.007 (0.02)	-0.048 (0.03)	0.02 (0.033)	0.012 (0.085)	0.054 (0.058)	Body fat %
rs10777259	12	91248014	0,98	C	T	0.01 (0.002)	-0.009 (0.018)	-0.003 (0.027)	-0.009 (0.03)	-0.057 (0.078)	-0.012 (0.051)	Body fat %
rs59066241	12	97925364	0,96	G	T	0.016 (0.003)	0.003 (0.027)	0.051 (0.039)	-0.039 (0.045)	-0.196 (0.124)	0.016 (0.08)	Body fat %
rs11113445	12	108088682	1,00	G	A	0.012 (0.002)	-0.001 (0.018)	-0.012 (0.027)	0.018 (0.03)	-0.041 (0.079)	0.001 (0.052)	Body fat %
rs11609659	12	108296260	0,99	T	C	0.014 (0.002)	-0.017 (0.021)	-0.064 (0.031)	0.006 (0.035)	0.038 (0.093)	0.068 (0.061)	Body fat %
rs3764002	12	108618630	1,00	C	T	0.016 (0.002)	-0.006 (0.02)	-0.015 (0.029)	-0.007 (0.034)	-0.055 (0.087)	0.055 (0.058)	Body fat %
rs10492229	12	110602173	1,00	T	C	0.012 (0.002)	-0.036 (0.022)	-0.04 (0.033)	-0.063 (0.036)	0.119 (0.093)	-0.014 (0.06)	Body fat %
rs61945850	12	120846213	0,86	G	A	0.032 (0.005)	-0.056 (0.053)	-0.106 (0.078)	-0.008 (0.089)	0.158 (0.24)	-0.089 (0.142)	Body fat %
rs75412871	12	121709430	0,88	C	T	0.025 (0.004)	-0.026 (0.043)	-0.039 (0.068)	-0.04 (0.068)	0.259 (0.175)	-0.067 (0.114)	Body fat %
rs77234932	12	122472678	0,84	C	T	0.015 (0.002)	-0.047 (0.023)	-0.049 (0.034)	0.006 (0.037)	-0.07 (0.099)	-0.196 (0.066)	Body fat %
rs35249105	12	122507810	0,97	G	A	0.011 (0.002)	-0.007 (0.018)	-0.03 (0.028)	0.024 (0.03)	0.115 (0.079)	-0.068 (0.052)	Body fat %
rs147730268	12	123024476	0,82	G	T	0.032 (0.003)	-0.044 (0.035)	-0.001 (0.053)	-0.005 (0.056)	-0.12 (0.159)	-0.245 (0.092)	Body fat %
rs10773394	12	123124778	0,98	A	C	0.013 (0.002)	-0.043 (0.02)	-0.026 (0.03)	-0.028 (0.032)	-0.161 (0.083)	-0.096 (0.056)	Body fat %
rs7133378	12	124409502	0,98	A	G	0.019 (0.002)	-0.017 (0.02)	-0.019 (0.029)	-0.008 (0.032)	0.001 (0.084)	-0.044 (0.055)	Body fat %
rs825452	12	124509177	1,00	G	A	0.015 (0.002)	-0.01 (0.018)	-0.017 (0.027)	-0.014 (0.03)	-0.004 (0.079)	0.027 (0.052)	Body fat %
rs11619393	13	20262266	0,96	C	T	0.015 (0.002)	0.017 (0.027)	0.028 (0.041)	-0.012 (0.043)	0.036 (0.111)	0.062 (0.074)	Body fat %
rs1928496	13	31012904	1,00	T	C	0.012 (0.002)	0.019 (0.021)	0.032 (0.03)	-0.005 (0.034)	0.13 (0.092)	-0.004 (0.058)	Body fat %
rs9568867	13	54107352	1,00	A	G	0.017 (0.003)	0.048 (0.027)	0.06 (0.041)	0.055 (0.044)	-0.102 (0.116)	0.056 (0.076)	Body fat %
rs7982447	13	54453811	1,00	C	T	0.015 (0.002)	-0.008 (0.022)	-0.053 (0.033)	0.037 (0.037)	0.033 (0.095)	0 (0.062)	Body fat %
rs7319102	13	58252801	0,99	G	A	0.013 (0.002)	-0.036 (0.022)	-0.038 (0.034)	-0.045 (0.036)	0.054 (0.091)	-0.045 (0.062)	Body fat %
rs11839227	13	59280705	1,00	T	C	0.014 (0.002)	0.017 (0.023)	-0.022 (0.035)	0.017 (0.039)	0.167 (0.102)	0.096 (0.067)	Body fat %
rs1218307	13	79417832	0,99	G	A	0.011 (0.002)	0.004 (0.018)	0.011 (0.027)	-0.017 (0.03)	0.07 (0.077)	0.014 (0.052)	Body fat %
rs1441264	13	79580919	1,00	A	G	0.012 (0.002)	-0.01 (0.018)	-0.013 (0.027)	-0.008 (0.03)	-0.11 (0.08)	0.035 (0.052)	Body fat %
rs72632799	13	86493418	0,92	G	A	0.013 (0.002)	-0.003 (0.022)	-0.02 (0.033)	0.02 (0.036)	-0.051 (0.096)	0.01 (0.061)	Body fat %
rs6491427	13	99113166	0,98	A	G	0.015 (0.002)	0.015 (0.02)	0.007 (0.029)	0.038 (0.033)	0.042 (0.085)	-0.039 (0.057)	Body fat %
rs35413307	13	112191778	0,97	G	T	0.012 (0.002)	0.012 (0.019)	-0.006 (0.028)	0.023 (0.03)	-0.04 (0.079)	0.069 (0.052)	Body fat %
rs9522279	13	112221296	0,99	T	C	0.013 (0.002)	0.022 (0.018)	0.028 (0.027)	0.034 (0.03)	-0.106 (0.078)	0.028 (0.052)	Body fat %
rs4981693	14	29680331	0,99	A	G	0.014 (0.002)	0.037 (0.021)	0.07 (0.032)	0.02 (0.035)	0.091 (0.093)	-0.065 (0.062)	Body fat %
rs1959430	14	30184162	0,99	C	T	0.01 (0.002)	-0.019 (0.018)	-0.049 (0.027)	0.029 (0.03)	-0.049 (0.078)	-0.042 (0.052)	Body fat %

rs61979560	14	30727033	0,84	A	C	0.012 (0.002)	0.042 (0.022)	0.022 (0.032)	0.037 (0.036)	0.083 (0.094)	0.116 (0.062)	Body fat %
rs2239647	14	33292743	1,00	A	C	0.013 (0.002)	0.025 (0.018)	0.035 (0.027)	0.011 (0.03)	0.056 (0.076)	0.014 (0.051)	Body fat %
rs61975142	14	59402590	1,00	G	A	0.015 (0.002)	0.002 (0.023)	-0.051 (0.034)	0.053 (0.039)	-0.043 (0.101)	0.078 (0.069)	Body fat %
rs217672	14	62361021	0,99	C	A	0.012 (0.002)	0.014 (0.021)	-0.021 (0.031)	0.042 (0.034)	0.075 (0.088)	0.032 (0.058)	Body fat %
rs12890931	14	69753369	1,00	G	T	0.012 (0.002)	0.016 (0.018)	0.037 (0.027)	-0.008 (0.031)	0.094 (0.081)	-0.024 (0.053)	Body fat %
rs2370982	14	79890677	1,00	T	C	0.019 (0.002)	0.025 (0.022)	0.023 (0.032)	0.025 (0.036)	0.09 (0.096)	0.005 (0.062)	Body fat %
rs1075472	14	93108131	0,99	A	G	0.015 (0.002)	0.014 (0.024)	0.017 (0.036)	0.016 (0.039)	-0.058 (0.097)	0.03 (0.065)	Body fat %
rs6575340	14	94023972	1,00	A	G	0.015 (0.002)	0.014 (0.019)	0.029 (0.028)	0.019 (0.031)	-0.048 (0.079)	-0.03 (0.054)	Body fat %
rs3803286	14	103246470	0,99	A	G	0.013 (0.002)	-0.015 (0.019)	-0.03 (0.029)	-0.014 (0.032)	0.038 (0.081)	0.01 (0.054)	Body fat %
rs8042404	15	31680016	1,00	A	G	0.012 (0.002)	-0.028 (0.02)	-0.073 (0.03)	-0.004 (0.033)	0.014 (0.085)	0.051 (0.058)	Body fat %
rs28538451	15	41455769	0,99	A	C	0.013 (0.002)	0.018 (0.02)	0.01 (0.03)	-0.005 (0.034)	0.022 (0.086)	0.121 (0.06)	Body fat %
rs11852419	15	41456374	0,99	T	A	0.013 (0.002)	0.018 (0.02)	0.01 (0.03)	-0.005 (0.034)	0.022 (0.086)	0.121 (0.06)	Body fat %
rs3751585	15	51741056	0,98	A	G	0.013 (0.002)	-0.009 (0.023)	-0.044 (0.036)	0.038 (0.038)	0.019 (0.099)	-0.044 (0.065)	Body fat %
rs2165991	15	53101700	0,98	G	A	0.012 (0.002)	-0.024 (0.021)	-0.007 (0.031)	-0.04 (0.035)	-0.092 (0.091)	-0.005 (0.059)	Body fat %
rs17291497	15	67362994	0,98	C	T	0.01 (0.002)	-0.004 (0.018)	0.014 (0.027)	0.014 (0.03)	-0.089 (0.08)	-0.089 (0.052)	Body fat %
rs7166081	15	67492301	1,00	A	G	0.015 (0.002)	0.034 (0.022)	-0.007 (0.032)	0.039 (0.036)	0.14 (0.091)	0.111 (0.06)	Body fat %
rs16951304	15	68089618	1,00	T	C	0.02 (0.002)	0.021 (0.023)	0.036 (0.034)	0.026 (0.037)	-0.071 (0.092)	0.003 (0.063)	Body fat %
rs7164727	15	73093991	0,99	T	C	0.015 (0.002)	0.012 (0.019)	0.011 (0.029)	0.029 (0.032)	-0.027 (0.082)	-0.015 (0.055)	Body fat %
rs2660824	15	73618309	0,96	C	T	0.011 (0.002)	0.006 (0.018)	0.033 (0.027)	-0.019 (0.03)	0.096 (0.079)	-0.06 (0.052)	Body fat %
rs34769775	15	80989172	0,97	C	T	0.011 (0.002)	-0.04 (0.02)	-0.062 (0.03)	-0.028 (0.033)	-0.001 (0.089)	-0.013 (0.058)	Body fat %
rs6602997	15	84521398	0,99	T	C	0.022 (0.002)	-0.022 (0.02)	-0.028 (0.03)	0.013 (0.033)	-0.139 (0.086)	-0.051 (0.057)	Body fat %
rs2135877	15	84663107	0,99	G	A	0.021 (0.002)	0.011 (0.019)	0.004 (0.028)	0.026 (0.031)	-0.087 (0.082)	0.037 (0.055)	Body fat %
rs62021171	15	85128550	0,94	G	A	0.016 (0.002)	-0.027 (0.023)	0.01 (0.035)	-0.024 (0.037)	-0.101 (0.098)	-0.142 (0.068)	Body fat %
rs1879529	15	89414295	0,91	G	T	0.015 (0.002)	0.002 (0.021)	0.008 (0.031)	0.023 (0.035)	0.058 (0.089)	-0.127 (0.063)	Body fat %
rs57221746	15	99230035	0,97	G	A	0.012 (0.002)	0.02 (0.022)	-0.006 (0.034)	0.054 (0.036)	-0.041 (0.095)	0.032 (0.06)	Body fat %
rs412243	16	339672	0,99	T	C	0.012 (0.002)	0.009 (0.019)	-0.008 (0.028)	0.029 (0.031)	-0.042 (0.079)	0.033 (0.053)	Body fat %
rs879620	16	4015729	0,99	T	C	0.016 (0.002)	0.006 (0.018)	-0.021 (0.027)	0.033 (0.03)	0.149 (0.081)	-0.04 (0.054)	Body fat %
rs4785955	16	4297651	0,96	T	G	0.013 (0.002)	0.031 (0.022)	0.071 (0.033)	-0.001 (0.036)	0.077 (0.095)	-0.039 (0.064)	Body fat %
rs7193783	16	4949608	0,97	A	C	0.011 (0.002)	-0.022 (0.019)	0.01 (0.029)	-0.032 (0.031)	0.061 (0.083)	-0.144 (0.054)	Body fat %
rs4474693	16	20246313	0,99	A	G	0.017 (0.003)	-0.018 (0.026)	-0.006 (0.039)	0.022 (0.044)	-0.221 (0.113)	-0.084 (0.074)	Body fat %
rs200534	16	24752641	1,00	A	G	0.015 (0.002)	0.013 (0.02)	-0.01 (0.03)	0.044 (0.034)	0.057 (0.086)	-0.007 (0.058)	Body fat %
rs9939450	16	28301487	1,00	T	C	0.014 (0.002)	0.027 (0.018)	0.031 (0.027)	0.019 (0.03)	0.02 (0.077)	0.041 (0.051)	Body fat %
rs56186137	16	28825953	0,96	G	A	0.023 (0.002)	0.041 (0.019)	0.061 (0.028)	0.014 (0.03)	0.15 (0.079)	0.005 (0.052)	Body fat %
rs72798148	16	29926552	0,87	T	C	0.018 (0.002)	0.029 (0.024)	0.014 (0.035)	0.1 (0.04)	0.017 (0.098)	-0.124 (0.071)	Body fat %
rs9746755	16	30519628	0,81	G	A	0.01 (0.002)	0 (0.02)	-0.024 (0.03)	0.03 (0.033)	0.082 (0.083)	-0.036 (0.055)	Body fat %
rs34898535	16	31025641	0,98	C	T	0.016 (0.002)	-0.014 (0.019)	-0.019 (0.028)	-0.006 (0.031)	0 (0.08)	-0.023 (0.052)	Body fat %
rs11642015	16	53802494	0,99	T	C	0.04 (0.002)	0.035 (0.018)	0.021 (0.028)	0.079 (0.03)	-0.032 (0.077)	-0.013 (0.052)	Body fat %
rs7202116	16	53821615	1,00	G	A	0.039 (0.002)	0.033 (0.018)	0.009 (0.027)	0.092 (0.03)	-0.033 (0.077)	-0.023 (0.052)	Body fat %
rs9940346	16	64716851	0,95	C	T	0.011 (0.002)	0.001 (0.019)	-0.015 (0.028)	-0.022 (0.031)	0.05 (0.086)	0.102 (0.051)	Body fat %
rs11866219	16	69549749	0,97	A	C	0.017 (0.002)	0.013 (0.019)	0.011 (0.028)	0.042 (0.031)	0.128 (0.08)	-0.117 (0.053)	Body fat %
rs889398	16	69556715	0,99	C	T	0.016 (0.002)	0.023 (0.018)	0.009 (0.028)	0.04 (0.03)	0.178 (0.077)	-0.051 (0.052)	Body fat %
rs7191938	16	71407530	1,00	G	A	0.012 (0.002)	0 (0.02)	-0.032 (0.029)	0.029 (0.033)	-0.072 (0.083)	0.064 (0.056)	Body fat %
rs2012817	16	72038659	0,99	C	T	0.011 (0.002)	0.025 (0.019)	0.011 (0.029)	0.055 (0.032)	0.052 (0.083)	-0.024 (0.055)	Body fat %
rs4411525	16	74595502	0,95	A	C	0.01 (0.002)	0.019 (0.019)	0.038 (0.029)	0.02 (0.031)	0.076 (0.08)	-0.069 (0.053)	Body fat %
rs55637757	16	89535888	0,88	C	T	0.018 (0.003)	0.026 (0.03)	0.016 (0.049)	0.087 (0.048)	-0.175 (0.125)	-0.037 (0.078)	Body fat %
rs77733403	16	90080723	0,85	T	C	0.015 (0.002)	0.026 (0.027)	0.019 (0.043)	0.078 (0.042)	-0.002 (0.118)	-0.083 (0.067)	Body fat %
rs4790841	17	1835482	0,93	C	T	0.022 (0.002)	0.004 (0.025)	-0.033 (0.039)	0.067 (0.041)	-0.02 (0.106)	-0.052 (0.07)	Body fat %
rs34356467	17	3978531	0,99	T	G	0.014 (0.002)	0.033 (0.024)	0.012 (0.035)	0.07 (0.04)	0.159 (0.102)	-0.051 (0.066)	Body fat %
rs2242449	17	7095507	0,92	T	C	0.012 (0.002)	0.022 (0.019)	0.01 (0.028)	0.024 (0.031)	0.17 (0.08)	-0.004 (0.054)	Body fat %

rs9902386	17	21280185	0,93	T	G	0.012 (0.002)	-0.037 (0.02)	-0.053 (0.031)	-0.057 (0.032)	-0.016 (0.087)	0.066 (0.055)	Body fat %
rs71371126	17	28002642	0,97	A	G	0.012 (0.002)	-0.021 (0.02)	-0.034 (0.029)	-0.029 (0.032)	-0.038 (0.083)	0.053 (0.055)	Body fat %
rs11872020	17	28553489	0,93	A	G	0.013 (0.002)	-0.021 (0.019)	-0.023 (0.028)	-0.057 (0.032)	-0.055 (0.084)	0.105 (0.054)	Body fat %
rs2285639	17	34951327	0,99	C	T	0.014 (0.002)	0.034 (0.018)	0.01 (0.027)	0.096 (0.03)	-0.032 (0.08)	-0.037 (0.052)	Body fat %
rs2855818	17	42290015	0,96	A	G	0.017 (0.002)	-0.001 (0.021)	-0.014 (0.031)	0.017 (0.035)	0.005 (0.097)	-0.009 (0.061)	Body fat %
rs8074938	17	43153006	0,97	A	G	0.011 (0.002)	-0.007 (0.019)	-0.035 (0.028)	0.037 (0.032)	0.085 (0.083)	-0.075 (0.055)	Body fat %
rs17698176	17	44819595	0,82	G	T	0.012 (0.002)	0.015 (0.025)	NA (NA)	0.078 (0.037)	-0.063 (0.1)	-0.004 (0.061)	Body fat %
rs208015	17	46252346	0,99	T	C	0.025 (0.003)	0.004 (0.033)	-0.036 (0.048)	0.054 (0.055)	0.057 (0.148)	-0.017 (0.102)	Body fat %
rs11079852	17	47095041	0,98	G	A	0.013 (0.002)	-0.004 (0.019)	0.009 (0.028)	-0.012 (0.031)	0.007 (0.08)	-0.032 (0.054)	Body fat %
rs12602556	17	65826861	0,98	G	A	0.022 (0.002)	-0.002 (0.023)	0.008 (0.034)	0 (0.037)	-0.052 (0.099)	-0.02 (0.064)	Body fat %
rs11150745	17	78757626	0,98	A	G	0.013 (0.002)	-0.006 (0.02)	0.023 (0.03)	-0.038 (0.032)	-0.089 (0.083)	0.024 (0.056)	Body fat %
rs11873650	18	13163903	0,98	G	A	0.011 (0.002)	-0.022 (0.02)	-0.028 (0.03)	0.002 (0.032)	0.039 (0.084)	-0.096 (0.056)	Body fat %
rs11874274	18	13164037	0,98	T	A	0.011 (0.002)	-0.022 (0.02)	-0.029 (0.03)	0.001 (0.032)	0.045 (0.084)	-0.097 (0.056)	Body fat %
rs1893659	18	21080859	0,98	C	A	0.015 (0.002)	0.026 (0.018)	0.014 (0.027)	0.042 (0.03)	0.153 (0.079)	-0.033 (0.051)	Body fat %
rs111581974	18	22205000	0,98	C	T	0.016 (0.002)	0.003 (0.024)	-0.001 (0.034)	0.034 (0.039)	-0.046 (0.103)	-0.055 (0.067)	Body fat %
rs2052607	18	40788387	0,99	G	A	0.015 (0.002)	-0.015 (0.019)	0.007 (0.028)	-0.04 (0.032)	-0.059 (0.081)	0.001 (0.054)	Body fat %
rs11876574	18	42595844	1,00	T	C	0.012 (0.002)	-0.01 (0.02)	0.008 (0.029)	-0.011 (0.032)	-0.002 (0.085)	-0.08 (0.056)	Body fat %
rs12967878	18	57826570	0,98	C	T	0.024 (0.002)	0.047 (0.022)	0.069 (0.032)	0.008 (0.036)	0.086 (0.095)	0.068 (0.061)	Body fat %
rs538656	18	57850422	0,99	T	G	0.023 (0.002)	0.045 (0.021)	0.064 (0.032)	0.012 (0.035)	0.084 (0.092)	0.059 (0.06)	Body fat %
rs111718521	18	58085845	0,97	G	A	0.072 (0.007)	0.007 (0.068)	-0.055 (0.102)	0.166 (0.111)	0.061 (0.302)	-0.242 (0.182)	Body fat %
rs193239997	18	58639547	0,94	T	C	0.046 (0.007)	0.126 (0.07)	0.089 (0.112)	0.179 (0.108)	0.292 (0.296)	-0.002 (0.19)	Body fat %
rs350832	19	4069426	0,98	A	G	0.015 (0.002)	0.044 (0.022)	0.048 (0.033)	0.02 (0.036)	0.19 (0.092)	0.032 (0.063)	Body fat %
rs273507	19	18221964	0,97	C	A	0.018 (0.002)	0.001 (0.02)	-0.016 (0.03)	0.024 (0.033)	-0.112 (0.085)	0.04 (0.057)	Body fat %
rs2302209	19	18324329	1,00	T	C	0.019 (0.002)	-0.004 (0.02)	-0.023 (0.03)	0.013 (0.033)	-0.069 (0.085)	0.047 (0.057)	Body fat %
rs10404726	19	18834514	0,98	C	T	0.013 (0.002)	0.014 (0.018)	0.029 (0.027)	-0.011 (0.03)	0.047 (0.078)	0.022 (0.051)	Body fat %
rs62104483	19	30300017	0,98	A	G	0.013 (0.002)	0.033 (0.019)	0.059 (0.028)	0.007 (0.032)	-0.085 (0.084)	0.064 (0.055)	Body fat %
rs12610925	19	33880349	0,99	G	A	0.017 (0.002)	0.022 (0.019)	0.021 (0.029)	0.024 (0.031)	-0.033 (0.081)	0.043 (0.054)	Body fat %
rs33836	19	34008600	0,98	C	T	0.017 (0.002)	-0.009 (0.018)	-0.011 (0.027)	-0.028 (0.03)	0.048 (0.078)	0.028 (0.052)	Body fat %
rs6857	19	45392254	0,96	C	T	0.016 (0.002)	0.053 (0.024)	0.071 (0.036)	0.038 (0.04)	0.002 (0.104)	0.054 (0.069)	Body fat %
rs429358	19	45411941	0,94	T	C	0.017 (0.002)	0.05 (0.027)	0.076 (0.042)	0.022 (0.043)	-0.038 (0.114)	0.09 (0.072)	Body fat %
rs11672660	19	46180184	0,98	C	T	0.023 (0.002)	0.055 (0.022)	0.033 (0.033)	0.081 (0.036)	0.112 (0.096)	0.032 (0.064)	Body fat %
rs3810291	19	47569003	0,93	A	G	0.012 (0.002)	-0.023 (0.02)	-0.04 (0.031)	0.022 (0.032)	-0.04 (0.087)	-0.094 (0.055)	Body fat %
rs7020	20	25278600	0,99	A	G	0.013 (0.002)	-0.005 (0.018)	-0.005 (0.027)	0.015 (0.03)	0.011 (0.079)	-0.067 (0.051)	Body fat %
rs819168	20	32903845	0,94	G	T	0.021 (0.004)	0.052 (0.035)	0.076 (0.052)	0.095 (0.057)	-0.033 (0.16)	-0.172 (0.109)	Body fat %
rs17265513	20	39832628	0,98	C	T	0.013 (0.002)	-0.004 (0.023)	-0.001 (0.034)	-0.004 (0.038)	-0.074 (0.101)	0.012 (0.064)	Body fat %
rs6103254	20	41990761	0,98	T	C	0.016 (0.003)	-0.017 (0.028)	-0.016 (0.043)	-0.036 (0.045)	-0.041 (0.114)	0.052 (0.078)	Body fat %
rs112852122	20	47498117	0,95	G	A	0.018 (0.002)	0.087 (0.025)	0.09 (0.038)	0.091 (0.042)	0.161 (0.109)	0.038 (0.07)	Body fat %
rs73142879	20	51195932	0,95	C	T	0.015 (0.002)	0.007 (0.024)	0.021 (0.036)	0.011 (0.039)	-0.249 (0.105)	0.047 (0.064)	Body fat %
rs1056441	20	62370349	0,97	C	T	0.013 (0.002)	0.039 (0.02)	-0.001 (0.03)	0.107 (0.032)	-0.071 (0.083)	0.019 (0.054)	Body fat %
rs76040172	21	46488959	0,94	G	A	0.028 (0.004)	0.052 (0.041)	-0.046 (0.06)	0.16 (0.068)	-0.091 (0.175)	0.18 (0.118)	Body fat %
rs394608	21	46581798	1,00	C	T	0.013 (0.002)	0.019 (0.018)	0.018 (0.027)	0.026 (0.03)	-0.028 (0.077)	0.022 (0.051)	Body fat %
rs395379	22	18212923	0,97	G	A	0.014 (0.002)	0.058 (0.034)	NA (NA)	0.083 (0.041)	-0.147 (0.104)	0.076 (0.071)	Body fat %
rs4820325	22	38599978	0,99	G	A	0.017 (0.002)	0.012 (0.018)	0.02 (0.027)	0.007 (0.03)	-0.073 (0.08)	0.034 (0.052)	Body fat %
rs202661	22	41812439	0,99	A	G	0.013 (0.002)	-0.016 (0.022)	-0.045 (0.034)	0 (0.036)	-0.1 (0.094)	0.078 (0.062)	Body fat %
rs10854853	22	48874412	0,98	T	G	0.011 (0.002)	-0.001 (0.018)	0.033 (0.028)	-0.053 (0.03)	-0.022 (0.08)	0.042 (0.052)	Body fat %
rs2493288	1	3330884	0,94	A	G	0.025 (0.003)	0.009 (0.026)	0.067 (0.039)	-0.069 (0.043)	0.032 (0.113)	0.016 (0.076)	DBP
rs2995034	1	7770901	0,99	C	T	0.013 (0.002)	0.002 (0.018)	0.008 (0.027)	0.006 (0.03)	0.121 (0.079)	-0.085 (0.051)	DBP
rs301805	1	8481016	1,00	G	T	0.017 (0.002)	-0.05 (0.018)	-0.043 (0.027)	-0.063 (0.03)	0.031 (0.077)	-0.072 (0.052)	DBP
rs682178	1	10798552	0,95	C	T	0.015 (0.002)	-0.004 (0.019)	-0.004 (0.028)	-0.025 (0.031)	0.013 (0.081)	0.049 (0.054)	DBP

rs7537765	1	11887303	1,00	A	G	0.034 (0.003)	0.004 (0.025)	0.004 (0.038)	-0.019 (0.041)	-0.132 (0.103)	0.128 (0.069)	DBP
rs55857306	1	11895795	1,00	G	A	0.034 (0.003)	0.004 (0.025)	0.003 (0.038)	-0.019 (0.041)	-0.123 (0.103)	0.127 (0.069)	DBP
rs72654647	1	25022314	0,93	A	G	0.016 (0.003)	-0.004 (0.021)	-0.004 (0.032)	-0.028 (0.035)	-0.031 (0.096)	0.078 (0.061)	DBP
rs2782652	1	43926305	1,00	C	T	0.016 (0.002)	0.008 (0.018)	-0.015 (0.027)	-0.026 (0.03)	0.12 (0.078)	0.141 (0.052)	DBP
rs351365	1	113046395	0,92	C	T	0.022 (0.003)	-0.004 (0.021)	-0.008 (0.032)	0.072 (0.035)	-0.138 (0.093)	-0.17 (0.062)	DBP
rs35023236	1	113085452	0,99	G	C	0.023 (0.003)	0.006 (0.022)	0.021 (0.033)	0.039 (0.037)	-0.029 (0.096)	-0.133 (0.063)	DBP
rs1627925	1	113559934	0,98	C	T	0.015 (0.002)	-0.005 (0.018)	0.005 (0.027)	-0.026 (0.03)	-0.113 (0.078)	0.067 (0.052)	DBP
rs11102914	1	115812379	1,00	G	A	0.014 (0.002)	0.018 (0.018)	0.033 (0.028)	-0.013 (0.03)	0.055 (0.08)	0.041 (0.052)	DBP
rs12405515	1	172357441	1,00	G	T	0.014 (0.002)	0.023 (0.018)	0.048 (0.027)	0.006 (0.03)	0.195 (0.079)	-0.093 (0.052)	DBP
rs7527583	1	201733944	0,97	G	A	0.016 (0.002)	0.026 (0.019)	0.029 (0.028)	0.019 (0.031)	-0.014 (0.08)	0.048 (0.053)	DBP
rs72742024	1	201737722	0,98	C	T	0.017 (0.002)	0.024 (0.019)	0.021 (0.029)	0.033 (0.031)	-0.012 (0.081)	0.025 (0.054)	DBP
rs6426577	1	227355918	0,99	C	A	0.015 (0.002)	-0.002 (0.018)	-0.018 (0.028)	0.022 (0.03)	0.024 (0.077)	-0.028 (0.052)	DBP
rs2004776	1	230848702	1,00	T	C	0.021 (0.003)	-0.056 (0.021)	-0.049 (0.031)	-0.066 (0.035)	0.009 (0.09)	-0.078 (0.06)	DBP
rs3943093	1	243458502	1,00	T	C	0.025 (0.002)	0.046 (0.019)	0.029 (0.028)	0.067 (0.032)	0.021 (0.081)	0.056 (0.054)	DBP
rs1275985	2	26911745	0,99	C	T	0.021 (0.002)	-0.042 (0.018)	-0.051 (0.027)	-0.031 (0.031)	0.034 (0.081)	-0.071 (0.052)	DBP
rs12712870	2	43161180	0,98	A	G	0.015 (0.002)	-0.02 (0.02)	-0.017 (0.03)	-0.027 (0.033)	-0.058 (0.086)	0.012 (0.056)	DBP
rs76326501	2	43167878	0,94	A	C	0.026 (0.004)	-0.024 (0.033)	-0.024 (0.052)	-0.04 (0.053)	0.16 (0.135)	-0.059 (0.089)	DBP
rs4952668	2	43386568	0,97	G	A	0.014 (0.002)	-0.021 (0.019)	-0.061 (0.028)	-0.001 (0.031)	0.049 (0.083)	0.038 (0.054)	DBP
rs6708660	2	43752382	1,00	T	C	0.016 (0.002)	-0.023 (0.018)	-0.022 (0.028)	-0.029 (0.03)	-0.043 (0.079)	-0.002 (0.052)	DBP
rs7604588	2	73571417	0,97	A	G	0.016 (0.003)	0.011 (0.02)	0.033 (0.03)	0.002 (0.033)	-0.064 (0.084)	-0.009 (0.058)	DBP
rs2010379	2	96180093	0,94	C	T	0.016 (0.002)	0.042 (0.019)	0.047 (0.029)	0.053 (0.032)	0.056 (0.082)	-0.018 (0.054)	DBP
rs7604842	2	96777340	1,00	C	T	0.02 (0.002)	0.038 (0.019)	0.058 (0.028)	0.046 (0.032)	0.035 (0.08)	-0.064 (0.054)	DBP
rs10177168	2	97280240	0,96	A	G	0.015 (0.002)	0.023 (0.02)	0.023 (0.031)	0.044 (0.032)	0.074 (0.087)	-0.059 (0.054)	DBP
rs4954192	2	135632981	0,99	C	T	0.014 (0.002)	0.033 (0.018)	0.026 (0.028)	0.036 (0.03)	0.063 (0.079)	0.036 (0.053)	DBP
rs786250	2	145701992	1,00	T	C	0.019 (0.002)	0.029 (0.019)	0.037 (0.027)	0.008 (0.031)	0.051 (0.082)	0.05 (0.054)	DBP
rs72854462	2	145720139	1,00	G	A	0.023 (0.003)	0.03 (0.02)	0.06 (0.03)	0 (0.034)	0.023 (0.091)	0.002 (0.059)	DBP
rs13028348	2	148757144	1,00	G	A	0.015 (0.002)	0.015 (0.019)	0.004 (0.029)	0.035 (0.032)	0.036 (0.083)	-0.013 (0.056)	DBP
rs3821292	2	159437399	1,00	C	T	0.017 (0.003)	-0.034 (0.02)	-0.073 (0.03)	-0.004 (0.033)	0.007 (0.086)	0.008 (0.057)	DBP
rs1446468	2	164963486	0,97	C	T	0.018 (0.002)	-0.01 (0.018)	-0.041 (0.027)	0.022 (0.03)	0.049 (0.076)	-0.02 (0.052)	DBP
rs908670	2	172690180	0,99	T	C	0.016 (0.003)	-0.029 (0.02)	-0.03 (0.03)	-0.05 (0.033)	0.119 (0.086)	-0.029 (0.058)	DBP
rs17362588	2	179721046	0,99	A	G	0.032 (0.004)	-0.024 (0.032)	-0.095 (0.046)	-0.005 (0.052)	0.084 (0.139)	0.155 (0.092)	DBP
rs12693302	2	183211443	0,98	G	A	0.023 (0.002)	0.021 (0.019)	0.015 (0.028)	0.047 (0.032)	0.071 (0.081)	-0.056 (0.055)	DBP
rs62182897	2	191471395	0,99	C	T	0.017 (0.003)	0.03 (0.025)	0.02 (0.038)	0.027 (0.04)	0.032 (0.102)	0.066 (0.065)	DBP
rs2571445	2	218683154	1,00	A	G	0.014 (0.002)	-0.028 (0.019)	-0.032 (0.028)	0.008 (0.031)	-0.162 (0.081)	-0.066 (0.052)	DBP
rs10210244	2	235792003	0,84	T	C	0.022 (0.004)	0.027 (0.03)	NA (NA)	0.043 (0.048)	0.137 (0.126)	0.104 (0.084)	DBP
rs3804972	3	7486556	1,00	A	G	0.014 (0.002)	0.039 (0.018)	0.038 (0.027)	0.02 (0.03)	0.051 (0.079)	0.091 (0.052)	DBP
rs1687295	3	14889756	0,99	T	C	0.018 (0.003)	0.026 (0.02)	0.046 (0.03)	0.008 (0.034)	0.1 (0.086)	-0.031 (0.058)	DBP
rs9848210	3	27470734	0,99	T	C	0.033 (0.006)	-0.046 (0.044)	-0.03 (0.066)	-0.001 (0.072)	-0.561 (0.196)	-0.037 (0.125)	DBP
rs9856633	3	42013850	0,99	A	G	0.029 (0.003)	0.049 (0.024)	0.007 (0.036)	0.117 (0.04)	0.036 (0.1)	0.014 (0.069)	DBP
rs6800730	3	48174210	0,97	G	A	0.016 (0.002)	0.02 (0.02)	0.019 (0.029)	0.04 (0.032)	-0.121 (0.083)	0.029 (0.054)	DBP
rs4855867	3	49745822	0,99	C	T	0.017 (0.002)	0.014 (0.018)	-0.008 (0.027)	0.02 (0.03)	0.019 (0.078)	0.071 (0.051)	DBP
rs2061573	3	57763642	1,00	A	G	0.016 (0.002)	-0.013 (0.018)	0.01 (0.027)	-0.026 (0.03)	-0.121 (0.079)	-0.013 (0.053)	DBP
rs9863881	3	122100023	1,00	T	C	0.015 (0.002)	0.027 (0.018)	0.066 (0.027)	-0.023 (0.03)	0.05 (0.08)	0.025 (0.052)	DBP
rs9869147	3	124614317	0,98	C	T	0.014 (0.002)	-0.02 (0.018)	-0.045 (0.027)	-0.012 (0.03)	0.003 (0.079)	0.034 (0.052)	DBP
rs9866391	3	141076084	0,99	C	T	0.016 (0.002)	0.021 (0.018)	0.015 (0.028)	0.02 (0.03)	0.007 (0.08)	0.052 (0.052)	DBP
rs57234617	3	154660134	0,95	T	G	0.021 (0.004)	0.012 (0.028)	0.003 (0.041)	-0.005 (0.048)	0.069 (0.123)	0.067 (0.081)	DBP
rs1273886	3	169091253	0,99	G	A	0.021 (0.002)	0.029 (0.018)	0.018 (0.027)	0.041 (0.031)	0.013 (0.079)	0.044 (0.053)	DBP
rs4955658	3	169171347	0,99	T	G	0.023 (0.002)	0.029 (0.018)	0.023 (0.027)	0.044 (0.03)	-0.024 (0.079)	0.032 (0.052)	DBP
rs6854757	4	17932555	0,98	G	A	0.018 (0.003)	0.02 (0.025)	-0.037 (0.037)	0.037 (0.04)	0.007 (0.102)	0.174 (0.069)	DBP

rs35107212	4	26799390	0,99	G	A	0.014 (0.002)	0.016 (0.018)	0.004 (0.028)	-0.008 (0.03)	0.058 (0.078)	0.111 (0.052)	DBP
rs60991988	4	54801228	0,96	G	T	0.023 (0.004)	0.002 (0.03)	-0.023 (0.045)	0.02 (0.049)	0.065 (0.127)	0.012 (0.09)	DBP
rs12509595	4	81182554	0,98	C	T	0.035 (0.003)	-0.037 (0.02)	-0.044 (0.03)	-0.03 (0.033)	-0.029 (0.087)	-0.033 (0.056)	DBP
rs1527351	4	102084331	1,00	T	C	0.014 (0.002)	-0.005 (0.019)	0.028 (0.029)	0.006 (0.032)	-0.111 (0.079)	-0.108 (0.055)	DBP
rs13107325	4	103188709	0,97	C	T	0.041 (0.004)	0.032 (0.035)	0.048 (0.052)	0.016 (0.058)	-0.047 (0.146)	0.053 (0.097)	DBP
rs223361	4	103769304	0,98	T	C	0.015 (0.002)	0.006 (0.019)	0.023 (0.029)	-0.005 (0.032)	0.078 (0.083)	-0.05 (0.055)	DBP
rs9994289	4	111338277	0,96	T	C	0.018 (0.003)	-0.043 (0.023)	-0.02 (0.035)	-0.072 (0.036)	-0.064 (0.096)	-0.019 (0.066)	DBP
rs66887589	4	120509279	0,99	C	T	0.014 (0.002)	-0.001 (0.018)	-0.017 (0.027)	-0.003 (0.03)	-0.022 (0.079)	0.072 (0.052)	DBP
rs930457	4	143785362	0,98	C	T	0.014 (0.002)	-0.018 (0.019)	-0.038 (0.029)	-0.011 (0.032)	0.048 (0.082)	0.002 (0.054)	DBP
rs28409115	4	156645933	0,99	A	G	0.014 (0.002)	0.026 (0.018)	0.046 (0.027)	0.019 (0.03)	-0.019 (0.078)	-0.012 (0.052)	DBP
rs11731886	4	156659819	0,99	A	C	0.017 (0.003)	0.026 (0.021)	0.036 (0.031)	0.028 (0.035)	0.027 (0.088)	-0.018 (0.059)	DBP
rs12656497	5	32831939	0,99	C	T	0.026 (0.002)	0.035 (0.018)	0.028 (0.027)	0.073 (0.031)	-0.004 (0.08)	-0.034 (0.052)	DBP
rs35807464	5	61941653	0,97	A	G	0.031 (0.005)	0.024 (0.033)	0.045 (0.047)	0.007 (0.057)	0.171 (0.153)	-0.097 (0.108)	DBP
rs7719522	5	122634868	0,97	G	T	0.014 (0.002)	0.002 (0.02)	0.011 (0.029)	-0.014 (0.032)	0.046 (0.082)	0 (0.056)	DBP
rs13156484	5	122653399	0,97	A	G	0.019 (0.002)	0.019 (0.018)	0.008 (0.028)	0.047 (0.03)	0.005 (0.078)	-0.019 (0.052)	DBP
rs6595828	5	127817549	1,00	C	T	0.017 (0.002)	0.013 (0.019)	-0.003 (0.027)	0.05 (0.031)	-0.141 (0.079)	0.036 (0.052)	DBP
rs1078721	5	148357999	1,00	C	T	0.019 (0.003)	0.002 (0.022)	-0.034 (0.032)	0.041 (0.036)	-0.067 (0.095)	0.047 (0.063)	DBP
rs113700836	5	157394503	0,95	T	C	0.016 (0.003)	-0.005 (0.022)	0.051 (0.032)	-0.053 (0.035)	-0.018 (0.093)	-0.051 (0.062)	DBP
rs13436194	5	157803588	0,98	A	G	0.014 (0.002)	-0.007 (0.018)	0.007 (0.027)	-0.011 (0.03)	0.06 (0.079)	-0.082 (0.054)	DBP
rs17717829	5	158470063	0,99	T	C	0.016 (0.002)	0.002 (0.018)	-0.03 (0.027)	0.042 (0.03)	0.014 (0.078)	-0.004 (0.052)	DBP
rs7705507	5	173358337	1,00	C	T	0.023 (0.002)	-0.057 (0.019)	-0.103 (0.028)	-0.034 (0.032)	0.006 (0.084)	0.017 (0.056)	DBP
rs530145	6	25546801	1,00	G	A	0.015 (0.002)	0.04 (0.018)	0.026 (0.028)	0.055 (0.03)	0.147 (0.078)	0.002 (0.052)	DBP
rs2032451	6	26092170	1,00	T	G	0.028 (0.003)	-0.016 (0.025)	0.005 (0.038)	-0.008 (0.042)	-0.085 (0.105)	-0.087 (0.072)	DBP
rs115740542	6	26123502	0,95	C	T	0.03 (0.004)	-0.023 (0.041)	0.06 (0.068)	-0.119 (0.064)	0.051 (0.158)	0.001 (0.1)	DBP
rs6917995	6	26327814	1,00	T	C	0.016 (0.002)	0.016 (0.018)	0.028 (0.027)	0.027 (0.03)	-0.13 (0.078)	0.003 (0.051)	DBP
rs9379851	6	26354780	1,00	A	C	0.02 (0.003)	0.042 (0.029)	0.007 (0.044)	0.071 (0.047)	-0.196 (0.124)	0.163 (0.076)	DBP
rs113039233	6	27430126	0,94	G	A	0.015 (0.003)	0.03 (0.021)	0.024 (0.032)	0.022 (0.034)	0.129 (0.092)	0.036 (0.057)	DBP
rs13437444	6	28070998	0,99	C	T	0.025 (0.003)	0.058 (0.024)	0.039 (0.036)	0.059 (0.039)	0.032 (0.099)	0.126 (0.063)	DBP
rs34477097	6	28197186	1,00	C	T	0.023 (0.003)	0.039 (0.022)	0.027 (0.032)	0.029 (0.036)	0.088 (0.093)	0.089 (0.06)	DBP
rs144447022	6	29244219	1,00	G	T	0.022 (0.004)	0.09 (0.032)	-0.002 (0.051)	0.12 (0.052)	0.06 (0.14)	0.258 (0.08)	DBP
rs1800628	6	31546850	0,99	G	A	0.027 (0.003)	0.118 (0.03)	0.063 (0.047)	0.165 (0.048)	-0.174 (0.123)	0.247 (0.075)	DBP
rs116667074	6	32285362	1,00	C	T	0.031 (0.003)	0.108 (0.03)	0.07 (0.048)	0.146 (0.048)	-0.212 (0.126)	0.232 (0.077)	DBP
rs60045856	6	32799845	0,99	T	G	0.028 (0.003)	0.08 (0.03)	0.063 (0.048)	0.083 (0.049)	-0.151 (0.127)	0.204 (0.078)	DBP
rs10484578	6	35246319	0,96	A	G	0.015 (0.002)	-0.007 (0.02)	-0.005 (0.03)	0.01 (0.032)	-0.102 (0.083)	-0.025 (0.054)	DBP
rs6927317	6	51415907	0,99	C	T	0.015 (0.002)	0 (0.018)	-0.03 (0.027)	0.071 (0.031)	-0.094 (0.079)	-0.057 (0.052)	DBP
rs9296668	6	51838263	1,00	G	A	0.017 (0.002)	0.007 (0.018)	0.027 (0.027)	0.019 (0.03)	-0.032 (0.076)	-0.082 (0.051)	DBP
rs9352691	6	79785607	0,99	T	C	0.017 (0.002)	-0.001 (0.019)	-0.026 (0.028)	0.038 (0.031)	-0.124 (0.08)	0.034 (0.053)	DBP
rs10944051	6	85641628	1,00	C	T	0.015 (0.003)	-0.018 (0.021)	0.019 (0.032)	-0.021 (0.035)	-0.158 (0.091)	-0.074 (0.058)	DBP
rs12527158	6	98408610	0,99	G	A	0.021 (0.003)	-0.004 (0.026)	0.046 (0.038)	-0.038 (0.044)	-0.089 (0.12)	-0.07 (0.076)	DBP
rs7744284	6	109616218	1,00	C	T	0.014 (0.002)	-0.016 (0.018)	-0.025 (0.026)	-0.004 (0.03)	-0.007 (0.079)	-0.024 (0.051)	DBP
rs1630266	6	118612943	1,00	A	G	0.025 (0.004)	-0.039 (0.031)	-0.099 (0.045)	0.026 (0.053)	-0.086 (0.148)	0.044 (0.094)	DBP
rs6923947	6	127098553	0,97	A	G	0.022 (0.002)	0.003 (0.018)	-0.009 (0.028)	0.025 (0.03)	0.003 (0.078)	-0.016 (0.053)	DBP
rs57139556	6	150998511	1,00	A	G	0.026 (0.004)	-0.005 (0.036)	-0.017 (0.056)	0.034 (0.059)	-0.106 (0.157)	-0.036 (0.099)	DBP
rs59641479	6	166162205	0,98	C	T	0.027 (0.005)	-0.014 (0.035)	0.006 (0.051)	-0.04 (0.058)	0.21 (0.159)	-0.105 (0.104)	DBP
rs1322640	6	169586887	0,97	T	C	0.019 (0.003)	0.007 (0.022)	-0.064 (0.033)	0.027 (0.036)	0.078 (0.096)	0.176 (0.062)	DBP
rs76450081	7	7264082	0,98	G	A	0.02 (0.003)	0.01 (0.023)	-0.006 (0.033)	-0.003 (0.04)	-0.096 (0.107)	0.175 (0.073)	DBP
rs2023843	7	27243221	1,00	T	C	0.027 (0.004)	0.04 (0.033)	-0.009 (0.048)	0.068 (0.056)	0.102 (0.142)	0.127 (0.098)	DBP
rs343011	7	35488349	0,99	T	G	0.021 (0.003)	0.004 (0.022)	0.002 (0.033)	0.019 (0.036)	0.066 (0.094)	-0.061 (0.06)	DBP
rs343052	7	35519519	0,99	G	T	0.02 (0.003)	-0.006 (0.024)	-0.005 (0.036)	0.002 (0.038)	0.105 (0.101)	-0.07 (0.064)	DBP

rs11977526	7	46008110	0,96	A	G	0.017 (0.002)	0.004 (0.019)	-0.016 (0.028)	-0.017 (0.031)	0.036 (0.08)	0.112 (0.052)	DBP
rs6460541	7	69747088	0,99	G	A	0.015 (0.003)	0.006 (0.02)	-0.045 (0.03)	0.032 (0.034)	0.29 (0.091)	-0.002 (0.058)	DBP
rs39254	7	89765708	0,90	G	T	0.017 (0.002)	0.014 (0.019)	0.039 (0.029)	-0.019 (0.031)	0.088 (0.079)	0 (0.054)	DBP
rs13247448	7	96460220	0,99	C	T	0.014 (0.002)	0.008 (0.018)	0.005 (0.027)	0.004 (0.03)	0.122 (0.077)	-0.023 (0.053)	DBP
rs13226502	7	100506381	0,99	T	C	0.023 (0.003)	0.02 (0.024)	0.025 (0.035)	0.036 (0.039)	0 (0.105)	-0.034 (0.068)	DBP
rs11556924	7	129663496	0,89	C	T	0.014 (0.002)	-0.059 (0.02)	-0.034 (0.03)	-0.089 (0.032)	-0.163 (0.089)	-0.019 (0.052)	DBP
rs3823483	7	131010943	0,99	C	T	0.014 (0.002)	0.031 (0.018)	0.037 (0.027)	0.043 (0.03)	-0.001 (0.078)	-0.014 (0.052)	DBP
rs891511	7	150704843	0,88	G	A	0.022 (0.002)	0.006 (0.02)	0.028 (0.031)	0.006 (0.032)	-0.032 (0.092)	-0.049 (0.055)	DBP
rs7006221	8	1218297	0,95	T	C	0.015 (0.003)	0.044 (0.02)	0.016 (0.03)	0.089 (0.033)	0.053 (0.089)	0.008 (0.058)	DBP
rs2921077	8	8304502	0,97	A	G	0.018 (0.002)	0.013 (0.018)	0.052 (0.027)	-0.032 (0.03)	-0.019 (0.079)	0.021 (0.052)	DBP
rs2979253	8	8919676	0,97	T	C	0.02 (0.002)	0.005 (0.018)	0.036 (0.027)	-0.019 (0.03)	-0.08 (0.08)	0.002 (0.052)	DBP
rs4075359	8	9487813	0,97	T	C	0.019 (0.002)	0.031 (0.019)	0.069 (0.029)	-0.009 (0.031)	-0.021 (0.082)	0.036 (0.054)	DBP
rs7837979	8	10198534	0,98	T	C	0.021 (0.002)	0.019 (0.019)	0.056 (0.028)	-0.01 (0.03)	-0.104 (0.081)	0.018 (0.052)	DBP
rs877116	8	10712945	0,98	G	T	0.021 (0.002)	0.019 (0.018)	0.039 (0.027)	-0.01 (0.031)	-0.011 (0.08)	0.04 (0.053)	DBP
rs34421088	8	11589042	0,99	A	G	0.019 (0.002)	0.02 (0.018)	0.07 (0.027)	-0.032 (0.03)	-0.08 (0.079)	0.031 (0.051)	DBP
rs7838131	8	11596163	0,99	G	A	0.02 (0.002)	0.014 (0.018)	0.053 (0.027)	-0.033 (0.03)	-0.084 (0.079)	0.058 (0.052)	DBP
rs11782995	8	120400847	0,99	G	A	0.023 (0.003)	-0.015 (0.021)	-0.024 (0.031)	-0.037 (0.035)	0.215 (0.091)	-0.013 (0.058)	DBP
rs12543849	8	143486698	0,97	A	G	0.014 (0.002)	0.014 (0.019)	0.049 (0.029)	-0.043 (0.032)	0.04 (0.082)	0.045 (0.054)	DBP
rs4741378	9	14456738	0,99	G	A	0.013 (0.002)	-0.008 (0.018)	-0.011 (0.027)	0.007 (0.03)	-0.05 (0.078)	-0.025 (0.051)	DBP
rs507666	9	136149399	0,99	G	A	0.023 (0.003)	0.032 (0.022)	0.052 (0.033)	0.031 (0.037)	0.011 (0.1)	-0.028 (0.065)	DBP
rs12379814	9	139519486	0,82	C	T	0.016 (0.002)	-0.008 (0.02)	-0.033 (0.032)	0.01 (0.033)	0.073 (0.079)	-0.026 (0.052)	DBP
rs12570727	10	18425519	0,99	A	G	0.016 (0.002)	0.026 (0.018)	0.031 (0.027)	0.024 (0.03)	0.01 (0.078)	0.024 (0.053)	DBP
rs1813353	10	18707448	1,00	T	C	0.021 (0.002)	-0.046 (0.019)	-0.047 (0.028)	-0.043 (0.031)	-0.036 (0.081)	-0.053 (0.053)	DBP
rs72831343	10	63515681	1,00	T	G	0.033 (0.003)	0.01 (0.026)	0.005 (0.04)	0.024 (0.043)	-0.231 (0.112)	0.093 (0.073)	DBP
rs1509966	10	64552607	0,99	G	A	0.02 (0.002)	0.005 (0.018)	0.016 (0.027)	-0.005 (0.03)	0.003 (0.077)	-0.001 (0.051)	DBP
rs7898861	10	65319678	0,99	C	T	0.02 (0.002)	0.011 (0.018)	-0.022 (0.027)	0.025 (0.03)	0.02 (0.077)	0.086 (0.051)	DBP
rs57866767	10	96023077	0,99	T	C	0.021 (0.002)	0.052 (0.018)	0.035 (0.027)	0.071 (0.03)	0.026 (0.078)	0.068 (0.052)	DBP
rs11187838	10	96038686	0,99	G	A	0.021 (0.002)	0.053 (0.018)	0.039 (0.027)	0.071 (0.03)	0.024 (0.078)	0.064 (0.052)	DBP
rs11188112	10	96666699	0,99	C	T	0.016 (0.003)	0.031 (0.022)	0.021 (0.032)	0.052 (0.035)	0.036 (0.092)	0 (0.061)	DBP
rs112184198	10	102604514	0,96	G	A	0.027 (0.004)	-0.051 (0.03)	-0.009 (0.045)	-0.042 (0.048)	-0.062 (0.128)	-0.214 (0.082)	DBP
rs11191580	10	104906211	0,99	T	C	0.032 (0.004)	0.006 (0.032)	0.004 (0.047)	0.012 (0.052)	-0.264 (0.136)	0.125 (0.095)	DBP
rs180940	10	115722411	0,99	G	A	0.018 (0.002)	-0.019 (0.019)	-0.034 (0.029)	-0.006 (0.032)	0.018 (0.082)	-0.02 (0.055)	DBP
rs2782980	10	115781527	0,98	C	T	0.025 (0.003)	0.017 (0.02)	0.006 (0.03)	0.032 (0.033)	0.124 (0.083)	-0.039 (0.058)	DBP
rs587961	11	1881256	0,92	C	T	0.017 (0.002)	0.014 (0.02)	0.007 (0.031)	-0.004 (0.032)	0.088 (0.084)	0.06 (0.055)	DBP
rs360153	11	9762274	1,00	C	T	0.015 (0.002)	0.018 (0.018)	0.031 (0.027)	0.023 (0.03)	-0.08 (0.079)	-0.002 (0.052)	DBP
rs2920152	11	10296756	0,99	G	A	0.025 (0.003)	0.025 (0.023)	0.037 (0.034)	0.002 (0.038)	0.049 (0.1)	0.042 (0.068)	DBP
rs1580004	11	10370420	1,00	C	A	0.016 (0.002)	-0.003 (0.018)	0.005 (0.027)	-0.015 (0.03)	0.16 (0.078)	-0.065 (0.052)	DBP
rs10832013	11	13295353	0,99	T	G	0.016 (0.002)	0.012 (0.02)	0.032 (0.029)	-0.046 (0.032)	0.065 (0.083)	0.087 (0.055)	DBP
rs1819083	11	14084591	1,00	T	C	0.014 (0.002)	0.01 (0.018)	0.024 (0.027)	0.001 (0.03)	0.015 (0.079)	-0.019 (0.051)	DBP
rs1966697	11	16306390	1,00	C	T	0.018 (0.003)	0.036 (0.023)	0.091 (0.034)	-0.012 (0.037)	-0.047 (0.095)	0.016 (0.063)	DBP
rs208078	11	31128721	1,00	T	G	0.014 (0.002)	-0.015 (0.019)	0.017 (0.028)	-0.065 (0.031)	0.083 (0.081)	-0.018 (0.053)	DBP
rs72910075	11	46359645	0,86	T	C	0.023 (0.004)	0.061 (0.031)	0.036 (0.048)	0.108 (0.049)	0.071 (0.129)	-0.005 (0.083)	DBP
rs11039149	11	47276675	0,99	G	A	0.016 (0.003)	0.022 (0.02)	0.005 (0.03)	0.025 (0.033)	0.058 (0.086)	0.064 (0.057)	DBP
rs7125196	11	61272565	0,99	T	C	0.035 (0.004)	0.058 (0.028)	0.05 (0.04)	0.008 (0.047)	0.322 (0.125)	0.134 (0.083)	DBP
rs751984	11	61278246	0,99	T	C	0.035 (0.004)	0.051 (0.028)	0.047 (0.039)	-0.008 (0.046)	0.337 (0.126)	0.133 (0.082)	DBP
rs2306363	11	65405600	0,97	G	T	0.02 (0.003)	-0.022 (0.023)	-0.008 (0.034)	-0.052 (0.037)	0.149 (0.095)	-0.056 (0.063)	DBP
rs11021233	11	95320986	0,98	G	A	0.022 (0.003)	-0.02 (0.026)	-0.028 (0.039)	-0.011 (0.043)	-0.089 (0.112)	0.007 (0.07)	DBP
rs604723	11	100610546	1,00	C	T	0.032 (0.003)	0.006 (0.02)	-0.03 (0.029)	0.059 (0.033)	-0.048 (0.088)	0.007 (0.058)	DBP
rs11212087	11	107097881	1,00	C	T	0.016 (0.003)	-0.006 (0.02)	0.03 (0.031)	-0.07 (0.033)	0.111 (0.087)	0.01 (0.057)	DBP

rs61911502	11	122524668	0,97	G	A	0.021 (0.004)	0.044 (0.028)	0.027 (0.041)	0.038 (0.046)	-0.034 (0.123)	0.147 (0.076)	DBP
rs7936928	11	130279168	0,96	C	T	0.021 (0.002)	0.006 (0.019)	-0.006 (0.028)	-0.008 (0.031)	0.03 (0.081)	0.081 (0.053)	DBP
rs4765675	12	2469497	0,94	G	T	0.014 (0.002)	-0.046 (0.019)	-0.063 (0.028)	-0.005 (0.031)	-0.082 (0.082)	-0.089 (0.056)	DBP
rs4762921	12	20184539	1,00	A	G	0.024 (0.003)	0.028 (0.02)	-0.011 (0.031)	0.045 (0.034)	0.073 (0.086)	0.089 (0.057)	DBP
rs73075659	12	20373541	0,98	A	G	0.022 (0.002)	0.025 (0.02)	0.037 (0.03)	0.017 (0.032)	-0.013 (0.082)	0.023 (0.054)	DBP
rs836179	12	50503082	0,99	G	A	0.021 (0.002)	-0.012 (0.019)	-0.001 (0.028)	-0.028 (0.031)	-0.046 (0.082)	0.01 (0.053)	DBP
rs11169572	12	51216890	0,97	T	C	0.016 (0.002)	0.013 (0.019)	0.013 (0.029)	0.001 (0.03)	0.058 (0.079)	0.029 (0.053)	DBP
rs73437338	12	90054619	0,98	T	C	0.024 (0.003)	-0.008 (0.025)	-0.014 (0.037)	-0.011 (0.041)	-0.142 (0.106)	0.081 (0.069)	DBP
rs1980235	12	90110782	1,00	A	G	0.018 (0.002)	0.014 (0.019)	0.001 (0.029)	0.02 (0.032)	0.01 (0.084)	0.04 (0.054)	DBP
rs7968960	12	111426615	0,87	C	A	0.021 (0.002)	-0.031 (0.02)	-0.043 (0.031)	0.028 (0.032)	-0.071 (0.082)	-0.145 (0.055)	DBP
rs597808	12	111973358	0,92	A	G	0.037 (0.002)	-0.037 (0.019)	-0.057 (0.028)	0.011 (0.031)	-0.099 (0.08)	-0.085 (0.053)	DBP
rs583140	12	111985954	0,99	G	A	0.024 (0.003)	-0.06 (0.022)	-0.084 (0.033)	-0.002 (0.037)	-0.102 (0.097)	-0.125 (0.064)	DBP
rs17630235	12	112591686	0,89	A	G	0.029 (0.002)	-0.005 (0.02)	-0.025 (0.032)	0.04 (0.031)	-0.065 (0.08)	-0.057 (0.054)	DBP
rs7300285	12	112849899	0,88	G	A	0.018 (0.002)	-0.03 (0.02)	-0.05 (0.031)	0.008 (0.033)	-0.112 (0.085)	-0.037 (0.056)	DBP
rs4767002	12	113169770	0,99	C	T	0.017 (0.002)	0.002 (0.018)	-0.021 (0.027)	0.026 (0.03)	0.085 (0.078)	-0.023 (0.052)	DBP
rs35441	12	115553115	0,99	C	T	0.017 (0.002)	-0.021 (0.019)	-0.051 (0.028)	-0.004 (0.031)	0 (0.08)	0.032 (0.054)	DBP
rs1498753	12	115933575	0,99	T	C	0.016 (0.002)	-0.006 (0.018)	0.024 (0.027)	-0.013 (0.03)	-0.043 (0.078)	-0.079 (0.052)	DBP
rs708809	12	116225350	0,99	C	T	0.016 (0.003)	-0.007 (0.021)	-0.005 (0.032)	0.008 (0.035)	-0.124 (0.089)	-0.006 (0.059)	DBP
rs28413626	12	123861452	0,88	G	A	0.021 (0.003)	-0.053 (0.024)	-0.055 (0.038)	-0.116 (0.037)	0.095 (0.098)	0.077 (0.064)	DBP
rs28715490	12	123903626	0,86	C	T	0.021 (0.003)	-0.053 (0.025)	-0.048 (0.04)	-0.116 (0.038)	0.046 (0.105)	0.077 (0.065)	DBP
rs7322054	13	38246708	1,00	C	A	0.013 (0.002)	0.012 (0.018)	0 (0.027)	0.014 (0.03)	0.02 (0.078)	0.049 (0.052)	DBP
rs656533	13	110793123	0,93	G	A	0.025 (0.004)	-0.052 (0.032)	-0.149 (0.052)	0.045 (0.051)	-0.144 (0.133)	-0.021 (0.088)	DBP
rs693831	13	110898375	0,98	T	C	0.019 (0.003)	-0.027 (0.022)	-0.04 (0.033)	-0.051 (0.038)	0.102 (0.095)	0.03 (0.063)	DBP
rs7338606	13	110900111	0,99	T	C	0.02 (0.003)	-0.03 (0.022)	-0.049 (0.033)	-0.042 (0.037)	0.086 (0.096)	0.022 (0.062)	DBP
rs7491716	13	114495479	0,77	C	A	0.014 (0.002)	0.015 (0.021)	NA (NA)	0.028 (0.03)	0.026 (0.078)	-0.025 (0.051)	DBP
rs72677850	14	50849397	0,72	G	A	0.059 (0.009)	0.016 (0.077)	NA (NA)	0.148 (0.132)	0.168 (0.311)	0.188 (0.2)	DBP
rs12432588	14	56041810	0,98	C	T	0.016 (0.003)	0.013 (0.02)	0.014 (0.031)	0.024 (0.034)	-0.006 (0.087)	-0.012 (0.058)	DBP
rs113044050	14	104007751	0,94	C	T	0.02 (0.003)	-0.015 (0.025)	-0.043 (0.037)	-0.009 (0.042)	0.112 (0.111)	0.02 (0.074)	DBP
rs2006281	14	104327732	0,91	C	T	0.013 (0.002)	-0.006 (0.019)	0.008 (0.029)	-0.018 (0.031)	0.044 (0.079)	-0.042 (0.053)	DBP
rs3736290	15	40321351	0,99	A	C	0.016 (0.002)	-0.017 (0.018)	-0.045 (0.027)	0.019 (0.03)	0.051 (0.078)	-0.048 (0.052)	DBP
rs12905926	15	41283408	1,00	G	A	0.017 (0.002)	0.017 (0.018)	0.013 (0.027)	-0.014 (0.03)	0.083 (0.077)	0.09 (0.051)	DBP
rs8038764	15	41907905	0,95	A	C	0.016 (0.002)	0.023 (0.019)	0.043 (0.028)	0.005 (0.03)	0.035 (0.078)	0.002 (0.052)	DBP
rs4923915	15	42119606	1,00	T	C	0.017 (0.002)	0.025 (0.019)	0.059 (0.028)	-0.003 (0.032)	-0.047 (0.083)	0.012 (0.054)	DBP
rs8036173	15	48820068	0,99	C	T	0.017 (0.003)	0.03 (0.021)	0.021 (0.031)	0.022 (0.034)	0.043 (0.089)	0.083 (0.059)	DBP
rs11636952	15	75114322	0,99	T	C	0.027 (0.002)	-0.042 (0.019)	-0.068 (0.029)	-0.028 (0.032)	-0.075 (0.083)	0.029 (0.056)	DBP
rs62012629	15	79070351	0,76	C	A	0.019 (0.003)	0.015 (0.023)	NA (NA)	-0.024 (0.036)	0.128 (0.091)	-0.039 (0.062)	DBP
rs2627316	15	81042816	0,99	G	A	0.014 (0.002)	-0.031 (0.018)	-0.059 (0.027)	-0.018 (0.03)	0.035 (0.077)	0.002 (0.051)	DBP
rs8027450	15	91418394	0,96	T	C	0.026 (0.002)	0.006 (0.02)	0.006 (0.028)	0.001 (0.033)	0.045 (0.085)	0.007 (0.056)	DBP
rs12906962	15	95312071	0,96	C	T	0.023 (0.002)	-0.012 (0.019)	-0.028 (0.029)	0.006 (0.032)	-0.014 (0.083)	-0.01 (0.056)	DBP
rs4984496	15	96635898	0,88	T	G	0.018 (0.002)	0.013 (0.02)	-0.008 (0.03)	0.048 (0.032)	-0.108 (0.086)	0.026 (0.055)	DBP
rs72762705	16	4020732	0,95	C	T	0.017 (0.003)	0.015 (0.023)	-0.033 (0.034)	0.048 (0.038)	0.245 (0.1)	-0.002 (0.067)	DBP
rs77924615	16	20392332	0,90	G	A	0.022 (0.003)	0.053 (0.024)	0.078 (0.036)	0.039 (0.039)	-0.032 (0.101)	0.042 (0.066)	DBP
rs11640961	16	30979818	0,91	C	T	0.016 (0.002)	-0.008 (0.019)	-0.011 (0.03)	-0.011 (0.031)	-0.007 (0.083)	0.01 (0.052)	DBP
rs28545584	16	69329590	1,00	G	A	0.017 (0.003)	0.005 (0.021)	-0.011 (0.032)	-0.003 (0.035)	0.088 (0.089)	0.049 (0.059)	DBP
rs12929303	16	81602264	0,99	A	G	0.015 (0.002)	0.041 (0.018)	0.041 (0.027)	0.03 (0.03)	0.052 (0.077)	0.065 (0.051)	DBP
rs7500448	16	83045790	0,98	G	A	0.017 (0.003)	0.023 (0.021)	0.042 (0.031)	0.004 (0.034)	-0.013 (0.092)	0.026 (0.06)	DBP
rs142426495	17	2161604	0,98	C	T	0.016 (0.002)	-0.016 (0.019)	-0.015 (0.029)	-0.039 (0.031)	-0.092 (0.084)	0.083 (0.054)	DBP
rs2603027	17	3944768	1,00	T	C	0.015 (0.002)	-0.009 (0.018)	0.008 (0.027)	-0.012 (0.03)	0.016 (0.08)	-0.073 (0.053)	DBP
rs35552228	17	4626318	0,80	G	T	0.016 (0.003)	-0.018 (0.024)	-0.016 (0.036)	-0.011 (0.039)	0.088 (0.101)	-0.092 (0.066)	DBP

rs3826408	17	7101292	1,00	T	C	0.014 (0.002)	0.022 (0.018)	0.007 (0.027)	0.025 (0.03)	0.156 (0.078)	0.007 (0.052)	DBP
rs74439044	17	7781019	0,99	C	T	0.026 (0.004)	-0.017 (0.03)	-0.015 (0.043)	-0.056 (0.049)	0.344 (0.138)	-0.052 (0.088)	DBP
rs2301597	17	43173273	0,99	T	C	0.017 (0.002)	0.002 (0.018)	-0.021 (0.027)	0.042 (0.03)	-0.002 (0.078)	-0.03 (0.052)	DBP
rs55688304	17	46587273	0,88	C	A	0.019 (0.003)	-0.016 (0.027)	0.023 (0.04)	-0.06 (0.044)	-0.034 (0.117)	-0.016 (0.076)	DBP
rs12940887	17	47402807	0,98	T	C	0.016 (0.002)	-0.02 (0.019)	-0.047 (0.028)	0.021 (0.031)	-0.16 (0.081)	0.019 (0.053)	DBP
rs59549190	17	57384444	0,84	G	A	0.015 (0.002)	0.034 (0.021)	0.069 (0.032)	0.013 (0.034)	-0.076 (0.091)	0.02 (0.058)	DBP
rs147295858	17	61298602	0,88	G	A	0.08 (0.012)	-0.101 (0.072)	-0.14 (0.111)	-0.142 (0.112)	0.224 (0.291)	0.022 (0.22)	DBP
rs1384367	17	76729757	1,00	C	A	0.013 (0.002)	-0.02 (0.018)	-0.039 (0.027)	-0.014 (0.03)	0.055 (0.078)	-0.006 (0.051)	DBP
rs56358311	18	42207147	0,98	G	A	0.015 (0.003)	-0.006 (0.02)	0.002 (0.031)	-0.001 (0.033)	-0.076 (0.087)	-0.024 (0.058)	DBP
rs1437649	18	48132646	0,98	G	A	0.016 (0.003)	0.028 (0.021)	0.02 (0.032)	0.04 (0.035)	0.019 (0.092)	0.022 (0.061)	DBP
rs36047283	19	7255701	0,84	A	G	0.031 (0.003)	-0.037 (0.031)	-0.027 (0.043)	-0.013 (0.053)	-0.16 (0.118)	NA (NA)	DBP
rs997669	19	30304483	1,00	C	T	0.023 (0.002)	0.039 (0.018)	0.058 (0.027)	0.013 (0.03)	-0.05 (0.08)	0.079 (0.052)	DBP
rs2032905	19	31905568	0,99	A	G	0.015 (0.002)	0.057 (0.018)	0.077 (0.028)	0.035 (0.03)	0.009 (0.078)	0.072 (0.052)	DBP
rs62107917	19	41136876	0,92	A	G	0.018 (0.003)	0.054 (0.024)	0.055 (0.036)	0.001 (0.04)	0.071 (0.108)	0.214 (0.071)	DBP
rs143273199	19	45744552	0,94	C	T	0.018 (0.003)	0.013 (0.021)	0.075 (0.032)	-0.011 (0.035)	-0.074 (0.09)	-0.084 (0.06)	DBP
rs73046792	19	49605705	0,90	G	A	0.018 (0.003)	0.002 (0.027)	0.058 (0.042)	-0.058 (0.043)	0.022 (0.112)	0.003 (0.071)	DBP
rs1051419	20	10620386	0,95	A	G	0.016 (0.002)	0.002 (0.019)	-0.013 (0.028)	0.04 (0.032)	0.021 (0.082)	-0.055 (0.055)	DBP
rs77098653	20	10742578	0,96	G	A	0.028 (0.003)	0.03 (0.021)	0.079 (0.031)	0.016 (0.034)	-0.187 (0.087)	0.002 (0.059)	DBP
rs1887320	20	10965998	1,00	A	G	0.024 (0.002)	-0.016 (0.018)	-0.027 (0.027)	0 (0.03)	0.056 (0.078)	-0.05 (0.051)	DBP
rs3827986	20	19475014	1,00	G	A	0.015 (0.003)	-0.033 (0.02)	-0.059 (0.03)	-0.011 (0.034)	-0.045 (0.089)	0.009 (0.059)	DBP
rs4810833	20	47226565	0,97	A	G	0.014 (0.002)	-0.055 (0.019)	-0.067 (0.028)	-0.057 (0.03)	-0.002 (0.081)	-0.03 (0.052)	DBP
rs78473917	20	47420709	1,00	T	C	0.021 (0.003)	-0.037 (0.025)	-0.055 (0.038)	-0.057 (0.042)	-0.034 (0.109)	0.088 (0.072)	DBP
rs6026748	20	57745815	0,98	A	G	0.03 (0.004)	-0.031 (0.027)	-0.071 (0.04)	-0.039 (0.045)	0.004 (0.124)	0.141 (0.081)	DBP
rs816943	20	62676009	0,97	G	A	0.014 (0.002)	0.008 (0.018)	-0.001 (0.027)	0.054 (0.03)	0 (0.078)	-0.09 (0.052)	DBP
rs35213536	20	62694319	0,97	T	G	0.017 (0.003)	-0.021 (0.021)	-0.044 (0.031)	0.017 (0.035)	-0.022 (0.088)	-0.043 (0.06)	DBP
rs28451064	21	35593827	0,88	A	G	0.021 (0.003)	-0.025 (0.029)	-0.019 (0.042)	0.005 (0.047)	-0.142 (0.125)	-0.088 (0.08)	DBP
rs2835510	21	38237500	0,99	A	G	0.013 (0.002)	0.002 (0.018)	0.022 (0.027)	-0.028 (0.03)	0.01 (0.077)	0.021 (0.051)	DBP
rs2836411	21	39819830	0,98	T	C	0.018 (0.002)	-0.006 (0.019)	0.022 (0.029)	0.005 (0.031)	-0.121 (0.08)	-0.091 (0.054)	DBP
rs463530	21	40023430	0,97	G	A	0.017 (0.003)	0.006 (0.022)	0.018 (0.033)	0.017 (0.036)	-0.009 (0.094)	-0.066 (0.062)	DBP
rs2007946	22	18481510	0,92	G	A	0.015 (0.003)	-0.047 (0.022)	-0.024 (0.033)	-0.058 (0.034)	-0.146 (0.089)	-0.04 (0.06)	DBP
rs134086	22	28067235	0,98	T	C	0.015 (0.002)	-0.01 (0.018)	-0.06 (0.028)	0.055 (0.03)	-0.098 (0.079)	0.014 (0.052)	DBP
rs2294239	22	29449477	1,00	A	G	0.014 (0.002)	0.004 (0.018)	0.01 (0.027)	-0.002 (0.03)	0.034 (0.079)	-0.018 (0.052)	DBP
rs1807579	22	40528220	0,97	T	C	0.017 (0.003)	0.018 (0.023)	0.024 (0.034)	0.037 (0.037)	-0.039 (0.097)	-0.034 (0.065)	DBP
rs2490543	1	1879084	0,82	C	T	0.014 (0.002)	0.013 (0.02)	0.034 (0.03)	0.001 (0.031)	0.056 (0.083)	-0.034 (0.054)	SBP
rs2493288	1	3330884	0,94	A	G	0.021 (0.003)	0.009 (0.026)	0.067 (0.039)	-0.069 (0.043)	0.032 (0.113)	0.016 (0.076)	SBP
rs302714	1	8486131	1,00	C	A	0.014 (0.002)	-0.033 (0.019)	-0.062 (0.029)	-0.001 (0.032)	0.043 (0.082)	-0.06 (0.054)	SBP
rs2153304	1	9441586	0,99	A	G	0.014 (0.002)	-0.007 (0.018)	0.021 (0.027)	-0.008 (0.03)	-0.021 (0.076)	-0.103 (0.052)	SBP
rs284278	1	10790536	0,94	A	G	0.019 (0.002)	-0.006 (0.019)	-0.013 (0.029)	-0.027 (0.032)	0.031 (0.084)	0.065 (0.054)	SBP
rs12567136	1	11883731	1,00	C	T	0.039 (0.003)	0.006 (0.025)	0.006 (0.038)	-0.016 (0.041)	-0.126 (0.103)	0.126 (0.069)	SBP
rs55892892	1	11896856	1,00	C	A	0.063 (0.005)	-0.015 (0.04)	-0.07 (0.058)	0.053 (0.066)	-0.239 (0.171)	0.083 (0.112)	SBP
rs3753581	1	11920189	0,99	C	A	0.025 (0.002)	0.01 (0.019)	0.022 (0.028)	-0.012 (0.031)	-0.169 (0.082)	0.106 (0.054)	SBP
rs71654213	1	42389815	0,92	C	T	0.016 (0.002)	0.008 (0.02)	0.007 (0.029)	0.032 (0.032)	-0.08 (0.084)	-0.019 (0.056)	SBP
rs2782652	1	43926305	1,00	C	T	0.016 (0.002)	0.008 (0.018)	-0.015 (0.027)	-0.026 (0.03)	0.12 (0.078)	0.141 (0.052)	SBP
rs1230004	1	56614762	1,00	T	C	0.015 (0.002)	-0.026 (0.018)	-0.02 (0.027)	-0.013 (0.03)	-0.026 (0.079)	-0.092 (0.053)	SBP
rs17535443	1	59646056	0,99	G	A	0.02 (0.003)	0.01 (0.021)	-0.005 (0.032)	0.024 (0.034)	-0.12 (0.088)	0.074 (0.057)	SBP
rs7553325	1	67010104	1,00	T	C	0.014 (0.002)	-0.016 (0.018)	-0.01 (0.027)	-0.017 (0.03)	-0.084 (0.077)	-0.004 (0.052)	SBP
rs430600	1	89225976	1,00	T	C	0.016 (0.002)	0.008 (0.019)	0.023 (0.028)	0.017 (0.031)	-0.022 (0.079)	-0.055 (0.052)	SBP
rs10776752	1	113044328	0,96	T	G	0.033 (0.004)	0.061 (0.034)	0.084 (0.049)	0.033 (0.057)	0.112 (0.153)	0.024 (0.105)	SBP
rs351365	1	113046395	0,92	C	T	0.019 (0.003)	-0.004 (0.021)	-0.008 (0.032)	0.072 (0.035)	-0.138 (0.093)	-0.17 (0.062)	SBP

rs6657049	1	115825531	0,99	A	G	0.016 (0.002)	0.02 (0.019)	0.035 (0.028)	-0.003 (0.031)	0.042 (0.081)	0.028 (0.054)	SBP
rs4639796	1	197126649	0,99	A	G	0.019 (0.003)	-0.014 (0.025)	-0.022 (0.037)	0.024 (0.04)	-0.187 (0.104)	-0.022 (0.071)	SBP
rs2004776	1	230848702	1,00	T	C	0.017 (0.003)	-0.056 (0.021)	-0.049 (0.031)	-0.066 (0.035)	0.009 (0.09)	-0.078 (0.06)	SBP
rs12714414	2	651407	0,98	T	C	0.019 (0.003)	-0.011 (0.024)	-0.045 (0.036)	-0.014 (0.04)	0.124 (0.104)	0.061 (0.069)	SBP
rs17759661	2	19729795	1,00	A	C	0.017 (0.002)	0.01 (0.018)	-0.017 (0.027)	0.027 (0.03)	0.03 (0.077)	0.046 (0.051)	SBP
rs2196792	2	25259953	0,99	G	A	0.017 (0.003)	0.024 (0.021)	0.025 (0.031)	0.052 (0.035)	-0.003 (0.093)	-0.05 (0.06)	SBP
rs1275984	2	26911509	0,99	A	C	0.023 (0.002)	-0.041 (0.019)	-0.053 (0.027)	-0.028 (0.031)	0.043 (0.081)	-0.07 (0.052)	SBP
rs6734118	2	37559355	1,00	C	A	0.019 (0.003)	0.013 (0.022)	-0.014 (0.032)	-0.014 (0.036)	0.074 (0.089)	0.166 (0.063)	SBP
rs6713844	2	43750265	1,00	A	G	0.015 (0.002)	-0.024 (0.018)	-0.021 (0.028)	-0.032 (0.03)	-0.043 (0.079)	-0.004 (0.052)	SBP
rs6733889	2	56199062	1,00	T	C	0.016 (0.002)	-0.002 (0.018)	-0.038 (0.028)	0.027 (0.03)	0.029 (0.08)	0.023 (0.053)	SBP
rs2249105	2	65287896	1,00	A	G	0.017 (0.002)	-0.017 (0.018)	0.002 (0.027)	0.002 (0.031)	-0.042 (0.08)	-0.136 (0.053)	SBP
rs1530558	2	135755616	0,73	T	C	0.02 (0.003)	0.009 (0.028)	0.031 (0.037)	NA (NA)	NA (NA)	NA (NA)	SBP
rs76591930	2	159540995	0,99	C	T	0.019 (0.003)	-0.04 (0.023)	-0.069 (0.035)	-0.026 (0.038)	0.049 (0.098)	-0.009 (0.068)	SBP
rs13026245	2	164936215	0,98	C	T	0.033 (0.003)	-0.009 (0.024)	-0.051 (0.037)	0.028 (0.04)	0.036 (0.098)	0.003 (0.069)	SBP
rs6726740	2	165050128	0,99	T	G	0.028 (0.003)	-0.003 (0.02)	-0.018 (0.031)	0.017 (0.033)	0.133 (0.086)	-0.068 (0.058)	SBP
rs7591820	2	177028144	1,00	T	G	0.015 (0.002)	-0.017 (0.02)	-0.026 (0.029)	-0.01 (0.032)	-0.002 (0.083)	-0.01 (0.055)	SBP
rs6732308	2	182996188	0,98	A	G	0.017 (0.003)	-0.035 (0.023)	-0.036 (0.034)	-0.034 (0.038)	-0.177 (0.1)	0.022 (0.063)	SBP
rs7570988	2	191560453	0,97	A	G	0.014 (0.002)	0 (0.02)	0.006 (0.031)	-0.022 (0.033)	-0.118 (0.088)	0.087 (0.056)	SBP
rs1250258	2	216300185	0,97	C	T	0.019 (0.003)	0.005 (0.021)	-0.036 (0.031)	0.07 (0.034)	0.009 (0.092)	-0.043 (0.06)	SBP
rs2447607	3	11552027	0,97	T	C	0.014 (0.002)	-0.036 (0.019)	-0.074 (0.028)	-0.003 (0.031)	-0.022 (0.078)	-0.011 (0.053)	SBP
rs1687318	3	14891798	0,99	T	C	0.016 (0.003)	0.025 (0.02)	0.045 (0.03)	0.008 (0.034)	0.096 (0.086)	-0.029 (0.057)	SBP
rs2643826	3	27562988	0,96	T	C	0.017 (0.002)	0.012 (0.018)	0.034 (0.028)	0.006 (0.03)	-0.139 (0.08)	0.019 (0.052)	SBP
rs6768542	3	41865474	0,93	G	A	0.021 (0.003)	-0.053 (0.026)	-0.021 (0.039)	-0.113 (0.042)	-0.037 (0.104)	0.008 (0.072)	SBP
rs9869147	3	124614317	0,98	C	T	0.015 (0.002)	-0.02 (0.018)	-0.045 (0.027)	-0.012 (0.03)	0.003 (0.079)	0.034 (0.052)	SBP
rs11915143	3	133985901	0,99	A	G	0.014 (0.002)	-0.021 (0.018)	-0.01 (0.027)	-0.034 (0.03)	0.052 (0.08)	-0.057 (0.052)	SBP
rs4955658	3	169171347	0,99	T	G	0.016 (0.002)	0.029 (0.018)	0.023 (0.027)	0.044 (0.03)	-0.024 (0.079)	0.032 (0.052)	SBP
rs979532	4	17901679	0,97	T	C	0.015 (0.003)	0.03 (0.021)	0.011 (0.032)	0.064 (0.034)	-0.041 (0.087)	0.027 (0.058)	SBP
rs35107212	4	26799390	0,99	G	A	0.014 (0.002)	0.016 (0.018)	0.004 (0.028)	-0.008 (0.03)	0.058 (0.078)	0.111 (0.052)	SBP
rs13125101	4	81174592	0,99	A	G	0.037 (0.003)	-0.034 (0.02)	-0.038 (0.029)	-0.032 (0.032)	-0.022 (0.086)	-0.032 (0.056)	SBP
rs72978881	4	86737468	0,99	G	A	0.028 (0.004)	0.013 (0.03)	-0.006 (0.045)	0.04 (0.049)	0.077 (0.13)	-0.025 (0.083)	SBP
rs1229984	4	100239319	0,79	C	T	0.052 (0.008)	0.025 (0.047)	-0.001 (0.076)	0.072 (0.072)	0.076 (0.197)	-0.095 (0.14)	SBP
rs4835266	4	146821725	0,92	T	C	0.016 (0.002)	0.038 (0.019)	0.028 (0.028)	0.031 (0.032)	-0.044 (0.082)	0.131 (0.054)	SBP
rs13143677	4	148419040	0,98	A	G	0.015 (0.003)	0.009 (0.02)	0.02 (0.03)	0.024 (0.033)	-0.039 (0.085)	-0.055 (0.056)	SBP
rs17033041	4	156391307	0,91	G	A	0.022 (0.003)	0.006 (0.024)	0.04 (0.036)	-0.032 (0.04)	0.098 (0.106)	-0.043 (0.068)	SBP
rs2341599	4	156433308	1,00	G	A	0.019 (0.002)	0.008 (0.019)	-0.017 (0.028)	0.02 (0.032)	0.036 (0.081)	0.057 (0.054)	SBP
rs3762988	5	32709653	0,99	C	T	0.028 (0.002)	-0.006 (0.018)	-0.012 (0.027)	0.02 (0.03)	-0.094 (0.078)	-0.023 (0.053)	SBP
rs12656497	5	32831939	0,99	C	T	0.032 (0.002)	0.035 (0.018)	0.028 (0.027)	0.073 (0.031)	-0.004 (0.08)	-0.034 (0.052)	SBP
rs17389148	5	77869903	0,99	C	A	0.013 (0.002)	-0.01 (0.018)	-0.022 (0.027)	-0.021 (0.03)	0.085 (0.077)	0.02 (0.052)	SBP
rs158172	5	107424308	0,99	A	G	0.018 (0.003)	0.008 (0.022)	-0.009 (0.033)	0.025 (0.036)	0.086 (0.096)	-0.013 (0.063)	SBP
rs1422279	5	122470209	1,00	T	C	0.02 (0.002)	-0.019 (0.018)	-0.025 (0.027)	-0.035 (0.03)	0.028 (0.079)	0.034 (0.053)	SBP
rs34790038	5	127862595	0,97	C	A	0.019 (0.002)	-0.027 (0.021)	-0.044 (0.031)	0.011 (0.034)	-0.193 (0.087)	-0.005 (0.056)	SBP
rs2192256	5	148333934	0,99	T	C	0.014 (0.002)	0.036 (0.02)	0.015 (0.029)	0.06 (0.032)	0.049 (0.081)	0.033 (0.055)	SBP
rs13436194	5	157803588	0,98	A	G	0.018 (0.002)	-0.007 (0.018)	0.007 (0.027)	-0.011 (0.03)	0.06 (0.079)	-0.082 (0.054)	SBP
rs7736883	5	158343969	1,00	G	A	0.017 (0.002)	0.016 (0.018)	0.036 (0.027)	0.009 (0.03)	0.048 (0.08)	-0.056 (0.052)	SBP
rs9349379	6	12903957	0,95	A	G	0.014 (0.002)	-0.03 (0.019)	-0.04 (0.029)	-0.014 (0.03)	-0.048 (0.078)	-0.032 (0.053)	SBP
rs1543310	6	22110418	0,92	C	T	0.014 (0.002)	-0.01 (0.019)	-0.028 (0.028)	0.004 (0.031)	-0.016 (0.081)	0.017 (0.053)	SBP
rs6918586	6	26097384	1,00	C	T	0.017 (0.002)	-0.034 (0.018)	-0.031 (0.027)	0.009 (0.03)	-0.075 (0.079)	-0.158 (0.053)	SBP
rs3130482	6	31839782	0,99	A	C	0.017 (0.002)	0.059 (0.018)	0.035 (0.027)	0.053 (0.03)	0.15 (0.079)	0.128 (0.052)	SBP
rs4151657	6	31917540	1,00	C	T	0.015 (0.002)	0.025 (0.019)	-0.006 (0.027)	0.028 (0.031)	0.072 (0.083)	0.125 (0.055)	SBP

rs2395622	6	35388758	0,99	C	T	0.017 (0.003)	-0.018 (0.024)	-0.016 (0.036)	-0.014 (0.039)	-0.067 (0.099)	-0.014 (0.065)	SBP
rs1925148	6	56095672	0,97	A	G	0.015 (0.002)	-0.023 (0.018)	-0.019 (0.028)	-0.032 (0.03)	-0.033 (0.081)	-0.008 (0.051)	SBP
rs7743378	6	58056014	0,95	C	T	0.015 (0.002)	0.017 (0.019)	0.042 (0.029)	-0.006 (0.031)	0.023 (0.08)	-0.004 (0.056)	SBP
rs4133870	6	62064508	0,99	A	G	0.015 (0.002)	0.016 (0.019)	0.036 (0.028)	-0.008 (0.031)	0.036 (0.08)	0.003 (0.053)	SBP
rs1931814	6	62589167	0,99	G	A	0.013 (0.002)	0.001 (0.018)	0.006 (0.027)	-0.002 (0.03)	0.088 (0.078)	-0.049 (0.051)	SBP
rs2971608	6	97039757	0,96	C	T	0.021 (0.003)	-0.045 (0.022)	-0.045 (0.034)	-0.04 (0.036)	-0.095 (0.091)	-0.035 (0.062)	SBP
rs6923947	6	127098553	0,97	A	G	0.024 (0.002)	0.003 (0.018)	-0.009 (0.028)	0.025 (0.03)	0.003 (0.078)	-0.016 (0.053)	SBP
rs59155780	6	151008874	0,99	C	A	0.031 (0.004)	-0.011 (0.037)	-0.016 (0.056)	0.014 (0.06)	-0.157 (0.155)	-0.009 (0.098)	SBP
rs9340985	6	152341587	1,00	C	T	0.022 (0.004)	-0.024 (0.028)	-0.054 (0.041)	-0.009 (0.046)	-0.011 (0.127)	0.045 (0.082)	SBP
rs434578	6	159693220	0,97	C	T	0.02 (0.003)	-0.034 (0.027)	-0.031 (0.041)	0.004 (0.045)	-0.24 (0.115)	-0.065 (0.075)	SBP
rs2074633	7	19035920	1,00	C	T	0.022 (0.003)	0.003 (0.022)	0.025 (0.032)	0.002 (0.035)	-0.196 (0.096)	0.01 (0.064)	SBP
rs2529055	7	24590331	0,99	A	G	0.017 (0.003)	0.039 (0.024)	0.004 (0.037)	0.075 (0.04)	0.138 (0.103)	0.014 (0.067)	SBP
rs2023843	7	27243221	1,00	T	C	0.038 (0.004)	0.04 (0.033)	-0.009 (0.048)	0.068 (0.056)	0.102 (0.142)	0.127 (0.098)	SBP
rs2204489	7	46002020	0,99	C	A	0.016 (0.002)	-0.021 (0.018)	0.007 (0.027)	-0.027 (0.03)	-0.016 (0.076)	-0.105 (0.051)	SBP
rs42032	7	92237426	0,97	G	A	0.02 (0.003)	-0.031 (0.02)	-0.022 (0.031)	-0.044 (0.033)	-0.014 (0.086)	-0.031 (0.059)	SBP
rs2392929	7	106414069	0,96	G	T	0.045 (0.003)	-0.02 (0.023)	-0.028 (0.034)	0.004 (0.037)	-0.123 (0.097)	-0.02 (0.066)	SBP
rs1813742	7	131006229	0,99	T	C	0.017 (0.002)	0.033 (0.018)	0.03 (0.027)	0.042 (0.03)	0.033 (0.08)	0.017 (0.052)	SBP
rs891511	7	150704843	0,88	G	A	0.015 (0.002)	0.006 (0.02)	0.028 (0.031)	0.006 (0.032)	-0.032 (0.092)	-0.049 (0.055)	SBP
rs10265221	7	151414329	0,92	C	T	0.015 (0.003)	-0.022 (0.021)	-0.022 (0.031)	-0.048 (0.035)	0.094 (0.088)	0 (0.059)	SBP
rs1915986	8	8114543	0,96	G	A	0.014 (0.002)	0.01 (0.018)	0.043 (0.027)	-0.025 (0.03)	0.002 (0.08)	-0.004 (0.053)	SBP
rs7013471	8	8687325	0,98	A	G	0.017 (0.002)	0.007 (0.018)	0.047 (0.027)	-0.038 (0.03)	-0.076 (0.078)	0.034 (0.052)	SBP
rs4075359	8	9487813	0,97	T	C	0.014 (0.002)	0.031 (0.019)	0.069 (0.029)	-0.009 (0.031)	-0.021 (0.082)	0.036 (0.054)	SBP
rs7017349	8	10077241	0,97	A	G	0.015 (0.002)	0.012 (0.018)	0.036 (0.027)	0 (0.03)	-0.151 (0.081)	0.032 (0.052)	SBP
rs7814142	8	10637552	1,00	G	A	0.02 (0.002)	0.009 (0.018)	0.032 (0.027)	-0.02 (0.03)	-0.05 (0.08)	0.035 (0.053)	SBP
rs13278982	8	11589033	0,99	A	G	0.018 (0.002)	-0.006 (0.019)	0.036 (0.028)	-0.053 (0.031)	-0.072 (0.082)	0.008 (0.054)	SBP
rs7838131	8	11596163	0,99	G	A	0.019 (0.002)	0.014 (0.018)	0.053 (0.027)	-0.033 (0.03)	-0.084 (0.079)	0.058 (0.052)	SBP
rs73563812	8	25900405	0,99	G	T	0.019 (0.003)	0.003 (0.021)	0.014 (0.031)	-0.039 (0.035)	0.04 (0.09)	0.069 (0.06)	SBP
rs72649639	8	51891208	0,99	G	A	0.022 (0.003)	-0.021 (0.024)	-0.037 (0.034)	-0.038 (0.039)	0.042 (0.103)	0.073 (0.069)	SBP
rs11778153	8	64503942	0,98	T	C	0.015 (0.002)	0.004 (0.019)	0.024 (0.029)	-0.035 (0.032)	-0.072 (0.082)	0.081 (0.054)	SBP
rs3901080	8	81410116	0,99	T	G	0.019 (0.003)	-0.042 (0.022)	-0.053 (0.033)	-0.036 (0.037)	-0.033 (0.098)	-0.026 (0.064)	SBP
rs2470004	8	120358445	0,99	C	T	0.02 (0.003)	0.007 (0.024)	0.013 (0.036)	0.021 (0.039)	-0.068 (0.101)	-0.019 (0.066)	SBP
rs113656763	8	135573096	0,88	G	A	0.039 (0.006)	-0.01 (0.059)	-0.073 (0.089)	-0.006 (0.098)	0.236 (0.243)	0.073 (0.16)	SBP
rs11997254	8	141083381	0,98	T	G	0.015 (0.002)	0.041 (0.018)	0.022 (0.027)	0.059 (0.03)	0.164 (0.079)	0.005 (0.052)	SBP
rs62560889	9	34171499	0,97	A	G	0.018 (0.003)	-0.029 (0.023)	-0.015 (0.034)	-0.029 (0.038)	-0.086 (0.099)	-0.058 (0.067)	SBP
rs10817007	9	113155247	0,97	G	T	0.02 (0.003)	0.035 (0.028)	0.017 (0.041)	0.039 (0.046)	-0.04 (0.121)	0.121 (0.079)	SBP
rs10818962	9	127182293	0,99	C	G	0.014 (0.002)	0.022 (0.019)	0.041 (0.028)	-0.008 (0.031)	0.058 (0.08)	0.029 (0.052)	SBP
rs72765275	9	127856566	1,00	G	A	0.02 (0.003)	-0.009 (0.027)	0.001 (0.04)	-0.048 (0.044)	-0.077 (0.115)	0.105 (0.078)	SBP
rs10819049	9	128139084	0,99	C	T	0.014 (0.002)	-0.018 (0.018)	-0.015 (0.027)	-0.002 (0.03)	-0.139 (0.077)	-0.023 (0.051)	SBP
rs10764331	10	18451836	1,00	G	A	0.017 (0.002)	0.032 (0.018)	0.05 (0.027)	0.026 (0.03)	-0.047 (0.077)	0.014 (0.052)	SBP
rs7070847	10	18726054	0,94	G	A	0.025 (0.003)	-0.056 (0.02)	-0.065 (0.03)	-0.041 (0.034)	-0.12 (0.089)	-0.041 (0.058)	SBP
rs7070797	10	63551773	0,99	G	A	0.029 (0.003)	0.009 (0.027)	0.005 (0.04)	0.031 (0.043)	-0.202 (0.113)	0.053 (0.074)	SBP
rs11527181	10	64546164	0,96	A	G	0.016 (0.002)	-0.011 (0.019)	0.009 (0.028)	-0.028 (0.031)	0 (0.079)	-0.035 (0.053)	SBP
rs11000734	10	75419663	0,99	G	T	0.021 (0.003)	0.014 (0.025)	0.027 (0.037)	-0.037 (0.041)	0 (0.107)	0.127 (0.073)	SBP
rs11187793	10	95896716	0,95	A	G	0.019 (0.002)	-0.006 (0.019)	-0.012 (0.028)	0.009 (0.031)	0.032 (0.08)	-0.047 (0.053)	SBP
rs11187838	10	96038686	0,99	G	A	0.022 (0.002)	0.053 (0.018)	0.039 (0.027)	0.071 (0.03)	0.024 (0.078)	0.064 (0.052)	SBP
rs11188112	10	96666699	0,99	C	T	0.018 (0.003)	0.031 (0.022)	0.021 (0.032)	0.052 (0.035)	0.036 (0.092)	0 (0.061)	SBP
rs10883543	10	102552752	1,00	T	G	0.031 (0.004)	-0.06 (0.029)	-0.033 (0.043)	-0.056 (0.048)	-0.071 (0.122)	-0.158 (0.079)	SBP
rs11191580	10	104906211	0,99	T	C	0.046 (0.004)	0.006 (0.032)	0.004 (0.047)	0.012 (0.052)	-0.264 (0.136)	0.125 (0.095)	SBP
rs11191841	10	105639611	0,99	T	C	0.014 (0.002)	0.071 (0.018)	0.069 (0.027)	0.08 (0.03)	-0.003 (0.077)	0.083 (0.051)	SBP

rs151599	10	115724039	0,99	A	G	0.014 (0.002)	-0.02 (0.019)	-0.033 (0.029)	-0.008 (0.032)	0.007 (0.082)	-0.021 (0.055)	SBP
rs2782980	10	115781527	0,98	C	T	0.018 (0.003)	0.017 (0.02)	0.006 (0.03)	0.032 (0.033)	0.124 (0.083)	-0.039 (0.058)	SBP
rs72830615	10	122988575	0,99	A	G	0.013 (0.002)	-0.004 (0.019)	-0.007 (0.028)	-0.001 (0.03)	0.094 (0.079)	-0.044 (0.052)	SBP
rs587961	11	1881256	0,92	C	T	0.025 (0.002)	0.014 (0.02)	0.007 (0.031)	-0.004 (0.032)	0.088 (0.084)	0.06 (0.055)	SBP
rs612652	11	1887216	0,95	C	T	0.025 (0.002)	0.013 (0.019)	-0.002 (0.029)	0.037 (0.031)	0.045 (0.079)	-0.018 (0.052)	SBP
rs360153	11	9762274	1,00	C	T	0.014 (0.002)	0.018 (0.018)	0.031 (0.027)	0.023 (0.03)	-0.08 (0.079)	-0.002 (0.052)	SBP
rs143175535	11	9953750	0,97	C	T	0.027 (0.004)	0.059 (0.032)	0.097 (0.048)	0.02 (0.053)	-0.035 (0.134)	0.079 (0.086)	SBP
rs56352102	11	10268593	1,00	T	C	0.025 (0.003)	0.026 (0.023)	0.035 (0.034)	0.002 (0.038)	0.065 (0.098)	0.043 (0.067)	SBP
rs2014408	11	16365282	0,98	T	C	0.017 (0.003)	0.035 (0.023)	0.094 (0.035)	-0.017 (0.037)	-0.021 (0.094)	0.018 (0.063)	SBP
rs11605215	11	30443248	0,99	A	G	0.017 (0.003)	-0.013 (0.022)	-0.003 (0.033)	-0.005 (0.036)	0.007 (0.094)	-0.082 (0.063)	SBP
rs72910075	11	46359645	0,86	T	C	0.023 (0.004)	0.061 (0.031)	0.036 (0.048)	0.108 (0.049)	0.071 (0.129)	-0.005 (0.083)	SBP
rs72897626	11	46884447	0,93	T	C	0.021 (0.004)	0.042 (0.029)	0.007 (0.043)	0.09 (0.049)	0.051 (0.124)	0.024 (0.084)	SBP
rs7107356	11	47676170	0,99	G	A	0.018 (0.002)	0.026 (0.018)	0.024 (0.027)	0.058 (0.03)	-0.141 (0.076)	0.012 (0.051)	SBP
rs10128536	11	48678432	0,91	G	A	0.021 (0.003)	0.022 (0.028)	0.019 (0.045)	0.056 (0.044)	-0.201 (0.119)	0.022 (0.074)	SBP
rs10769517	11	48930941	0,80	T	C	0.021 (0.003)	0.03 (0.03)	NA (NA)	0.05 (0.043)	-0.177 (0.114)	0.027 (0.074)	SBP
rs12224222	11	49699461	0,87	A	G	0.018 (0.003)	0.046 (0.026)	0.032 (0.044)	0.074 (0.04)	-0.093 (0.105)	0.055 (0.067)	SBP
rs78067132	11	50200440	0,93	G	A	0.018 (0.003)	0.054 (0.026)	0.049 (0.045)	0.075 (0.04)	-0.098 (0.105)	0.063 (0.068)	SBP
rs3866801	11	50374138	0,98	A	G	0.018 (0.003)	0.052 (0.025)	0.045 (0.04)	0.076 (0.039)	-0.096 (0.105)	0.065 (0.067)	SBP
rs4881829	11	51492274	0,93	C	T	0.018 (0.003)	0.056 (0.026)	0.055 (0.042)	0.078 (0.04)	-0.102 (0.105)	0.063 (0.067)	SBP
rs495119	11	55277909	0,99	G	A	0.016 (0.003)	0.036 (0.022)	0.013 (0.032)	0.076 (0.035)	-0.015 (0.094)	0.014 (0.06)	SBP
rs2306363	11	65405600	0,97	G	T	0.016 (0.003)	-0.022 (0.023)	-0.008 (0.034)	-0.052 (0.037)	0.149 (0.095)	-0.056 (0.063)	SBP
rs1939212	11	65626701	0,99	T	C	0.014 (0.002)	-0.006 (0.02)	-0.026 (0.029)	-0.006 (0.032)	0.12 (0.087)	0.018 (0.056)	SBP
rs2289125	11	89224453	0,92	C	A	0.017 (0.003)	0.005 (0.023)	0.021 (0.034)	-0.005 (0.037)	-0.06 (0.1)	-0.001 (0.064)	SBP
rs604723	11	100610546	1,00	C	T	0.031 (0.003)	0.006 (0.02)	-0.03 (0.029)	0.059 (0.033)	-0.048 (0.088)	0.007 (0.058)	SBP
rs4754196	11	107096777	0,96	G	A	0.02 (0.002)	-0.004 (0.018)	0.019 (0.027)	-0.049 (0.03)	0.064 (0.076)	0.018 (0.053)	SBP
rs1940735	11	112908855	1,00	T	G	0.018 (0.003)	0.028 (0.02)	0.078 (0.03)	-0.044 (0.033)	-0.056 (0.088)	0.1 (0.059)	SBP
rs11220480	11	126277570	0,97	A	G	0.015 (0.003)	-0.051 (0.022)	-0.059 (0.033)	-0.003 (0.036)	-0.119 (0.091)	-0.128 (0.06)	SBP
rs2875238	11	130282078	0,92	T	C	0.02 (0.002)	0.001 (0.02)	0.013 (0.029)	0.017 (0.032)	0.002 (0.084)	-0.084 (0.055)	SBP
rs10770612	12	20230639	0,90	A	G	0.019 (0.003)	-0.023 (0.023)	-0.02 (0.034)	-0.077 (0.039)	0.085 (0.097)	0.085 (0.069)	SBP
rs60691990	12	20368269	0,98	T	C	0.018 (0.002)	0.023 (0.02)	0.035 (0.03)	0.017 (0.032)	-0.018 (0.082)	0.022 (0.054)	SBP
rs146336654	12	49305999	0,81	A	G	0.036 (0.006)	-0.077 (0.052)	NA (NA)	-0.066 (0.086)	-0.111 (0.222)	0.213 (0.144)	SBP
rs4883481	12	50574311	0,99	T	C	0.015 (0.002)	-0.016 (0.019)	-0.001 (0.028)	-0.037 (0.03)	-0.018 (0.082)	-0.006 (0.053)	SBP
rs67772913	12	54435716	0,98	A	G	0.017 (0.003)	0.01 (0.02)	0.017 (0.03)	0.009 (0.033)	0.014 (0.086)	-0.013 (0.056)	SBP
rs2619472	12	58059973	0,95	A	G	0.027 (0.004)	-0.037 (0.035)	0.017 (0.052)	-0.061 (0.057)	-0.31 (0.145)	-0.033 (0.098)	SBP
rs2681492	12	90013089	1,00	T	C	0.035 (0.003)	-0.008 (0.025)	-0.014 (0.037)	-0.013 (0.041)	-0.121 (0.104)	0.08 (0.068)	SBP
rs597808	12	111973358	0,92	A	G	0.02 (0.002)	-0.037 (0.019)	-0.057 (0.028)	0.011 (0.031)	-0.099 (0.08)	-0.085 (0.053)	SBP
rs17630235	12	112591686	0,89	A	G	0.017 (0.002)	-0.005 (0.02)	-0.025 (0.032)	0.04 (0.031)	-0.065 (0.08)	-0.057 (0.054)	SBP
rs10744835	12	115353849	0,99	G	A	0.016 (0.002)	0.009 (0.02)	0.053 (0.03)	-0.013 (0.033)	-0.009 (0.085)	-0.069 (0.057)	SBP
rs35427	12	115556307	0,91	T	G	0.016 (0.002)	-0.026 (0.02)	-0.051 (0.029)	-0.014 (0.033)	-0.011 (0.084)	0.033 (0.057)	SBP
rs9507870	13	27878484	1,00	C	T	0.023 (0.004)	0.018 (0.027)	0.013 (0.038)	0.008 (0.045)	0.112 (0.118)	0.026 (0.079)	SBP
rs11616710	13	115060768	0,85	T	C	0.024 (0.004)	0.06 (0.034)	0.103 (0.053)	0.018 (0.053)	-0.048 (0.136)	0.105 (0.094)	SBP
rs365990	14	23861811	1,00	A	G	0.016 (0.002)	-0.039 (0.019)	-0.053 (0.028)	-0.04 (0.031)	0.189 (0.081)	-0.084 (0.052)	SBP
rs9888615	14	53377540	0,97	C	T	0.016 (0.003)	0.012 (0.02)	0.01 (0.03)	0.001 (0.033)	-0.012 (0.085)	0.064 (0.058)	SBP
rs11160546	14	100233353	1,00	T	C	0.015 (0.002)	0.016 (0.019)	0.055 (0.029)	-0.022 (0.032)	-0.082 (0.082)	0.027 (0.054)	SBP
rs3736290	15	40321351	0,99	A	C	0.016 (0.002)	-0.017 (0.018)	-0.045 (0.027)	0.019 (0.03)	0.051 (0.078)	-0.048 (0.052)	SBP
rs2164996	15	41536624	0,99	C	T	0.015 (0.002)	0.001 (0.018)	0.002 (0.027)	-0.034 (0.03)	0.071 (0.078)	0.072 (0.051)	SBP
rs2472299	15	75033400	1,00	A	G	0.021 (0.003)	-0.046 (0.02)	-0.053 (0.029)	-0.033 (0.033)	-0.136 (0.086)	-0.016 (0.058)	SBP
rs2759315	15	81009646	0,99	A	C	0.018 (0.002)	-0.027 (0.018)	-0.044 (0.027)	-0.034 (0.03)	0.079 (0.076)	0.004 (0.051)	SBP
rs8027450	15	91418394	0,96	T	C	0.028 (0.002)	0.006 (0.02)	0.006 (0.028)	0.001 (0.033)	0.045 (0.085)	0.007 (0.056)	SBP

rs9926609	16	3540962	0,93	A	C	0.016 (0.003)	0.035 (0.021)	0.026 (0.031)	0.042 (0.036)	-0.025 (0.095)	0.077 (0.06)	SBP
rs8061324	16	3541490	0,92	T	G	0.017 (0.003)	0.037 (0.021)	0.029 (0.031)	0.041 (0.035)	-0.035 (0.095)	0.082 (0.06)	SBP
rs67081976	16	3731992	0,97	A	G	0.017 (0.003)	0.024 (0.023)	0.019 (0.034)	0.042 (0.038)	-0.01 (0.102)	0.004 (0.065)	SBP
rs2303083	16	24835168	0,99	G	A	0.021 (0.003)	0.026 (0.023)	0.001 (0.035)	0.04 (0.039)	0.03 (0.102)	0.074 (0.066)	SBP
rs35300112	16	60659812	0,98	A	C	0.017 (0.003)	0.015 (0.021)	0.043 (0.031)	0.002 (0.034)	-0.211 (0.089)	0.052 (0.059)	SBP
rs62053102	16	71654365	0,87	T	A	0.032 (0.006)	-0.118 (0.048)	-0.08 (0.079)	-0.079 (0.075)	-0.158 (0.2)	-0.293 (0.12)	SBP
rs62052918	16	72578594	0,99	G	T	0.029 (0.004)	-0.052 (0.036)	-0.01 (0.054)	-0.08 (0.059)	0.078 (0.15)	-0.165 (0.096)	SBP
rs1010630	16	75328591	0,97	T	G	0.016 (0.002)	-0.015 (0.018)	0.025 (0.027)	-0.052 (0.031)	-0.018 (0.078)	-0.051 (0.053)	SBP
rs10852858	17	1357751	0,88	T	C	0.015 (0.002)	-0.005 (0.019)	0.026 (0.03)	-0.002 (0.031)	-0.13 (0.08)	-0.052 (0.052)	SBP
rs4480845	17	1958609	0,95	T	C	0.02 (0.002)	-0.003 (0.019)	0.015 (0.028)	-0.017 (0.032)	-0.05 (0.082)	-0.009 (0.054)	SBP
rs11658881	17	2072949	1,00	A	G	0.019 (0.002)	-0.035 (0.018)	-0.019 (0.027)	-0.063 (0.03)	-0.165 (0.079)	0.047 (0.052)	SBP
rs5418	17	7185092	0,97	A	G	0.014 (0.002)	0.011 (0.019)	0.026 (0.028)	-0.002 (0.03)	0.089 (0.081)	-0.03 (0.052)	SBP
rs78744936	17	7461343	0,97	A	G	0.016 (0.003)	-0.068 (0.021)	-0.036 (0.031)	-0.124 (0.034)	0.109 (0.087)	-0.096 (0.059)	SBP
rs60603802	17	42665402	0,98	G	A	0.025 (0.004)	-0.016 (0.032)	-0.031 (0.048)	-0.014 (0.052)	-0.008 (0.132)	0.025 (0.086)	SBP
rs12603813	17	43196584	0,98	C	T	0.023 (0.003)	0.01 (0.021)	-0.003 (0.031)	0.025 (0.034)	0.125 (0.088)	-0.044 (0.06)	SBP
rs117368197	17	43715924	0,89	A	G	0.017 (0.003)	-0.036 (0.023)	-0.062 (0.037)	-0.024 (0.037)	0.014 (0.095)	-0.014 (0.06)	SBP
rs17608766	17	45013271	0,98	C	T	0.028 (0.003)	-0.017 (0.027)	0.018 (0.043)	-0.021 (0.044)	-0.142 (0.112)	-0.055 (0.072)	SBP
rs76635230	17	45938281	0,91	C	G	0.027 (0.005)	0.024 (0.039)	0.036 (0.063)	0.074 (0.061)	-0.234 (0.163)	-0.039 (0.098)	SBP
rs56011283	17	46553117	0,92	G	A	0.032 (0.005)	-0.002 (0.037)	0.052 (0.055)	-0.024 (0.06)	-0.013 (0.166)	-0.13 (0.106)	SBP
rs6504411	17	46672154	0,98	C	T	0.023 (0.004)	0.007 (0.029)	0.023 (0.044)	-0.007 (0.048)	0.146 (0.131)	-0.067 (0.086)	SBP
rs1000423	17	59475642	0,93	T	C	0.019 (0.003)	-0.013 (0.021)	0.005 (0.032)	-0.029 (0.034)	-0.014 (0.092)	-0.021 (0.059)	SBP
rs3786132	17	60768921	0,99	C	A	0.014 (0.002)	-0.035 (0.018)	-0.048 (0.027)	-0.007 (0.03)	-0.088 (0.081)	-0.051 (0.052)	SBP
rs2306526	17	76798362	0,99	C	T	0.015 (0.002)	-0.016 (0.018)	-0.028 (0.027)	-0.015 (0.03)	0.061 (0.078)	-0.009 (0.051)	SBP
rs9945184	18	42004527	0,99	A	G	0.017 (0.002)	-0.028 (0.019)	-0.026 (0.028)	-0.036 (0.032)	0.156 (0.084)	-0.089 (0.054)	SBP
rs10048404	18	54578482	0,91	C	T	0.016 (0.002)	-0.017 (0.02)	-0.04 (0.031)	0.011 (0.032)	0.12 (0.087)	-0.077 (0.053)	SBP
rs55678414	19	2177625	0,97	G	T	0.031 (0.005)	0.012 (0.035)	0 (0.051)	0.001 (0.059)	0.197 (0.159)	0.018 (0.108)	SBP
rs36047283	19	7255701	0,84	A	G	0.035 (0.003)	-0.037 (0.031)	-0.027 (0.043)	-0.013 (0.053)	-0.16 (0.118)	NA (NA)	SBP
rs12978472	19	7257990	0,82	C	G	0.036 (0.003)	-0.026 (0.032)	-0.028 (0.045)	0.018 (0.054)	-0.16 (0.12)	NA (NA)	SBP
rs10409243	19	10332988	1,00	C	T	0.014 (0.002)	-0.056 (0.018)	-0.064 (0.027)	-0.044 (0.03)	-0.163 (0.08)	-0.018 (0.052)	SBP
rs10853912	19	31865517	1,00	T	C	0.015 (0.002)	0.056 (0.018)	0.069 (0.027)	0.033 (0.031)	0.052 (0.079)	0.076 (0.053)	SBP
rs73046792	19	49605705	0,90	G	A	0.024 (0.003)	0.002 (0.027)	0.058 (0.042)	-0.058 (0.043)	0.022 (0.112)	0.003 (0.071)	SBP
rs2423514	20	10693337	0,99	A	G	0.019 (0.002)	-0.015 (0.018)	-0.02 (0.027)	0.014 (0.03)	-0.104 (0.076)	-0.041 (0.052)	SBP
rs1887320	20	10965998	1,00	A	G	0.019 (0.002)	-0.016 (0.018)	-0.027 (0.027)	0 (0.03)	0.056 (0.078)	-0.05 (0.051)	SBP
rs60770750	20	42766030	0,92	T	C	0.02 (0.003)	0.032 (0.028)	-0.064 (0.042)	0.111 (0.045)	0.214 (0.117)	0.038 (0.079)	SBP
rs6026740	20	57740348	0,99	G	A	0.026 (0.004)	-0.03 (0.027)	-0.073 (0.04)	-0.029 (0.045)	0 (0.123)	0.133 (0.08)	SBP
rs6062543	20	62451079	0,89	T	C	0.017 (0.003)	0.05 (0.022)	-0.008 (0.034)	0.1 (0.036)	0.015 (0.095)	0.106 (0.063)	SBP
rs307349	1	1262966	0,75	T	C	0.029 (0.005)	0.031 (0.04)	NA (NA)	0.036 (0.062)	0.272 (0.174)	-0.071 (0.103)	PP
rs2803340	1	1878071	0,84	C	T	0.015 (0.002)	0.012 (0.019)	0.032 (0.03)	0.001 (0.031)	0.043 (0.082)	-0.035 (0.053)	PP
rs9662255	1	9441949	0,99	C	A	0.018 (0.002)	-0.008 (0.018)	0.02 (0.027)	-0.008 (0.03)	-0.022 (0.076)	-0.104 (0.052)	PP
rs284278	1	10790536	0,94	A	G	0.015 (0.002)	-0.006 (0.019)	-0.013 (0.029)	-0.027 (0.032)	0.031 (0.084)	0.065 (0.054)	PP
rs12567136	1	11883731	1,00	C	T	0.027 (0.003)	0.006 (0.025)	0.006 (0.038)	-0.016 (0.041)	-0.126 (0.103)	0.126 (0.069)	PP
rs55892892	1	11896856	1,00	C	A	0.045 (0.005)	-0.015 (0.04)	-0.07 (0.058)	0.053 (0.066)	-0.239 (0.171)	0.083 (0.112)	PP
rs61776719	1	38461319	0,84	A	C	0.025 (0.002)	0.008 (0.02)	-0.001 (0.03)	0.012 (0.032)	-0.063 (0.09)	0.045 (0.052)	PP
rs11210568	1	42514452	0,99	T	C	0.016 (0.002)	0.027 (0.018)	0.008 (0.027)	0.047 (0.03)	0.018 (0.078)	0.038 (0.052)	PP
rs1757915	1	56615809	1,00	A	G	0.018 (0.002)	-0.024 (0.019)	-0.014 (0.027)	-0.017 (0.031)	-0.012 (0.079)	-0.087 (0.054)	PP
rs2151391	1	56916499	0,99	G	A	0.022 (0.004)	-0.009 (0.03)	0.045 (0.045)	-0.041 (0.048)	-0.018 (0.119)	-0.097 (0.082)	PP
rs17535443	1	59646056	0,99	G	A	0.035 (0.003)	0.01 (0.021)	-0.005 (0.032)	0.024 (0.034)	-0.12 (0.088)	0.074 (0.057)	PP
rs12144047	1	67010510	1,00	G	T	0.017 (0.003)	0.015 (0.022)	0.067 (0.033)	-0.022 (0.036)	-0.013 (0.094)	-0.055 (0.063)	PP
rs430600	1	89225976	1,00	T	C	0.015 (0.002)	0.008 (0.019)	0.023 (0.028)	0.017 (0.031)	-0.022 (0.079)	-0.055 (0.052)	PP

rs11204675	1	150570985	0,99	G	T	0.014 (0.002)	0.01 (0.018)	0.036 (0.027)	0.008 (0.03)	-0.016 (0.079)	-0.065 (0.052)	PP
rs12731646	1	169090660	0,98	C	T	0.018 (0.002)	0.025 (0.019)	0.023 (0.028)	0.049 (0.03)	-0.094 (0.08)	0.01 (0.052)	PP
rs12118102	1	176634724	0,99	A	G	0.034 (0.005)	-0.007 (0.04)	-0.019 (0.058)	-0.032 (0.067)	0.108 (0.174)	0.069 (0.12)	PP
rs536070	1	201748800	0,98	C	T	0.015 (0.002)	-0.025 (0.018)	-0.024 (0.028)	-0.025 (0.03)	-0.015 (0.077)	-0.029 (0.052)	PP
rs12063025	1	208123610	0,99	C	A	0.014 (0.002)	0.022 (0.019)	0.014 (0.028)	0.055 (0.031)	-0.1 (0.08)	0.011 (0.052)	PP
rs12133169	1	219792380	0,96	G	A	0.019 (0.003)	0.006 (0.022)	0.009 (0.033)	0.027 (0.036)	0.028 (0.094)	-0.076 (0.06)	PP
rs6721594	2	305203	0,89	C	T	0.015 (0.002)	0.009 (0.019)	0.039 (0.03)	-0.034 (0.03)	0.005 (0.079)	0.048 (0.054)	PP
rs7603740	2	19710771	0,98	A	G	0.026 (0.002)	0.009 (0.018)	-0.022 (0.028)	0.03 (0.03)	0.024 (0.077)	0.047 (0.052)	PP
rs17759661	2	19729795	1,00	A	C	0.026 (0.002)	0.01 (0.018)	-0.017 (0.027)	0.027 (0.03)	0.03 (0.077)	0.046 (0.051)	PP
rs7255	2	20878820	1,00	C	T	0.016 (0.002)	0.031 (0.018)	0.062 (0.027)	-0.033 (0.03)	0.167 (0.078)	0.051 (0.052)	PP
rs1275984	2	26911509	0,99	A	C	0.015 (0.002)	-0.041 (0.019)	-0.053 (0.027)	-0.028 (0.031)	0.043 (0.081)	-0.07 (0.052)	PP
rs11690961	2	46363336	0,99	A	C	0.025 (0.004)	0.02 (0.029)	-0.044 (0.044)	0.059 (0.048)	0.161 (0.124)	0.064 (0.082)	PP
rs11899888	2	56102744	0,97	A	G	0.027 (0.003)	-0.016 (0.026)	0.009 (0.041)	-0.032 (0.042)	-0.116 (0.106)	0.001 (0.072)	PP
rs34552760	2	56189503	0,99	A	G	0.018 (0.002)	-0.002 (0.018)	-0.033 (0.027)	0.023 (0.03)	0.016 (0.08)	0.031 (0.053)	PP
rs2723064	2	65279805	1,00	T	C	0.018 (0.002)	-0.017 (0.018)	0.012 (0.027)	-0.008 (0.031)	-0.078 (0.08)	-0.126 (0.053)	PP
rs11689667	2	85491365	1,00	T	C	0.018 (0.002)	-0.002 (0.018)	0.007 (0.027)	-0.01 (0.03)	0.005 (0.078)	-0.015 (0.051)	PP
rs59076415	2	96393944	0,91	A	G	0.016 (0.002)	-0.035 (0.02)	-0.056 (0.031)	-0.033 (0.032)	-0.073 (0.083)	0.045 (0.056)	PP
rs7058	2	96917588	0,99	T	G	0.018 (0.002)	0.005 (0.018)	0 (0.027)	-0.004 (0.03)	-0.051 (0.076)	0.078 (0.052)	PP
rs12620124	2	164940271	0,98	C	T	0.034 (0.003)	-0.009 (0.024)	-0.051 (0.037)	0.026 (0.04)	0.036 (0.098)	0.008 (0.069)	PP
rs6726740	2	165050128	0,99	T	G	0.023 (0.003)	-0.003 (0.02)	-0.018 (0.031)	0.017 (0.033)	0.133 (0.086)	-0.068 (0.058)	PP
rs560887	2	169763148	1,00	C	T	0.02 (0.003)	-0.003 (0.02)	0.019 (0.029)	0.007 (0.032)	-0.058 (0.086)	-0.091 (0.056)	PP
rs72914576	2	175529967	0,96	G	C	0.017 (0.003)	0 (0.023)	0.002 (0.034)	-0.036 (0.038)	0.177 (0.099)	0.025 (0.067)	PP
rs34720456	2	177027292	1,00	A	G	0.016 (0.002)	-0.017 (0.02)	-0.026 (0.029)	-0.01 (0.032)	-0.002 (0.083)	-0.01 (0.055)	PP
rs17362588	2	179721046	0,99	G	A	0.027 (0.004)	0.024 (0.032)	0.095 (0.046)	0.005 (0.052)	-0.084 (0.139)	-0.155 (0.092)	PP
rs10497529	2	179839888	0,87	G	A	0.04 (0.006)	0.006 (0.058)	0.039 (0.09)	-0.025 (0.095)	0.007 (0.23)	-0.013 (0.156)	PP
rs4673240	2	203783009	0,93	C	T	0.022 (0.003)	-0.013 (0.027)	-0.021 (0.04)	-0.057 (0.044)	0.087 (0.112)	0.091 (0.073)	PP
rs55881728	2	204306356	0,96	A	G	0.014 (0.002)	0.015 (0.018)	-0.019 (0.028)	0.035 (0.03)	-0.023 (0.08)	0.096 (0.053)	PP
rs1250258	2	216300185	0,97	C	T	0.026 (0.003)	0.005 (0.021)	-0.036 (0.031)	0.07 (0.034)	0.009 (0.092)	-0.043 (0.06)	PP
rs4674114	2	217659266	0,98	G	A	0.018 (0.003)	-0.028 (0.023)	-0.053 (0.035)	-0.037 (0.037)	0.046 (0.097)	0.054 (0.064)	PP
rs13032404	2	238233483	0,99	G	A	0.015 (0.002)	0.013 (0.02)	0.059 (0.029)	-0.032 (0.032)	0.072 (0.084)	-0.043 (0.055)	PP
rs9837162	3	11548202	0,97	T	C	0.014 (0.002)	-0.035 (0.019)	-0.072 (0.028)	-0.001 (0.031)	-0.021 (0.079)	-0.01 (0.053)	PP
rs2643826	3	27562988	0,96	T	C	0.017 (0.002)	0.012 (0.018)	0.034 (0.028)	0.006 (0.03)	-0.139 (0.08)	0.019 (0.052)	PP
rs28576724	3	36959899	1,00	T	C	0.014 (0.002)	-0.028 (0.018)	-0.018 (0.027)	-0.03 (0.03)	-0.107 (0.078)	-0.027 (0.052)	PP
rs6801957	3	38767315	1,00	T	C	0.014 (0.002)	-0.001 (0.018)	0.023 (0.027)	-0.041 (0.03)	-0.149 (0.077)	0.104 (0.052)	PP
rs6768542	3	41865474	0,93	G	A	0.048 (0.003)	-0.053 (0.026)	-0.021 (0.039)	-0.113 (0.042)	-0.037 (0.104)	0.008 (0.072)	PP
rs352139	3	52258372	0,99	C	T	0.015 (0.002)	0.01 (0.018)	0.014 (0.027)	0.029 (0.03)	-0.041 (0.078)	-0.04 (0.052)	PP
rs2710323	3	52815905	1,00	C	T	0.017 (0.002)	0.012 (0.018)	0.012 (0.027)	0.009 (0.03)	-0.034 (0.077)	0.04 (0.052)	PP
rs2241823	3	63965093	0,99	C	A	0.015 (0.003)	-0.014 (0.02)	-0.031 (0.029)	0.016 (0.032)	0.041 (0.084)	-0.065 (0.056)	PP
rs9840088	3	66442545	0,99	A	C	0.02 (0.003)	0.001 (0.023)	0.026 (0.034)	-0.019 (0.038)	-0.005 (0.101)	-0.028 (0.064)	PP
rs1599116	3	115077351	0,99	G	T	0.019 (0.003)	-0.014 (0.026)	-0.04 (0.038)	-0.023 (0.042)	0.188 (0.109)	0.022 (0.074)	PP
rs62270945	3	128201889	0,72	T	C	0.048 (0.007)	0.031 (0.059)	NA (NA)	0.003 (0.094)	-0.204 (0.221)	0.132 (0.15)	PP
rs56394279	3	160171092	0,99	C	T	0.014 (0.002)	0.006 (0.018)	-0.003 (0.027)	0.022 (0.03)	-0.003 (0.078)	-0.009 (0.051)	PP
rs1290933	4	2668217	0,98	C	A	0.017 (0.002)	0.026 (0.02)	0.018 (0.03)	0.004 (0.032)	0.043 (0.085)	0.113 (0.057)	PP
rs2498323	4	3451109	0,98	A	G	0.026 (0.004)	-0.012 (0.032)	-0.029 (0.051)	-0.022 (0.05)	0.173 (0.131)	-0.017 (0.084)	PP
rs6838613	4	48476539	1,00	A	G	0.014 (0.002)	0.011 (0.018)	0.042 (0.027)	-0.008 (0.03)	-0.151 (0.077)	0.028 (0.051)	PP
rs871606	4	54799245	0,97	T	C	0.043 (0.004)	-0.001 (0.03)	0.018 (0.044)	-0.016 (0.048)	-0.069 (0.124)	0.005 (0.088)	PP
rs1800809	4	55093914	1,00	G	A	0.017 (0.003)	0.001 (0.022)	-0.033 (0.034)	0.007 (0.037)	0.04 (0.095)	0.085 (0.062)	PP
rs11099097	4	81167309	0,98	T	C	0.023 (0.003)	-0.034 (0.02)	-0.038 (0.029)	-0.033 (0.033)	-0.01 (0.087)	-0.035 (0.057)	PP
rs57129466	4	86740933	0,96	T	C	0.027 (0.004)	0.023 (0.031)	0 (0.046)	0.058 (0.051)	0.087 (0.133)	-0.026 (0.085)	PP

rs1229984	4	100239319	0,79	C	T	0.056 (0.008)	0.025 (0.047)	-0.001 (0.076)	0.072 (0.072)	0.076 (0.197)	-0.095 (0.14)	PP
rs7662069	4	146809016	0,97	T	G	0.016 (0.002)	0.035 (0.018)	0.029 (0.027)	0.026 (0.03)	-0.052 (0.078)	0.124 (0.052)	PP
rs6855875	4	148402737	0,98	T	C	0.02 (0.003)	0 (0.024)	0.007 (0.036)	0.03 (0.039)	-0.042 (0.1)	-0.091 (0.066)	PP
rs17033050	4	156401693	1,00	G	A	0.027 (0.003)	-0.003 (0.024)	0.023 (0.036)	-0.044 (0.041)	0.15 (0.108)	-0.048 (0.069)	PP
rs13129779	4	169726027	0,99	C	T	0.02 (0.002)	-0.03 (0.018)	-0.019 (0.027)	-0.031 (0.03)	-0.025 (0.078)	-0.073 (0.051)	PP
rs3828589	5	32713327	0,99	T	C	0.024 (0.002)	-0.006 (0.019)	-0.011 (0.028)	0.017 (0.03)	-0.094 (0.078)	-0.018 (0.053)	PP
rs7733331	5	32828846	0,99	C	T	0.024 (0.002)	0.034 (0.018)	0.027 (0.027)	0.072 (0.031)	-0.021 (0.081)	-0.029 (0.052)	PP
rs7709552	5	71674855	0,98	G	A	0.015 (0.002)	-0.029 (0.018)	-0.01 (0.027)	-0.06 (0.03)	-0.109 (0.079)	0.026 (0.051)	PP
rs10474561	5	77898383	0,99	A	G	0.015 (0.002)	-0.018 (0.018)	-0.028 (0.027)	-0.032 (0.03)	0.075 (0.077)	0.015 (0.052)	PP
rs77844865	5	108074229	0,99	T	C	0.028 (0.004)	0.028 (0.032)	0.036 (0.047)	0.01 (0.054)	0.145 (0.14)	0.005 (0.09)	PP
rs1644318	5	122471989	1,00	C	T	0.018 (0.002)	-0.019 (0.018)	-0.026 (0.027)	-0.033 (0.03)	0.025 (0.079)	0.028 (0.053)	PP
rs2303719	5	122682154	1,00	T	G	0.019 (0.003)	-0.015 (0.02)	-0.008 (0.03)	0 (0.033)	-0.002 (0.084)	-0.091 (0.057)	PP
rs10077690	5	147788798	1,00	A	G	0.016 (0.003)	0.004 (0.022)	0.021 (0.032)	-0.026 (0.036)	0.019 (0.09)	0.02 (0.061)	PP
rs4705298	5	148335243	0,98	C	T	0.015 (0.002)	0.038 (0.02)	0.018 (0.029)	0.065 (0.032)	0.04 (0.081)	0.029 (0.056)	PP
rs13436194	5	157803588	0,98	A	G	0.014 (0.002)	-0.007 (0.018)	0.007 (0.027)	-0.011 (0.03)	0.06 (0.079)	-0.082 (0.054)	PP
rs11135046	5	158230013	1,00	T	G	0.019 (0.002)	0.006 (0.018)	0.014 (0.027)	0.01 (0.03)	-0.022 (0.077)	-0.024 (0.051)	PP
rs11964049	6	7709052	0,99	T	G	0.013 (0.002)	0.009 (0.018)	0.018 (0.027)	-0.015 (0.03)	-0.024 (0.077)	0.063 (0.051)	PP
rs9349379	6	12903957	0,95	A	G	0.023 (0.002)	-0.03 (0.019)	-0.04 (0.029)	-0.014 (0.03)	-0.048 (0.078)	-0.032 (0.053)	PP
rs4712936	6	25417423	0,99	G	T	0.025 (0.004)	-0.108 (0.038)	-0.042 (0.061)	-0.103 (0.062)	-0.134 (0.156)	-0.254 (0.091)	PP
rs7748167	6	25904652	1,00	C	A	0.027 (0.003)	-0.076 (0.03)	-0.076 (0.048)	-0.03 (0.049)	-0.174 (0.128)	-0.154 (0.078)	PP
rs6918586	6	26097384	1,00	C	T	0.019 (0.002)	-0.034 (0.018)	-0.031 (0.027)	0.009 (0.03)	-0.075 (0.079)	-0.158 (0.053)	PP
rs34477427	6	26589359	0,72	A	G	0.021 (0.003)	-0.009 (0.034)	NA (NA)	0.043 (0.052)	0.01 (0.134)	-0.038 (0.085)	PP
rs34864993	6	26970895	0,99	A	C	0.024 (0.003)	-0.094 (0.029)	-0.022 (0.045)	-0.1 (0.046)	-0.143 (0.119)	-0.252 (0.074)	PP
rs6904596	6	27491299	1,00	A	G	0.027 (0.003)	-0.081 (0.031)	-0.026 (0.049)	-0.086 (0.05)	-0.033 (0.13)	-0.225 (0.078)	PP
rs149990	6	27998258	1,00	A	G	0.023 (0.003)	-0.06 (0.028)	-0.003 (0.044)	-0.039 (0.046)	-0.012 (0.12)	-0.295 (0.074)	PP
rs144447022	6	29244219	1,00	T	G	0.025 (0.004)	-0.09 (0.032)	0.002 (0.051)	-0.12 (0.052)	-0.06 (0.14)	-0.258 (0.08)	PP
rs114006078	6	31211473	0,93	G	A	0.028 (0.005)	0.012 (0.042)	-0.118 (0.067)	0.117 (0.065)	0.456 (0.174)	-0.116 (0.109)	PP
rs9266233	6	31325430	0,90	G	A	0.014 (0.002)	-0.038 (0.019)	-0.051 (0.03)	-0.016 (0.03)	0.003 (0.078)	-0.083 (0.052)	PP
rs3749953	6	31713124	1,00	A	G	0.036 (0.004)	0.026 (0.027)	-0.008 (0.041)	0.08 (0.043)	0.156 (0.115)	-0.087 (0.078)	PP
rs115463529	6	32143478	0,93	C	T	0.053 (0.008)	-0.005 (0.058)	-0.032 (0.089)	-0.011 (0.092)	0.248 (0.26)	0.006 (0.171)	PP
rs9272450	6	32605478	0,77	T	C	0.021 (0.003)	-0.023 (0.024)	NA (NA)	-0.071 (0.035)	0.215 (0.099)	-0.1 (0.059)	PP
rs10456726	6	56057665	0,98	A	G	0.035 (0.003)	-0.025 (0.026)	0.018 (0.038)	-0.053 (0.042)	-0.101 (0.11)	-0.065 (0.075)	PP
rs9382873	6	57627150	0,79	A	C	0.015 (0.002)	-0.019 (0.02)	NA (NA)	-0.015 (0.032)	-0.005 (0.084)	-0.033 (0.055)	PP
rs9370723	6	58075040	0,80	T	C	0.015 (0.002)	-0.016 (0.021)	-0.005 (0.032)	-0.021 (0.033)	0.084 (0.086)	-0.083 (0.057)	PP
rs1608351	6	61942664	0,93	C	A	0.015 (0.002)	-0.038 (0.02)	-0.039 (0.032)	-0.02 (0.031)	0.01 (0.081)	-0.102 (0.052)	PP
rs580359	6	62573369	0,99	A	G	0.013 (0.002)	-0.022 (0.018)	-0.016 (0.028)	-0.013 (0.03)	0.018 (0.079)	-0.086 (0.051)	PP
rs10943073	6	73658988	1,00	T	G	0.016 (0.003)	-0.015 (0.02)	-0.014 (0.03)	-0.025 (0.034)	0.036 (0.089)	-0.012 (0.058)	PP
rs9486719	6	97060124	0,96	A	G	0.021 (0.003)	-0.064 (0.023)	-0.062 (0.036)	-0.069 (0.038)	-0.055 (0.094)	-0.055 (0.065)	PP
rs10457318	6	117937494	0,99	T	C	0.015 (0.002)	-0.015 (0.018)	-0.031 (0.027)	0.012 (0.03)	-0.01 (0.076)	-0.042 (0.052)	PP
rs9482167	6	121780762	0,94	T	C	0.015 (0.002)	0.001 (0.019)	0.019 (0.029)	-0.007 (0.031)	0.041 (0.078)	-0.054 (0.052)	PP
rs78470074	6	121962553	0,92	G	A	0.05 (0.007)	-0.085 (0.051)	-0.108 (0.074)	-0.08 (0.083)	0.188 (0.237)	-0.117 (0.154)	PP
rs11752784	6	122193876	0,93	A	G	0.039 (0.004)	-0.06 (0.031)	-0.058 (0.045)	-0.063 (0.051)	0.01 (0.142)	-0.088 (0.089)	PP
rs9388518	6	127103785	0,98	A	C	0.016 (0.002)	0.003 (0.018)	-0.006 (0.027)	0.018 (0.03)	0.026 (0.078)	-0.018 (0.052)	PP
rs1570350	6	143592386	1,00	A	G	0.016 (0.002)	0.012 (0.018)	-0.003 (0.027)	0 (0.03)	0.023 (0.079)	0.095 (0.051)	PP
rs9340985	6	152341587	1,00	C	T	0.044 (0.004)	-0.024 (0.028)	-0.054 (0.041)	-0.009 (0.046)	-0.011 (0.127)	0.045 (0.082)	PP
rs62428928	6	155567861	1,00	A	C	0.016 (0.002)	0.016 (0.018)	-0.002 (0.027)	0.014 (0.03)	0.058 (0.081)	0.073 (0.053)	PP
rs434578	6	159693220	0,97	C	T	0.032 (0.003)	-0.034 (0.027)	-0.031 (0.041)	0.004 (0.045)	-0.24 (0.115)	-0.065 (0.075)	PP
rs1322640	6	169586887	0,97	C	T	0.024 (0.003)	-0.007 (0.022)	0.064 (0.033)	-0.027 (0.036)	-0.078 (0.096)	-0.176 (0.062)	PP
rs3253	6	169616112	1,00	C	T	0.018 (0.002)	-0.01 (0.019)	-0.022 (0.028)	-0.011 (0.032)	0.017 (0.084)	0.026 (0.055)	PP

rs9505895	6	169622490	0,98	G	A	0.027 (0.003)	0.004 (0.023)	0.05 (0.033)	-0.041 (0.038)	-0.042 (0.099)	-0.02 (0.066)	PP
rs2107595	7	19049388	0,99	A	G	0.036 (0.003)	0.006 (0.024)	0.051 (0.035)	-0.032 (0.04)	-0.19 (0.108)	0.032 (0.072)	PP
rs6461992	7	27220831	0,97	G	A	0.032 (0.004)	0.057 (0.034)	0.004 (0.049)	0.129 (0.056)	0.195 (0.15)	-0.006 (0.099)	PP
rs4723954	7	40417925	0,99	G	A	0.024 (0.004)	0.008 (0.029)	0.013 (0.041)	-0.013 (0.048)	0.081 (0.135)	0.021 (0.083)	PP
rs1496499	7	45979023	1,00	T	G	0.032 (0.002)	-0.02 (0.018)	0.007 (0.027)	-0.021 (0.03)	-0.016 (0.076)	-0.115 (0.051)	PP
rs11977526	7	46008110	0,96	G	A	0.033 (0.002)	-0.004 (0.019)	0.016 (0.028)	0.017 (0.031)	-0.036 (0.08)	-0.112 (0.052)	PP
rs11770630	7	89805241	0,99	T	C	0.014 (0.002)	-0.021 (0.018)	-0.043 (0.027)	0.013 (0.03)	-0.076 (0.077)	-0.021 (0.052)	PP
rs73222720	7	90464141	0,99	C	T	0.018 (0.003)	-0.016 (0.023)	-0.046 (0.035)	0.032 (0.038)	-0.197 (0.099)	0.037 (0.065)	PP
rs12704628	7	91478070	0,98	A	G	0.014 (0.002)	-0.016 (0.018)	-0.039 (0.027)	0.012 (0.03)	0.108 (0.078)	-0.07 (0.053)	PP
rs42377	7	92243672	0,97	G	A	0.028 (0.003)	-0.046 (0.02)	-0.019 (0.029)	-0.091 (0.032)	-0.051 (0.084)	-0.009 (0.056)	PP
rs12705090	7	100467700	0,99	C	T	0.023 (0.003)	-0.03 (0.023)	-0.046 (0.034)	-0.039 (0.038)	-0.016 (0.104)	0.055 (0.067)	PP
rs2392929	7	106414069	0,96	G	T	0.069 (0.003)	-0.02 (0.023)	-0.028 (0.034)	0.004 (0.037)	-0.123 (0.097)	-0.02 (0.066)	PP
rs35680304	7	130973495	0,96	T	C	0.014 (0.002)	0.041 (0.019)	0.049 (0.028)	0.05 (0.03)	0.023 (0.082)	-0.003 (0.053)	PP
rs73727605	7	149474622	0,97	A	G	0.03 (0.005)	-0.033 (0.039)	-0.058 (0.06)	0.005 (0.062)	-0.095 (0.157)	-0.037 (0.104)	PP
rs1115866	8	10638333	0,98	C	T	0.014 (0.002)	0.01 (0.018)	0.032 (0.027)	-0.018 (0.03)	-0.026 (0.08)	0.032 (0.052)	PP
rs73563812	8	25900405	0,99	G	T	0.017 (0.003)	0.003 (0.021)	0.014 (0.031)	-0.039 (0.035)	0.04 (0.09)	0.069 (0.06)	PP
rs2978456	8	42324765	0,96	C	T	0.015 (0.002)	0.017 (0.018)	0.029 (0.028)	0.01 (0.03)	-0.044 (0.08)	0.022 (0.051)	PP
rs4873492	8	51947549	0,98	T	C	0.018 (0.003)	-0.019 (0.024)	-0.025 (0.035)	-0.039 (0.039)	0.021 (0.103)	0.046 (0.069)	PP
rs1449543	8	76591987	0,99	C	T	0.014 (0.002)	0.022 (0.018)	0.02 (0.027)	0.008 (0.03)	0.088 (0.078)	0.041 (0.052)	PP
rs11985148	8	77643348	1,00	A	G	0.021 (0.003)	-0.028 (0.024)	0.014 (0.036)	-0.032 (0.04)	-0.227 (0.105)	-0.091 (0.071)	PP
rs7009170	8	92149429	1,00	C	T	0.015 (0.002)	0.003 (0.019)	0.016 (0.029)	0.015 (0.032)	0.008 (0.084)	-0.081 (0.054)	PP
rs79253921	8	120458928	0,99	C	T	0.039 (0.003)	0.018 (0.022)	0.037 (0.034)	0.027 (0.036)	-0.23 (0.095)	0.036 (0.061)	PP
rs80309268	8	120466959	0,98	C	T	0.04 (0.003)	0.018 (0.022)	0.037 (0.034)	0.026 (0.037)	-0.241 (0.097)	0.04 (0.061)	PP
rs11997254	8	141083381	0,98	T	G	0.02 (0.002)	0.041 (0.018)	0.022 (0.027)	0.059 (0.03)	0.164 (0.079)	0.005 (0.052)	PP
rs7042232	9	14533251	0,97	G	A	0.015 (0.002)	-0.037 (0.019)	-0.054 (0.029)	-0.048 (0.032)	0.149 (0.083)	-0.026 (0.053)	PP
rs1333045	9	22119195	0,95	C	T	0.015 (0.002)	-0.032 (0.019)	-0.044 (0.028)	0.015 (0.031)	-0.123 (0.08)	-0.083 (0.053)	PP
rs989393	9	101743336	0,99	T	C	0.016 (0.003)	-0.018 (0.02)	-0.016 (0.03)	-0.006 (0.033)	-0.138 (0.085)	-0.003 (0.057)	PP
rs700071	9	125845307	0,99	G	A	0.021 (0.004)	0.004 (0.028)	-0.001 (0.042)	0.062 (0.046)	-0.21 (0.118)	-0.049 (0.078)	PP
rs72765275	9	127856566	1,00	G	A	0.027 (0.003)	-0.009 (0.027)	0.001 (0.04)	-0.048 (0.044)	-0.077 (0.115)	0.105 (0.078)	PP
rs550057	9	136146597	1,00	T	C	0.015 (0.003)	-0.026 (0.02)	-0.049 (0.03)	-0.01 (0.033)	-0.022 (0.085)	0.014 (0.058)	PP
rs7100710	10	12250861	0,99	A	G	0.017 (0.003)	-0.016 (0.022)	-0.02 (0.033)	-0.027 (0.037)	0.034 (0.095)	0.012 (0.062)	PP
rs518549	10	18226000	0,99	C	T	0.014 (0.002)	0.023 (0.019)	0.014 (0.028)	0.056 (0.031)	-0.18 (0.079)	0.045 (0.053)	PP
rs11591541	10	18421314	0,99	G	A	0.018 (0.003)	0.038 (0.024)	0.056 (0.036)	0.043 (0.04)	-0.178 (0.106)	0.048 (0.07)	PP
rs11014012	10	18681659	0,97	G	T	0.015 (0.002)	-0.04 (0.018)	-0.036 (0.027)	-0.048 (0.03)	0.007 (0.078)	-0.047 (0.052)	PP
rs7070847	10	18726054	0,94	G	A	0.018 (0.003)	-0.056 (0.02)	-0.065 (0.03)	-0.041 (0.034)	-0.12 (0.089)	-0.041 (0.058)	PP
rs10732433	10	21037294	0,99	T	C	0.015 (0.002)	-0.04 (0.018)	-0.036 (0.027)	-0.08 (0.03)	-0.045 (0.078)	0.067 (0.052)	PP
rs9337951	10	30317073	0,86	A	G	0.022 (0.002)	0.037 (0.02)	0.053 (0.03)	0.031 (0.034)	0.055 (0.089)	-0.012 (0.059)	PP
rs7920682	10	30317826	0,99	A	G	0.019 (0.002)	0.005 (0.018)	0.009 (0.027)	0.006 (0.03)	0.071 (0.079)	-0.038 (0.051)	PP
rs11813059	10	31369709	0,99	T	G	0.018 (0.003)	0.047 (0.02)	-0.005 (0.03)	0.126 (0.033)	0.112 (0.086)	-0.026 (0.057)	PP
rs72786794	10	32105718	0,99	C	T	0.015 (0.003)	-0.037 (0.02)	-0.031 (0.03)	-0.052 (0.033)	-0.054 (0.084)	-0.008 (0.055)	PP
rs10763559	10	60347410	1,00	T	C	0.017 (0.002)	-0.008 (0.018)	-0.011 (0.027)	-0.021 (0.029)	0.164 (0.08)	-0.028 (0.052)	PP
rs10821949	10	63811678	0,98	C	T	0.014 (0.002)	-0.06 (0.018)	-0.076 (0.027)	-0.042 (0.03)	0.016 (0.079)	-0.086 (0.052)	PP
rs78249997	10	75422314	0,98	T	C	0.021 (0.003)	0.014 (0.026)	0.029 (0.038)	-0.042 (0.042)	0.003 (0.112)	0.137 (0.074)	PP
rs10824065	10	75822074	0,96	C	T	0.018 (0.002)	-0.027 (0.018)	-0.053 (0.028)	-0.012 (0.03)	0.002 (0.079)	0.008 (0.052)	PP
rs1538311	10	76415509	1,00	G	T	0.015 (0.002)	-0.009 (0.018)	0.002 (0.027)	-0.005 (0.03)	-0.027 (0.078)	-0.056 (0.052)	PP
rs11187793	10	95896716	0,95	A	G	0.016 (0.002)	-0.006 (0.019)	-0.012 (0.028)	0.009 (0.031)	0.032 (0.08)	-0.047 (0.053)	PP
rs11187844	10	96056629	0,96	C	A	0.028 (0.004)	0.017 (0.028)	0.01 (0.041)	0.043 (0.046)	-0.07 (0.12)	0.006 (0.081)	PP
rs11190709	10	102552663	1,00	A	G	0.022 (0.004)	-0.056 (0.029)	-0.031 (0.043)	-0.049 (0.047)	-0.064 (0.122)	-0.156 (0.079)	PP
rs45516493	10	104378363	0,88	A	G	0.02 (0.003)	-0.023 (0.029)	-0.022 (0.045)	-0.03 (0.046)	-0.216 (0.129)	0.072 (0.082)	PP

rs112913898	10	104958900	0,98	G	A	0.037 (0.004)	0.003 (0.032)	0.013 (0.047)	-0.004 (0.052)	-0.274 (0.135)	0.123 (0.096)	PP
rs7089271	10	105176914	0,98	A	G	0.014 (0.002)	0.017 (0.018)	0.032 (0.027)	-0.002 (0.03)	-0.115 (0.078)	0.077 (0.051)	PP
rs4630220	10	105459116	0,92	A	G	0.015 (0.003)	0.004 (0.021)	0.051 (0.032)	-0.066 (0.034)	0.002 (0.09)	0.056 (0.059)	PP
rs10885409	10	114808072	1,00	C	T	0.014 (0.002)	-0.034 (0.018)	-0.02 (0.027)	-0.059 (0.03)	0.032 (0.077)	-0.042 (0.052)	PP
rs72830615	10	122988575	0,99	A	G	0.014 (0.002)	-0.004 (0.019)	-0.007 (0.028)	-0.001 (0.03)	0.094 (0.079)	-0.044 (0.052)	PP
rs612652	11	1887216	0,95	C	T	0.022 (0.002)	0.013 (0.019)	-0.002 (0.029)	0.037 (0.031)	0.045 (0.079)	-0.018 (0.052)	PP
rs686722	11	1891722	0,97	T	C	0.022 (0.002)	0.004 (0.019)	0.007 (0.029)	-0.007 (0.031)	0.093 (0.077)	-0.016 (0.054)	PP
rs12577815	11	17266868	1,00	A	G	0.016 (0.002)	-0.005 (0.02)	0.04 (0.029)	-0.039 (0.032)	-0.056 (0.084)	-0.041 (0.055)	PP
rs11605215	11	30443248	0,99	A	G	0.019 (0.003)	-0.013 (0.022)	-0.003 (0.033)	-0.005 (0.036)	0.007 (0.094)	-0.082 (0.063)	PP
rs3740781	11	45255926	0,97	T	C	0.021 (0.003)	-0.018 (0.02)	-0.006 (0.029)	-0.044 (0.034)	-0.089 (0.086)	0.048 (0.058)	PP
rs7107356	11	47676170	0,99	G	A	0.014 (0.002)	0.026 (0.018)	0.024 (0.027)	0.058 (0.03)	-0.141 (0.076)	0.012 (0.051)	PP
rs7117878	11	57421457	0,98	C	A	0.014 (0.002)	-0.002 (0.019)	0.017 (0.029)	-0.008 (0.032)	-0.08 (0.084)	-0.022 (0.055)	PP
rs75694075	11	76494760	0,98	C	T	0.018 (0.003)	-0.01 (0.024)	0.007 (0.035)	-0.026 (0.04)	-0.002 (0.101)	-0.034 (0.067)	PP
rs10830279	11	89224326	0,95	A	G	0.025 (0.003)	0.01 (0.02)	0.03 (0.029)	-0.016 (0.033)	-0.017 (0.087)	0.023 (0.057)	PP
rs2289125	11	89224453	0,92	C	A	0.033 (0.003)	0.005 (0.023)	0.021 (0.034)	-0.005 (0.037)	-0.06 (0.1)	-0.001 (0.064)	PP
rs604723	11	100610546	1,00	C	T	0.018 (0.003)	0.006 (0.02)	-0.03 (0.029)	0.059 (0.033)	-0.048 (0.088)	0.007 (0.058)	PP
rs4754196	11	107096777	0,96	G	A	0.017 (0.002)	-0.004 (0.018)	0.019 (0.027)	-0.049 (0.03)	0.064 (0.076)	0.018 (0.053)	PP
rs1245114	11	112950782	0,99	T	C	0.014 (0.002)	0.054 (0.018)	0.092 (0.027)	0.02 (0.03)	-0.031 (0.079)	0.054 (0.052)	PP
rs573455	11	117267884	0,98	A	G	0.021 (0.002)	0.008 (0.018)	-0.002 (0.027)	0.019 (0.03)	-0.048 (0.078)	0.038 (0.052)	PP
rs4936099	11	130280725	0,96	C	A	0.04 (0.002)	-0.002 (0.019)	0.006 (0.028)	0.01 (0.031)	-0.048 (0.08)	-0.047 (0.053)	PP
rs2291347	11	130286766	1,00	G	A	0.029 (0.002)	-0.004 (0.018)	0.003 (0.026)	-0.004 (0.03)	-0.002 (0.076)	-0.033 (0.051)	PP
rs7939270	11	130449163	0,99	T	C	0.019 (0.002)	-0.018 (0.019)	0.006 (0.028)	-0.043 (0.031)	-0.096 (0.08)	0 (0.054)	PP
rs12369319	12	12622025	0,96	A	G	0.014 (0.002)	-0.014 (0.019)	-0.043 (0.029)	0.007 (0.032)	0.1 (0.079)	-0.03 (0.054)	PP
rs10845601	12	12820134	0,99	G	A	0.014 (0.002)	0.013 (0.019)	0.018 (0.028)	0.029 (0.031)	-0.06 (0.077)	-0.02 (0.053)	PP
rs10770612	12	20230639	0,90	A	G	0.031 (0.003)	-0.023 (0.023)	-0.02 (0.034)	-0.077 (0.039)	0.085 (0.097)	0.085 (0.069)	PP
rs11047527	12	24762501	0,99	C	A	0.021 (0.003)	0.035 (0.025)	0.071 (0.037)	0.017 (0.042)	0.096 (0.108)	-0.074 (0.073)	PP
rs1050288	12	27955296	0,88	T	C	0.016 (0.002)	-0.025 (0.02)	-0.027 (0.03)	-0.033 (0.034)	-0.017 (0.091)	0.006 (0.06)	PP
rs61915449	12	27957674	0,85	C	T	0.02 (0.003)	-0.021 (0.029)	-0.021 (0.041)	0.015 (0.049)	0.025 (0.126)	-0.15 (0.084)	PP
rs4587807	12	33510115	1,00	A	G	0.014 (0.002)	0.026 (0.018)	0.008 (0.027)	0.041 (0.03)	0.07 (0.077)	0.031 (0.051)	PP
rs67772913	12	54435716	0,98	A	G	0.015 (0.003)	0.01 (0.02)	0.017 (0.03)	0.009 (0.033)	0.014 (0.086)	-0.013 (0.056)	PP
rs2681492	12	90013089	1,00	T	C	0.029 (0.003)	-0.008 (0.025)	-0.014 (0.037)	-0.013 (0.041)	-0.121 (0.104)	0.08 (0.068)	PP
rs111337717	12	90037506	0,81	T	C	0.036 (0.005)	-0.03 (0.043)	-0.037 (0.065)	-0.018 (0.07)	-0.029 (0.178)	-0.038 (0.117)	PP
rs78941936	12	94732711	1,00	C	T	0.029 (0.004)	-0.019 (0.035)	0.018 (0.055)	-0.085 (0.058)	0.104 (0.138)	-0.006 (0.091)	PP
rs7954260	12	95518223	1,00	T	C	0.019 (0.003)	-0.01 (0.023)	-0.035 (0.035)	0.028 (0.038)	-0.204 (0.103)	0.043 (0.066)	PP
rs12875271	13	110792743	0,92	G	A	0.024 (0.004)	0.052 (0.033)	0.15 (0.052)	-0.044 (0.051)	0.159 (0.134)	0.009 (0.089)	PP
rs28730765	14	23855511	0,77	T	C	0.061 (0.01)	-0.087 (0.094)	NA (NA)	-0.104 (0.131)	-0.491 (0.366)	0.352 (0.255)	PP
rs365990	14	23861811	1,00	A	G	0.028 (0.002)	-0.039 (0.019)	-0.053 (0.028)	-0.04 (0.031)	0.189 (0.081)	-0.084 (0.052)	PP
rs403739	14	23868776	0,98	T	C	0.022 (0.002)	-0.026 (0.019)	-0.064 (0.028)	-0.002 (0.032)	0.197 (0.081)	-0.061 (0.054)	PP
rs9888615	14	53377540	0,97	C	T	0.015 (0.003)	0.012 (0.02)	0.01 (0.03)	0.001 (0.033)	-0.012 (0.085)	0.064 (0.058)	PP
rs4903064	14	73279420	0,96	C	T	0.019 (0.003)	0.164 (0.021)	0.205 (0.032)	0.128 (0.035)	0.039 (0.088)	0.188 (0.061)	PP
rs59084020	14	73302086	0,99	G	T	0.016 (0.002)	0.055 (0.018)	0.046 (0.027)	0.102 (0.03)	-0.003 (0.077)	-0.023 (0.051)	PP
rs1899445	14	85779193	0,99	T	C	0.015 (0.003)	0.016 (0.02)	0.062 (0.031)	-0.044 (0.033)	-0.012 (0.085)	0.047 (0.057)	PP
rs12431811	14	94455787	0,96	C	T	0.015 (0.002)	0.009 (0.019)	0.035 (0.028)	-0.042 (0.031)	0.078 (0.078)	0.03 (0.052)	PP
rs1381288	14	98592534	0,99	A	G	0.017 (0.002)	-0.002 (0.019)	-0.01 (0.028)	0.022 (0.031)	-0.113 (0.08)	-0.001 (0.053)	PP
rs7158073	14	100141689	0,94	T	C	0.016 (0.002)	0.012 (0.019)	0.022 (0.028)	0.001 (0.031)	-0.057 (0.077)	0.04 (0.053)	PP
rs11160546	14	100233353	1,00	T	C	0.018 (0.002)	0.016 (0.019)	0.055 (0.029)	-0.022 (0.032)	-0.082 (0.082)	0.027 (0.054)	PP
rs2015637	15	48716853	0,96	T	C	0.039 (0.004)	-0.072 (0.031)	-0.096 (0.048)	-0.021 (0.049)	0.141 (0.127)	-0.259 (0.088)	PP
rs1036477	15	48914926	1,00	A	G	0.038 (0.004)	-0.064 (0.029)	-0.063 (0.042)	-0.046 (0.047)	0.11 (0.122)	-0.211 (0.085)	PP
rs62019074	15	50726023	0,97	T	C	0.018 (0.003)	-0.008 (0.025)	0.034 (0.039)	-0.038 (0.041)	-0.089 (0.105)	-0.022 (0.069)	PP

rs956006	15	62808539	0,92	C	T	0.016 (0.002)	-0.007 (0.02)	-0.004 (0.03)	-0.012 (0.033)	0.064 (0.086)	-0.033 (0.058)	PP
rs12912010	15	67467143	0,99	T	G	0.021 (0.003)	-0.066 (0.022)	-0.026 (0.032)	-0.102 (0.036)	-0.198 (0.094)	-0.044 (0.064)	PP
rs4337253	15	71609306	1,00	C	G	0.014 (0.002)	-0.013 (0.019)	-0.04 (0.029)	0.024 (0.032)	-0.017 (0.08)	-0.022 (0.055)	PP
rs4491476	15	79157405	0,96	G	A	0.017 (0.002)	0 (0.019)	-0.011 (0.028)	0.028 (0.031)	-0.042 (0.079)	-0.026 (0.053)	PP
rs1407588	15	81052947	0,99	A	G	0.014 (0.002)	-0.02 (0.018)	-0.043 (0.027)	-0.017 (0.03)	0.08 (0.076)	0.006 (0.051)	PP
rs8027450	15	91418394	0,96	T	C	0.018 (0.002)	0.006 (0.02)	0.006 (0.028)	0.001 (0.033)	0.045 (0.085)	0.007 (0.056)	PP
rs2303083	16	24835168	0,99	G	A	0.017 (0.003)	0.026 (0.023)	0.001 (0.035)	0.04 (0.039)	0.03 (0.102)	0.074 (0.066)	PP
rs1423791	16	65274433	1,00	C	A	0.018 (0.002)	0.004 (0.018)	-0.002 (0.027)	0.021 (0.03)	-0.072 (0.079)	0.005 (0.051)	PP
rs79698266	16	69163702	1,00	C	T	0.018 (0.003)	-0.006 (0.025)	-0.003 (0.038)	0.001 (0.041)	-0.108 (0.105)	0.004 (0.07)	PP
rs62053158	16	71774099	0,86	A	G	0.043 (0.005)	-0.106 (0.044)	-0.068 (0.073)	-0.092 (0.069)	-0.121 (0.183)	-0.22 (0.11)	PP
rs62058277	16	72281950	0,94	G	T	0.044 (0.005)	-0.089 (0.046)	-0.026 (0.072)	-0.096 (0.073)	-0.006 (0.203)	-0.268 (0.118)	PP
rs62051558	16	72862128	0,89	C	T	0.044 (0.005)	-0.084 (0.047)	0.008 (0.075)	-0.114 (0.075)	0.026 (0.203)	-0.28 (0.119)	PP
rs1010630	16	75328591	0,97	T	G	0.015 (0.002)	-0.015 (0.018)	0.025 (0.027)	-0.052 (0.031)	-0.018 (0.078)	-0.051 (0.053)	PP
rs6565101	16	83044388	0,99	G	A	0.019 (0.002)	-0.008 (0.018)	-0.026 (0.027)	0.009 (0.03)	0.004 (0.079)	0.004 (0.051)	PP
rs7500448	16	83045790	0,98	A	G	0.027 (0.003)	-0.023 (0.021)	-0.042 (0.031)	-0.004 (0.034)	0.013 (0.092)	-0.026 (0.06)	PP
rs10852858	17	1357751	0,88	T	C	0.016 (0.002)	-0.005 (0.019)	0.026 (0.03)	-0.002 (0.031)	-0.13 (0.08)	-0.052 (0.052)	PP
rs4790870	17	1970201	0,99	C	A	0.017 (0.002)	-0.006 (0.018)	0 (0.027)	-0.014 (0.031)	-0.021 (0.079)	0.002 (0.052)	PP
rs930526	17	6473353	0,99	A	C	0.018 (0.002)	0.036 (0.018)	0.043 (0.027)	0.037 (0.03)	0.003 (0.079)	0.023 (0.054)	PP
rs4796392	17	7174897	0,96	T	C	0.013 (0.002)	-0.026 (0.018)	-0.017 (0.028)	-0.033 (0.03)	0.066 (0.081)	-0.074 (0.052)	PP
rs1642763	17	7557419	0,99	G	A	0.016 (0.003)	-0.037 (0.022)	-0.015 (0.032)	-0.063 (0.036)	0.08 (0.092)	-0.088 (0.06)	PP
rs35447453	17	40132591	0,91	A	G	0.022 (0.004)	-0.004 (0.031)	0.027 (0.046)	-0.028 (0.05)	0.058 (0.126)	-0.066 (0.085)	PP
rs35296742	17	40999303	0,87	G	A	0.024 (0.004)	0.02 (0.032)	-0.03 (0.052)	0.02 (0.051)	0.09 (0.131)	0.112 (0.083)	PP
rs71373532	17	43153235	0,91	T	C	0.034 (0.004)	-0.017 (0.036)	-0.019 (0.057)	0.02 (0.058)	-0.002 (0.153)	-0.12 (0.095)	PP
rs17608766	17	45013271	0,98	C	T	0.036 (0.003)	-0.017 (0.027)	0.018 (0.043)	-0.021 (0.044)	-0.142 (0.112)	-0.055 (0.072)	PP
rs62076103	17	45888374	0,92	G	A	0.031 (0.005)	0.024 (0.039)	0.028 (0.065)	0.083 (0.061)	-0.249 (0.164)	-0.038 (0.098)	PP
rs1468270	17	46125191	0,98	C	T	0.021 (0.003)	-0.012 (0.022)	-0.011 (0.032)	-0.008 (0.036)	-0.042 (0.092)	-0.02 (0.063)	PP
rs34786235	17	46884282	0,94	T	C	0.051 (0.007)	-0.034 (0.061)	0.055 (0.103)	-0.071 (0.094)	0.014 (0.255)	-0.155 (0.155)	PP
rs1000423	17	59475642	0,93	T	C	0.017 (0.003)	-0.013 (0.021)	0.005 (0.032)	-0.029 (0.034)	-0.014 (0.092)	-0.021 (0.059)	PP
rs740698	17	60767151	0,98	C	T	0.018 (0.002)	-0.029 (0.018)	-0.042 (0.028)	-0.008 (0.03)	-0.042 (0.08)	-0.04 (0.052)	PP
rs3786132	17	60768921	0,99	C	A	0.019 (0.002)	-0.035 (0.018)	-0.048 (0.027)	-0.007 (0.03)	-0.088 (0.081)	-0.051 (0.052)	PP
rs9912402	17	62767325	0,99	T	C	0.018 (0.003)	0.021 (0.024)	0.026 (0.034)	0.031 (0.039)	-0.169 (0.1)	0.059 (0.068)	PP
rs9945184	18	42004527	0,99	A	G	0.018 (0.002)	-0.028 (0.019)	-0.026 (0.028)	-0.036 (0.032)	0.156 (0.084)	-0.089 (0.054)	PP
rs7236548	18	43097750	1,00	A	C	0.03 (0.003)	0.012 (0.023)	0.034 (0.034)	0.025 (0.038)	-0.29 (0.099)	0.026 (0.066)	PP
rs10048404	18	54578482	0,91	C	T	0.015 (0.002)	-0.017 (0.02)	-0.04 (0.031)	0.011 (0.032)	0.12 (0.087)	-0.077 (0.053)	PP
rs55678414	19	2177625	0,97	G	T	0.045 (0.005)	0.012 (0.035)	0 (0.051)	0.001 (0.059)	0.197 (0.159)	0.018 (0.108)	PP
rs1982468	19	4953351	1,00	T	G	0.016 (0.002)	0.004 (0.019)	-0.023 (0.028)	-0.01 (0.031)	0.101 (0.081)	0.101 (0.053)	PP
rs7508679	19	7222832	1,00	C	T	0.015 (0.002)	-0.004 (0.018)	0.024 (0.027)	-0.041 (0.03)	-0.029 (0.078)	0.018 (0.052)	PP
rs36047283	19	7255701	0,84	A	G	0.023 (0.003)	-0.037 (0.031)	-0.027 (0.043)	-0.013 (0.053)	-0.16 (0.118)	NA (NA)	PP
rs10409243	19	10332988	1,00	C	T	0.014 (0.002)	-0.056 (0.018)	-0.064 (0.027)	-0.044 (0.03)	-0.163 (0.08)	-0.018 (0.052)	PP
rs1044006	19	15285052	0,99	C	T	0.031 (0.004)	0.019 (0.03)	0.036 (0.043)	0.04 (0.051)	-0.041 (0.128)	-0.081 (0.087)	PP
rs7412	19	45412079	0,94	C	T	0.027 (0.004)	0.015 (0.035)	0.055 (0.054)	-0.013 (0.055)	-0.042 (0.144)	0 (0.095)	PP
rs73046792	19	49605705	0,90	G	A	0.019 (0.003)	0.002 (0.027)	0.058 (0.042)	-0.058 (0.043)	0.022 (0.112)	0.003 (0.071)	PP
rs11087740	20	6657554	0,99	C	T	0.014 (0.002)	0.004 (0.018)	0.017 (0.027)	0 (0.03)	0.029 (0.077)	-0.043 (0.051)	PP
rs653807	20	10467451	1,00	A	G	0.025 (0.002)	0.009 (0.018)	0.026 (0.027)	-0.015 (0.03)	-0.041 (0.077)	0.04 (0.052)	PP
rs2206815	20	10669188	0,98	C	A	0.031 (0.002)	-0.014 (0.018)	-0.022 (0.027)	0.007 (0.03)	-0.094 (0.078)	-0.012 (0.052)	PP
rs6046140	20	19476530	1,00	A	G	0.024 (0.003)	0.031 (0.02)	0.057 (0.03)	0.009 (0.034)	0.035 (0.089)	-0.004 (0.059)	PP
rs6062543	20	62451079	0,89	T	C	0.017 (0.003)	0.05 (0.022)	-0.008 (0.034)	0.1 (0.036)	0.015 (0.095)	0.106 (0.063)	PP
rs2409327	21	30127668	1,00	A	G	0.024 (0.003)	-0.008 (0.026)	-0.046 (0.039)	-0.032 (0.043)	0.124 (0.111)	0.139 (0.073)	PP
rs1034565	22	19984211	1,00	T	C	0.017 (0.003)	-0.012 (0.02)	0.016 (0.029)	-0.053 (0.033)	-0.052 (0.085)	0.019 (0.057)	PP

rs12485003	22	40635276	0,87	A	G	0.031 (0.004)	0.01 (0.033)	-0.001 (0.048)	-0.017 (0.055)	0.247 (0.158)	0.054 (0.098)	PP
rs56253436	22	40855131	0,88	G	A	0.034 (0.005)	0.01 (0.038)	-0.02 (0.052)	0.021 (0.066)	0.176 (0.19)	0.063 (0.118)	PP
rs12748152	1	27138393	0,99	C	T	0.051 (0.006)	-0.073 (0.032)	-0.054 (0.047)	-0.171 (0.054)	0.008 (0.15)	0.108 (0.093)	HDL
rs4660293	1	40028180	1,00	A	G	0.035 (0.004)	0.017 (0.021)	0.034 (0.031)	-0.018 (0.035)	0.122 (0.091)	0.013 (0.06)	HDL
rs12145743	1	156700651	1,00	G	T	0.02 (0.004)	0.033 (0.019)	0.033 (0.028)	0.01 (0.031)	0.032 (0.083)	0.107 (0.055)	HDL
rs4650994	1	178515312	1,00	G	A	0.021 (0.003)	-0.018 (0.018)	-0.022 (0.027)	-0.044 (0.03)	0.015 (0.079)	0.053 (0.05)	HDL
rs1689800	1	182168885	0,99	A	G	0.034 (0.004)	0.035 (0.019)	0.07 (0.028)	-0.003 (0.031)	-0.013 (0.082)	0.04 (0.053)	HDL
rs4846914	1	230295691	0,97	A	G	0.048 (0.003)	0.019 (0.019)	0.033 (0.028)	0.004 (0.03)	-0.104 (0.081)	0.073 (0.054)	HDL
rs1042034	2	21225281	1,00	C	T	0.066 (0.004)	-0.007 (0.022)	0.044 (0.032)	-0.049 (0.036)	-0.106 (0.096)	-0.031 (0.064)	HDL
rs12328675	2	165540800	1,00	C	T	0.045 (0.005)	-0.015 (0.027)	0.012 (0.04)	-0.043 (0.045)	-0.179 (0.117)	0.035 (0.078)	HDL
rs1047891	2	211540507	0,90	C	A	0.027 (0.004)	-0.009 (0.02)	-0.006 (0.031)	-0.036 (0.033)	-0.029 (0.085)	0.064 (0.057)	HDL
rs2972146	2	227100698	1,00	G	T	0.032 (0.004)	-0.034 (0.019)	-0.012 (0.028)	-0.055 (0.031)	-0.103 (0.082)	-0.021 (0.053)	HDL
rs2606736	3	11400249	0,99	C	T	0.025 (0.004)	0.023 (0.019)	0.043 (0.028)	-0.002 (0.031)	0.04 (0.079)	0.016 (0.054)	HDL
rs2290547	3	47061183	0,98	G	A	0.03 (0.005)	-0.014 (0.024)	-0.002 (0.035)	0 (0.04)	0.124 (0.102)	-0.161 (0.067)	HDL
rs2013208	3	50129399	1,00	T	C	0.025 (0.004)	-0.017 (0.018)	0.003 (0.027)	-0.011 (0.03)	-0.106 (0.078)	-0.072 (0.052)	HDL
rs13326165	3	52532118	0,97	A	G	0.029 (0.004)	0.039 (0.023)	0.083 (0.034)	0.008 (0.039)	-0.072 (0.095)	0.013 (0.065)	HDL
rs6805251	3	119560606	1,00	T	C	0.02 (0.004)	-0.041 (0.018)	-0.047 (0.027)	-0.052 (0.03)	0.039 (0.079)	-0.025 (0.052)	HDL
rs17404153	3	132163200	1,00	T	G	0.01 (0.005)	0.03 (0.027)	0.044 (0.04)	0.023 (0.045)	0.074 (0.114)	-0.026 (0.078)	HDL
rs10019888	4	26062990	0,98	A	G	0.027 (0.005)	0.019 (0.024)	0.055 (0.036)	-0.02 (0.04)	-0.011 (0.104)	0.015 (0.069)	HDL
rs3822072	4	89741269	0,98	G	A	0.025 (0.003)	-0.011 (0.018)	-0.018 (0.027)	0.029 (0.03)	-0.051 (0.078)	-0.088 (0.052)	HDL
rs2602836	4	100014805	0,99	A	G	0.019 (0.003)	0.005 (0.018)	0.019 (0.027)	-0.023 (0.03)	0.077 (0.079)	0.006 (0.052)	HDL
rs13107325	4	103188709	0,97	C	T	0.071 (0.008)	0.032 (0.035)	0.048 (0.052)	0.016 (0.058)	-0.047 (0.146)	0.053 (0.097)	HDL
rs6450176	5	53298025	0,99	G	A	0.025 (0.004)	-0.008 (0.021)	0.011 (0.031)	-0.006 (0.034)	-0.086 (0.088)	-0.047 (0.058)	HDL
rs2814944	6	34552797	1,00	G	A	0.033 (0.005)	-0.052 (0.025)	0.028 (0.037)	-0.133 (0.041)	-0.145 (0.103)	-0.059 (0.072)	HDL
rs998584	6	43757896	0,84	C	A	0.026 (0.004)	-0.028 (0.02)	NA (NA)	0.002 (0.032)	-0.007 (0.083)	-0.054 (0.055)	HDL
rs1936800	6	127436064	0,98	C	T	0.02 (0.003)	0.018 (0.018)	0.027 (0.027)	0.024 (0.03)	-0.01 (0.078)	-0.018 (0.051)	HDL
rs605066	6	139829666	0,98	T	C	0.028 (0.005)	0.031 (0.018)	-0.008 (0.028)	0.062 (0.03)	0.054 (0.079)	0.072 (0.053)	HDL
rs702485	7	6449272	0,99	G	A	0.024 (0.003)	-0.008 (0.018)	0.005 (0.027)	-0.009 (0.03)	0.018 (0.077)	-0.062 (0.052)	HDL
rs4142995	7	17919258	0,99	G	T	0.026 (0.004)	-0.013 (0.018)	-0.017 (0.027)	-0.04 (0.03)	0.075 (0.077)	0.039 (0.053)	HDL
rs4917014	7	50305863	1,00	G	T	0.022 (0.004)	0.018 (0.019)	0.011 (0.029)	0.026 (0.032)	-0.038 (0.084)	0.046 (0.055)	HDL
rs17145738	7	72982874	0,96	T	C	0.041 (0.005)	-0.084 (0.029)	-0.14 (0.044)	-0.018 (0.047)	-0.168 (0.122)	-0.062 (0.079)	HDL
rs4731702	7	130433384	0,99	T	C	0.029 (0.003)	-0.008 (0.018)	0.002 (0.027)	-0.031 (0.03)	0.019 (0.077)	0.014 (0.051)	HDL
rs17173637	7	150529449	0,99	T	C	0.036 (0.006)	-0.05 (0.031)	-0.06 (0.045)	-0.014 (0.051)	-0.096 (0.135)	-0.098 (0.089)	HDL
rs9987289	8	9183358	0,99	G	A	0.082 (0.006)	0.007 (0.032)	0.022 (0.048)	-0.022 (0.052)	0.226 (0.139)	-0.05 (0.092)	HDL
rs12678919	8	19844222	1,00	G	A	0.155 (0.006)	0.056 (0.03)	0.057 (0.045)	0.05 (0.049)	0.105 (0.134)	0.046 (0.086)	HDL
rs2293889	8	116599199	0,98	G	T	0.031 (0.004)	-0.043 (0.018)	-0.034 (0.027)	-0.049 (0.03)	-0.069 (0.083)	-0.052 (0.052)	HDL
rs638491	9	15290012	0,99	G	A	0.042 (0.005)	0.05 (0.024)	0.016 (0.036)	0.089 (0.041)	-0.002 (0.104)	0.083 (0.07)	HDL
rs1883025	9	107664301	0,98	C	T	0.07 (0.004)	0.005 (0.021)	-0.006 (0.032)	0.015 (0.034)	-0.06 (0.088)	0.039 (0.058)	HDL
rs970548	10	46013277	1,00	C	A	0.026 (0.004)	-0.001 (0.021)	-0.008 (0.031)	-0.002 (0.034)	0.132 (0.091)	-0.03 (0.058)	HDL
rs2923084	11	10388782	1,00	A	G	0.026 (0.004)	0.024 (0.023)	0.021 (0.034)	0.039 (0.038)	0.086 (0.098)	-0.035 (0.067)	HDL
rs11246602	11	51512090	0,91	C	T	0.034 (0.005)	-0.056 (0.03)	NA (NA)	-0.081 (0.045)	0.156 (0.122)	-0.005 (0.075)	HDL
rs174546	11	61569830	1,00	C	T	0.039 (0.004)	0.026 (0.019)	0.009 (0.028)	0.03 (0.032)	0.093 (0.078)	0.041 (0.054)	HDL
rs12801636	11	65391317	1,00	A	G	0.024 (0.004)	0.011 (0.022)	0.003 (0.032)	0.042 (0.036)	-0.159 (0.093)	0.02 (0.061)	HDL
rs499974	11	75455021	1,00	C	A	0.026 (0.004)	-0.007 (0.024)	-0.017 (0.035)	-0.027 (0.04)	0.088 (0.106)	0.057 (0.07)	HDL
rs3741298	11	116657561	0,97	T	C	0.051 (0.006)	0.017 (0.023)	-0.003 (0.034)	0.014 (0.037)	0.094 (0.095)	0.066 (0.066)	HDL
rs7134375	12	20473758	1,00	A	C	0.021 (0.004)	-0.011 (0.018)	-0.02 (0.027)	-0.001 (0.03)	0.034 (0.077)	-0.028 (0.052)	HDL
rs11613352	12	57792580	0,99	T	C	0.028 (0.004)	0 (0.022)	-0.075 (0.033)	0.084 (0.036)	0.008 (0.091)	0.008 (0.06)	HDL
rs7134594	12	110000193	1,00	T	C	0.035 (0.005)	-0.037 (0.018)	-0.045 (0.027)	-0.032 (0.03)	0.102 (0.077)	-0.084 (0.052)	HDL
rs4759375	12	123796238	0,95	T	C	0.056 (0.01)	-0.019 (0.03)	-0.023 (0.043)	-0.01 (0.049)	-0.094 (0.132)	0.003 (0.087)	HDL

rs4765127	12	124460167	1,00	T	G	0.032 (0.005)	-0.019 (0.019)	-0.024 (0.028)	-0.014 (0.032)	0.007 (0.081)	-0.031 (0.054)	HDL
rs838880	12	125261593	0,97	C	T	0.048 (0.004)	-0.009 (0.02)	-0.013 (0.029)	0.025 (0.032)	-0.115 (0.084)	-0.053 (0.056)	HDL
rs4983559	14	105277209	0,99	G	A	0.02 (0.004)	-0.059 (0.018)	-0.043 (0.027)	-0.071 (0.03)	-0.026 (0.078)	-0.1 (0.053)	HDL
rs1532085	15	58683366	1,00	A	G	0.107 (0.004)	0.012 (0.019)	0.017 (0.027)	0.02 (0.031)	0.037 (0.081)	-0.037 (0.052)	HDL
rs2652834	15	63396867	0,99	G	A	0.028 (0.004)	0.02 (0.022)	-0.023 (0.033)	0.083 (0.036)	-0.104 (0.097)	0.044 (0.065)	HDL
rs1121980	16	53809247	0,98	G	A	0.02 (0.003)	-0.024 (0.018)	-0.017 (0.027)	-0.057 (0.03)	0.05 (0.077)	0.016 (0.052)	HDL
rs3764261	16	56993324	1,00	A	C	0.241 (0.004)	0.025 (0.019)	0.05 (0.028)	0.017 (0.032)	-0.15 (0.082)	0.038 (0.055)	HDL
rs16942887	16	67928042	1,00	A	G	0.083 (0.005)	-0.048 (0.027)	-0.047 (0.039)	-0.036 (0.045)	0.095 (0.119)	-0.153 (0.078)	HDL
rs2925979	16	81534790	1,00	C	T	0.035 (0.004)	0.004 (0.02)	-0.004 (0.03)	0.016 (0.032)	0.031 (0.087)	-0.016 (0.055)	HDL
rs1877031	17	37814080	1,00	A	G	0.034 (0.004)	0.027 (0.019)	0.042 (0.029)	0.01 (0.032)	0.1 (0.081)	-0.012 (0.054)	HDL
rs4148005	17	66882466	1,00	T	G	0.028 (0.004)	0.002 (0.02)	-0.032 (0.029)	0.036 (0.032)	-0.018 (0.085)	0.032 (0.055)	HDL
rs4129767	17	76403984	1,00	A	G	0.024 (0.003)	0.024 (0.018)	0.013 (0.027)	0.05 (0.03)	-0.09 (0.079)	0.04 (0.052)	HDL
rs7241918	18	47160953	1,00	T	G	0.09 (0.006)	0.051 (0.024)	0.052 (0.036)	0.088 (0.041)	0.021 (0.103)	-0.041 (0.068)	HDL
rs12967135	18	57849023	0,99	G	A	0.026 (0.004)	-0.046 (0.021)	-0.064 (0.032)	-0.013 (0.035)	-0.095 (0.092)	-0.062 (0.06)	HDL
rs7255436	19	8433196	1,00	A	C	0.032 (0.005)	-0.007 (0.018)	0.002 (0.027)	-0.045 (0.03)	-0.043 (0.076)	0.085 (0.051)	HDL
rs737337	19	11347493	0,99	T	C	0.056 (0.006)	0.005 (0.032)	-0.016 (0.046)	-0.006 (0.055)	-0.037 (0.142)	0.15 (0.095)	HDL
rs731839	19	33899065	0,99	A	G	0.022 (0.004)	-0.006 (0.019)	-0.017 (0.029)	-0.027 (0.031)	0.037 (0.083)	0.073 (0.054)	HDL
rs4420638	19	45422946	0,83	A	G	0.067 (0.007)	0.05 (0.026)	NA (NA)	0.013 (0.043)	-0.082 (0.11)	0.119 (0.064)	HDL
rs17695224	19	52324216	0,99	G	A	0.029 (0.004)	0.006 (0.021)	0.055 (0.031)	-0.079 (0.034)	0.008 (0.086)	0.079 (0.057)	HDL
rs103294	19	54797848	1,00	T	C	0.052 (0.004)	-0.03 (0.022)	-0.01 (0.034)	-0.076 (0.037)	-0.073 (0.097)	0.045 (0.061)	HDL
rs1800961	20	43042364	0,96	C	T	0.127 (0.01)	-0.015 (0.054)	0.028 (0.084)	-0.045 (0.084)	-0.472 (0.23)	0.156 (0.157)	HDL
rs6065906	20	44554015	1,00	T	C	0.059 (0.004)	-0.014 (0.023)	0.033 (0.034)	-0.066 (0.038)	0.107 (0.096)	-0.092 (0.066)	HDL
rs10903129	1	25768937	1,00	G	A	0.033 (0.004)	0.011 (0.018)	0.028 (0.027)	0.029 (0.03)	-0.053 (0.076)	-0.077 (0.051)	LDL
rs12748152	1	27138393	0,99	T	C	0.05 (0.007)	0.073 (0.032)	0.054 (0.047)	0.171 (0.054)	-0.008 (0.15)	-0.108 (0.093)	LDL
rs2479409	1	55504650	0,90	G	A	0.064 (0.004)	-0.016 (0.02)	NA (NA)	-0.006 (0.032)	0.01 (0.088)	-0.005 (0.054)	LDL
rs2131925	1	63025942	1,00	T	G	0.049 (0.004)	-0.019 (0.019)	0.01 (0.029)	-0.044 (0.032)	-0.063 (0.082)	-0.03 (0.053)	LDL
rs629301	1	109818306	1,00	T	G	0.167 (0.005)	0.026 (0.022)	0.016 (0.032)	0.038 (0.036)	-0.032 (0.094)	0.052 (0.061)	LDL
rs267733	1	150958836	1,00	A	G	0.033 (0.005)	-0.031 (0.025)	-0.014 (0.037)	-0.059 (0.041)	-0.004 (0.109)	-0.023 (0.071)	LDL
rs2642442	1	220973563	0,92	T	C	0.036 (0.005)	0.002 (0.02)	-0.027 (0.03)	0.012 (0.033)	-0.043 (0.088)	0.094 (0.056)	LDL
rs484084	1	234857676	0,96	C	T	0.033 (0.005)	0.004 (0.018)	NA (NA)	0.031 (0.03)	-0.076 (0.078)	0.007 (0.052)	LDL
rs1367117	2	21263900	0,96	A	G	0.119 (0.004)	0.032 (0.02)	0.031 (0.03)	0.023 (0.032)	0.194 (0.085)	-0.005 (0.054)	LDL
rs4299376	2	44072576	0,98	G	T	0.081 (0.004)	0.012 (0.02)	NA (NA)	-0.016 (0.032)	0.134 (0.082)	0.067 (0.055)	LDL
rs2710642	2	63149557	1,00	A	G	0.024 (0.004)	0.012 (0.019)	0.004 (0.028)	0.01 (0.031)	0.097 (0.08)	0.01 (0.054)	LDL
rs10490626	2	118835841	1,00	G	A	0.051 (0.007)	-0.07 (0.033)	-0.045 (0.048)	-0.112 (0.055)	0.036 (0.144)	-0.096 (0.096)	LDL
rs2030746	2	121309488	1,00	T	C	0.021 (0.004)	-0.003 (0.018)	0.012 (0.027)	-0.025 (0.03)	-0.07 (0.081)	0.037 (0.052)	LDL
rs1250229	2	216304384	0,96	C	T	0.024 (0.004)	-0.007 (0.021)	0.032 (0.031)	-0.064 (0.035)	-0.013 (0.091)	0.028 (0.06)	LDL
rs11563251	2	234679384	1,00	T	C	0.034 (0.006)	0.034 (0.03)	0.019 (0.045)	0.087 (0.048)	0.02 (0.118)	-0.058 (0.08)	LDL
rs7640978	3	32533010	0,98	C	T	0.039 (0.007)	0.008 (0.033)	-0.013 (0.048)	-0.007 (0.054)	0.307 (0.143)	0.004 (0.092)	LDL
rs17404153	3	132163200	1,00	G	T	0.034 (0.005)	-0.03 (0.027)	-0.044 (0.04)	-0.023 (0.045)	-0.074 (0.114)	0.026 (0.078)	LDL
rs6831256	4	3473139	1,00	G	A	0.019 (0.004)	-0.025 (0.018)	-0.01 (0.027)	-0.032 (0.03)	-0.108 (0.078)	-0.022 (0.052)	LDL
rs12916	5	74656539	1,00	C	T	0.073 (0.004)	0.003 (0.018)	-0.007 (0.027)	-0.025 (0.03)	0.159 (0.078)	0.054 (0.052)	LDL
rs4530754	5	122855416	1,00	A	G	0.028 (0.004)	0.005 (0.018)	0.01 (0.026)	-0.025 (0.03)	0.138 (0.078)	0.02 (0.051)	LDL
rs6882076	5	156390297	0,99	C	T	0.046 (0.004)	-0.028 (0.019)	-0.021 (0.028)	-0.062 (0.031)	0.061 (0.08)	0.002 (0.053)	LDL
rs3757354	6	16127407	1,00	C	T	0.038 (0.004)	-0.009 (0.021)	-0.012 (0.031)	0.033 (0.036)	-0.095 (0.094)	-0.085 (0.063)	LDL
rs1800562	6	26093141	0,99	G	A	0.062 (0.008)	0.011 (0.04)	-0.078 (0.064)	0.109 (0.063)	-0.06 (0.154)	0.01 (0.1)	LDL
rs3798236	6	116309649	0,99	T	C	0.023 (0.004)	0.024 (0.019)	0.027 (0.028)	-0.002 (0.032)	0.066 (0.08)	0.068 (0.053)	LDL
rs1564348	6	160578860	1,00	C	T	0.048 (0.005)	-0.013 (0.024)	-0.084 (0.037)	0.032 (0.04)	-0.041 (0.104)	0.114 (0.068)	LDL
rs12670798	7	21607352	0,99	C	T	0.034 (0.004)	-0.005 (0.021)	0.044 (0.032)	-0.062 (0.036)	-0.012 (0.092)	-0.017 (0.062)	LDL
rs4722551	7	25991826	0,99	C	T	0.039 (0.005)	-0.02 (0.024)	-0.06 (0.035)	0.023 (0.04)	-0.074 (0.109)	0.027 (0.07)	LDL

rs9987289	8	9183358	0,99	G	A	0.071 (0.007)	0.007 (0.032)	0.022 (0.048)	-0.022 (0.052)	0.226 (0.139)	-0.05 (0.092)	LDL
rs10102164	8	55421614	0,99	A	G	0.032 (0.004)	-0.087 (0.023)	-0.073 (0.035)	-0.114 (0.038)	-0.042 (0.1)	-0.073 (0.063)	LDL
rs2081687	8	59388565	1,00	T	C	0.031 (0.005)	0.06 (0.019)	0.056 (0.028)	0.067 (0.031)	0.155 (0.084)	0.012 (0.054)	LDL
rs10808546	8	126495818	1,00	C	T	0.054 (0.004)	-0.025 (0.018)	-0.059 (0.027)	-0.006 (0.03)	0.025 (0.08)	0.024 (0.051)	LDL
rs11136341	8	145043543	0,94	G	A	0.045 (0.006)	0.058 (0.019)	0.085 (0.029)	0.042 (0.031)	-0.077 (0.082)	0.068 (0.054)	LDL
rs3780181	9	2640759	1,00	A	G	0.044 (0.007)	0.03 (0.037)	0.125 (0.056)	-0.048 (0.059)	-0.294 (0.166)	0.065 (0.103)	LDL
rs2255141	10	113933886	1,00	A	G	0.03 (0.004)	0.016 (0.02)	-0.019 (0.03)	0.038 (0.033)	-0.027 (0.086)	0.097 (0.058)	LDL
rs11220462	11	126243952	1,00	A	G	0.059 (0.006)	0.024 (0.026)	0.039 (0.038)	0.017 (0.042)	0.013 (0.112)	-0.013 (0.076)	LDL
rs11065987	12	112072424	0,96	A	G	0.027 (0.004)	0.008 (0.019)	0.03 (0.028)	-0.038 (0.03)	0.07 (0.08)	0.05 (0.052)	LDL
rs1169288	12	121416650	0,95	C	A	0.038 (0.004)	0.044 (0.02)	0.027 (0.029)	0.05 (0.032)	0.046 (0.084)	0.091 (0.056)	LDL
rs4942486	13	32953388	1,00	T	C	0.024 (0.004)	-0.013 (0.018)	0.002 (0.027)	-0.032 (0.03)	0.062 (0.076)	-0.045 (0.051)	LDL
rs8017377	14	24883887	0,99	A	G	0.03 (0.004)	0.006 (0.018)	-0.018 (0.027)	0.016 (0.03)	0.032 (0.076)	0.054 (0.052)	LDL
rs3764261	16	56993324	1,00	C	A	0.053 (0.004)	-0.025 (0.019)	-0.05 (0.028)	-0.017 (0.032)	0.15 (0.082)	-0.038 (0.055)	LDL
rs2000999	16	72108093	0,99	A	G	0.065 (0.005)	0.031 (0.022)	0.035 (0.033)	0.03 (0.038)	0.006 (0.097)	0.028 (0.065)	LDL
rs314253	17	7091650	1,00	T	C	0.024 (0.004)	-0.024 (0.019)	-0.01 (0.028)	-0.036 (0.031)	-0.051 (0.081)	-0.029 (0.054)	LDL
rs7206971	17	45425115	0,98	A	G	0.029 (0.006)	0.004 (0.018)	0.053 (0.027)	-0.038 (0.03)	0.038 (0.077)	-0.064 (0.051)	LDL
rs6511720	19	11202306	0,98	G	T	0.221 (0.006)	-0.042 (0.029)	-0.033 (0.044)	-0.048 (0.047)	-0.009 (0.113)	-0.072 (0.078)	LDL
rs10401969	19	19407718	0,99	T	C	0.118 (0.007)	-0.05 (0.034)	-0.077 (0.051)	-0.028 (0.057)	-0.179 (0.146)	0.039 (0.095)	LDL
rs4420638	19	45422946	0,83	G	A	0.225 (0.008)	-0.05 (0.026)	NA (NA)	-0.013 (0.043)	0.082 (0.11)	-0.119 (0.064)	LDL
rs364585	20	12962718	1,00	G	A	0.025 (0.004)	-0.029 (0.019)	-0.039 (0.028)	-0.024 (0.031)	-0.102 (0.079)	0.028 (0.053)	LDL
rs2328223	20	17845921	0,97	C	A	0.03 (0.005)	-0.003 (0.023)	-0.002 (0.034)	-0.017 (0.038)	-0.033 (0.096)	0.048 (0.066)	LDL
rs2902940	20	39091487	0,99	A	G	0.027 (0.004)	0.033 (0.02)	0.032 (0.03)	0.036 (0.033)	-0.106 (0.085)	0.09 (0.056)	LDL
rs6016505	20	39678289	0,99	T	C	0.04 (0.005)	0.001 (0.018)	0.003 (0.028)	-0.008 (0.03)	0.09 (0.077)	-0.023 (0.051)	LDL
rs5763662	22	30378703	0,99	T	C	0.077 (0.012)	-0.04 (0.058)	-0.046 (0.085)	-0.102 (0.097)	0.145 (0.25)	0.099 (0.178)	LDL
rs4253772	22	46627603	1,00	T	C	0.031 (0.006)	0.002 (0.029)	0.026 (0.043)	-0.016 (0.048)	-0.184 (0.117)	0.063 (0.082)	LDL
rs1077514	1	23766233	0,98	T	C	0.03 (0.005)	0.007 (0.026)	0.046 (0.038)	-0.009 (0.043)	-0.148 (0.108)	-0.018 (0.074)	Total cholesterol
rs10903129	1	25768937	1,00	G	A	0.029 (0.004)	0.011 (0.018)	0.028 (0.027)	0.029 (0.03)	-0.053 (0.076)	-0.077 (0.051)	Total cholesterol
rs2479409	1	55504650	0,90	G	A	0.054 (0.004)	-0.016 (0.02)	NA (NA)	-0.006 (0.032)	0.01 (0.088)	-0.005 (0.054)	Total cholesterol
rs2131925	1	63025942	1,00	T	G	0.075 (0.004)	-0.019 (0.019)	0.01 (0.029)	-0.044 (0.032)	-0.063 (0.082)	-0.03 (0.053)	Total cholesterol
rs7515577	1	93009438	1,00	A	C	0.037 (0.006)	-0.006 (0.022)	-0.023 (0.033)	0.018 (0.037)	0.062 (0.098)	-0.04 (0.063)	Total cholesterol
rs629301	1	109818306	1,00	T	G	0.134 (0.005)	0.026 (0.022)	0.016 (0.032)	0.038 (0.036)	-0.032 (0.094)	0.052 (0.061)	Total cholesterol
rs2642442	1	220973563	0,92	T	C	0.035 (0.005)	0.002 (0.02)	-0.027 (0.03)	0.012 (0.033)	-0.043 (0.088)	0.094 (0.056)	Total cholesterol
rs484084	1	234857676	0,96	C	T	0.035 (0.005)	0.004 (0.018)	NA (NA)	0.031 (0.03)	-0.076 (0.078)	0.007 (0.052)	Total cholesterol
rs1367117	2	21263900	0,96	A	G	0.1 (0.004)	0.032 (0.02)	0.031 (0.03)	0.023 (0.032)	0.194 (0.085)	-0.005 (0.054)	Total cholesterol
rs1260326	2	27730940	1,00	T	C	0.051 (0.004)	-0.004 (0.018)	0.01 (0.027)	-0.016 (0.03)	-0.117 (0.08)	0.03 (0.053)	Total cholesterol
rs4299376	2	44072576	0,98	G	T	0.079 (0.004)	0.012 (0.02)	NA (NA)	-0.016 (0.032)	0.134 (0.082)	0.067 (0.055)	Total cholesterol
rs10490626	2	118835841	1,00	G	A	0.042 (0.007)	-0.07 (0.033)	-0.045 (0.048)	-0.112 (0.055)	0.036 (0.144)	-0.096 (0.096)	Total cholesterol
rs2030746	2	121309488	1,00	T	C	0.02 (0.004)	-0.003 (0.018)	0.012 (0.027)	-0.025 (0.03)	-0.07 (0.081)	0.037 (0.052)	Total cholesterol
rs7570971	2	135837906	0,93	A	C	0.03 (0.004)	-0.02 (0.02)	-0.035 (0.031)	-0.01 (0.033)	-0.054 (0.086)	0.014 (0.057)	Total cholesterol
rs2287623	2	169830155	1,00	G	A	0.027 (0.004)	0.008 (0.018)	-0.028 (0.027)	0.043 (0.03)	-0.065 (0.079)	0.066 (0.053)	Total cholesterol
rs11694172	2	203532304	1,00	G	A	0.028 (0.004)	0.013 (0.021)	-0.014 (0.031)	0.039 (0.035)	0.161 (0.092)	-0.03 (0.06)	Total cholesterol
rs11563251	2	234679384	1,00	T	C	0.037 (0.006)	0.034 (0.03)	0.019 (0.045)	0.087 (0.048)	0.02 (0.118)	-0.058 (0.08)	Total cholesterol
rs7956	3	12624763	1,00	T	C	0.035 (0.006)	-0.011 (0.022)	0.021 (0.033)	-0.01 (0.036)	-0.022 (0.091)	-0.118 (0.061)	Total cholesterol
rs7640978	3	32533010	0,98	C	T	0.038 (0.007)	0.008 (0.033)	-0.013 (0.048)	-0.007 (0.054)	0.307 (0.143)	0.004 (0.092)	Total cholesterol
rs13315871	3	58381287	1,00	G	A	0.036 (0.006)	-0.008 (0.031)	-0.041 (0.047)	-0.046 (0.052)	0.107 (0.134)	0.178 (0.089)	Total cholesterol
rs6831256	4	3473139	1,00	G	A	0.023 (0.004)	-0.025 (0.018)	-0.01 (0.027)	-0.032 (0.03)	-0.108 (0.078)	-0.022 (0.052)	Total cholesterol
rs12916	5	74656539	1,00	C	T	0.068 (0.004)	0.003 (0.018)	-0.007 (0.027)	-0.025 (0.03)	0.159 (0.078)	0.054 (0.052)	Total cholesterol
rs4530754	5	122855416	1,00	A	G	0.023 (0.004)	0.005 (0.018)	0.01 (0.026)	-0.025 (0.03)	0.138 (0.078)	0.02 (0.051)	Total cholesterol
rs6882076	5	156390297	0,99	C	T	0.051 (0.004)	-0.028 (0.019)	-0.021 (0.028)	-0.062 (0.031)	0.061 (0.08)	0.002 (0.053)	Total cholesterol

rs3757354	6	16127407	1,00	C	T	0.035 (0.004)	-0.009 (0.021)	-0.012 (0.031)	0.033 (0.036)	-0.095 (0.094)	-0.085 (0.063)	Total cholesterol
rs1800562	6	26093141	0,99	G	A	0.056 (0.008)	0.011 (0.04)	-0.078 (0.064)	0.109 (0.063)	-0.06 (0.154)	0.01 (0.1)	Total cholesterol
rs2814982	6	34546560	1,00	C	T	0.044 (0.006)	-0.048 (0.028)	0.011 (0.042)	-0.126 (0.046)	-0.154 (0.122)	0.03 (0.083)	Total cholesterol
rs2758886	6	39250837	1,00	A	G	0.023 (0.004)	0.045 (0.02)	0.056 (0.03)	0.031 (0.033)	0.191 (0.084)	-0.017 (0.055)	Total cholesterol
rs3798236	6	116309649	0,99	T	C	0.026 (0.004)	0.024 (0.019)	0.027 (0.028)	-0.002 (0.032)	0.066 (0.08)	0.068 (0.053)	Total cholesterol
rs9376090	6	135411228	0,99	T	C	0.025 (0.004)	-0.025 (0.02)	-0.036 (0.03)	-0.021 (0.034)	0.028 (0.089)	-0.02 (0.059)	Total cholesterol
rs1564348	6	160578860	1,00	C	T	0.049 (0.005)	-0.013 (0.024)	-0.084 (0.037)	0.032 (0.04)	-0.041 (0.104)	0.114 (0.068)	Total cholesterol
rs1997243	7	1083777	1,00	G	A	0.033 (0.005)	0.053 (0.025)	0.069 (0.035)	0.001 (0.041)	0.035 (0.11)	0.153 (0.071)	Total cholesterol
rs12670798	7	21607352	0,99	C	T	0.036 (0.004)	-0.005 (0.021)	0.044 (0.032)	-0.062 (0.036)	-0.012 (0.092)	-0.017 (0.062)	Total cholesterol
rs4722551	7	25991826	0,99	C	T	0.029 (0.005)	-0.02 (0.024)	-0.06 (0.035)	0.023 (0.04)	-0.074 (0.109)	0.027 (0.07)	Total cholesterol
rs9987289	8	9183358	0,99	G	A	0.084 (0.006)	0.007 (0.032)	0.022 (0.048)	-0.022 (0.052)	0.226 (0.139)	-0.05 (0.092)	Total cholesterol
rs1495741	8	18272881	1,00	G	A	0.032 (0.006)	0.01 (0.022)	-0.004 (0.032)	0.017 (0.036)	-0.041 (0.094)	0.066 (0.061)	Total cholesterol
rs10102164	8	55421614	0,99	A	G	0.03 (0.004)	-0.087 (0.023)	-0.073 (0.035)	-0.114 (0.038)	-0.042 (0.1)	-0.073 (0.063)	Total cholesterol
rs2081687	8	59388565	1,00	T	C	0.038 (0.005)	0.06 (0.019)	0.056 (0.028)	0.067 (0.031)	0.155 (0.084)	0.012 (0.054)	Total cholesterol
rs2737229	8	116648565	1,00	A	C	0.029 (0.004)	-0.004 (0.02)	-0.045 (0.03)	0.04 (0.032)	-0.082 (0.084)	0.044 (0.055)	Total cholesterol
rs10808546	8	126495818	1,00	C	T	0.059 (0.004)	-0.025 (0.018)	-0.059 (0.027)	-0.006 (0.03)	0.025 (0.08)	0.024 (0.051)	Total cholesterol
rs11136341	8	145043543	0,94	G	A	0.038 (0.006)	0.058 (0.019)	0.085 (0.029)	0.042 (0.031)	-0.077 (0.082)	0.068 (0.054)	Total cholesterol
rs3780181	9	2640759	1,00	A	G	0.044 (0.007)	0.03 (0.037)	0.125 (0.056)	-0.048 (0.059)	-0.294 (0.166)	0.065 (0.103)	Total cholesterol
rs638491	9	15290012	0,99	G	A	0.033 (0.005)	0.05 (0.024)	0.016 (0.036)	0.089 (0.041)	-0.002 (0.104)	0.083 (0.07)	Total cholesterol
rs1883025	9	107664301	0,98	C	T	0.067 (0.004)	0.005 (0.021)	-0.006 (0.032)	0.015 (0.034)	-0.06 (0.088)	0.039 (0.058)	Total cholesterol
rs10904908	10	17260290	0,96	G	A	0.025 (0.004)	-0.043 (0.019)	-0.018 (0.028)	-0.067 (0.03)	-0.029 (0.08)	-0.063 (0.052)	Total cholesterol
rs970548	10	46013277	1,00	C	A	0.025 (0.004)	-0.001 (0.021)	-0.008 (0.031)	-0.002 (0.034)	0.132 (0.091)	-0.03 (0.058)	Total cholesterol
rs2255141	10	113933886	1,00	A	G	0.031 (0.004)	0.016 (0.02)	-0.019 (0.03)	0.038 (0.033)	-0.027 (0.086)	0.097 (0.058)	Total cholesterol
rs10128711	11	18632984	1,00	C	T	0.031 (0.004)	-0.017 (0.02)	-0.071 (0.03)	0.024 (0.034)	0.041 (0.086)	0.042 (0.058)	Total cholesterol
rs174546	11	61569830	1,00	C	T	0.048 (0.004)	0.026 (0.019)	0.009 (0.028)	0.03 (0.032)	0.093 (0.078)	0.041 (0.054)	Total cholesterol
rs3741298	11	116657561	0,97	C	T	0.067 (0.006)	-0.017 (0.023)	0.003 (0.034)	-0.014 (0.037)	-0.094 (0.095)	-0.066 (0.066)	Total cholesterol
rs11603023	11	118486067	1,00	T	C	0.022 (0.004)	-0.005 (0.018)	0.017 (0.028)	-0.015 (0.03)	-0.065 (0.078)	-0.026 (0.05)	Total cholesterol
rs11220462	11	126243952	1,00	A	G	0.047 (0.006)	0.024 (0.026)	0.039 (0.038)	0.017 (0.042)	0.013 (0.112)	-0.013 (0.076)	Total cholesterol
rs11065987	12	112072424	0,96	A	G	0.031 (0.004)	0.008 (0.019)	0.03 (0.028)	-0.038 (0.03)	0.07 (0.08)	0.05 (0.052)	Total cholesterol
rs1169288	12	121416650	0,95	C	A	0.032 (0.004)	0.044 (0.02)	0.027 (0.029)	0.05 (0.032)	0.046 (0.084)	0.091 (0.056)	Total cholesterol
rs1532085	15	58683366	1,00	A	G	0.054 (0.004)	0.012 (0.019)	0.017 (0.027)	0.02 (0.031)	0.037 (0.081)	-0.037 (0.052)	Total cholesterol
rs3764261	16	56993324	1,00	A	C	0.05 (0.004)	0.025 (0.019)	0.05 (0.028)	0.017 (0.032)	-0.15 (0.082)	0.038 (0.055)	Total cholesterol
rs2000999	16	72108093	0,99	A	G	0.062 (0.004)	0.031 (0.022)	0.035 (0.033)	0.03 (0.038)	0.006 (0.097)	0.028 (0.065)	Total cholesterol
rs314253	17	7091650	1,00	T	C	0.023 (0.004)	-0.024 (0.019)	-0.01 (0.028)	-0.036 (0.031)	-0.051 (0.081)	-0.029 (0.054)	Total cholesterol
rs7206971	17	45425115	0,98	A	G	0.03 (0.005)	0.004 (0.018)	0.053 (0.027)	-0.038 (0.03)	0.038 (0.077)	-0.064 (0.051)	Total cholesterol
rs7241918	18	47160953	1,00	T	G	0.058 (0.007)	0.051 (0.024)	0.052 (0.036)	0.088 (0.041)	0.021 (0.103)	-0.041 (0.068)	Total cholesterol
rs6511720	19	11202306	0,98	G	T	0.185 (0.006)	-0.042 (0.029)	-0.033 (0.044)	-0.048 (0.047)	-0.009 (0.113)	-0.072 (0.078)	Total cholesterol
rs10401969	19	19407718	0,99	T	C	0.137 (0.007)	-0.05 (0.034)	-0.077 (0.051)	-0.028 (0.057)	-0.179 (0.146)	0.039 (0.095)	Total cholesterol
rs4420638	19	45422946	0,83	G	A	0.197 (0.007)	-0.05 (0.026)	NA (NA)	-0.013 (0.043)	0.082 (0.11)	-0.119 (0.064)	Total cholesterol
rs492602	19	49206417	1,00	G	A	0.031 (0.004)	-0.019 (0.018)	-0.009 (0.027)	-0.015 (0.03)	0.043 (0.076)	-0.093 (0.05)	Total cholesterol
rs2277862	20	34152782	1,00	C	T	0.035 (0.005)	0.02 (0.026)	0.053 (0.038)	0 (0.042)	-0.066 (0.106)	-0.001 (0.074)	Total cholesterol
rs2902940	20	39091487	0,99	A	G	0.024 (0.004)	0.033 (0.02)	0.032 (0.03)	0.036 (0.033)	-0.106 (0.085)	0.09 (0.056)	Total cholesterol
rs6016505	20	39678289	0,99	T	C	0.037 (0.005)	0.001 (0.018)	0.003 (0.028)	-0.008 (0.03)	0.09 (0.077)	-0.023 (0.051)	Total cholesterol
rs1800961	20	43042364	0,96	C	T	0.106 (0.01)	-0.015 (0.054)	0.028 (0.084)	-0.045 (0.084)	-0.472 (0.23)	0.156 (0.157)	Total cholesterol
rs138777	22	35711098	1,00	A	G	0.021 (0.004)	-0.022 (0.019)	-0.022 (0.028)	-0.02 (0.031)	-0.016 (0.081)	-0.027 (0.054)	Total cholesterol
rs4253772	22	46627603	1,00	T	C	0.032 (0.006)	0.002 (0.029)	0.026 (0.043)	-0.016 (0.048)	-0.184 (0.117)	0.063 (0.082)	Total cholesterol
rs12748152	1	27138393	0,99	T	C	0.037 (0.006)	0.073 (0.032)	0.054 (0.047)	0.171 (0.054)	-0.008 (0.15)	-0.108 (0.093)	Triglycerides
rs2131925	1	63025942	1,00	T	G	0.066 (0.004)	-0.019 (0.019)	0.01 (0.029)	-0.044 (0.032)	-0.063 (0.082)	-0.03 (0.053)	Triglycerides
rs4846914	1	230295691	0,97	G	A	0.04 (0.003)	-0.019 (0.019)	-0.033 (0.028)	-0.004 (0.03)	0.104 (0.081)	-0.073 (0.054)	Triglycerides

rs1042034	2	21225281	1,00	T	C	0.07 (0.004)	0.007 (0.022)	-0.044 (0.032)	0.049 (0.036)	0.106 (0.096)	0.031 (0.064)	Triglycerides
rs1260326	2	27730940	1,00	T	C	0.115 (0.003)	-0.004 (0.018)	0.01 (0.027)	-0.016 (0.03)	-0.117 (0.08)	0.03 (0.053)	Triglycerides
rs10195252	2	165513091	0,99	T	C	0.028 (0.004)	0.04 (0.018)	0.011 (0.027)	0.05 (0.03)	0.167 (0.079)	0.06 (0.053)	Triglycerides
rs2972146	2	227100698	1,00	T	G	0.028 (0.003)	0.034 (0.019)	0.012 (0.028)	0.055 (0.031)	0.103 (0.082)	0.021 (0.053)	Triglycerides
rs645040	3	135926622	1,00	T	G	0.029 (0.004)	0.014 (0.022)	-0.011 (0.034)	0.07 (0.036)	0.062 (0.092)	-0.084 (0.061)	Triglycerides
rs6831256	4	3473139	1,00	G	A	0.026 (0.004)	-0.025 (0.018)	-0.01 (0.027)	-0.032 (0.03)	-0.108 (0.078)	-0.022 (0.052)	Triglycerides
rs442177	4	88030261	0,99	T	G	0.031 (0.003)	0.014 (0.018)	-0.014 (0.027)	0.01 (0.03)	0.025 (0.08)	0.125 (0.053)	Triglycerides
rs9686661	5	55861786	0,97	T	C	0.038 (0.004)	0.019 (0.024)	0.03 (0.036)	0.03 (0.038)	-0.119 (0.097)	0.013 (0.068)	Triglycerides
rs6882076	5	156390297	0,99	C	T	0.029 (0.004)	-0.028 (0.019)	-0.021 (0.028)	-0.062 (0.031)	0.061 (0.08)	0.002 (0.053)	Triglycerides
rs998584	6	43757896	0,84	A	C	0.029 (0.004)	0.028 (0.02)	0.051 (0.03)	-0.002 (0.032)	0.007 (0.083)	0.054 (0.055)	Triglycerides
rs1936800	6	127436064	0,98	T	C	0.018 (0.003)	-0.018 (0.018)	-0.027 (0.027)	-0.024 (0.03)	0.01 (0.078)	0.018 (0.051)	Triglycerides
rs4722551	7	25991826	0,99	T	C	0.027 (0.004)	0.02 (0.024)	0.06 (0.035)	-0.023 (0.04)	0.074 (0.109)	-0.027 (0.07)	Triglycerides
rs17145738	7	72982874	0,96	C	T	0.115 (0.005)	0.084 (0.029)	0.14 (0.044)	0.018 (0.047)	0.168 (0.122)	0.062 (0.079)	Triglycerides
rs38855	7	116358044	1,00	A	G	0.019 (0.003)	0.006 (0.018)	-0.02 (0.027)	0.042 (0.03)	-0.036 (0.078)	0.014 (0.052)	Triglycerides
rs2271357	8	10683623	1,00	A	G	0.022 (0.004)	-0.011 (0.019)	0.009 (0.028)	-0.018 (0.031)	-0.054 (0.079)	-0.042 (0.053)	Triglycerides
rs1495741	8	18272881	1,00	G	A	0.04 (0.006)	0.01 (0.022)	-0.004 (0.032)	0.017 (0.036)	-0.041 (0.094)	0.066 (0.061)	Triglycerides
rs12678919	8	19844222	1,00	A	G	0.17 (0.006)	-0.056 (0.03)	-0.057 (0.045)	-0.05 (0.049)	-0.105 (0.134)	-0.046 (0.086)	Triglycerides
rs10808546	8	126495818	1,00	C	T	0.077 (0.003)	-0.025 (0.018)	-0.059 (0.027)	-0.006 (0.03)	0.025 (0.08)	0.024 (0.051)	Triglycerides
rs1832007	10	5254847	1,00	A	G	0.033 (0.005)	-0.016 (0.025)	0.024 (0.038)	-0.086 (0.042)	0.285 (0.103)	-0.094 (0.07)	Triglycerides
rs7080386	10	65048306	0,98	C	A	0.028 (0.005)	0.009 (0.019)	-0.019 (0.028)	-0.005 (0.03)	0.086 (0.077)	0.109 (0.052)	Triglycerides
rs2068888	10	94839642	1,00	G	A	0.024 (0.003)	0.002 (0.018)	-0.009 (0.027)	0.017 (0.03)	-0.091 (0.078)	0.037 (0.052)	Triglycerides
rs174546	11	61569830	1,00	T	C	0.045 (0.003)	-0.026 (0.019)	-0.009 (0.028)	-0.03 (0.032)	-0.093 (0.078)	-0.041 (0.054)	Triglycerides
rs3741298	11	116657561	0,97	C	T	0.147 (0.006)	-0.017 (0.023)	0.003 (0.034)	-0.014 (0.037)	-0.094 (0.095)	-0.066 (0.066)	Triglycerides
rs11613352	12	57792580	0,99	C	T	0.028 (0.004)	0 (0.022)	0.075 (0.033)	-0.084 (0.036)	-0.008 (0.091)	-0.008 (0.06)	Triglycerides
rs4765127	12	124460167	1,00	G	T	0.029 (0.005)	0.019 (0.019)	0.024 (0.028)	0.014 (0.032)	-0.007 (0.081)	0.031 (0.054)	Triglycerides
rs2412710	15	42683787	0,90	A	G	0.099 (0.013)	0.112 (0.067)	0.085 (0.102)	0.278 (0.106)	-0.205 (0.297)	-0.214 (0.192)	Triglycerides
rs2929282	15	44245931	0,98	T	A	0.072 (0.012)	0.042 (0.041)	0.054 (0.061)	0.05 (0.066)	-0.133 (0.182)	0.051 (0.129)	Triglycerides
rs1532085	15	58683366	1,00	A	G	0.031 (0.003)	0.012 (0.019)	0.017 (0.027)	0.02 (0.031)	0.037 (0.081)	-0.037 (0.052)	Triglycerides
rs3198697	16	15129940	0,97	C	T	0.02 (0.003)	-0.014 (0.019)	0.004 (0.029)	-0.024 (0.03)	-0.014 (0.076)	-0.043 (0.052)	Triglycerides
rs11649653	16	30918487	0,89	C	G	0.027 (0.005)	-0.01 (0.02)	-0.007 (0.031)	-0.012 (0.031)	-0.024 (0.085)	-0.005 (0.052)	Triglycerides
rs1121980	16	53809247	0,98	A	G	0.017 (0.003)	0.024 (0.018)	0.017 (0.027)	0.057 (0.03)	-0.05 (0.077)	-0.016 (0.052)	Triglycerides
rs8077889	17	41878166	0,99	C	A	0.025 (0.004)	0.006 (0.022)	-0.018 (0.034)	0.019 (0.036)	0.179 (0.095)	-0.025 (0.062)	Triglycerides
rs7248104	19	7224431	1,00	G	A	0.022 (0.003)	-0.002 (0.018)	0.028 (0.027)	-0.042 (0.03)	-0.031 (0.078)	0.026 (0.052)	Triglycerides
rs10401969	19	19407718	0,99	T	C	0.121 (0.006)	-0.05 (0.034)	-0.077 (0.051)	-0.028 (0.057)	-0.179 (0.146)	0.039 (0.095)	Triglycerides
rs731839	19	33899065	0,99	G	A	0.022 (0.004)	0.006 (0.019)	0.017 (0.029)	0.027 (0.031)	-0.037 (0.083)	-0.073 (0.054)	Triglycerides
rs439401	19	45414451	1,00	C	T	0.066 (0.004)	-0.016 (0.019)	-0.013 (0.027)	-0.003 (0.031)	-0.041 (0.081)	-0.058 (0.054)	Triglycerides
rs6065906	20	44554015	1,00	C	T	0.053 (0.004)	0.014 (0.023)	-0.033 (0.034)	0.066 (0.038)	-0.107 (0.096)	0.092 (0.066)	Triglycerides
rs5756931	22	38546033	0,97	T	C	0.02 (0.004)	-0.006 (0.019)	-0.024 (0.028)	0.027 (0.031)	0.022 (0.083)	-0.048 (0.052)	Triglycerides
rs340874[a]	1	214159256	1,00	C	T	0.068 (0.01)	-0.023 (0.018)	0.017 (0.027)	-0.065 (0.03)	-0.106 (0.077)	-0.013 (0.052)	Type 2 diabetes
rs780094	2	27741237	1,00	C	T	0.058 (0.01)	0.003 (0.018)	-0.013 (0.027)	0.017 (0.03)	0.145 (0.08)	-0.042 (0.053)	Type 2 diabetes
rs77981966[a]	2	43777964	0,97	C	T	0.148 (0.022)	0.058 (0.035)	0.037 (0.053)	0.081 (0.058)	-0.068 (0.141)	0.127 (0.099)	Type 2 diabetes
rs243020	2	60585028	1,00	G	A	0.058 (0.01)	0.028 (0.018)	0.034 (0.027)	0.03 (0.03)	-0.149 (0.08)	0.069 (0.051)	Type 2 diabetes
rs75297654	2	165545615	1,00	C	T	0.104 (0.014)	0.015 (0.027)	-0.011 (0.04)	0.04 (0.045)	0.181 (0.117)	-0.037 (0.078)	Type 2 diabetes
rs2943645[b]	2	227099180	1,00	T	C	0.086 (0.009)	0.035 (0.019)	0.012 (0.028)	0.06 (0.031)	0.088 (0.082)	0.02 (0.053)	Type 2 diabetes
rs17036160[b]	3	12329783	0,98	C	T	0.131 (0.014)	0.03 (0.027)	0.026 (0.039)	0.014 (0.045)	0.088 (0.126)	0.077 (0.08)	Type 2 diabetes
rs17676309	3	64730121	0,96	C	T	0.068 (0.01)	-0.054 (0.019)	-0.018 (0.028)	-0.099 (0.03)	-0.063 (0.078)	-0.042 (0.052)	Type 2 diabetes
rs11708067[a]	3	123065778	0,98	A	G	0.104 (0.009)	-0.047 (0.022)	-0.053 (0.034)	-0.032 (0.036)	-0.009 (0.096)	-0.086 (0.061)	Type 2 diabetes
rs35510946	3	185518910	0,98	A	G	0.131 (0.009)	-0.009 (0.02)	0.017 (0.029)	-0.03 (0.032)	0.019 (0.083)	-0.052 (0.055)	Type 2 diabetes

rs1046314	4	6303955	0,99	A	G	0.086 (0.009)	0.012 (0.018)	0.011 (0.027)	0.01 (0.03)	0.073 (0.078)	-0.003 (0.052)	Type 2 diabetes
rs7732130	5	76435004	0,98	G	A	0.077 (0.014)	0.02 (0.02)	-0.019 (0.03)	0.033 (0.033)	0.138 (0.083)	0.058 (0.056)	Type 2 diabetes
rs35261542[a]	6	20675792	0,99	A	C	0.157 (0.013)	-0.016 (0.02)	-0.035 (0.03)	0.006 (0.034)	0.091 (0.086)	-0.058 (0.058)	Type 2 diabetes
rs10276674	7	14922007	0,98	C	T	0.077 (0.01)	-0.005 (0.024)	-0.039 (0.035)	0.029 (0.038)	0.061 (0.101)	-0.014 (0.068)	Type 2 diabetes
rs1974620	7	15065467	0,97	T	C	0.058 (0.01)	-0.014 (0.018)	-0.045 (0.027)	0.007 (0.03)	0.012 (0.076)	0.028 (0.053)	Type 2 diabetes
rs1513272	7	28200097	0,99	C	T	0.095 (0.009)	0.032 (0.018)	0.037 (0.027)	0.042 (0.03)	-0.027 (0.076)	0.012 (0.051)	Type 2 diabetes
rs878521	7	44255643	0,97	A	G	0.068 (0.01)	0.013 (0.022)	-0.015 (0.033)	0.029 (0.035)	-0.009 (0.09)	0.069 (0.059)	Type 2 diabetes
rs13266634[a]	8	118184783	1,00	C	T	0.113 (0.014)	-0.005 (0.02)	-0.014 (0.029)	0.02 (0.032)	-0.081 (0.082)	-0.01 (0.056)	Type 2 diabetes
rs10974438	9	4291928	0,95	C	A	0.068 (0.01)	0.001 (0.019)	0.024 (0.029)	-0.026 (0.031)	-0.067 (0.084)	0.03 (0.055)	Type 2 diabetes
rs10811660[a]	9	22134068	0,90	G	A	0.239 (0.016)	-0.002 (0.025)	-0.034 (0.037)	0.025 (0.043)	0.017 (0.102)	0.036 (0.076)	Type 2 diabetes
rs11257658	10	12309268	0,97	A	G	0.086 (0.009)	-0.005 (0.023)	-0.015 (0.034)	-0.019 (0.038)	0.067 (0.096)	0.035 (0.064)	Type 2 diabetes
rs11187140[a]	10	94466910	0,99	G	A	0.113 (0.009)	0.008 (0.019)	0.02 (0.028)	0.015 (0.031)	-0.159 (0.083)	0.015 (0.054)	Type 2 diabetes
rs7903146[a]	10	114758349	1,00	T	C	0.307 (0.011)	-0.046 (0.02)	-0.078 (0.03)	-0.041 (0.033)	0.124 (0.087)	-0.021 (0.057)	Type 2 diabetes
rs231360	11	2692249	0,97	T	C	0.077 (0.01)	-0.023 (0.019)	0.003 (0.028)	-0.059 (0.03)	0.051 (0.08)	-0.038 (0.053)	Type 2 diabetes
rs2237895	11	2857194	0,91	C	A	0.068 (0.01)	0.008 (0.019)	-0.01 (0.029)	0.016 (0.03)	-0.033 (0.084)	0.065 (0.055)	Type 2 diabetes
rs5215	11	17408630	1,00	C	T	0.068 (0.01)	0.016 (0.019)	0.027 (0.027)	0.016 (0.031)	0.026 (0.081)	-0.033 (0.054)	Type 2 diabetes
rs74333814	11	72457487	0,99	C	T	0.095 (0.014)	0.043 (0.025)	0.014 (0.037)	0.073 (0.041)	0.006 (0.104)	0.073 (0.069)	Type 2 diabetes
rs10830963	11	92708710	0,93	G	C	0.086 (0.011)	0.017 (0.021)	-0.007 (0.032)	0.038 (0.034)	0.059 (0.092)	0.017 (0.057)	Type 2 diabetes
rs2583941	12	66204598	0,99	A	G	0.104 (0.014)	-0.032 (0.03)	-0.085 (0.045)	-0.009 (0.05)	0.095 (0.128)	0.04 (0.086)	Type 2 diabetes
rs1169288	12	121416650	0,95	C	A	0.086 (0.009)	0.044 (0.02)	0.027 (0.029)	0.05 (0.032)	0.046 (0.084)	0.091 (0.056)	Type 2 diabetes
rs1800574	12	121416864	0,75	T	C	0.199 (0.035)	-0.033 (0.061)	NA (NA)	-0.097 (0.094)	-0.087 (0.252)	0.056 (0.164)	Type 2 diabetes
rs7172432	15	62396389	1,00	A	G	0.058 (0.01)	0.001 (0.018)	0.001 (0.027)	-0.005 (0.03)	-0.048 (0.08)	0.042 (0.052)	Type 2 diabetes
rs3803563	15	91531352	1,00	A	C	0.077 (0.01)	-0.021 (0.023)	-0.007 (0.034)	-0.054 (0.04)	-0.163 (0.1)	0.089 (0.069)	Type 2 diabetes
rs7193144	16	53810686	0,99	C	T	0.131 (0.009)	0.034 (0.018)	0.01 (0.027)	0.092 (0.03)	-0.034 (0.077)	-0.021 (0.052)	Type 2 diabetes
rs4430796	17	36098040	0,96	G	A	0.086 (0.009)	-0.015 (0.018)	-0.007 (0.028)	0.001 (0.03)	-0.167 (0.077)	-0.022 (0.051)	Type 2 diabetes
rs72999033	19	19366632	0,97	T	C	0.148 (0.018)	0.05 (0.036)	0.09 (0.053)	0.022 (0.06)	0.116 (0.158)	-0.037 (0.1)	Type 2 diabetes
rs4399645	19	46166073	0,89	T	C	0.058 (0.01)	-0.04 (0.02)	-0.085 (0.03)	0.006 (0.032)	-0.072 (0.085)	-0.005 (0.054)	Type 2 diabetes
rs2238689	19	46178661	0,95	C	T	0.077 (0.01)	-0.028 (0.019)	-0.016 (0.028)	-0.045 (0.03)	0.002 (0.079)	-0.029 (0.053)	Type 2 diabetes
rs1800961	20	43042364	0,96	T	C	0.148 (0.027)	0.015 (0.054)	-0.028 (0.084)	0.045 (0.084)	0.472 (0.23)	-0.156 (0.157)	Type 2 diabetes
rs340874	1	214159256	1,00	C	T	0.018 (0.003)	-0.023 (0.018)	0.017 (0.027)	-0.065 (0.03)	-0.106 (0.077)	-0.013 (0.052)	Fasting glucose
rs780094	2	27741237	1,00	C	T	0.036 (0.003)	0.003 (0.018)	-0.013 (0.027)	0.017 (0.03)	0.145 (0.08)	-0.042 (0.053)	Fasting glucose
rs560887	2	169763148	1,00	C	T	0.094 (0.003)	-0.003 (0.02)	0.019 (0.029)	0.007 (0.032)	-0.058 (0.086)	-0.091 (0.056)	Fasting glucose
rs11715915	3	49455330	0,98	C	T	0.016 (0.003)	0.004 (0.02)	-0.005 (0.03)	0.007 (0.032)	0.039 (0.084)	0.016 (0.056)	Fasting glucose
rs11708067	3	123065778	0,98	A	G	0.03 (0.003)	-0.047 (0.022)	-0.053 (0.034)	-0.032 (0.036)	-0.009 (0.096)	-0.086 (0.061)	Fasting glucose
rs1280	3	170713290	1,00	T	C	0.035 (0.004)	-0.014 (0.026)	0.016 (0.039)	-0.051 (0.043)	0.13 (0.115)	-0.079 (0.078)	Fasting glucose
rs7651090	3	185513392	1,00	G	A	0.017 (0.003)	-0.012 (0.019)	0.013 (0.029)	-0.031 (0.032)	0 (0.082)	-0.055 (0.055)	Fasting glucose
rs7708285	5	76425867	0,99	G	A	0.015 (0.003)	0.026 (0.02)	-0.017 (0.03)	0.05 (0.033)	0.148 (0.085)	0.048 (0.057)	Fasting glucose
rs4869272	5	95539448	1,00	T	C	0.023 (0.003)	-0.001 (0.019)	-0.023 (0.029)	0.009 (0.032)	0.091 (0.082)	0.007 (0.055)	Fasting glucose
rs17762454	6	7213200	1,00	T	C	0.016 (0.003)	0.005 (0.021)	0.014 (0.031)	-0.011 (0.034)	0.085 (0.089)	-0.014 (0.058)	Fasting glucose
rs9368222	6	20686996	1,00	A	C	0.019 (0.003)	-0.016 (0.02)	-0.036 (0.03)	0.006 (0.034)	0.101 (0.085)	-0.062 (0.058)	Fasting glucose
rs2191349	7	15064309	1,00	T	G	0.038 (0.003)	-0.016 (0.018)	-0.047 (0.027)	0 (0.03)	0.022 (0.075)	0.034 (0.052)	Fasting glucose
rs2908289	7	44223942	0,99	A	G	0.076 (0.004)	0.018 (0.025)	-0.024 (0.038)	0.042 (0.04)	0.01 (0.101)	0.077 (0.067)	Fasting glucose
rs6943153	7	50791579	1,00	T	C	0.02 (0.003)	-0.045 (0.02)	-0.024 (0.029)	-0.064 (0.032)	0.088 (0.082)	-0.119 (0.054)	Fasting glucose
rs983309	8	9177732	1,00	T	G	0.034 (0.004)	-0.02 (0.029)	-0.012 (0.044)	0.001 (0.047)	-0.219 (0.121)	-0.025 (0.083)	Fasting glucose
rs11558471	8	118185733	0,99	A	G	0.038 (0.003)	-0.006 (0.02)	-0.018 (0.029)	0.023 (0.032)	-0.071 (0.082)	-0.018 (0.055)	Fasting glucose
rs10814916	9	4293150	0,99	C	A	0.021 (0.003)	0.022 (0.018)	0.068 (0.027)	-0.03 (0.03)	-0.037 (0.078)	0.028 (0.052)	Fasting glucose
rs10811661	9	22134094	0,90	T	C	0.031 (0.004)	-0.002 (0.025)	-0.035 (0.037)	0.025 (0.043)	0.017 (0.102)	0.036 (0.076)	Fasting glucose
rs16913693	9	111680359	0,99	T	G	0.057 (0.009)	-0.093 (0.057)	-0.077 (0.088)	-0.017 (0.092)	-0.139 (0.228)	-0.356 (0.161)	Fasting glucose

rs3829109	9	139256766	0,90	G	A	0.023 (0.004)	-0.069 (0.021)	-0.066 (0.033)	-0.064 (0.034)	-0.015 (0.088)	-0.113 (0.058)	Fasting glucose
rs11195502	10	113039667	0,99	C	T	0.043 (0.005)	0.038 (0.032)	0.039 (0.05)	0.04 (0.053)	-0.035 (0.135)	0.056 (0.086)	Fasting glucose
rs7901695	10	114754088	1,00	C	T	0.026 (0.003)	-0.042 (0.02)	-0.062 (0.03)	-0.044 (0.032)	0.107 (0.086)	-0.032 (0.055)	Fasting glucose
rs11605924	11	45873091	1,00	A	C	0.027 (0.003)	-0.03 (0.018)	-0.063 (0.027)	0.001 (0.03)	0.076 (0.078)	-0.048 (0.051)	Fasting glucose
rs11039182	11	47346723	0,98	T	C	0.031 (0.003)	-0.024 (0.02)	-0.008 (0.03)	-0.03 (0.033)	-0.05 (0.086)	-0.056 (0.058)	Fasting glucose
rs174576	11	61603510	0,99	C	A	0.026 (0.003)	0.03 (0.019)	0.022 (0.029)	0.034 (0.031)	0.067 (0.078)	0.03 (0.054)	Fasting glucose
rs11603334	11	72432985	1,00	G	A	0.025 (0.004)	0.043 (0.025)	0.014 (0.037)	0.073 (0.041)	-0.025 (0.105)	0.087 (0.069)	Fasting glucose
rs11020124	11	92690661	0,99	C	T	0.082 (0.003)	0.034 (0.02)	-0.006 (0.029)	0.087 (0.033)	0.12 (0.085)	-0.018 (0.057)	Fasting glucose
rs2657879	12	56865338	0,93	G	A	0.016 (0.004)	-0.012 (0.024)	-0.041 (0.036)	0.026 (0.038)	-0.017 (0.099)	-0.029 (0.067)	Fasting glucose
rs10747083	12	133041618	0,95	A	G	0.018 (0.003)	0.03 (0.02)	0.017 (0.03)	0.026 (0.033)	0.025 (0.083)	0.087 (0.056)	Fasting glucose
rs11619319	13	28487599	1,00	G	A	0.026 (0.003)	-0.004 (0.021)	-0.037 (0.032)	0.022 (0.035)	0.04 (0.091)	0.02 (0.061)	Fasting glucose
rs576674	13	33554302	0,98	G	A	0.022 (0.004)	-0.039 (0.024)	-0.09 (0.035)	-0.017 (0.041)	0.223 (0.102)	-0.021 (0.07)	Fasting glucose
rs3783347	14	100839261	0,97	G	T	0.022 (0.003)	-0.003 (0.022)	-0.007 (0.034)	-0.035 (0.037)	-0.029 (0.093)	0.105 (0.06)	Fasting glucose
rs4502156	15	62383155	0,98	T	C	0.03 (0.003)	0 (0.018)	-0.009 (0.028)	0.008 (0.03)	-0.032 (0.08)	0.024 (0.052)	Fasting glucose
rs12440695	15	62435156	1,00	T	C	0.014 (0.003)	0.014 (0.019)	-0.006 (0.028)	0.018 (0.031)	0.017 (0.082)	0.071 (0.052)	Fasting glucose
rs2302593	19	46196634	0,96	C	G	0.019 (0.003)	0.007 (0.018)	-0.033 (0.027)	0.056 (0.03)	0.076 (0.078)	-0.025 (0.051)	Fasting glucose
rs6113722	20	22557099	0,94	G	A	0.046 (0.007)	-0.01 (0.044)	0.09 (0.061)	-0.053 (0.073)	-0.15 (0.19)	-0.292 (0.14)	Fasting glucose
rs6072275	20	39743905	1,00	A	G	0.021 (0.004)	-0.012 (0.025)	0.007 (0.037)	-0.008 (0.041)	-0.123 (0.106)	-0.041 (0.072)	Fasting glucose
rs2820436	1	219640680	0,99	C	A	0.028 (0.005)	0.005 (0.02)	-0.021 (0.029)	0.035 (0.032)	0.071 (0.082)	-0.024 (0.054)	Fasting insulin
rs1530559	2	135755629	0,80	A	G	0.026 (0.005)	0.032 (0.021)	0.044 (0.032)	0.016 (0.034)	0.095 (0.088)	0.008 (0.059)	Fasting insulin
rs10195252	2	165513091	0,99	T	C	0.029 (0.005)	0.04 (0.018)	0.011 (0.027)	0.05 (0.03)	0.167 (0.079)	0.06 (0.053)	Fasting insulin
rs2972143	2	227116365	1,00	G	A	0.026 (0.005)	0.036 (0.019)	0.016 (0.028)	0.056 (0.031)	0.104 (0.082)	0.02 (0.053)	Fasting insulin
rs17036328	3	12390484	1,00	T	C	0.028 (0.007)	0.031 (0.026)	0.033 (0.039)	0.011 (0.044)	0.035 (0.119)	0.085 (0.079)	Fasting insulin
rs3822072	4	89741269	0,98	A	G	0.017 (0.004)	0.011 (0.018)	0.018 (0.027)	-0.029 (0.03)	0.051 (0.078)	0.088 (0.052)	Fasting insulin
rs9884482	4	106081636	0,99	C	T	0.03 (0.004)	0.053 (0.018)	0.049 (0.027)	0.069 (0.03)	-0.014 (0.08)	0.048 (0.053)	Fasting insulin
rs6822892	4	157734675	0,99	A	G	0.019 (0.005)	0.003 (0.019)	0.019 (0.029)	-0.031 (0.032)	0.077 (0.083)	0.013 (0.055)	Fasting insulin
rs4865796	5	53272664	0,99	A	G	0.027 (0.005)	0.007 (0.02)	0.013 (0.029)	-0.008 (0.032)	-0.007 (0.084)	0.035 (0.056)	Fasting insulin
rs459193	5	55806751	0,98	G	A	0.026 (0.005)	-0.02 (0.021)	-0.038 (0.031)	-0.036 (0.034)	0.096 (0.089)	0.048 (0.06)	Fasting insulin
rs6912327	6	34764922	0,99	T	C	0.029 (0.006)	0.016 (0.022)	0.02 (0.033)	-0.003 (0.036)	0.117 (0.096)	0.009 (0.062)	Fasting insulin
rs2745353	6	127452935	0,99	T	C	0.026 (0.004)	-0.023 (0.018)	-0.026 (0.027)	-0.03 (0.03)	-0.048 (0.077)	0.02 (0.051)	Fasting insulin
rs1167800	7	75176196	0,99	A	G	0.028 (0.005)	0.001 (0.018)	-0.011 (0.027)	-0.015 (0.03)	0.031 (0.077)	0.081 (0.052)	Fasting insulin
rs983309	8	9177732	1,00	T	G	0.052 (0.007)	-0.02 (0.029)	-0.012 (0.044)	0.001 (0.047)	-0.219 (0.121)	-0.025 (0.083)	Fasting insulin
rs7903146	10	114758349	1,00	C	T	0.033 (0.005)	0.046 (0.02)	0.078 (0.03)	0.041 (0.033)	-0.124 (0.087)	0.021 (0.057)	Fasting insulin
rs1421085	16	53800954	0,99	C	T	0.036 (0.005)	0.035 (0.018)	0.021 (0.028)	0.079 (0.03)	-0.032 (0.077)	-0.012 (0.052)	Fasting insulin
rs731839	19	33899065	0,99	G	A	0.026 (0.005)	0.006 (0.019)	0.017 (0.029)	0.027 (0.031)	-0.037 (0.083)	-0.073 (0.054)	Fasting insulin

CHR: Chromosome. BP: Base position. EffAl: Effect allele. OthAl:Other allele. GE: Genotype-to-Exposure. GD: Genotype-to-Disease. β: Beta estimate. SE: Standard error. BMI: Body mass index. SBP: Systolic blood pressure. DBP: diastolic blood pressure. PP: Pulse [illegible]

[a]Beta-cell dysfunction SNPs within Type 2 diabetes.

[b]Insulin resistance SNPs within Type 2 diabetes.
