# Supplementary material for: The influence of obesity-related factors in the etiology of renal cell carcinoma—A mendelian randomization study
Source: PLoS Med. 2019 Jan 3;16(1):e1002724. doi: 10.1371/journal.pmed.1002724 (PMC6317776; doi:10.1371/journal.pmed.1002724)
Supplement: S2 Table — GWAS, genome-wide association study; RCC, renal cell carcinoma. (PDF) [file pmed.1002724.s002.pdf]

S2 Table- Description of the studies participating in the RCC GWAS meta-analysis.

Study Name (Abbreviation)	Design; location	Study Population	Cases (n)	Controls (n)	Genotyping Array
**IARC scan**			**5219**	**8010**	
The European Prospective Investigation into Cancer and Nutrition (EPIC)	Prospective cohort, 10 European countries	Adults aged 35-74 enrolled between 1992 and 2000 (n=521,468), Incident cancers identified through health insurance records, cancer and pathology registries and active follow-up (subjects and next of skin); Genomic DNA extracted from buffy coat	402	551	Omni5/610Q /550K
CeRePP	Case series; France	Pathologically confirmed RCC recruited through the CeRePP network; Controls recruited from a systematic urologic screening program, which includes men or women who had no history or symptoms of kidney disease with a normal morphologic status of kidneys at ultrasonographic examination. Genomic DNA extracted from blood and saliva samples.	460	568	Omni5/610Q /317K
Umea	Prospective cohort, Sweden	Population-based cohort, recruting since 1985; resident from the Västerbotten county in northen Sweden. Incident cases identified throuhg linkage to national death and cancer registries. Genomic DNA estracted from blood	297	301	Omni5
Karolinska	2 prospectiv cohorts; Sweden	COSM: Cohort of Swedish Men (45,306 men enrolled in 1997) and SMC: Swedish Mammography Cohort (61,433 women enrolled between 1987 and 1990), in central Sweden. Incident cases identified thorugh linkage to national death anc cancer registries. Genomic DNA extracted from saliva.	131	295	Omni5

Consortium For the Investication of Renal Malignancies / Melbourne Collaborative Cohort Study (ConFIRM/MCCS)	Case-control and cohort studies; Australia	Incident cases diagnosed in Victoria and Queensland in the period 2011-2014 and aged 18-74 years (ConFIRM), and from within the MCCS when fresh blood collected; Controls selected from healthy participants in the MCCS cohort; Genomic DNA extracted from whole blood (ConFIRM) and buffy coat (MCCS).	184	396	Omni5
The IARC K2 study	Case-control study; 4 cental/eastern Europe countries	Mutlicentric study conducted in 2007-2013 in Russia, Czech Republic, Romania, and Serbia. Hospital or population-based controls depending on recruting centers.	1640	1388	Omni5/Omni Express
The Nord-Trondelag Health (HUNT2) and Tromso Studies (Tromso IV)	2 prospective cohorts; Norway	HUNT2: Adults aged 20+ enrolled between 1995 and 1997 (N=65,285); Tromso IV:Adults aged 25+ enrolled between 1994-1995 (N=27,158); Cases identidied through Norwegian Cancer Registries; Genomic DNA extracted from buffy coat.	133	388	610Q/317K
The NCI/IARC study in central Europe (CE)	Case-control study; 4 European countries	Hostipatl based study conducted between 1999-2003 (except Poland) 2004-2007 (Poland). Controls matched to lung cancer and included in previous GWAS of lung cancer; Genomic DNA extracted from buffy coat and RBC.	1096	2058	317K
Arsenic Health Risk Assessment and Molecular Epidemiology study in Central Europe (ASHRAM)	Case-control study; Slovakia and Hungary	Cases recruited in 6 counties between 2002-2004; Contorls matched to lung cancer and included in previous GWAS of lung cancer: Genomic DNA extracted from whole blood.	84	399	610Q/317K
The Leeds cohort	Case series; UK	RCC from the Leeds cohort recruited between 1998-2007; Genomic DNA extracted from blood	348	-	610Q
SEARCH Kidney cancer study (SEARCH)	Case series; UK	Population based case series including all patients with a kidney cancer aged 70- and diagnosed between 2002-2006; Genomic DNA extracted from blood.	181	-	610Q

WTCCC	Controls; UK	Dataset generated by the Wellcome Trust Sanger Institute in collaboration with the 1958 Birth cohort; Genomic DNA extracted from blood	-	1361	550K
Moscow	Case-control study; Moscow	Incident cases were recruited between 2007-2009. Hospital based controls were match and recruited during same period; Genomic DNA extracted from blood	263	305	610Q
**NCI scan**			**3728**	**7816**	
Agricultural Health Study (AHS)	Prospective cohort; USA (Iowa, North Carolina)	Private and commercial pesticide applicators and spouses of private applicators, entrolled between 1993 and 1997 (n=89,655). Incident cancer identified through linkage with state cancer registries. Germline DNA extracted from buccal cell samples.	7	-	Omni Express
Alpha-Tocopherol, Beta-Carotene Cancer Prevention Study (ATBC)	Prospective cohort /randomized trial; Finland	Male smokers aged 50-69 at entry, enrolled between 1985 and 1988 (n=29,133). Incident cancers identified through linkaged to Finnish Cancer Registry. Germline DNA extracted from whole blood samples.	202	1509	Omni Express/ Omni2.5/ 660W/ 610K
BioVU	Hospital biorepository ; USA (Nashville)	Renal cancer patients treated at Venderbilt University Medical Center between 2006 and 2012. Controls selected from among patients admitted for non-cancer condition, frequency matched by age and sex.	629	207	Omni Express
American Cancer Society Cancer Prevention Study II Nutrition Cohort (CPS-II)	Prospective cohort; USA	Men and women aged 50-74 at entry, enrolled between 1992 and 1993 (n=184,194). Incident cancers identified from biannual follow-up questionnaires or linkage with state cancer registries. Germline DNA extracted from blood and buccal cell samples.	272	942	Omni Express/ Omni2.5/ 660W/ 610K
Dana-Farber/Harvard Cancer Center (DFHCC)	Case series: USA (Boston)	Patients with localized renal cancer trated at DFHCC between 2002 and 2010. Genomic DNA extracted from blood samples.	206	-	Omni Express

Health Professionals Follow-up Study (HPFS)	Prospective cohort; USA	Men in selected health professions, enrolled in 1986 (n=51,529). Incident cancers identified from follow-up questionnarires and subsequent medical record review. Germline DNA extracted from blood samples.	38	86	Omni Express
Nurses' Health Study (NHS)	Prospective cohort; USA	Female registered nurses aged 30-55, enrolled in 1976 (n=121,700). Incident cancers identified by mailed follow-up questionnarie and subsequent medical record review. Germline DNA extracted from blood samples.	57	434	Omni Express
Physicians Health Sudy (PHS)	Prospective cohort / randomized trial; USA	Male physicians aged 50+ enrolled between 1982 and 1984 (n=22,071). Incident cancers identified by mailed follow-up questionnaire. Germline DNA extracted from blood samples.	21	-	Omni Express
Prostate, Lung, Colorectal and Ovarian Cancer Screening Trial (PLCO)	Prospective cohort / cancer screening trial; USA	Men and women aged 55-74 at entry, enrolled between 1993 and 2001 (n=155,000). Incident cancers identified from annual questionnaires, with confirmation through medical records. Germline DNA extracted from blood and buccal cell samples.	377	3.848	Omni Express/ Omni2.5/ 660W/ 610K/ 550K
National Cancer Institue U.S. Kidney Cancer Study (USKC)	Case-control; USA (Chicago, Detroit)	Cases: 1227 men and women aged 20-79 at diagnoses, identified from Chicago hospital records and Metropolitan Detroit Cancer Surveillance System between 2003 and 2007. Controls: 1235 men and women recruited from the state Department of Motor Vehicle recores (ages 30-64) and Medicare beneficiary records (ages 65+), frequency matched to cases on age, race, sex, region. Genomic DNA extracted from blood and buccal cell sampels.	662	561	610K
Van Andel Research Institute (VARI)	Case series; USA	Renal cancer patients treated at Spectrum Health Hospital in Grand Rapids, MI, and in hospitals affiliated with the Cooperative Human Tissue Network (CHTN). Germline DNA extracted from adjacent normal kidney tissue.	920	-	Omni Express
Vitamins and Lifestyle Study (VITAL)	Prospective cohort; USA (Washington State)	Men and women aged 50-76, enrolled between 2000 and 2002. Incident cancers identified by linkage to the western Washington SEER cancer registry. Germline DNA extracted from buccal cell free sampels	91	-	Omni Express

Women's Health Initiative (WHI)	Prospective cohort/ randomized trial; USA	Women aged 50-79, enrolled between 1993 and 1998. Incident cancers were identified by mailed follow-up questionnaires, and verified by medical records. Genomic DNA extracted from buffy coat samples.	239	229	Omni Express
Women's Health Study (WHS)	Prospective cohort/ randomized trial; USA	Female health professionals aged 45 years or older, enrolled in 1993. Incident cancers identified by mailed follow-up questionnarie. Genomc DNA extracted from blood smaples.	7	-	Omni Express
**MD Anderson scan**	Case-control; USA (Texas)	Cases: 894 Caucasian men and women treated for renal cancer at the University of Texas MD Anderson Cancer Center. Controls: 556 Caucasian men and women identified through random-digit dialing. Genomc DNA extracted from blood samples.	**893**	**556**	660W
**UK scan**	Case series; UK	Cases:adult renal cancer patients identified from UK clinical oncology centers and the institute of Cancer Research and Royal Marsden NHS Hospitals Trust. Controls: previously genotyped samples from the Wellcome Trust Case Control Consortium 2 1958 birth cohort and UK Blood Sevice Control Group (WTCC controls included in the IARC scan excluded). Genomic DNA extracted from blood samples.	**944**	**4024**	Omni Express/ Hap 1.2M Duo
