# Supplementary material for: The influence of obesity-related factors in the etiology of renal cell carcinoma—A mendelian randomization study
Source: PLoS Med. 2019 Jan 3;16(1):e1002724. doi: 10.1371/journal.pmed.1002724 (PMC6317776; doi:10.1371/journal.pmed.1002724)
Supplement: S3 Table — LCI, lower confidence interval; n SNPs, number of SNPs; OR, odds ratio; P, P value; PSNP-Heterogeneity, heterogeneity P value between instrumental SNP causal estimates (βGD/βGE) from genetic effects in S1 Table; UCI, upper confidence interval. (PDF) [file pmed.1002724.s003.pdf]

S3 Table- Pleiotropy assessment and risk increase of obesity-related factors on RCC provided by sensitivity tests.

			MR-PRESSO approach			Weigthed median approach				MR-Egger test							
**Phenotype**	**n SNP**	**$P_{SNP-Heterogeneity}$**	**$P_{Horizontal\ pleiotropy}$**	**Outlier SNPs**	**$P_{Outlier\ bias}$**	**OR**	**LCI**	**UCI**	**P**	**Intercept**	**LCI**	**UCI**	**P**	**OR**	**LCI**	**UCI**	**P**
Body mass index	709	$9x10^{-6}$	$<1x10^{-4}$	-	-	1,75	1,50	2,03	$1x10^{-12}$	$-3x10^{-3}$	-0,01	$8x10^{-4}$	0,12	1,99	1,49	2,65	$3x10^{-6}$
Waist to hip ratio	355	$1x10^{-18}$	$<1x10^{-4}$	-	-	1,45	1,14	1,86	$3x10^{-3}$	$-1x10^{-3}$	-0,01	0,01	0,68	1,97	0,93	4,19	0,08
Body fat %	398	$1x10^{-5}$	$<1x10^{-4}$	-	-	1,63	1,31	2,03	$1x10^{-5}$	$3x10^{-3}$	$-3x10^{-3}$	0,01	0,33	1,41	0,75	2,66	0,29
Systolic blood pressure	199	$2x10^{-8}$	$<1x10^{-4}$	-	-	1,09	0,86	1,37	0,50	$5x10^{-3}$	-0,01	0,01	0,36	0,75	0,34	1,66	0,48
Diastolic blood pressure	233	$4x10^{-10}$	$<1x10^{-4}$	-	-	1,30	1,05	1,60	0,02	$2x10^{-3}$	-0,01	0,01	0,63	1,14	0,49	2,64	0,76
Pulse pressure	260	$2x10^{-9}$	$<1x10^{-4}$	-	-	0,81	0,66	1,00	0,05	$3x10^{-5}$	$-4x10^{-3}$	0,01	0,39	0,64	0,38	1,06	0,08
High density cholesterol	69	$1x10^{-4}$	$3x10^{-4}$	-	-	1,11	0,96	1,28	0,15	-0,01	-0,02	$-2x10^{-3}$	0,02	1,20	0,98	1,46	0,07
Low density cholesterol	52	$6x10^{-4}$	$6x10^{-4}$	-	-	0,87	0,74	1,02	0,09	$3x10^{-3}$	-0,01	0,01	0,49	0,94	0,76	1,16	0,54
Total cholesterol	70	$4x10^{-4}$	$6x10^{-4}$	-	-	0,93	0,80	1,09	0,39	0,01	$-3x10^{-3}$	0,02	0,19	0,91	0,72	1,15	0,43
Triglycerides	41	0,08	0,07	-	-	0,89	0,74	1,08	0,24	0,01	$-3x10^{-3}$	0,02	0,16	0,86	0,68	1,10	0,22
Type 2 Diabetes	39	0,01	0,01	-	-	0,94	0,86	1,03	0,20	0,01	-0,01	0,02	0,38	0,93	0,77	1,13	0,45
Fasting glucose	37	0,02	0,02	-	-	0,98	0,72	1,34	0,92	-0,01	-0,02	$2x10^{-3}$	0,10	1,23	0,75	1,99	0,40
Fasting insulin	17	0,17	0,16	-	-	1,79	1,08	2,97	0,02	$2x10^{-3}$	-0,05	0,05	0,92	2,14	0,13	36,58	0,58

n SNPs: Number of SNPs. $P_{SNP-Heterogeneity}$: Heterogeneity P value between instrumental SNP causal estimates (β_{GD}/β_{GE}) from genetic effects in Table S1. OR: Odds ratio. LCI: Lower confidence interval. UCI: Upper confidence interval. P: P value.
