# Supplementary material for: The influence of obesity-related factors in the etiology of renal cell carcinoma—A mendelian randomization study
Source: PLoS Med. 2019 Jan 3;16(1):e1002724. doi: 10.1371/journal.pmed.1002724 (PMC6317776; doi:10.1371/journal.pmed.1002724)

**Fig A - Statistical power to detect associations with risk for genetic instruments explaining different proportions of a risk factor in a Mendelian randomization analysis based on 10,784 cases and 20,406 controls.**

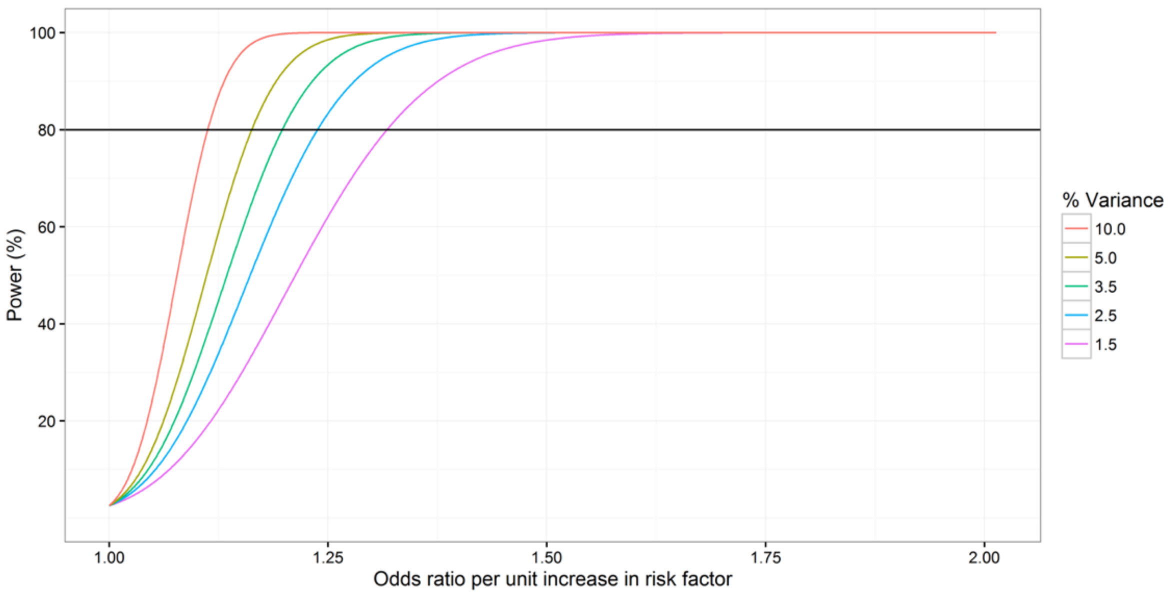

**Fig B - Forest plot of RCC risk for each SD increment in BMI stratified by study.** OR were estimated using likelihood-based MR approach. 95% CI: 95% Confidence Interval; P: P value. $I^2$: between-strata heterogeneity (%). Phet: P value of between-strata heterogeneity.

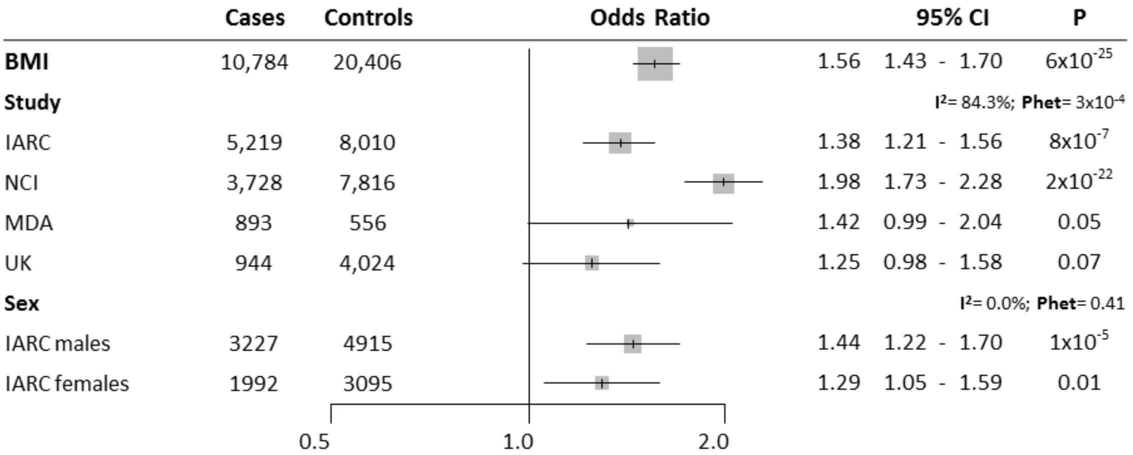

| | Cases | Controls | Odds Ratio | 95% CI | P |
|---|---|---|---|---|---|
| **BMI** | 10,784 | 20,406 | 1.56 | 1.43 - 1.70 | $6x10^{-25}$ |
| **Study** | | | | $I^2$= 84.3%; **Phet**= $3x10^{-4}$ | |
| IARC | 5,219 | 8,010 | 1.38 | 1.21 - 1.56 | $8x10^{-7}$ |
| NCI | 3,728 | 7,816 | 1.98 | 1.73 - 2.28 | $2x10^{-22}$ |
| MDA | 893 | 556 | 1.42 | 0.99 - 2.04 | 0.05 |
| UK | 944 | 4,024 | 1.25 | 0.98 - 1.58 | 0.07 |
| **Sex** | | | | $I^2$= 0.0%; **Phet**= 0.41 | |
| IARC males | 3227 | 4915 | 1.44 | 1.22 - 1.70 | $1x10^{-5}$ |
| IARC females | 1992 | 3095 | 1.29 | 1.05 - 1.59 | 0.01 |

**Fig C - Forest plot of RCC risk for each SD increment in waist-to-hip ratio stratified by study.** OR were estimated using likelihood-based MR approach. 95% CI: 95% Confidence Interval; P: P value. $I^2$: between-strata heterogeneity (%). Phet: P value of between-strata heterogeneity.

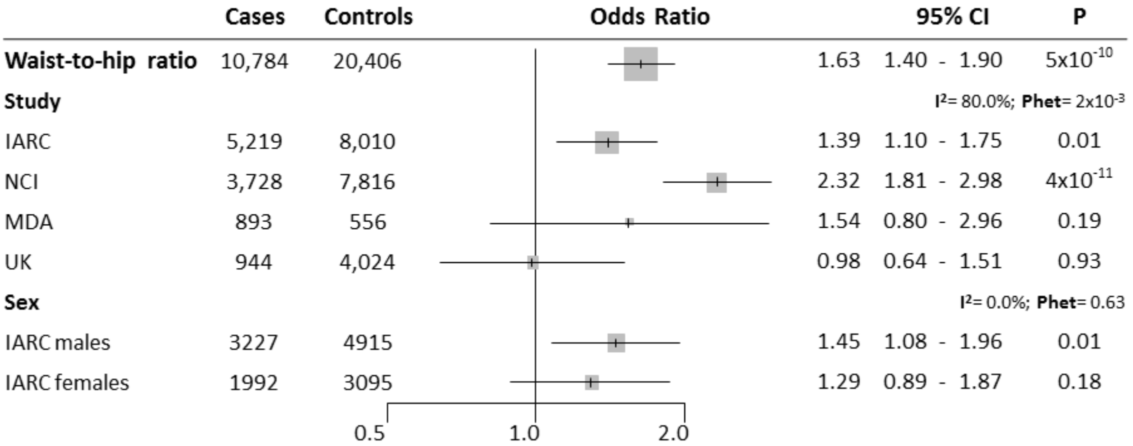

| | Cases | Controls | Odds Ratio | 95% CI | P |
|---|---|---|---|---|---|
| **Waist-to-hip ratio** | 10,784 | 20,406 | 1.63 | 1.40 - 1.90 | $5x10^{-10}$ |
| **Study** | | | | $I^2$= 80.0%; **Phet**= $2x10^{-3}$ | |
| IARC | 5,219 | 8,010 | 1.39 | 1.10 - 1.75 | 0.01 |
| NCI | 3,728 | 7,816 | 2.32 | 1.81 - 2.98 | $4x10^{-11}$ |
| MDA | 893 | 556 | 1.54 | 0.80 - 2.96 | 0.19 |
| UK | 944 | 4,024 | 0.98 | 0.64 - 1.51 | 0.93 |
| **Sex** | | | | $I^2$= 0.0%; **Phet**= 0.63 | |
| IARC males | 3227 | 4915 | 1.45 | 1.08 - 1.96 | 0.01 |
| IARC females | 1992 | 3095 | 1.29 | 0.89 - 1.87 | 0.18 |

**Fig D - Forest plot of RCC risk for each SD increment in body fat % stratified by study.** OR were estimated using likelihood-based MR approach. 95% CI: 95% Confidence Interval; P: P value. $I^2$: between-strata heterogeneity (%). Phet: P value of between-strata heterogeneity.

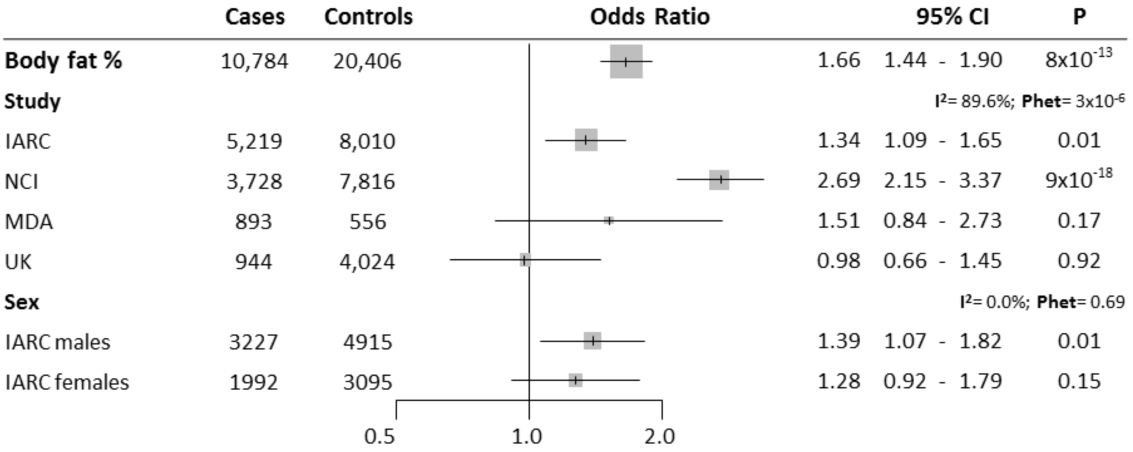

| | Cases | Controls | Odds Ratio | 95% CI | P |
|---|---|---|---|---|---|
| **Body fat %** | 10,784 | 20,406 | 1.66 | 1.44 - 1.90 | $8x10^{-13}$ |
| **Study** | | | | $I^2$= 89.6%; **Phet**= $3x10^{-6}$ | |
| IARC | 5,219 | 8,010 | 1.34 | 1.09 - 1.65 | 0.01 |
| NCI | 3,728 | 7,816 | 2.69 | 2.15 - 3.37 | $9x10^{-18}$ |
| MDA | 893 | 556 | 1.51 | 0.84 - 2.73 | 0.17 |
| UK | 944 | 4,024 | 0.98 | 0.66 - 1.45 | 0.92 |
| **Sex** | | | | $I^2$= 0.0%; **Phet**= 0.69 | |
| IARC males | 3227 | 4915 | 1.39 | 1.07 - 1.82 | 0.01 |
| IARC females | 1992 | 3095 | 1.28 | 0.92 - 1.79 | 0.15 |

**Fig E - The leave-one-out histogram reflects the distribution of the causal effect estimate of BMI instrument when one of the SNPs is removed.** OR: odds ratio.

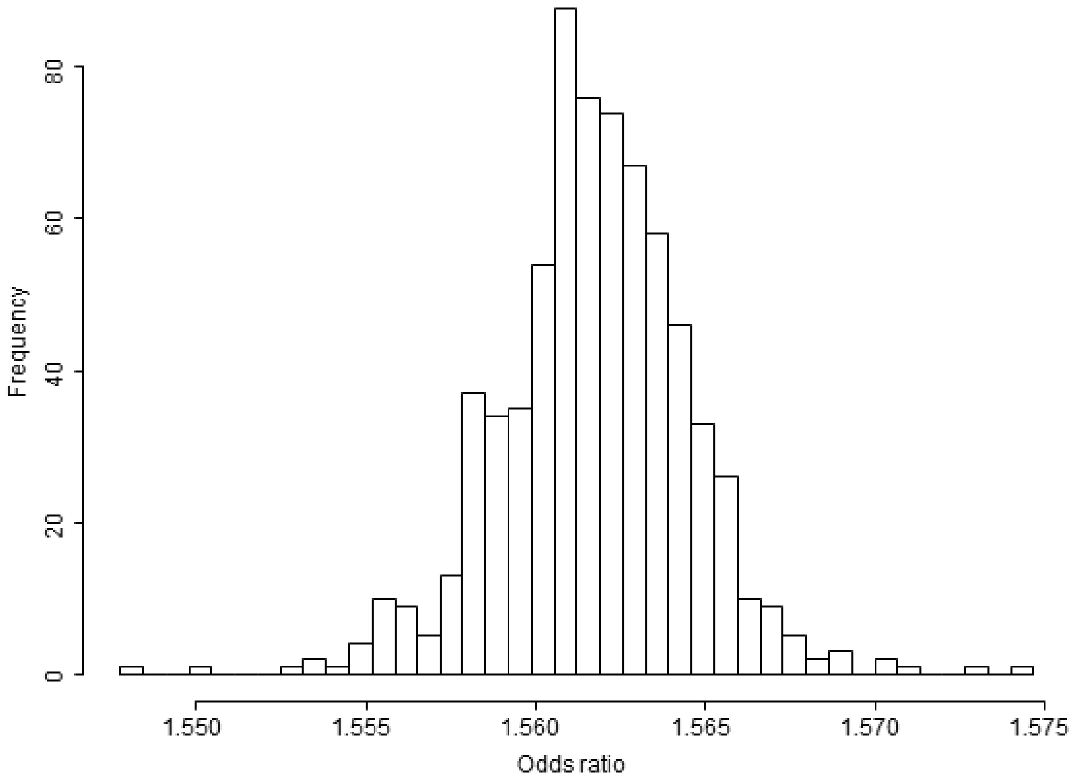


**Fig F - The leave-one-out histogram reflects the distribution of the causal effect estimate of WHR instrument when one of the SNPs is removed.** OR: odds ratio.

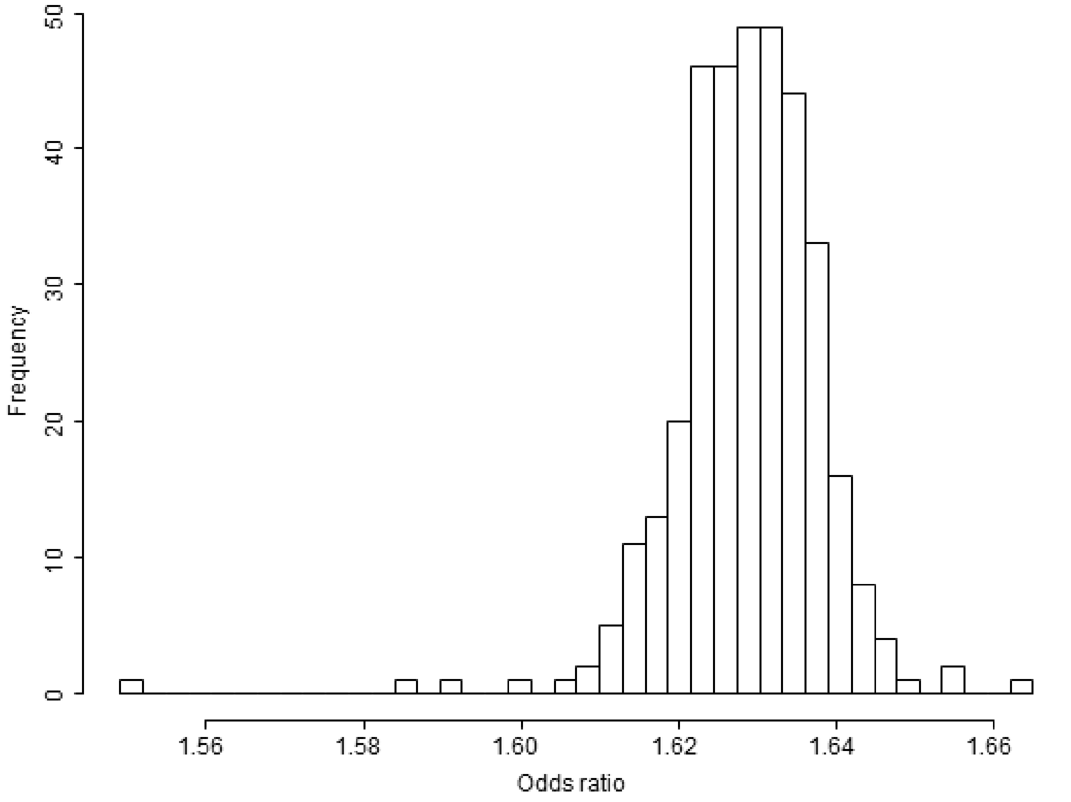

**Fig G - For each SNP used in the genetic instrument of body fat %, the funnel plot (A) depicts the weight exerted into the causal effect estimate along the y-axis ($\beta_{GP}/SE_{GD}$), and estimates of the causal effect on RCC odds along the x-axis ($exp(\beta_{GD}/\beta_{GP})$). The leave-one-out histogram (B) reflects the distribution of the causal effect estimate of body fat % instrument when one of the SNPs is removed.** OR: odds ratio. Funnel plot X-axis is in logarithmic scale.

A- Funnel plot

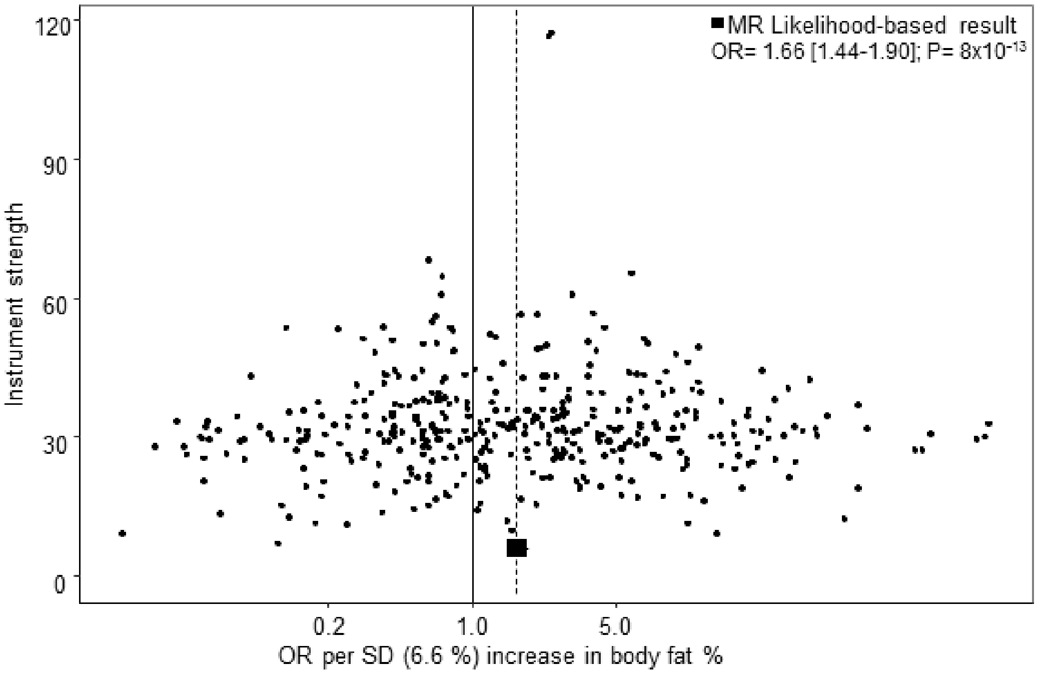


B- Leave-one-out histogram

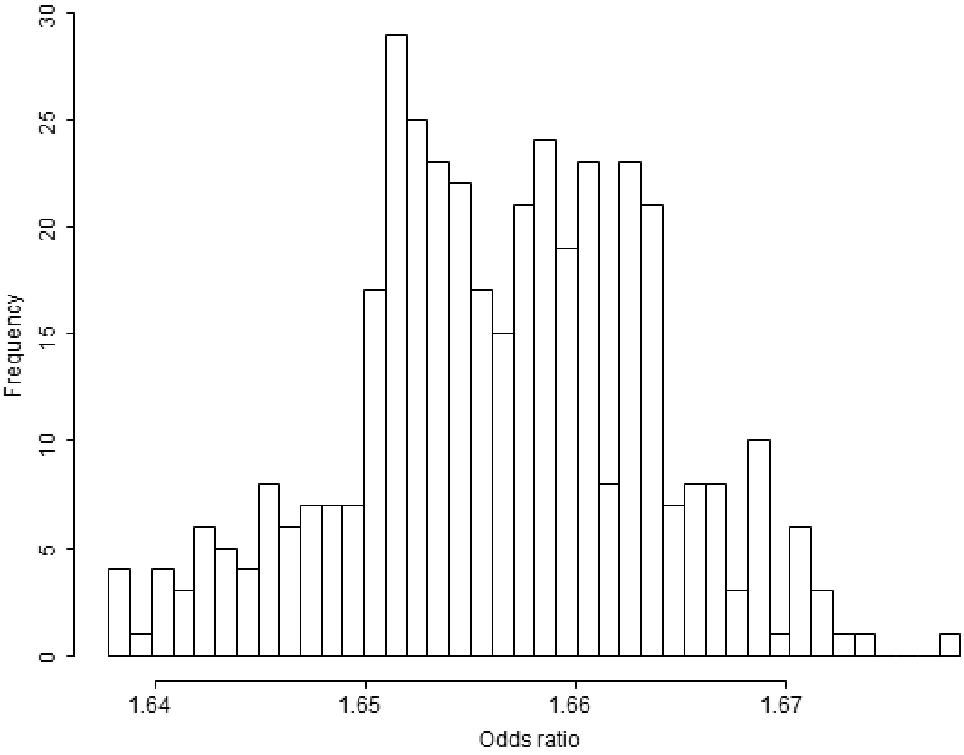

**Fig H - Forest plot of RCC risk for each SD increment in DBP stratified by study.** OR were estimated using likelihood-based MR approach. 95% CI: 95% Confidence Interval; P: P value. I2: between-strata heterogeneity (%). Phet: P value of between-strata heterogeneity.

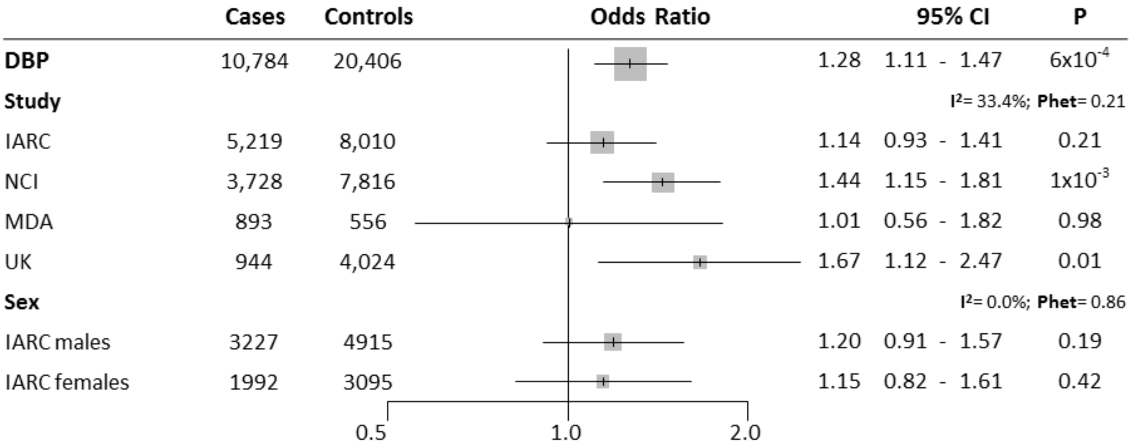


**Fig I - Forest plot of RCC risk for each SD increment in SBP stratified by study.** OR were estimated using likelihood-based MR approach. 95% CI: 95% Confidence Interval; P: P value. I2: between-strata heterogeneity (%). Phet: P value of between-strata heterogeneity.

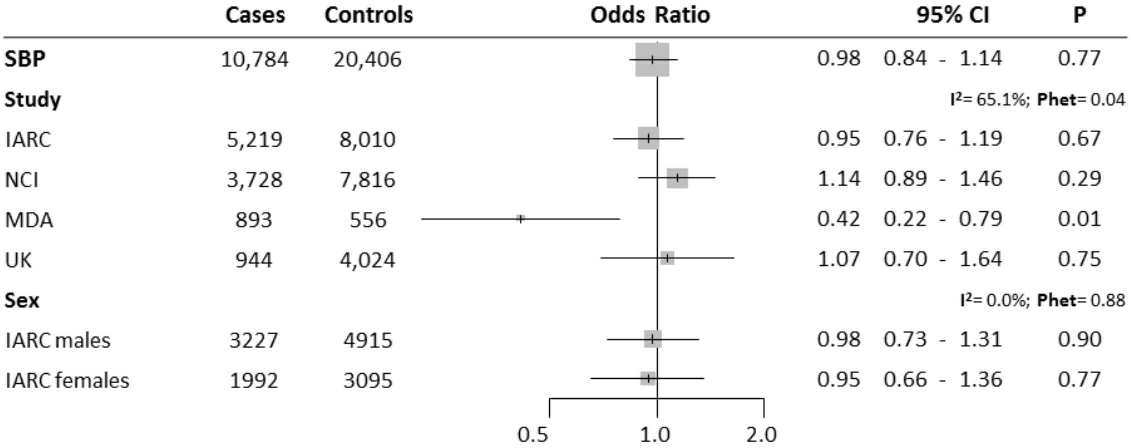


**Fig J - Forest plot of RCC risk for each SD increment in PP stratified by study.** OR were estimated using likelihood-based MR approach. 95% CI: 95% Confidence Interval; P: P value. I2: between-strata heterogeneity (%). Phet: P value of between-strata heterogeneity.

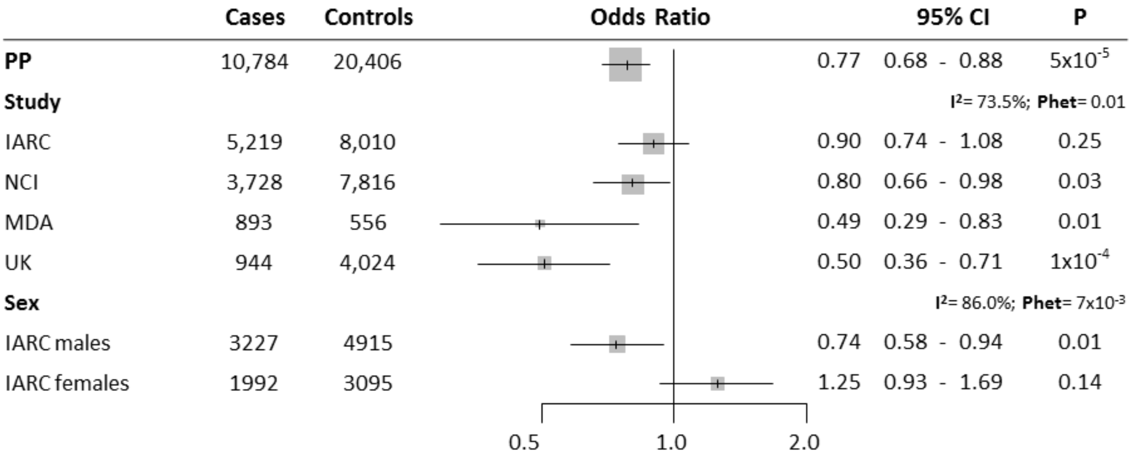

**Fig K - The leave-one-out histogram reflects the distribution of the causal effect estimate of DBP instrument when one of the SNPs is removed.** OR: odds ratio.

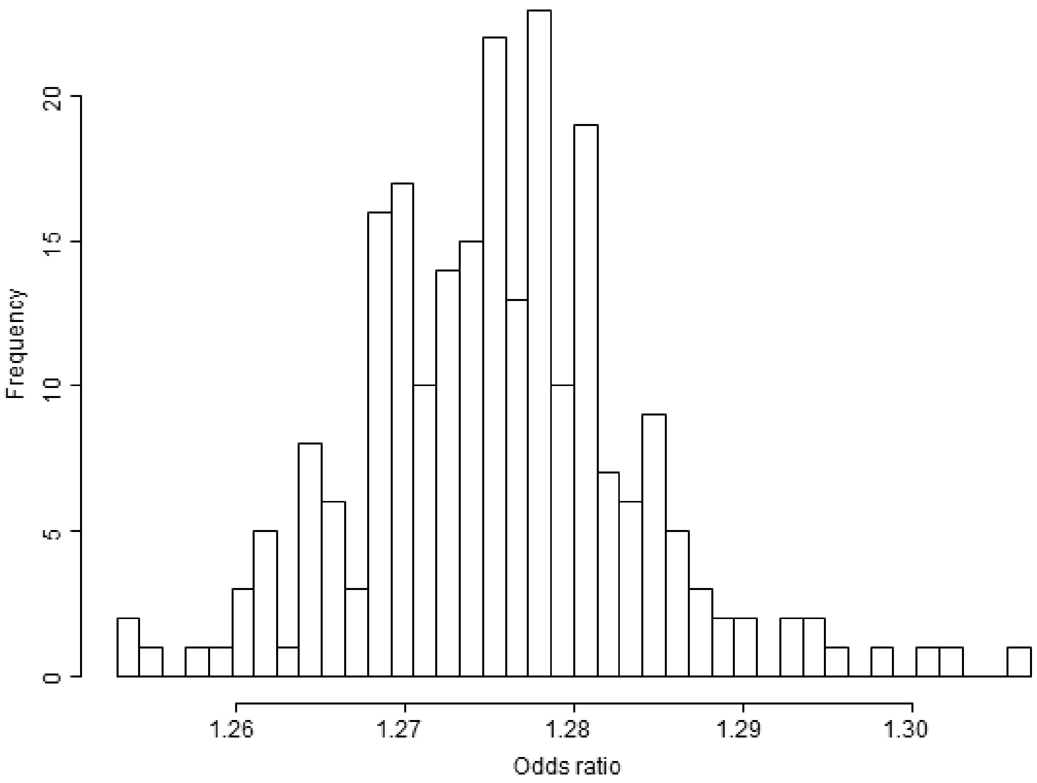

**Fig L - For each SNP used in the genetic instrument of SBP, the funnel plot (A) depicts the weight exerted into the causal effect estimate along the y-axis ($\beta_{GP}/SE_{GD}$), and estimates of the causal effect on RCC odds along the x-axis ($exp(\beta_{GD}/\beta_{GP})$). The leave-one-out histogram (B) reflects the distribution of the causal effect estimate of SBP instrument when one of the SNPs is removed.** OR: odds ratio. Funnel plot X-axis is in logarithmic scale.

A- Funnel plot

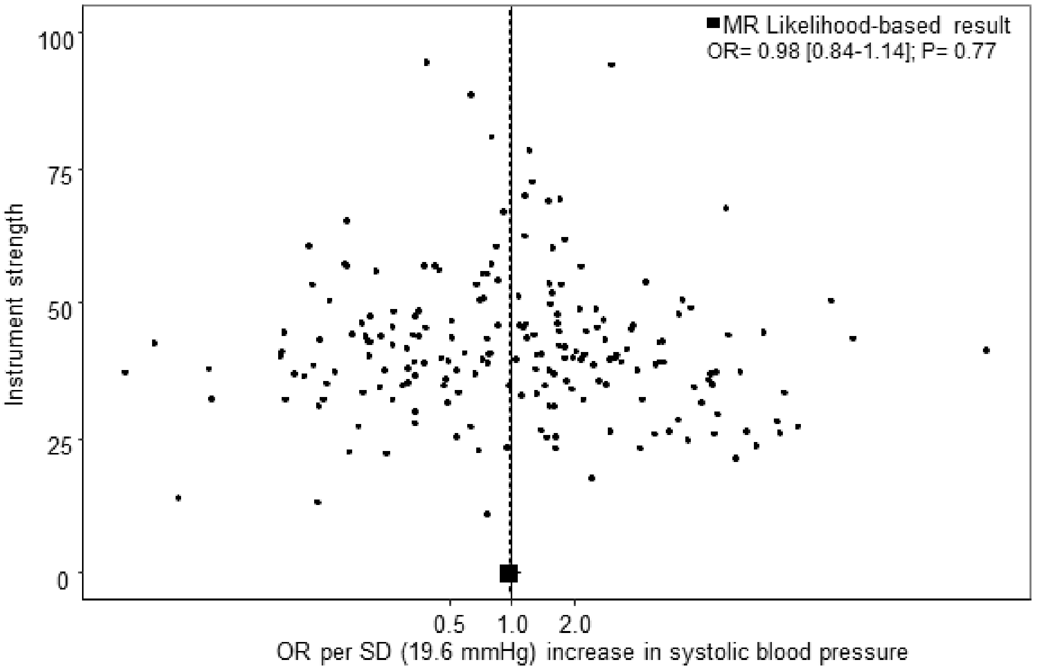


B- Leave-one-out histogram

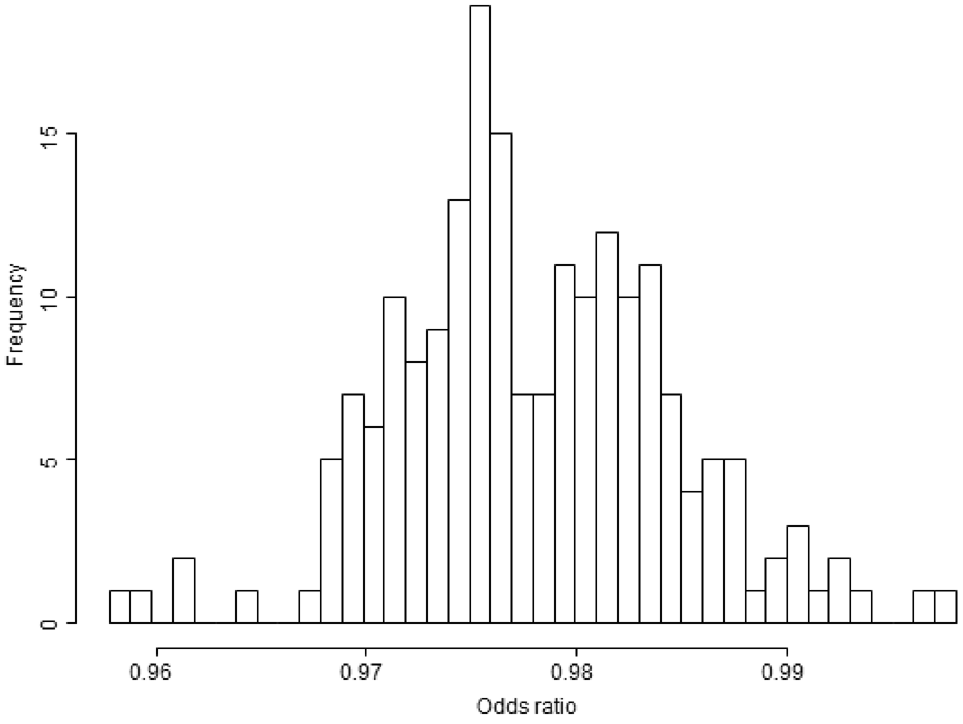

**Fig M - For each SNP used in the genetic instrument of PP, the funnel plot (A) depicts the weight exerted into the causal effect estimate along the y-axis ($\beta_{GP}/SE_{GD}$), and estimates of the causal effect on RCC odds along the x-axis ($exp(\beta_{GD}/\beta_{GP})$). The leave-one-out histogram (B) reflects the distribution of the causal effect estimate of PP instrument when one of the SNPs is removed.** OR: odds ratio. Funnel plot X-axis is in logarithmic scale.

A- Funnel plot

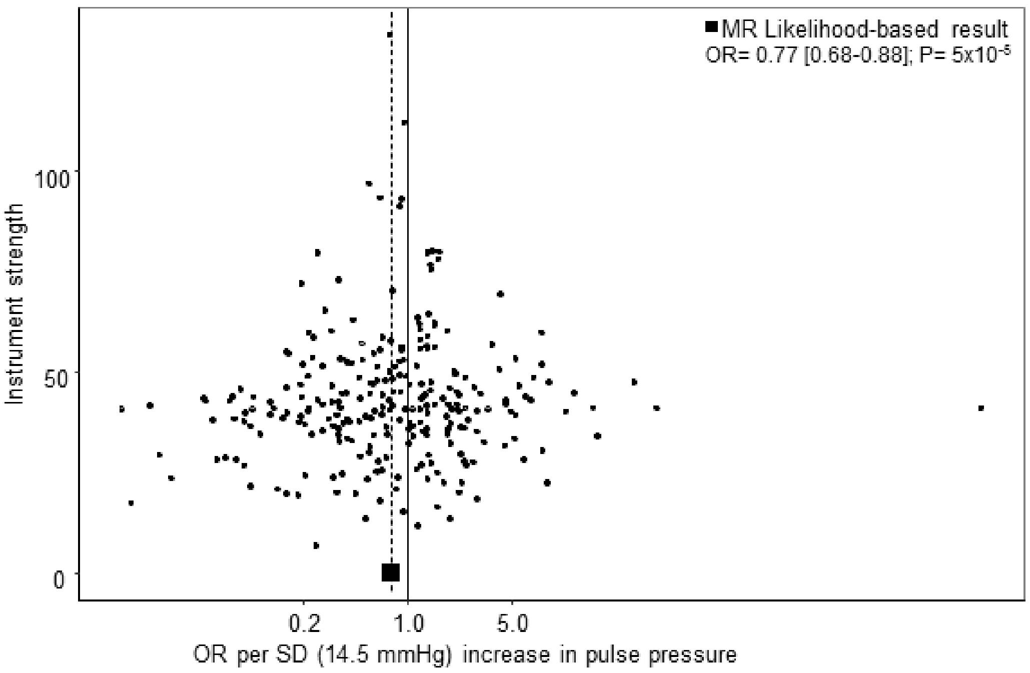


B- Leave-one-out histogram

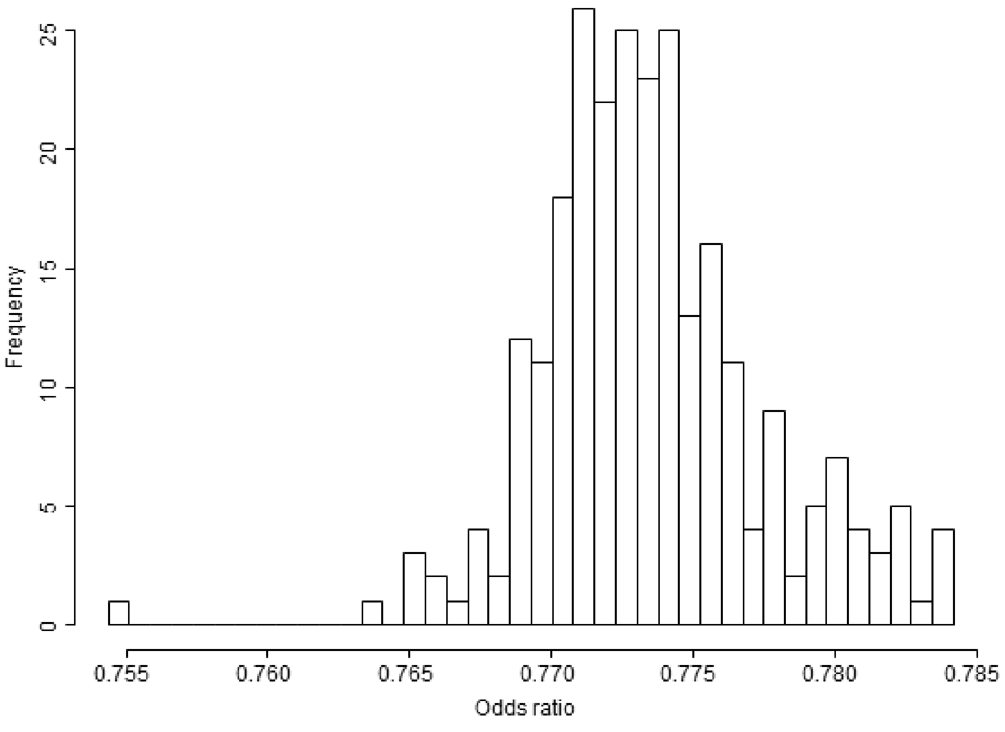

**Fig N - Forest plot of RCC risk for each SD increment in HDL stratified by study.** OR were estimated using likelihood-based MR approach. 95% CI: 95% Confidence Interval; P: P value. I2: between-strata heterogeneity (%). Phet: P value of between-strata heterogeneity**.**

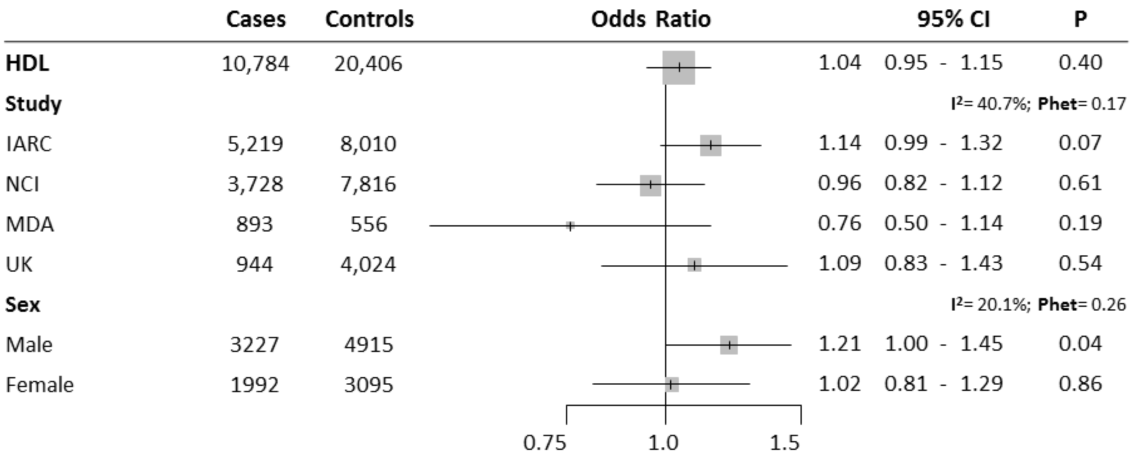


**Fig O - Forest plot of RCC risk for each SD increment in LDL stratified by study.** OR were estimated using likelihood-based MR approach. 95% CI: 95% Confidence Interval; P: P value. I2: between-strata heterogeneity (%). Phet: P value of between-strata heterogeneity.

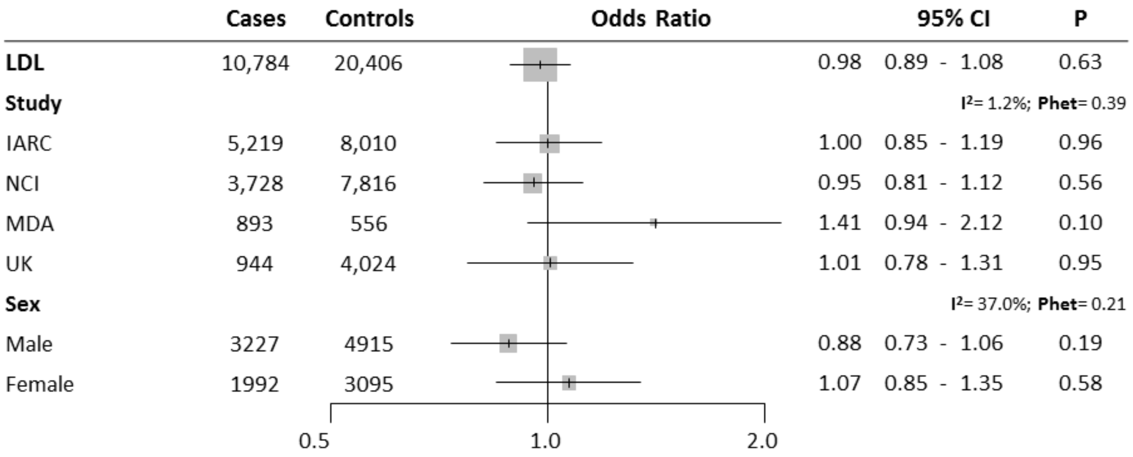

**Fig P - Forest plot of RCC risk for each SD increment in total cholesterol stratified by study.** OR were estimated using likelihood-based MR approach. 95% CI: 95% Confidence Interval; P: P value. I2: between-strata heterogeneity (%). Phet: P value of between-strata heterogeneity.

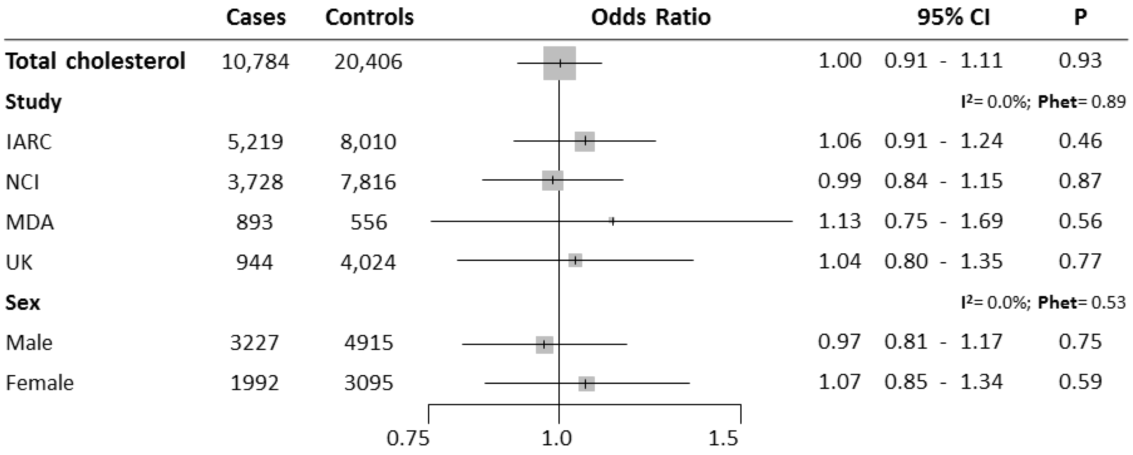


**Fig Q - Forest plot of RCC risk for each SD increment in triglycerides stratified by study.** OR were estimated using likelihood-based MR approach. 95% CI: 95% Confidence Interval; P: P value. I2: between-strata heterogeneity (%). Phet: P value of between-strata heterogeneity.

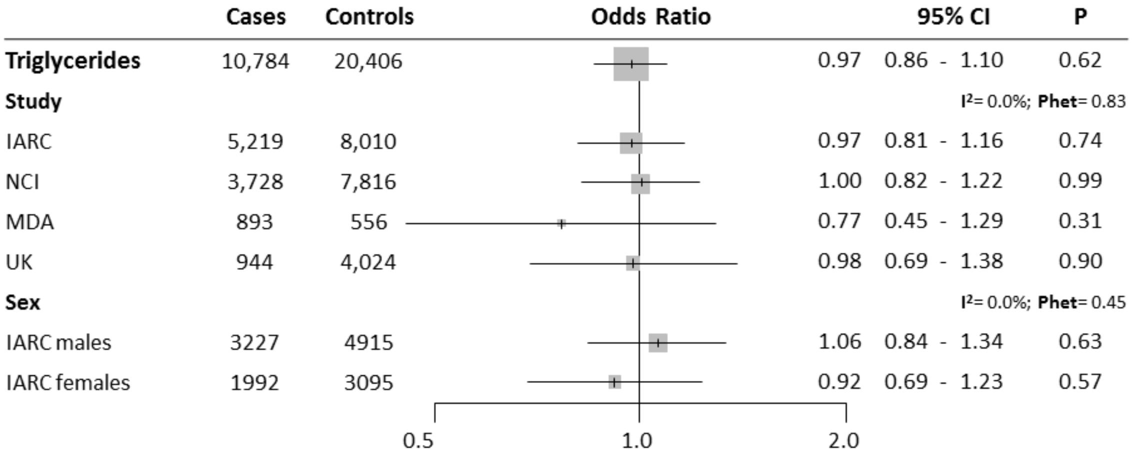

**Fig R - For each SNP used in the genetic instrument of HDL, the funnel plot (A) depicts the weight exerted into the causal effect estimate along the y-axis ($\beta_{GP}/SE_{GD}$), and estimates of the causal effect on RCC odds along the x-axis ($exp(\beta_{GD}/\beta_{GP})$). The leave-one-out histogram (B) reflects the distribution of the causal effect estimate of HDL instrument when one of the SNPs is removed.** OR: odds ratio. Funnel plot X-axis is in logarithmic scale.

A- Funnel plot

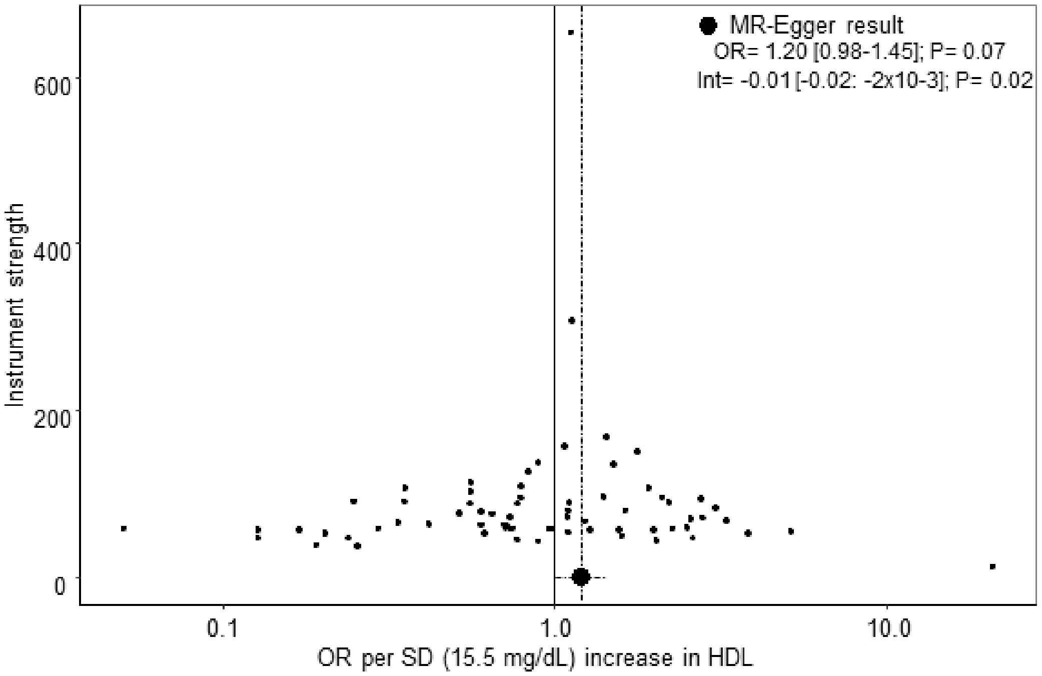


B- Leave-one-out histogram

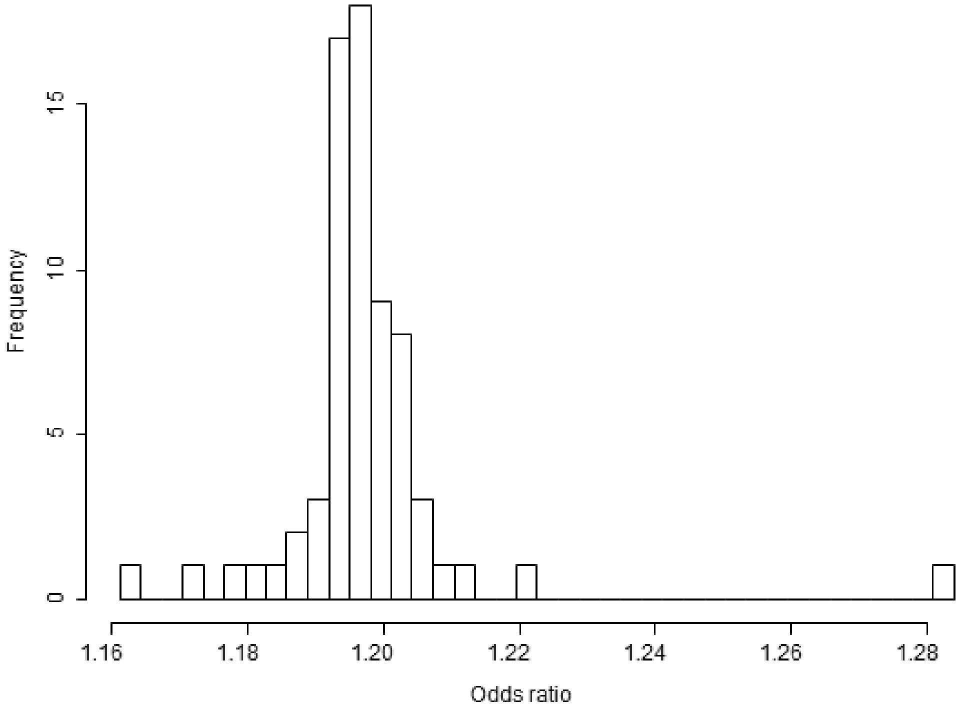

**Fig S - For each SNP used in the genetic instrument of LDL, the funnel plot (A) depicts the weight exerted into the causal effect estimate along the y-axis ($\beta_{GP}/SE_{GD}$), and estimates of the causal effect on RCC odds along the x-axis ($exp(\beta_{GD}/\beta_{GP})$). The leave-one-out histogram (B) reflects the distribution of the causal effect estimate of LDL instrument when one of the SNPs is removed.** OR: odds ratio. Funnel plot X-axis is in logarithmic scale.

A- Funnel plot

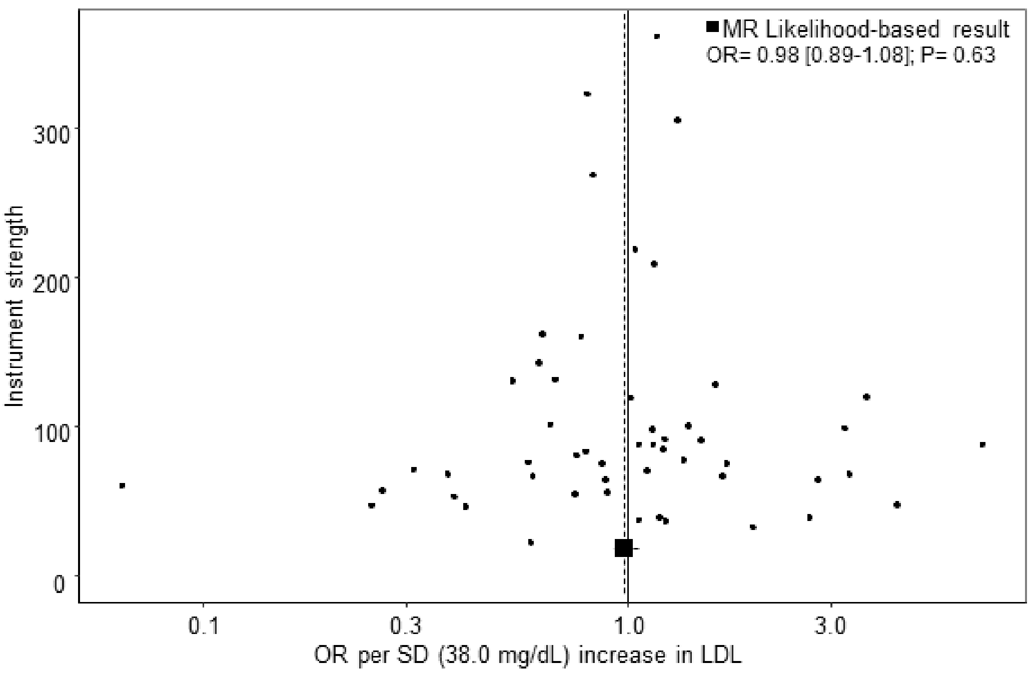


B- Leave-one-out histogram

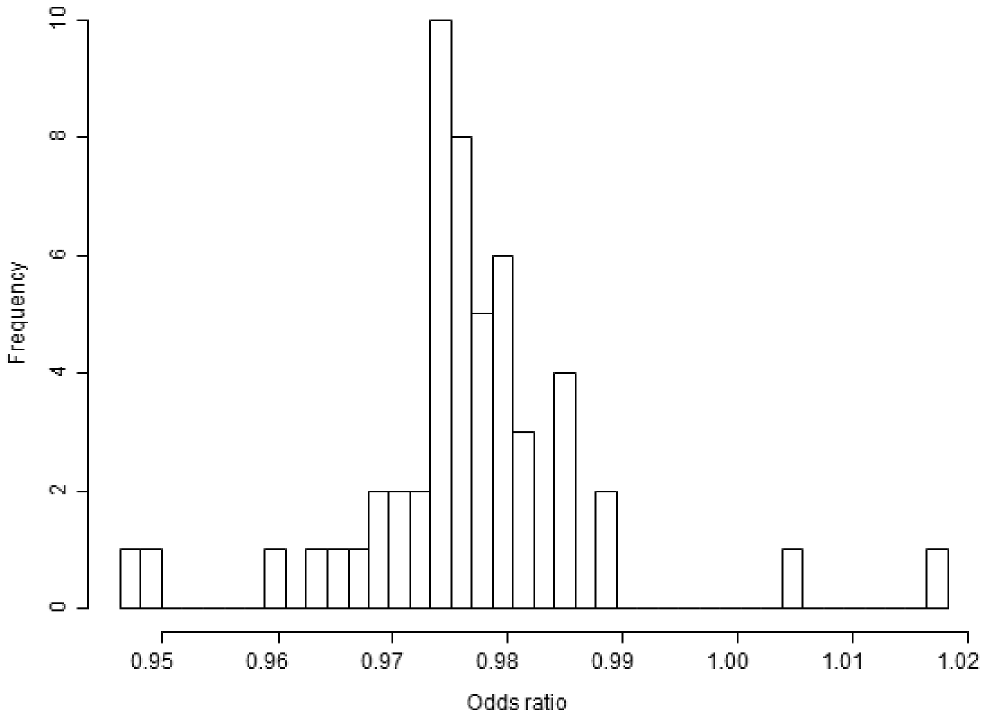

**Fig T - For each SNP used in the genetic instrument of total cholesterol, the funnel plot (A) depicts the weight exerted into the causal effect estimate along the y-axis ($\beta_{GP}/SE_{GD}$), and estimates of the causal effect on RCC odds along the x-axis ($\exp(\beta_{GD}/\beta_{GP})$). The leave-one-out histogram (B) reflects the distribution of the causal effect estimate of total cholesterol instrument when one of the SNPs is removed.** OR: odds ratio. Funnel plot X-axis is in logarithmic scale.

A- Funnel plot

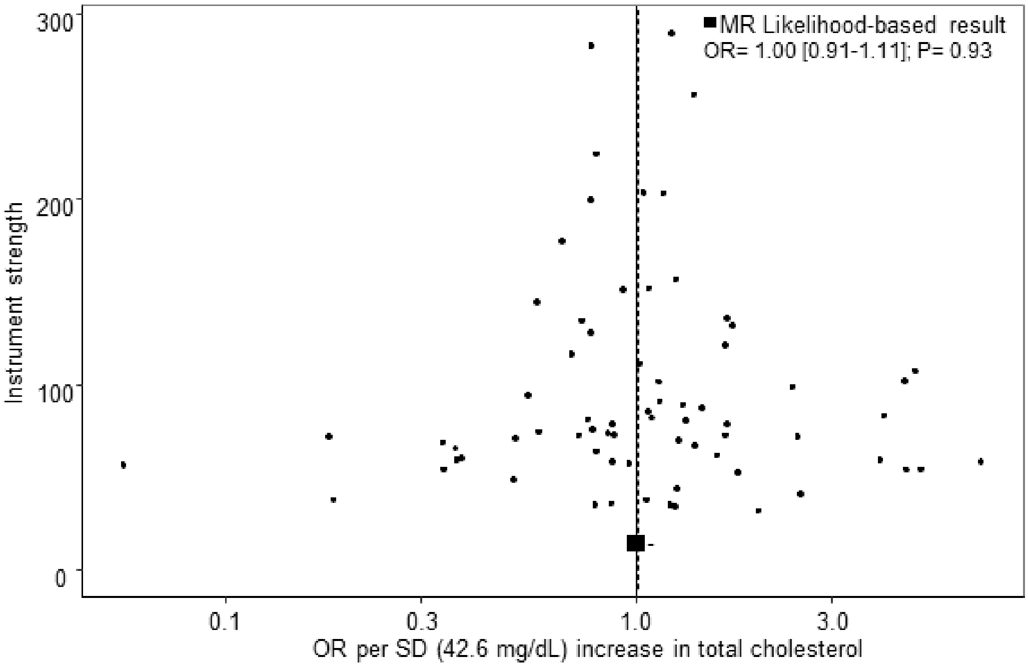


B- Leave-one-out histogram

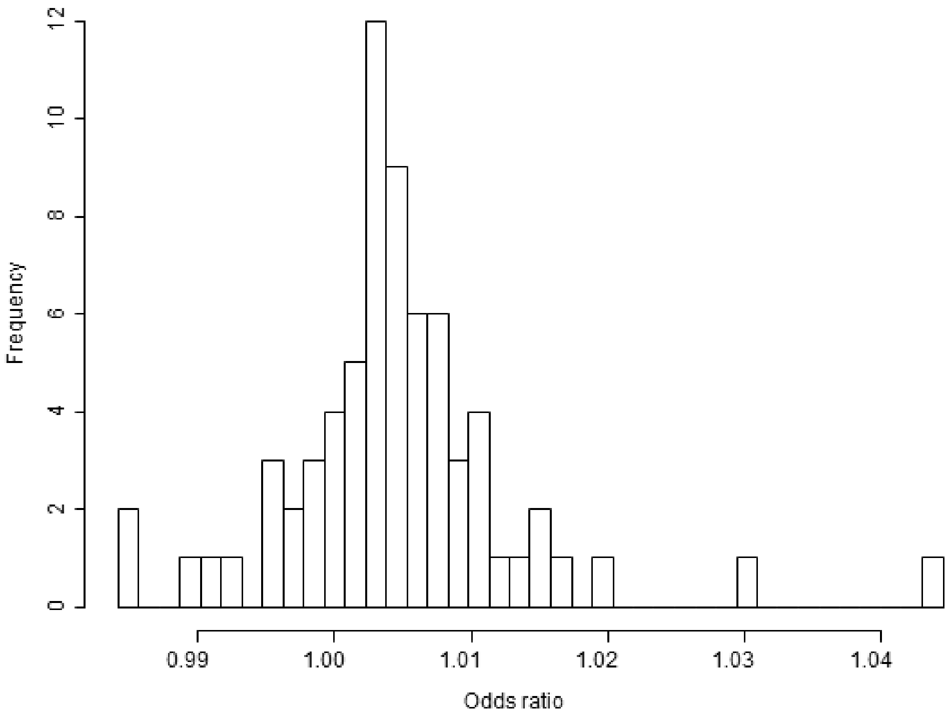

**Fig U - For each SNP used in the genetic instrument of triglycerides, the funnel plot (A) depicts the weight exerted into the causal effect estimate along the y-axis ($\beta_{GP}/SE_{GD}$), and estimates of the causal effect on RCC odds along the x-axis ($exp(\beta_{GD}/\beta_{GP})$). The leave-one-out histogram (B) reflects the distribution of the causal effect estimate of triglycerides instrument when one of the SNPs is removed.** OR: odds ratio. Funnel plot X-axis is in logarithmic scale.

A- Funnel plot

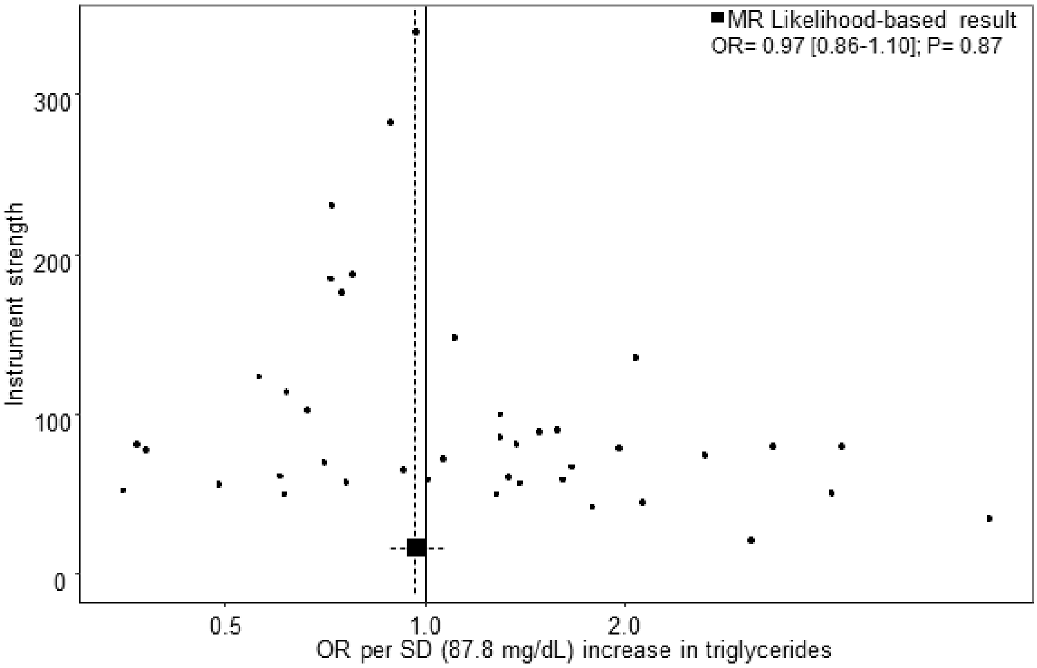


B- Leave-one-out histogram

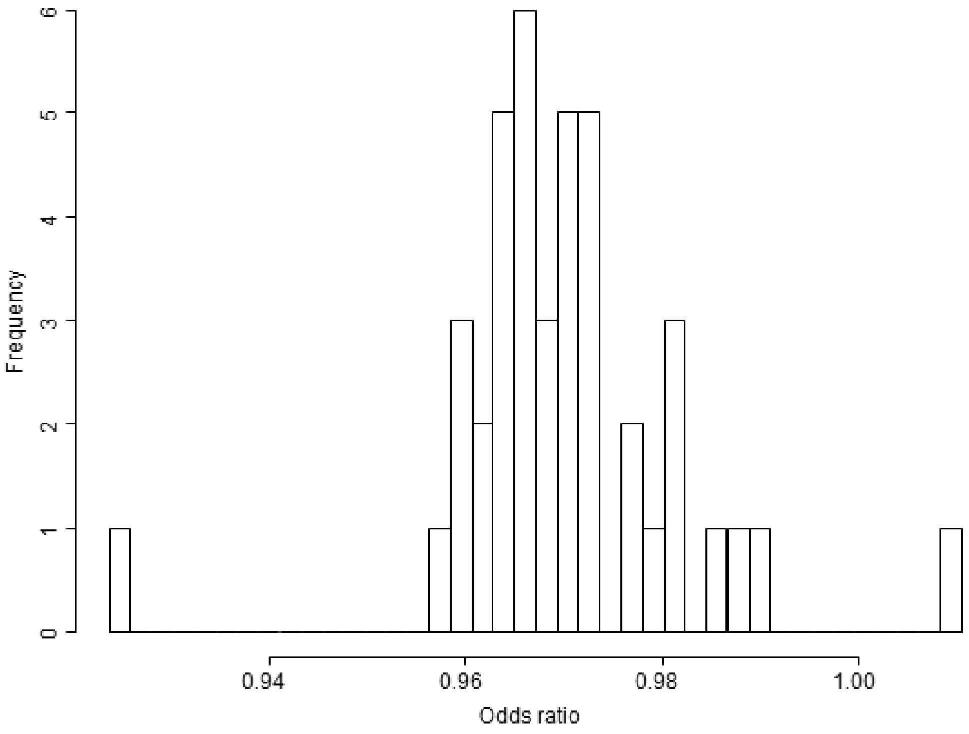

**Fig V - Forest plot of RCC risk for each SD increment in type 2 diabetes stratified by study.** OR were estimated using likelihood-based MR approach. 95% CI: 95% Confidence Interval; P: P value. I2: between-strata heterogeneity (%). Phet: P value of between-strata heterogeneity.

| | Cases | Controls | Odds Ratio | 95% CI | P |
|---|---|---|---|---|---|
| **Type 2 diabetes** | 10,784 | 20,406 | 0.99 | 0.93 - 1.05 | 0.74 |
| **Study** | | | | | I²= 28.1%; **Phet**= 0.24 |
| IARC | 5,219 | 8,010 | 0.92 | 0.84 - 1.01 | 0.09 |
| NCI | 3,728 | 7,816 | 1.04 | 0.94 - 1.16 | 0.40 |
| MDA | 893 | 556 | 1.11 | 0.85 - 1.44 | 0.44 |
| UK | 944 | 4,024 | 1.03 | 0.86 - 1.22 | 0.77 |
| **Sex** | | | | | I²= 0.0%; **Phet**= 0.53 |
| IARC males | 3227 | 4915 | 0.94 | 0.84 - 1.06 | 0.35 |
| IARC females | 1992 | 3095 | 0.89 | 0.77 - 1.03 | 0.12 |

**Fig W - Forest plot of RCC risk for each SD increment in fasting insulin stratified by study.** OR were estimated using likelihood-based MR approach. 95% CI: 95% Confidence Interval; P: P value. I2: between-strata heterogeneity (%). Phet: P value of between-strata heterogeneity.

| | Cases | Controls | Odds Ratio | 95% CI | P |
|---|---|---|---|---|---|
| **Fasting insulin** | 10,784 | 20,406 | 1.82 | 1.30 - 2.55 | $5x10^{-4}$ |
| **Study** | | | | | I²= 0.0%; **Phet**= 0.95 |
| IARC | 5,219 | 8,010 | 1.62 | 0.98 - 2.68 | 0.06 |
| NCI | 3,728 | 7,816 | 1.96 | 1.13 - 3.40 | 0.02 |
| MDA | 893 | 556 | 1.82 | 0.43 - 7.66 | 0.42 |
| UK | 944 | 4,024 | 2.12 | 0.82 - 5.50 | 0.12 |
| **Sex** | | | | | I²= 0.0%; **Phet**= 0.57 |
| IARC males | 3227 | 4915 | 1.43 | 0.75 - 2.72 | 0.28 |
| IARC females | 1992 | 3095 | 1.93 | 0.86 - 4.32 | 0.11 |

**Fig X - Forest plot of RCC risk for each SD increment in fasting glucose stratified by study.** OR were estimated using likelihood-based MR approach. 95% CI: 95% Confidence Interval; P: P value. I2: between-strata heterogeneity (%). Phet: P value of between-strata heterogeneity.

| | Cases | Controls | Odds Ratio | 95% CI | P |
|---|---|---|---|---|---|
| **Fasting glucose** | 10,784 | 20,406 | 0.92 | 0.76 - 1.12 | 0.43 |
| **Study** | | | | | I²= 64.8%; **Phet**= 0.04 |
| IARC | 5,219 | 8,010 | 0.75 | 0.56 - 1.01 | 0.05 |
| NCI | 3,728 | 7,816 | 1.23 | 0.89 - 1.70 | 0.21 |
| MDA | 893 | 556 | 1.71 | 0.73 - 3.96 | 0.21 |
| UK | 944 | 4,024 | 0.65 | 0.37 - 1.13 | 0.13 |
| **Sex** | | | | | I²= 0.0%; **Phet**= 0.85 |
| IARC males | 3227 | 4915 | 0.74 | 0.51 - 1.08 | 0.12 |
| IARC females | 1992 | 3095 | 0.78 | 0.49 - 1.26 | 0.32 |

**Fig Y - For each SNP used in the genetic instrument of type 2 diabetes, the funnel plot (A) depicts the weight exerted into the causal effect estimate along the y-axis ($\beta_{GP}/SE_{GD}$), and estimates of the causal effect on RCC odds along the x-axis ($\exp(\beta_{GD}/\beta_{GP})$)*. The leave-one-out histogram (B) reflects the distribution of the causal effect estimate of type 2 diabetes instrument when one of the SNPs is removed.** OR: odds ratio. Funnel plot X-axis is in logarithmic scale. Beta-cell disfunction SNPs and insulin resistance SNPs are depicted as triangles and squares, respectively.

A- Funnel plot

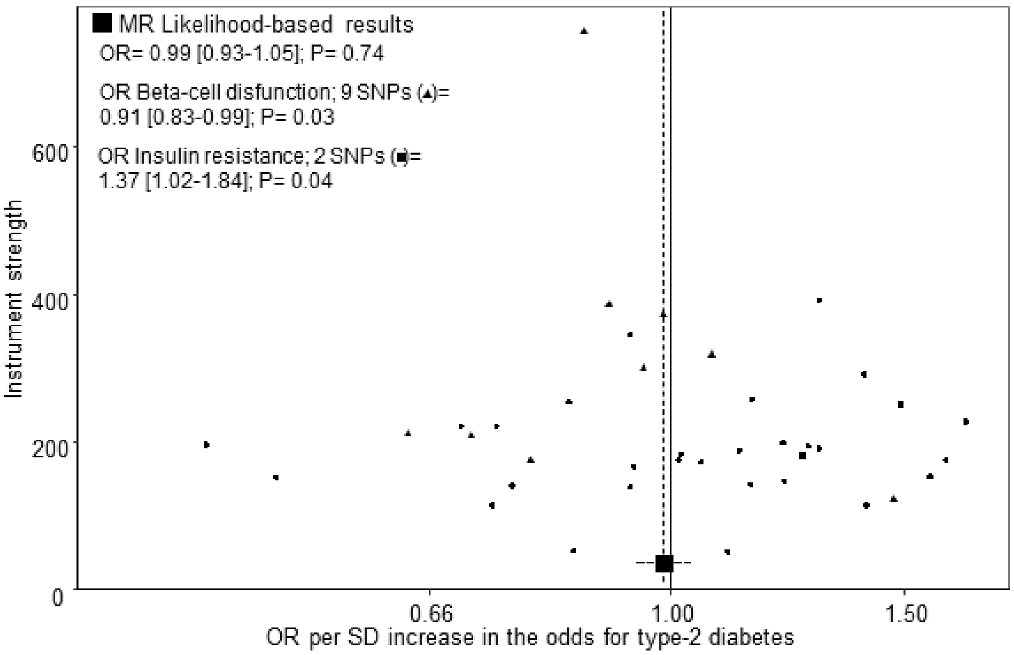


B- Leave-one-out histogram

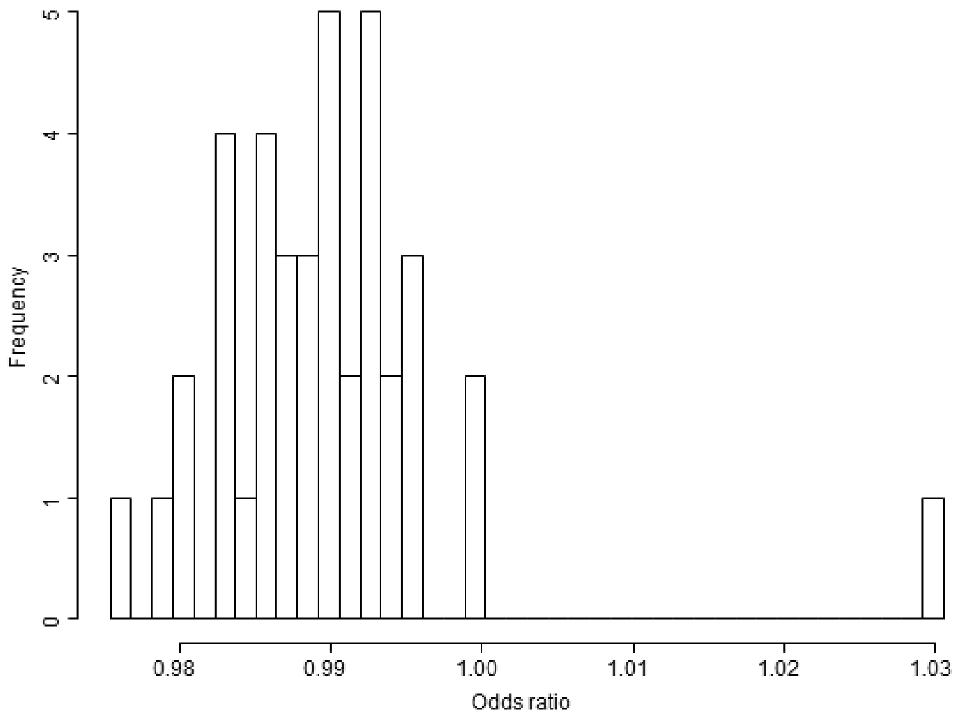

**Fig Z - The leave-one-out histogram reflects the distribution of the causal effect estimate of fasting insulin instrument when one of the SNPs is removed.** OR: odds ratio.

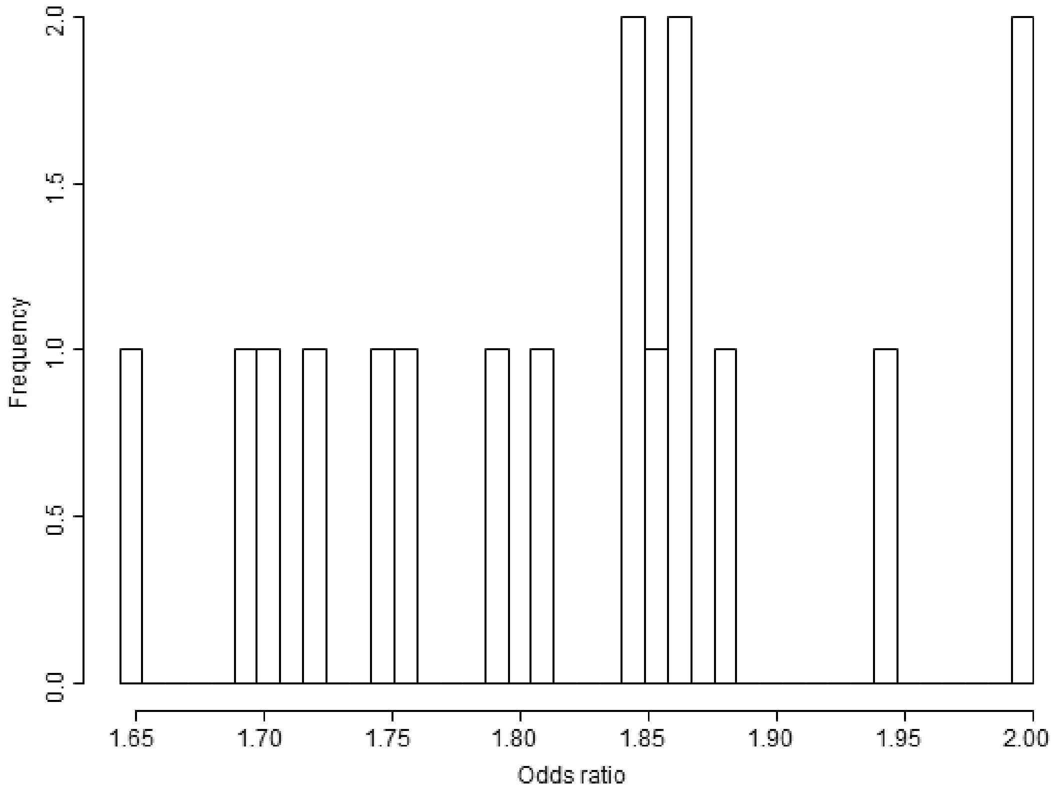

**Fig AA - For each SNP used in the genetic instrument of fasting glucose, the funnel plot (A) depicts the weight exerted into the causal effect estimate along the y-axis ($\beta_{GP}/SE_{GD}$), and estimates of the causal effect on RCC odds along the x-axis ($exp(\beta_{GD}/\beta_{GP})$). The leave-one-out histogram (B) reflects the distribution of the causal effect estimate of fasting glucose instrument when one of the SNPs is removed.** OR: odds ratio. Funnel plot X-axis is in logarithmic scale.

A- Funnel plot

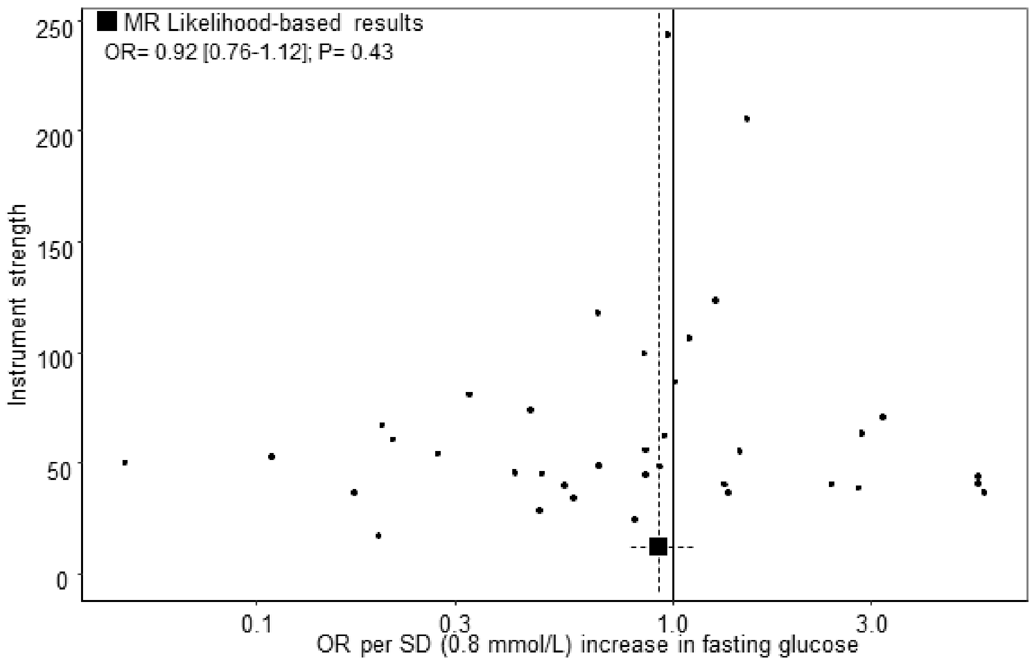


B- Leave-one-out histogram

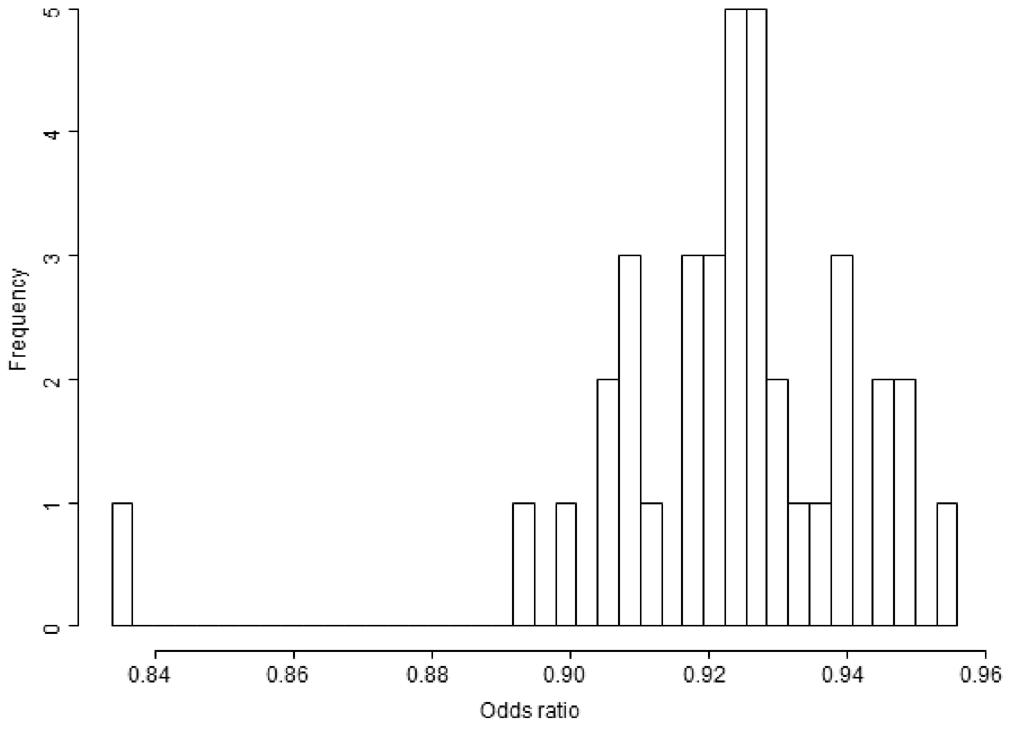

Supplement: S1 Text — (PDF) [file pmed.1002724.s004.pdf]
